# VASA protein and gene expression analysis of human non-obstructive azoospermia and normal by immunohistochemistry, immunocytochemistry, and bioinformatics analysis

**DOI:** 10.1038/s41598-022-22137-9

**Published:** 2022-10-14

**Authors:** Mehdi Amirian, Hossein Azizi, Danial Hashemi Karoii, Thomas Skutella

**Affiliations:** 1 Institute for Anatomy and Cell Biology, Medical Faculty, University of Heidelberg, Im Neuenheimer Feld 307, 69120 Heidelberg, Germany; 2 Faculty of Biotechnology, Amol University of Special Modern Technologies, Amol, Iran

**Keywords:** Biochemistry, Cell biology, Genetics, Molecular biology, Stem cells

## Abstract

VASA, also known as DDX4, is a member of the DEAD-box proteins and an RNA binding protein with an ATP-dependent RNA helicase. The VASA gene expression, which is required for human germ cell development, may lead to infertility. Immunocytochemistry and immunohistochemistry were used to examine the expression of VASA protein in the human testis sections of azoospermic patients, in-vitro and in-silico models. Some studies of fertile humans showed VASA expression in the basal and adluminal compartments of seminiferous tubules. Our Immunocytochemistry and immunohistochemistry in infertile humans showed expression of VASA in the luminal compartments of the seminiferous tubule. The immunohistochemical analysis of three human cases with different levels of non-obstructive azoospermia revealed a higher expression of VASA-positive cells. For this purpose, Enrichr and Shiny Gene Ontology databases were used for pathway enrichment analysis and gene ontology. STRING and Cytoscape online evaluation were applied to predict proteins' functional and molecular interactions and performed to recognize the master genes, respectively. According to the obtained results, the main molecular functions of the up-regulated and downregulated genes include the meiotic cell cycle, RNA binding, and differentiation. STRING and Cytoscape analyses presented seven genes, i.e., DDX5, TNP2, DDX3Y, TDRD6, SOHL2, DDX31, and SYCP3, as the hub genes involved in infertility with VASA co-function and protein–protein interaction. Our findings suggest that VASA and its interacting hub proteins could help determine the pathophysiology of germ cell abnormalities and infertility.

## Introduction

Defective spermatogenesis is a common cause of infertility in human. Understanding normal spermatogenesis is vital for developing human subfertility and infertility. Several RNA-binding proteins are required for germ cell development. Humans, cattle, pigs, chickens, rhesus macaques, and goats have shown VASA expression in germ cells^1^. The VASA gene encodes an ATP-dependent RNA helicase and RNA-binding protein. In human testicular tissues, VASA protein expression can be used to identify spermatogonia, spermatocytes, and spherical spermatids^2,3^. Studying expression patterns in these proteins within different germ cells at various stages can help better understand human spermatogenesis.

Vegetative and invertebrate VASA genes are conserved and expressed in the germ cell lineage. This family includes *C. elegans*, Drosophila, Xenopus, zebrafish, chicken, rainbow trout, rat, and humans^4,5^. The helicase activity is required to translate two mRNAs involved in germ cell migration and development^6,7^. VASA is a component of the germplasm, a poorly understood ribonucleoprotein complex necessary for germ cell determination^8,9^. VASA is essential for Drosophila germ cell formation^10^. Mouse VASA homolog (MVH) knockout mice generate primordial germ cells, although they are infertile due to abnormal proliferation, colonization, and death of zygotene spermatocytes^11,12^. VASA mRNA and protein are produced in germ cells of both sexes throughout their development^13^. In immunohistochemistry, human migrating primordial germ cells have the VASA protein^8,14^. In contrast, the protein is generated in mice after the primordial germ cells reach the genital ridge^15,16^.

Some studies show that Vasa protein in Drosophila was distributed uniformly in the cytoplasm of cells, which proceed as RNA chaperones and linked with the chromatoid body (CB). Another study domesticated that VASA served as CB and transcribed mRNA remaining in spermatozoa when the geneome becomes dormant. VASA is not only required for spermatogenesis, but also for the embryonic stem cells differentiating into primordial germ cells and spermatogonium stem cells. Guo et al. show that higher VASA expression during SSC might be associated with the abnormal differentiation of primordial germ cells or SSC, which leads to the decreasing of production of SSc and decreased sperm production^17^.

Human germ cell development is unknown due to the inaccessibility of early embryonic stages^18^. In addition, most human germ cell development knowledge comes from in vivo studies with mice^19,20^. A lack of internal or external mechanisms that govern meiosis has lowered the success rate of mature gamete geneeration in both mouse and human in vitro systems^21^. Nevertheless, there is a considerable transcriptional silence at specific phases of development, suggesting the vitality of translation regulation^22^. These proteins, found in flies, worms, frogs, mice, and humans, are required for germ cell development, with some being species-specific. Members of multiple human DAZL gene families have previously affected germ cell formation, initiation, and progression in vitro. The VASA gene is expressed in the germline of many model animals, such as flies, worms, frogs, mice, and humans. VASA protein is cytoplasmic and is produced in mammals from premeiotic phases (starting during the migration or after the entrance of germ cells onto the gonadal ridge) until gametogenesis^23,24^.

Our studies aimed to analyze VASA expression in the seminiferous tubules of humans with azoospermic testis in-vivo and in-vitro. Furthermore, we tried to identify VASA protein interaction partners, which could help determine the function of the VASA protein network during germ cell abnormalities and infertility.

## Methods

### Testicular tissue and experimental design

From October 2017 to September 2020, testicular material was collected from 3 adult males with varying medical histories. All human material studies done here were approved by the local ethical committees (University Hospitals of Tübingene, Heidelberg, and Amol), and all human participants provided informed written permission. The patients' ages varied from 23 to 67 years. Experts performed histopathological tests of the testicular tissue utilized in this research at the Department of Pathology (University Clinic, Tubingen). Short-term (2 weeks after matrix selection) SSC cultures and long-term (> 3 months, up to 8 months) human adult germline stem cells (haGSC) cultures from testicular tissues of all three males were examined on gene expression profiles to assess the character of testicular adult stem cells in this investigation. Single cells from various cell groups were initially evaluated on gene expression profiles using a Biomark Real-Time quantitative PCR (qPCR) instrument (Fluidigm), followed by microarray analysis in comparison to hESCs. The Biomark Real-Time quantitative PCR system was used to verify the chosen set of genes from the microarray study. We focused on genes involved with pluripotency and germ cells^25–27^. Table 1 characteristics of men included in the program of in vitro fertilization providing sperm samples analyzed in this study.

**Table 1:** Characteristics of men included in the program of sperm quality and in vitro fertilization providing sperm samples analyzed.

Patient (sample)	Indication	Sperm quality	Fertilization	Pregnancy	Source of sperm
Patient 1	Non-obstructive azoospermia	Rare, motile spermatozoa	+	**−**	Testis
Patient 2	Non-obstructive azoospermia	Rare, motile spermatozoa	+	**−**	Testis
Patient 3	Non-obstructive azoospermia	Rare, motile spermatozoa	+	**−**	Testis

### Isolation and cultivation of haGSCs

The obtained human testicular tissues were mechanically disturbed after the tunica albuginea was removed to separate the tubules. The characterization of isolated testicular cells was carried out in the same method as stated in our previous study^25^. The dissociated tubules were enzymatically digested for 25 min at 38 °C with 700 U/mL collagenase type IV (Sigma), 0.3 mg/mL dispase II (Roche), and 10 g/mL DNase in HBSS buffer with Ca++ and Mg++ (PAA) to yield a single-cell suspension. The digestion was then halted with 10% Embryonic stem (ES) cell-qualified Fetal Bovine Serum (FBS, Gipco). The cell suspension was centrifuged for 10 min at 1000 rpm after passing through a 100 Mm cell strainer. The pellet was rinsed with HBSS buffer containing Ca++ and Mg++ after the supernatant was removed. After washing, the cells were plated into culture dishes (d = 10 cm), coated with 0.1 percent gelatin (Sigma), in haGSC medium consisting of Human Embryonic Stem Cell CultureStemPro (StemPro hESC) medium, 1 percent N2-supplement (Invitrogen), 6 mg/mL D + glucose (Sigma), 5 g/mL bovine serum albumin (Sigma), 1 percent l-glutamine (PA, Sigma). The cells were cultured in this medium for 90 h in a CO_2_ incubator at 37 °C and 5% CO_2_ in the air. After 70 h, half of the culture media was changed with a new culture medium of the same volume, and the cells were cultivated for an additional 4 days. The culture media was carefully removed on day 7, and the testis cell culture was genetly washed with 5 mL DMEM/F12 culture medium with l-glutamine per plate to harvest the germ cells adhered to the monolayer of adherent somatic cells connected to the dish bottom. Pipetting 5 mL of DMEM/F12 culture media was used to repeat this procedure. The cell suspension was spun for 10 min at 1000 rpm after being pooled from 10 culture plates per tissue sample. The pellet was resuspended in 10 mL of Magnetic Activated Cell Sorting (MACS) buffer and centrifuged for 5 min further before being purified using MACS separation (Miltenyi), CD49f-FITC (AbD Serotec), and anti-FITC beads (Miltenyi). Cells were transferred to plates coated with collagene I (5 g/cm^2^, Becton & Dickinson) after MACS separation and incubated at 37 °C for 6 h. Cells were isolated and pelleted at 1000 rpm when they did not adhere to collagene I dishes. The ColNB cells were suspended in media and plated in 12-well plates precoated with laminin (4.4 g/cm^2^, Sigma) at 0.5–1 106 cells per mL each well. The plated ColNB cells were incubated for 1 h at 37 °C, and unbound cells (ColNB/LamNB cells) were serially diluted away from bound cells (LamB cells) and discarded. The LamB cells were washed with 1 mL media twice. Then, the LamB cells were genetly pipetted and plated over an irradiated CF-1 feeder layer on a 12-well plate with haGSC growth media. Every 3–4 days, a half volume of culture media was removed and replaced with a new haGSC culture medium. Spermatogonia proliferated in a diverse manner under these settings. The finest cell cultures were split in half every two to three weeks. It was vital not to dilute the cells too much and maintain the proper cell number in the wells^25,28^.

### Collection of single cells from enriched human spermatogonia stem cell (haSSC) population using a micromanipulation system

To separate the spermatogonia from the associated monolayer of somatic cells or feeder layer in a culture plate, the haSSC cells were washed with culture media in each sample. The cells were resuspended before being transferred to a single cell suspension at the top of a small culture dish (d = 4 cm). The dish's top was put on a prewarmed working platform of a Zeiss inverted microscope equipped with a micromanipulation system. A micromanipulation pipette was used to harvest the cells step by step at a magnification of 20×. The characteristic morphology of short-term cultivated spermatogonia was clearly visible. This was mainly due to their round size, a diameter of 6–12 μm, and high nucleus-to-cytoplasm ratio, which was seen as a distinct little bright cytoplasmic ring between the round nucleus and the outer cell membrane^7,29^.

### Immunohistochemistry staining

Male testis tissue was removed and stored in 4% paraformaldehyde for 24 h at room temperature. Dehydrating testis tissue blocks were cut using a 10-µM-thick microtome. Sections were stored at room temperature on Superfrost Plus slides. All sections were deparaffinized with xylene and rehydrated in ethanol before staining. Non-specific binding was blocked using serum (Gipco)/Triton X-100 in PBS, and immunofluorescence labeling proceeded as stated previously^24,27^. The testis tissue was cryosectioned as described above for Immunohistochemistry (IMH). The tissue was incubated in 10 PBS for 14–16 h before embedding in Tissue Tek. A cryostat was used to split frozen blocks into 14–18 μm pieces (Leica CM 3050S). The slices were maintained at – 30 °C on Superfrost Plus glass slides until the analysis. Before immunofluorescence staining, all frozen sections were dried at room temperature for 45 min. Our study used the Anti-DDX4/MVH antibody from Abcam (ab 13840) to identify VASA expression in immunohistochemistry and immunocytochemistry.

### Immunocytochemistry staining

Cells were grown and treated with 4% paraformaldehyde on 24 plates. Following a PBS rinse, samples were permeabilized with 0.1% Triton/PBS and blocked with 1% BSA/PBS. After removing the blocking solution, the cells were treated with primary antibodies. Following 30 rinsings, the procedure was followed by incubation with species-specific secondary antibodies conjugated with various fluorochromes. Labeled cells were counterstained for 5 min at room temperature with 0.2 g/mL DAPI (4′,6-diamidino-2-phenylindole) before fixing them with Mowiol 4-88 reagent. The absence of all primary antibodies in the sample served as a negative control for all indicators. A confocal Zeiss LSM 700 microscope was used to study the labeled cells, and pictures were captured using a Zeiss LSM-TPMT camera^30^.

### DNA extraction and PCR

The peqGOLD TriFast reagent extracted differentiated ES-like cells for total RNA. This procedure was followed by reverse transcription using the M-MLV Reverse Transcriptase kit. About 2 µL total RNA was used for cDNA synthesis, which was reverse transcribed in 40 mL of water. The Mastercycler gradient machine (Eppendorf, Germany) used single PCR tubes with a reaction volume of 50 µL per sample. We performed 34 cycles of 35 s of denaturation at 95 °C, 35 s of annealing at primer-specific temperatures, and 30 s of extension at 74 °C. A 4 °C cooldown followed a 15-min final extension at 74 °C. In addition, we used an agarose gel in Tris–acetate buffer EDTA. The gel was stained with ethidium bromide at 0.75 g/mL and photographed using a UV transilluminator (INTAS, Germany)^30,31^.

### Search procedure and data preparation for network analysis

A gene database (https://www.ncbi.nlm.nih.gov/gene/) was used to search for spermatogenesis-related datasets (724 genes). Spermatogenesis and "Homo sapiens" were the search terms (porgn: txid9606). The gene expression profile was then saved in an excel spreadsheet, and p 0.05 was used to identify gene interactions and clusters.

### Network analysis of protein–protein interactions (PPI)

The online program STRING^32^ (v.11.5) was used to predict protein–protein biological and functional interactions (https://stringdb.org/). The STRING web tool was used to upload spermatogenesis genes having a substantial function in VASA (DDX4). The master regulators of VASA and signaling pathways relevant to spermatogenesis were emphasized. The highlighted genes were imported into Cytoscape^33^ (version 3.8.2) to further research and visualize protein–protein interaction networks.

### Microarray analysis

Total RNA was isolated from haGSC cultures using the RNeasy Mini Kit (Qiagene), followed by amplification with the MessageAmp aRNA Kit (Ambion). The micromanipulation system collected 150 cells per probe in each sample, which were then transferred directly into 100 RNA direct lysed solution and kept at − 70 °C. The samples were analyzed at the University of Tübingene Hospital's Microarray laboratory in Germany. The Human U133 + 2.0 Genome Oligonucleotide Array was used to analyze gene expression (Affymetrix). We evaluated 724 genes related to spermatogenesis^25^.

### Microarray data normalization and analyses

Data from microarrays were imported into R Statistical Environment version 4.1.2. (accessed in 2021-11-07). The Bioconductor software affy version 3.14 was used to condense the data. A multi-lowess technique—a multi-dimensional version of the lowess normalization approach—was used to perform additional normalizing between samples. A part of the data was put into the IPA Ingenuity tool to assess gene functions and pathways.

### Pathway enrichment analysis and gene ontology (GO) investigation

Enrichr (http://amp.pharm.mssm.edu/Enrichr/), an online software tool for functional gene annotation, was used to study the KEGG (Kyoto encyclopedia of genes and genomes)^1,34^ and Reactom enrichment pathway. To confirm the biological roles of the genes involved in the PPI network of first protein–protein interaction nodes with VASA (DDX4), we have performed functional gene enrichment analysis using the STRING enrichment analysis in the Cytoscape software. The biological process mediated by related infertility genes was highlighted by the ShinyGO tool.

### Network study of protein–protein interactions (PPI)

The online tool Search Tool for the Retrieval of Interacting Genes (STRING v.11.5) was used to determine the functional relationships of proteins (https://stringdb.org/). The STRING tool was used to upload the up-regulated genes that play a key role in germ cells and infertility. PPIs, both known and projected, were emphasized. Infertility-related signaling pathways were highlighted in order to find the master regulator of infertility. The chosen genes were imported into Cytoscape (version 3.9.0) using the CentiScape plugin for further analysis and display of the PPI network.

### Informed consent

By stating that informed consent was obtained, the authors are attesting that the participants were aware of the study's purpose, risks, and benefits. All methods were carried out in accordance with relevant guidelines and regulations. All patients have read and provided information and have had the opportunity to ask questions. They understand that their participation is voluntary and that they are free to withdraw at any time, without giving a reason and without cost. They understand that I will be given a copy of this consent form. They voluntarily agree to take part in this study. All methods were carried out in accordance with relevant guidelines and regulations (See Informed Consent and Ethical Committee approved in the related file).

### Ethics approval and consent to participate

The local ethics councils permitted all tests with human material directed here (Amol University of Special Modern Technologies (Approval code: Ir.ausmt.rec.1400.05) and University Hospitals of Tubingen and Heidelberg).

## Result

### VASA expression in seminiferous tubules by immunohistochemistry

**Figure 1:**
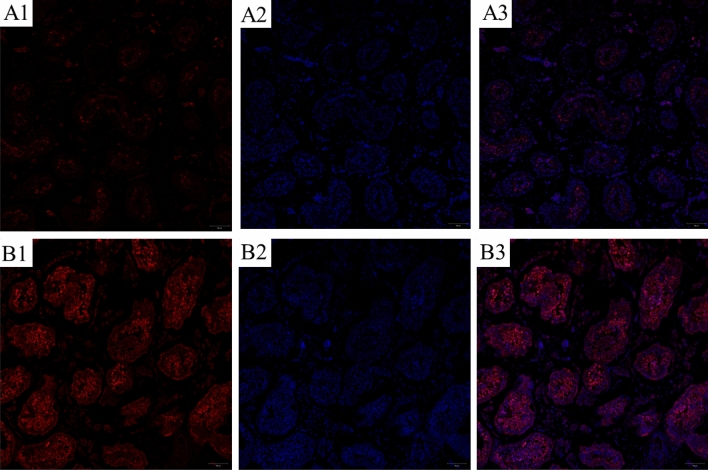
Immunohistochemy image of normal and non-obstructive azoospermia cells. (**A**) Immunohistochemy of non-obstructive azoospermia. (**B**) Immunohistochemy image of normal cells. VASA expression in normal is higher than non-obstructive azoospermia in-vivo; (1) VASA = red, (2) DAPI = blue nuclear staining, and (3) merge (Scale bar: 100 μm).

**Figure 2:**
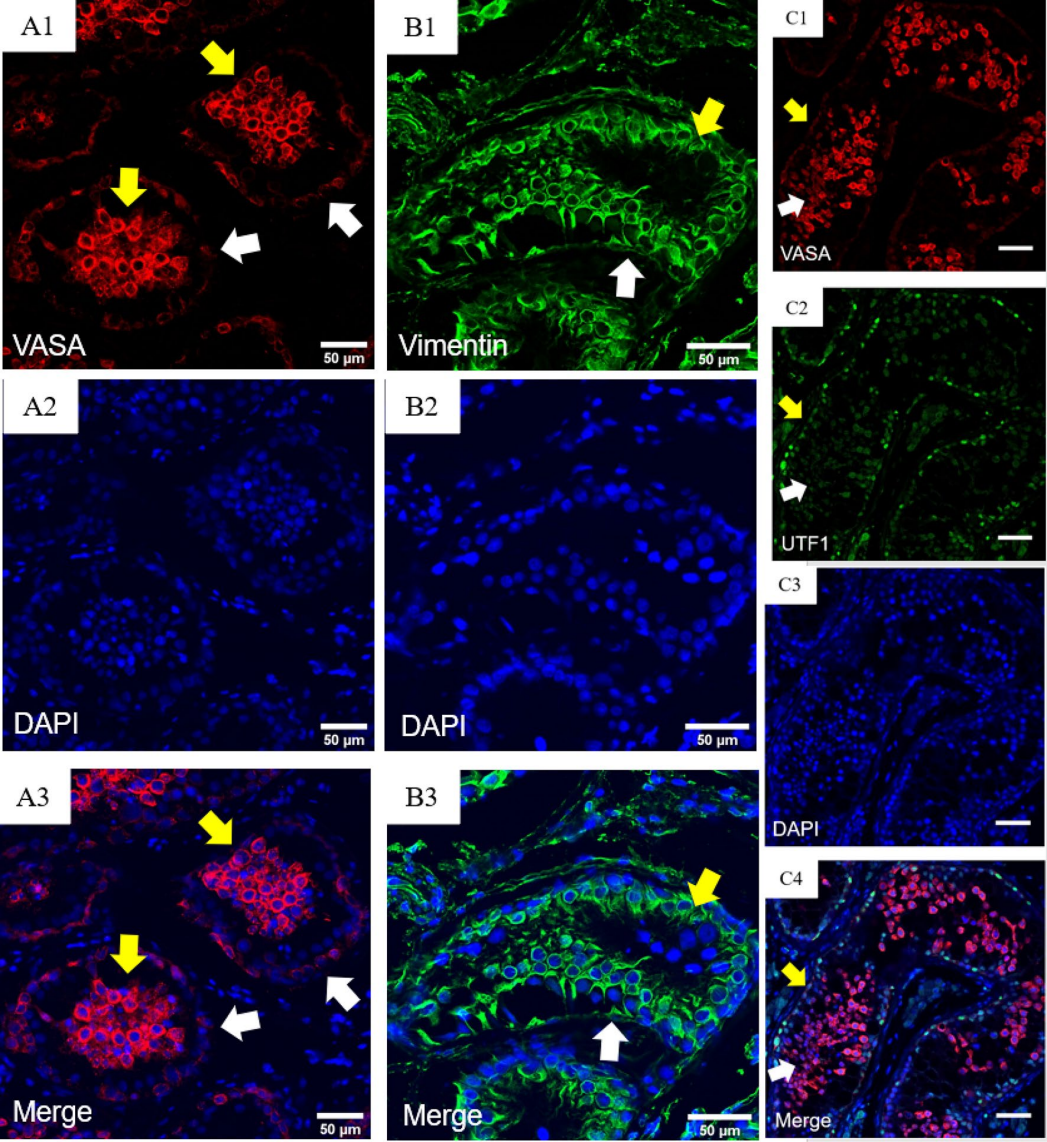
Immunohistochemical analysis of abnormal human testicular tissues from infertile men. (**A**) Low basal and high luminal VASA expression in this case: (A1) VASA = red, (A2) DAPI = blue nuclear staining, and (A3) merging; the white arrow shows undifferentiation spermatogonia, and the yellow arrow shows that differentiation cell (Scale bar: 50 μm). (**B**) Immunohistochemical analysis of abnormal human testicular tissues from infertile men (Sertoli cell). Vimentin expression in Sertoli cell: (B1) vimentin = green, (B2) Dapi = blue nuclear staining, and (B3) merging. The white arrow shows Sertoli cells (Scale bar: 50 μm). (**C**) Immunohistochemical study of infertile men's abnormal human testicular tissues. Low basal and high luminal VASA expression in cases. (C1) VASA = red , (C2) UTF1 = green, (C3) Dapi = blue nuclear staining, and (C4) merging. The white and yellow arrows show undifferentiated and differentiated spermatogonia, respectively (Scale bar: 50 μm).

In this investigation, we initially used an Immunohistochemistry image to investigate VASA expression in a section of human seminiferous tubules in normal and non-obstructive azoospermia cells (Fig. 1A,B). Figure 1 shows that VASA expression in normal is higher than non-obstructive azoospermia in-vivo.Then we used immunohistochemistry to investigate VASA expression in a section of human seminiferous tubules. The analysis revealed high VASA expression in differentiating germ cells in the adluminal and luminal compartments of seminiferous tubules but low VASA expression in undifferentiated cells in the basal compartment. Additionally, immunohistochemistry analysis was performed (Fig. 2A,B). Vimentin-specific marker to distinguish differentiated germ cells. IMH analysis detected high expression of Vimentin and VASA in differentiating germ cells in the adluminal. In the following experiment, we utilized the UTF1-specific marker to distinguish between undifferentiated spermatogonia and differentiating germ cells. UTF1 was highly expressed in undifferentiated spermatogonia, although it was low in differentiating germ cells (Fig. 2C).

### VASA's expression by immunocytochemical analysis

**Figure 3:**
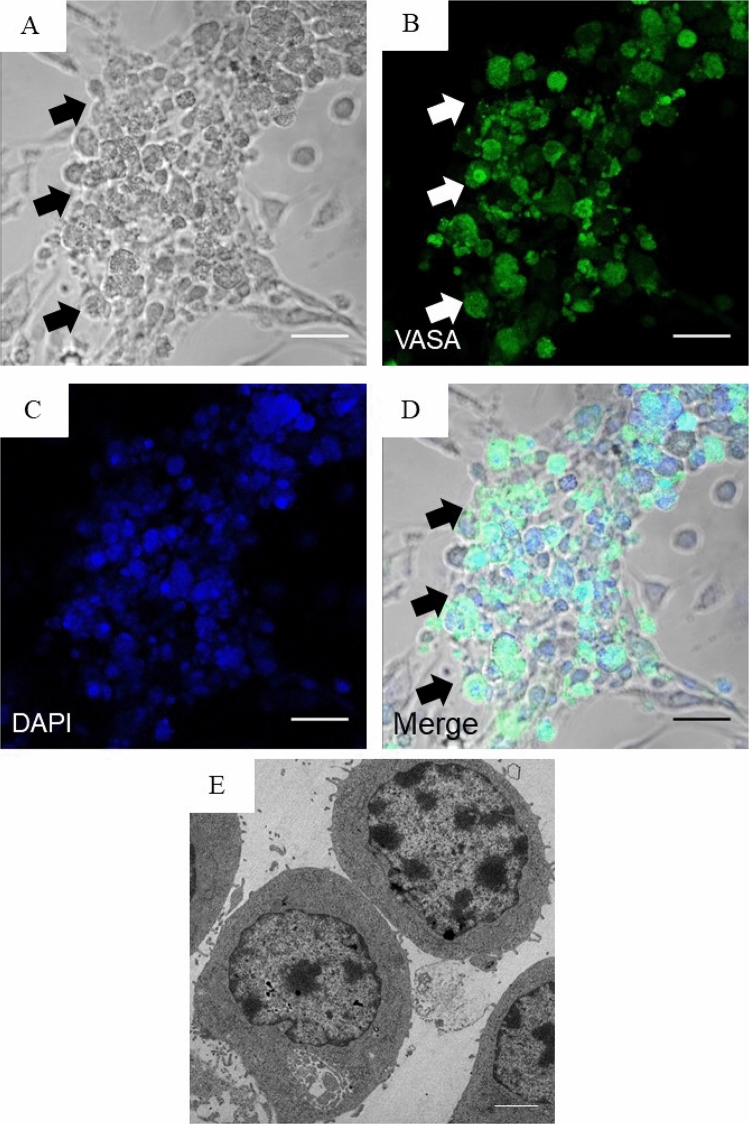
Bright-field images of testicular cells from infertile humans in vitro. Testicular cells from human cases after expansion in culture: (**A**) Infertile human testicular cell expansion in culture, (**B**) Immunohistochemical analysis of VASA in human cells, (**C**) DAPI, (**D**) merge of DAPI and VASA, and (**E**) electron microscopy image representing cell features and the various diameters of the nucleus of infertility human SSCs in vitro. Scale bar (**A**–**D**) (25 μm) and (**E**) (2 μm).

Isolated cells were cultured in the presence of growth factors after enzyme digestion. We used immunocytochemistry (ICC) to examine VASA expression. Images from the confocal scanning UV laser microscope used in ICC analysis revealed that VASA expression was high and changed localization expression in undifferentiated germ cells (Fig. 3).

### Protein–protein interaction visualization of VASA in spermatogenesis process

**Figure 4:**
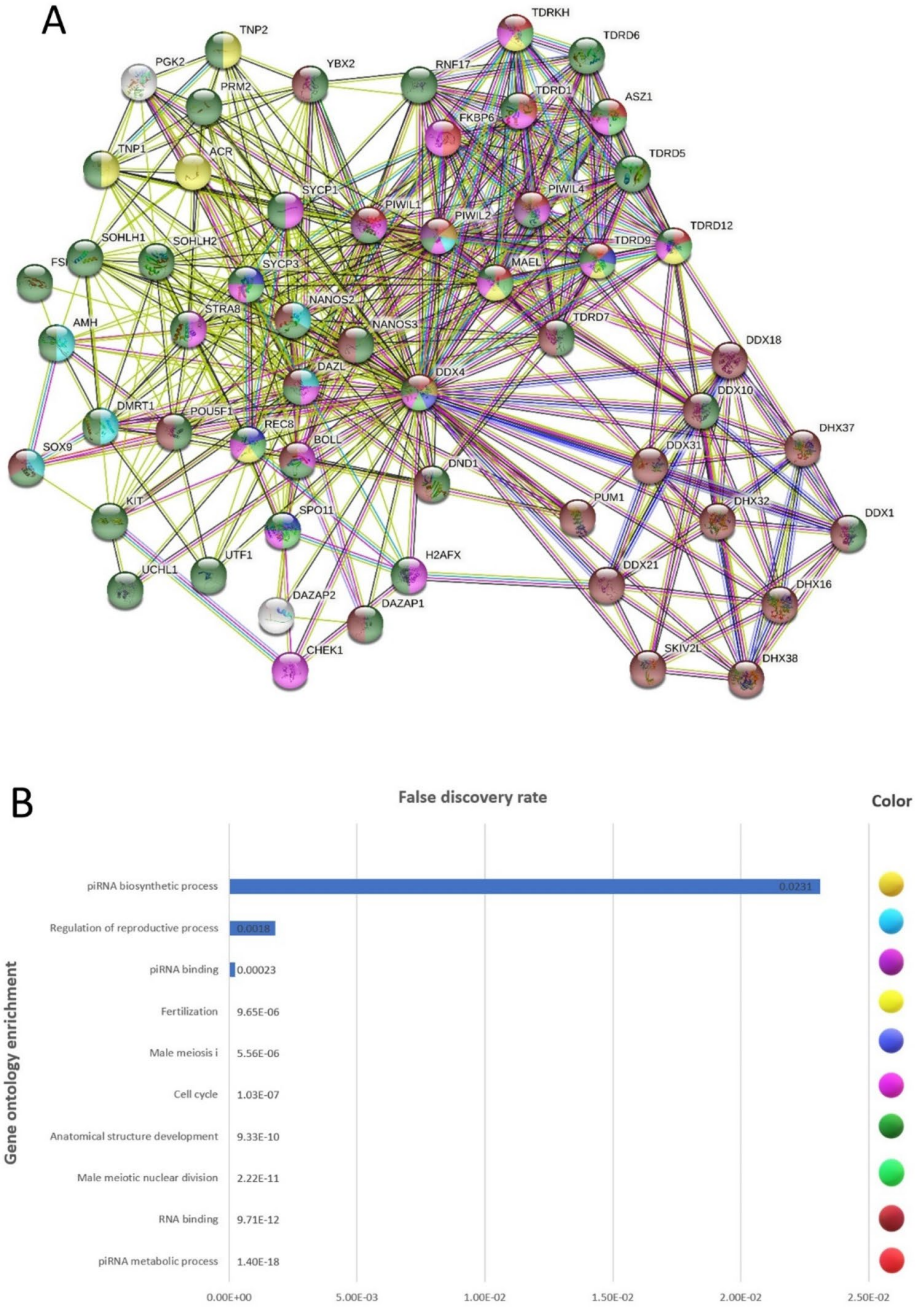
Based on the Reactome and KEGG pathways and the STRING database, the STRING protein–protein interaction network is formed. (**A**) Spermatogenesis-regulated protein–protein interaction revealed significant coexpression. (**B**) Chosen regulation function interaction with VASA. Highlighting nodes by piRNA biosynthetic process, regulation of the reproductive process, piRNA binding, fertilization, male meiosis I, cell cycle, RNA binding, and other functions demonstrating hub genes.

Using the STRING (https://string-db.org/) (v.11.5) database, the protein–protein interaction network was visualized with 728 genes. It showed a close association between interaction and regulated VASA during the spermatogenesis process. We discovered a significant amount of interaction between VASA and DDX5, TNP2, DDX3Y, TDRD6, SOHL2, DDX31, and SYCP3 (Fig. 4A). Reactome (https://reactome.org/) and KEGG choose any spermatogenesis-related signaling pathway to highlight the master regulator of the spermatogenesis pathways. As illustrated in Fig. 4A, the highlighted genes showed a significant correlation.

### Functional enrichments in the PPI network

Enrichment analysis was carried out to recognize enriched biological processes and molecular functions associated with VASA (DDX4) (Fig. 4A). We selected some biological processes based on the purpose of our experiment, including the piRNA biosynthetic process, regulation of the reproductive process, piRNA binding, fertilization, male meiosis I, cell cycle, RNA binding, and other functions demonstrating hub genes (Fig. 4B).

### Microarray analysis

**Figure 5:**
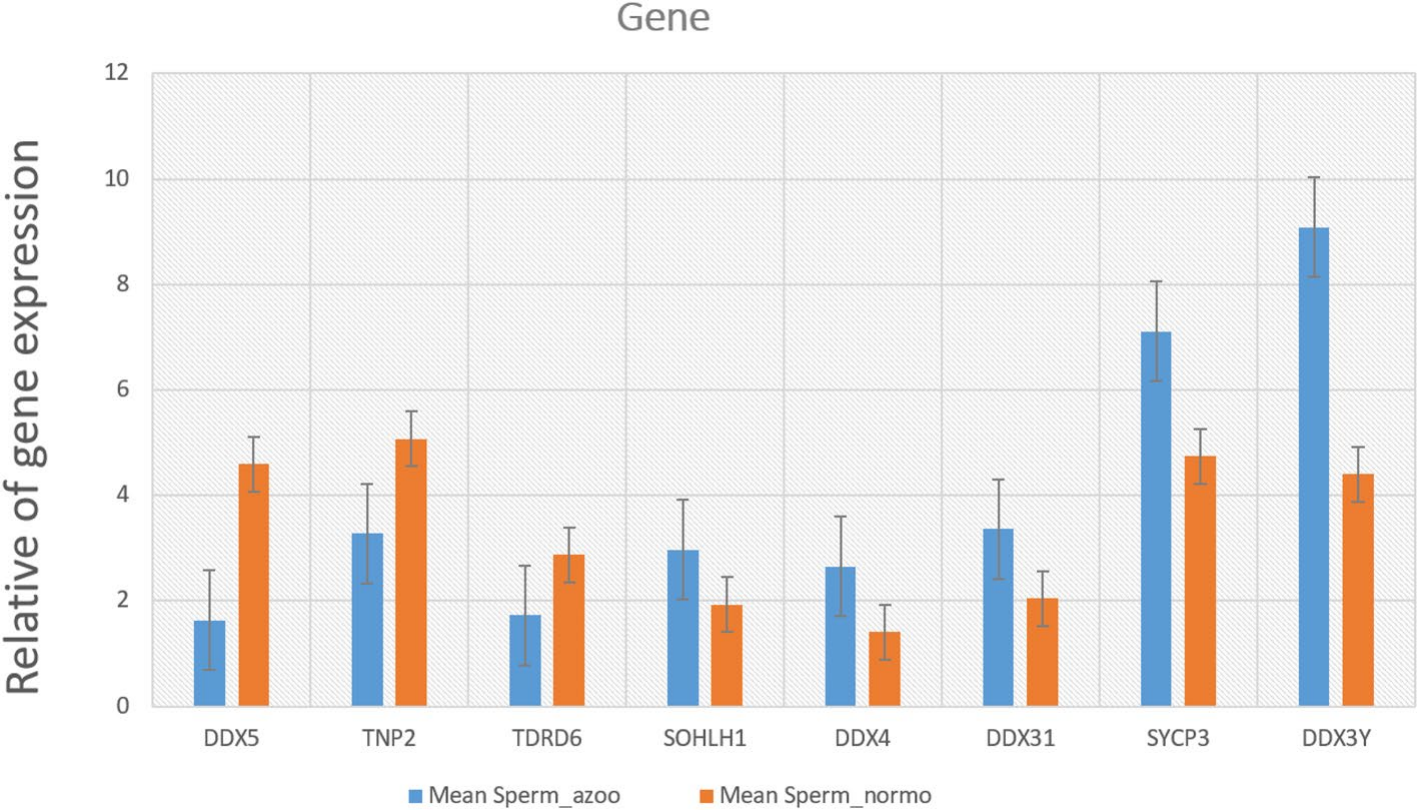
The ratio of infertility genes expression: DDX5, TNP2, DDX3Y, TDRD6, SOHL2, DDX31, and SYCP3 in infertility cell (azoospermia) in comparison to a normal cell. There is a significant decrease in gene expression of DDX5, TNP2, and TDRD6 and an increase in DDX3Y, SOHL2, DDX31, and SYCP3 compared to a normal cell.

According to genes related to spermatogenesis and correlation with VASA, the seven most significant genes (based on fold change > 1 and p-value 0.05) are shown in Supplementary 1, Table 2, and Fig. 5. The ratio of infertility genes expression: DDX5, TNP2, DDX3Y, TDRD6, SOHL2, DDX31, and SYCP3 in infertility cell (azoospermia) in comparison to a normal cell. There is a significant decrease in gene expression of DDX5, TNP2, and TDRD6 and an increase in DDX3Y, SOHL2, DDX31, and SYCP3 compared to a normal cell.

**Table 2:** Gene members of infertility (non-obstructive azoospermia) are differentially expressed in the normal cell.

Gene name	Description	Mean Sperm_azoo	Mean Sperm_normo	Fold change
DDX5	DDX5 regulates the expression of cell cycle genes in undifferentiated spermatogonia post-transcriptionally and is required for cell proliferation and survival	1.629902003	4.594894116	− 2.964
TNP2	Teratozoospermia in mice lacking the transition protein 2	3.276	5.069	− 1.793
TDRD6	Homo sapiens tudor domain containing 6	1.723	2.876	− 1.153
SOHLH1	Homo sapiens spermatogenesis and oogenesis specific basic helix-loop-helix 1	2.969	1.933	1.036
DDX4	Homo sapiens DEAD (Asp-Glu-Ala-Asp) box polypeptide 4	2.652	1.411	1.264
DDX31	Homo sapiens DEAD (Asp-Glu-Ala-Asp) box polypeptide 31	3.359	2.044	1.315
SYCP3	Homo sapiens synaptonemal complex protein 3	7.107	4.745	2.365
DDX3Y	Homo sapiens DEAD (Asp-Glu-Ala-Asp) box polypeptide 3, Y-linked (DDX3Y)	9.087	4.402	4.6856

## Discussion

During development, VASA is expressed in the middle of seminiferous tubules in newborn humans' testicular cords. These cells, which appear to be T1-prospermatogonia, migrate to the seminiferous tubules and produce T2 pre-spermatogonia during the first post-natal week. These cells fill the basement membrane and begin the spermatogenesis pathway throughout the post-pubertal life^35,36^.

We found VASA protein expression in the cells layer of the middle and central testicular cords in the abnormal testis' seminiferous tubule. Also, the results showed a reduction in VASA protein expression throughout spermiogenesis. Some findings suggest that VASA is expressed in the round cell and spermatozoa of the abnormal testis, is missing in SSCs, and is faintly expressed in spermatogonia in neonatal and adult human testis sections^37^.

We predicted protein–protein interactions between VASA and DDX5, TNP2, DDX3Y, TDRD6, SOHL2, DDX31, and SYCP3. DDX5, which regulates the appropriate splicing of critical genes required for spermatogenesis, is one of the VASA-related genes. Furthermore, DDX5 post-transcriptionally controls the expression of cell cycle genes in undifferentiated spermatogonia and is required for cell proliferation^38^, survival, and regulating gene expression programs and activity in undifferentiated spermatogonia^39,40^.

Tdrd6, as a major Chromatoid Body (CB) component, is required for CB synthesis, including numerous other CB proteins^41^. Sohlh1 (spermatogenesis and oogenesis-specific basic helix–loop–helix transcription factor) is another germ-cell-specific transcription factor that is preferentially expressed in spermatogonia and Type A spermatogonia. Sohlh1 deficiency can cause infertility by compromising with spermatogonial development into spermatocytes^42^.

The SYCP3 gene is considered to be a testis-specific gene that forms the core of the synaptonemal complex's lateral elements. It regulates DNA binding to the chromatid axis, sister chromatid cohesion, synapsis, and recombination. DDX3Y is a testis-specific gene that performs as an early spermatogenesis regulator and is expressed from the AZF regions of the Y chromosome^43^. We suggested that DDX5, TNP2, DDX3Y, Tdrd6, SOHL2, DDX31, and SYCP3 gene expression and function may be disturbed by up/down VASA expression.

In contrast to the in vivo observations, we discovered that VASA expression in culture exhibits more significance than in the cells in vivo. This difference might be related to histological alterations in the stem cell compartment, such as the dissociation of feeder cells from Sertoli cells. Somatic Sertoli feeder cells may reduce male germline stem cell preservation and quantity in vitro in mice. Because of these feeder cells in culture, human SSCs are also differentiated into functional sperm^28,29,44^.

As previously reported, the somatic niche influences sex-specific expression in addition to germ cell-specific expression. In the context of predominantly female germ cell gene expression, ectopic VASA expression may affect the translation of proteins like ACROSIN. This observation suggests that ACROSIN expression in non-haploid cells may be altered by the ectopic expression of key meiotic regulators such as the RNA-binding proteins DAZL and VASA^45,46^.

DAZL and VASA both increased advancement during the meiosis. However, ectopic expression of DAZL generated the most haploid cells, with no indication of synergy with ectopic expression of VASA^47^. However, researchers found that both DAZL and VASA overexpression increased the proportion of ACROSIN-positive cells, suggesting that both proteins may be involved in the growth of this late male germ cell marker. Neither ectopic expression of either translational component was associated with alterations in overall gene expression patterns. As previously stated, ectopic DAZL expression seems to be involved in early population formation, maintenance, and meiosis stimulation^48,49^.

In addition, VASA ectopic expression increased ACROSIN-positive cells but was not involved in forming or maintaining early germ cell populations. Erasing the epigenetic imprinting and subsequent re-establishing the sub-specificity is necessary for embryonic development, organogenesis, and viability. Re-establishing epigenetic fingerprints of imprinted loci is a germline-specific process. The maternally imprinted H19 locus showed a significant reduction in the CpG methylation pattern of its differentially methylated region when treating the cells with ectopic production of VASA but not DAZL^50^. Thus, imprint erasure is a VASA specialty for controlling DNA methylation/demethylation. However, further studies should be conducted to investigate the function of potential VASA targets in germline epigenetic reprogramming. Other researchers have discovered that the gonadal niche is crucial for effective meiotic initiation. DAZL and VASA are evolutionarily different RNA-binding proteins in this regard^51^.

Mature spermatozoa are usually thought of as just carrying genetic information. Evidence has shown that mature ejaculated spermatozoa contain a wide range of mRNAs involved in sperm motility, capacitation, and acrosomal response. Microarray techniques detected 268 transcripts in ejaculated spermatozoa of normal fertile men^52^. Also, 149 genes were expressed at higher levels in both testes, which ejaculated spermatozoa using the microarray methods. A recent study discovered that ejaculated spermatozoa messenger RNA was delivered to the egg during fertilization, indicating a role in early human embryo development. Since the mRNAs are unknown in human sperm, ejaculated spermatozoa may be used as a noninvasive surrogate for testing testis-specific infertility^8,29,53^.

In mice with systematic genetic deletions of the VASA gene, males exhibit a reproductive deficiency with a loss of sperm production. The male GSCs die at the zygotene step of the meiosis phases, whereas the ovarian function appears to be normal. It has been observed that VASA is localized in PGCs in mice from embryonic day 12.5 onwards directly after entering the gonadal anlage^16^. Unlike spermatozoa, a previous study found that VASA was expressed in human spermatocytes. We identified VASA mRNA in ejaculated spermatozoa. The present study suggested that VASA protein on the cytoplasmic membrane of spermatozoa heads and tails is significantly downregulated in oligozoospermic men. On the other hand, these data suggest that VASA might effectively diagnose male infertility.

Two previous studies discovered the VASA protein in the cytoplasm of human cells, acting as an RNA chaperone and connected to the chromatid body (CB). According to previous research, one of the major components of the CB is a DEAD-box RNA helicase VASA that belongs to a class of proteins thought to act as RNA chaperones^54^.VASA is required for spermatogenesis and the differentiation of embryonic stem cells into primordial germ cells and spermatogonium stem cells^55^.

Lower spermatogenic cell formation and sperm production may be associated with increased VASA expression during spermatogenesis. The VASA gene was expressed in human ejaculated sperm but not in infertile men with oligozoospermia. Anomalies in this gene's expression have been associated with spermatogenic malfunction, contributing to male infertility. Thus, sperm mRNA analysis may help determine testis function in infertile men^26,56^.

## Conclusion

This research shows that RNA-binding proteins that are evolutionarily distant and divergent may enhance the meiotic development of human germ cells generated in vitro. The findings offer a foundation for further investigation into the details of human meiosis, a program that is surprisingly vulnerable to mistakes that take place in human aneuploidy and illness. Also, it was found that VASA protein is increased throughout the spermatogenesis differentiation phases in an aberrant human germ cell. Our results indicate that VASA and its interacting partners may help identify germ cell defects and infertility etiology. The VASA gene was not expressed in spermatogenesis in vitro, which may be linked to aberrant differentiation of primordial germ cells and male infertility.

## Supplementary Information

Supplementary Table 1.

## Data Availability

The data sets analyzed for the current study are available from the corresponding author on reasonable request.

## Abbreviations

DEAD: Asp-Glu-Ala-Asp
MVH: Mouse VASA homolog
qPCR: Quantitative polymerase chain reaction
PPI: Protein–protein interactions
KEGG: Kyoto encyclopedia of genes and genomes
IMH: Immunohistochemistry
ICC: Immunocytochemical
IGF-1: Insulin-like growth factor 1
bFGF: Basic fibroblast growth factor
