# Supplementary material for: VASA protein and gene expression analysis of human non-obstructive azoospermia and normal by immunohistochemistry, immunocytochemistry, and bioinformatics analysis
Source: Sci Rep. 2022 Oct 14;12:17259. doi: 10.1038/s41598-022-22137-9 (PMC9568577; doi:10.1038/s41598-022-22137-9)
Supplement: Supplementary file 1 — Supplementary Table 1. [file 41598_2022_22137_MOESM1_ESM.docx]

**Supplementary table 1:** According to genes related to spermatogenesis and correlation with VASA, the seven most significant genes (based on fold change > 1 and p-value 0.05) are shown in Supplementary 1, Table 1 and Figure 5. The ratio of infertility genes expression: DDX5, TNP2, DDX3Y, TDRD6, SOHL2, DDX31, and SYCP3 in infertility cells (azoospermia) in comparison to a normal cell.

| **GeneName** | **Description** | **Mean Sperm_azoo** | **Mean Sperm_normo** | **Fold** | **T-Test PV** |
| --- | --- | --- | --- | --- | --- |
| DDX5 | DDX5 regulates expression of cell cycle genes in undifferentiated spermatogonia post-transcriptionally and is required for cell proliferation and survival. | 1.629902 | 4.594894 | -2.96499 | 0.888223 |
| TNP2 | Teratozoospermia in mice lacking the transition protein 2 | 3.276775 | 5.069941 | -1.79317 | 0.888223 |
| TDRD6 | Homo sapiens tudor domain containing 6 | 1.723137 | 2.876935 | -1.1538 | 0.888223 |
| RTN4RL2 | Homo sapiens reticulon 4 receptor-like 2 (RTN4RL2), mRNA [NM_178570] | 4.098711 | 5.064935 | -0.96622 | 0.888223 |
| GJB5 | Homo sapiens gap junction protein, beta 5, 31.1kDa (GJB5), mRNA [NM_005268] | 1.458261 | 2.419705 | -0.96144 | 0.888223 |
| CDH2 | Homo sapiens cadherin 2, type 1, N-cadherin (neuronal) (CDH2), mRNA [NM_001792] | 1.893177 | 2.85047 | -0.95729 | 0.888223 |
| KCNQ2 | Homo sapiens cDNA FLJ60440 complete cds, highly similar to Potassium voltage-gated channel subfamily KQT member 2. [AK293727] | 5.963192 | 6.91652 | -0.95333 | 0.888223 |
| MDGA1 | Homo sapiens MAM domain containing glycosylphosphatidylinositol anchor 1 (MDGA1), mRNA [NM_153487] | 1.399984 | 2.351595 | -0.95161 | 0.888223 |
| RUNX1 | Homo sapiens runt-related transcription factor 1 (RUNX1), transcript variant 2, mRNA [NM_001001890] | 1.992434 | 2.942163 | -0.94973 | 0.888223 |
| CDK5 | Homo sapiens cyclin-dependent kinase 5 (CDK5), transcript variant 1, mRNA [NM_004935] | 1.815506 | 2.762083 | -0.94658 | 0.888223 |
| ENST00000421059 | P450 (cytochrome) oxidoreductase [Source:HGNC Symbol;Acc:9208] [ENST00000421059] | 5.806321 | 6.746041 | -0.93972 | 0.888223 |
| TH | Homo sapiens tyrosine hydroxylase (TH), transcript variant 1, mRNA [NM_199292] | 4.161072 | 5.099199 | -0.93813 | 0.946102 |
| LCK | Homo sapiens lymphocyte-specific protein tyrosine kinase (LCK), transcript variant 2, mRNA [NM_005356] | 2.299773 | 3.236435 | -0.93666 | 0.888223 |
| ACSL4 | Homo sapiens acyl-CoA synthetase long-chain family member 4 (ACSL4), transcript variant 1, mRNA [NM_004458] | 1.526689 | 2.449197 | -0.92251 | 0.888223 |
| MKL1 | Homo sapiens megakaryoblastic leukemia (translocation) 1 (MKL1), mRNA [NM_020831] | 2.051559 | 2.97389 | -0.92233 | 0.888223 |
| A_33_P3285764 | EPH receptor B1 [Source:HGNC Symbol;Acc:3392] [ENST00000467013] | 2.774319 | 3.690323 | -0.916 | 0.888223 |
| ADRB1 | Homo sapiens adrenergic, beta-1-, receptor (ADRB1), mRNA [NM_000684] | 1.638385 | 2.553126 | -0.91474 | 0.888223 |
| HLA-DMA | Homo sapiens major histocompatibility complex, class II, DM alpha (HLA-DMA), mRNA [NM_006120] | 1.353608 | 2.262553 | -0.90895 | 0.888223 |
| CRB1 | Homo sapiens crumbs homolog 1 (Drosophila) (CRB1), transcript variant 1, mRNA [NM_201253] | 1.996388 | 2.903745 | -0.90736 | 0.888223 |
| CARD11 | Homo sapiens cDNA FLJ39820 fis, clone SPLEN2010625. [AK097139] | 1.620398 | 2.527523 | -0.90713 | 0.888223 |
| CACNA1C | Homo sapiens calcium channel, voltage-dependent, L type, alpha 1C subunit (CACNA1C), transcript variant 1, mRNA [NM_199460] | 1.330766 | 2.236904 | -0.90614 | 0.888223 |
| PLXNA2 | Homo sapiens plexin A2 (PLXNA2), mRNA [NM_025179] | 1.377763 | 2.281163 | -0.9034 | 0.888223 |
| ADM | Homo sapiens adrenomedullin (ADM), mRNA [NM_001124] | 6.270611 | 7.171227 | -0.90062 | 0.888223 |
| PAX8 | Homo sapiens paired box 8 (PAX8), transcript variant PAX8A, mRNA [NM_003466] | 1.404272 | 2.290718 | -0.88645 | 0.888223 |
| CYP11A1 | Homo sapiens cytochrome P450, family 11, subfamily A, polypeptide 1 (CYP11A1), nuclear gene encoding mitochondrial protein, transcript variant 1, mRNA [NM_000781] | 3.079328 | 3.964591 | -0.88526 | 0.888223 |
| ENPP1 | Homo sapiens ectonucleotide pyrophosphatase/phosphodiesterase 1 (ENPP1), mRNA [NM_006208] | 3.584446 | 4.466289 | -0.88184 | 0.888223 |
| CAPN5 | Homo sapiens calpain 5 (CAPN5), mRNA [NM_004055] | 4.264165 | 5.142341 | -0.87818 | 0.898433 |
| NKX6-1 | Homo sapiens NK6 homeobox 1 (NKX6-1), mRNA [NM_006168] | 1.645064 | 2.521466 | -0.8764 | 0.888223 |
| POU3F1 | Homo sapiens POU class 3 homeobox 1 (POU3F1), mRNA [NM_002699] | 2.009628 | 2.885918 | -0.87629 | 0.888223 |
| HSP90AA1 | Homo sapiens heat shock protein 90kDa alpha (cytosolic), class A member 1 (HSP90AA1), transcript variant 1, mRNA [NM_001017963] | 4.157951 | 5.033902 | -0.87595 | 0.888223 |
| FARP2 | Homo sapiens FERM, RhoGEF and pleckstrin domain protein 2 (FARP2), mRNA [NM_014808] | 1.606177 | 2.482071 | -0.87589 | 0.888223 |
| LAMA1 | Homo sapiens laminin, alpha 1 (LAMA1), mRNA [NM_005559] | 2.441752 | 3.316783 | -0.87503 | 0.888223 |
| ENST00000393559 | laminin, beta 1 [Source:HGNC Symbol;Acc:6486] [ENST00000393559] | 1.550481 | 2.41642 | -0.86594 | 0.888223 |
| FSTL3 | Homo sapiens follistatin-like 3 (secreted glycoprotein) (FSTL3), mRNA [NM_005860] | 2.914322 | 3.779031 | -0.86471 | 0.888223 |
| EMX1 | Homo sapiens empty spiracles homeobox 1, mRNA (cDNA clone IMAGE:5198260), complete cds. [BC037242] | 2.980023 | 3.841659 | -0.86164 | 0.888223 |
| EPB42 | Homo sapiens erythrocyte membrane protein band 4.2 (EPB42), transcript variant 1, mRNA [NM_000119] | 1.669518 | 2.530508 | -0.86099 | 0.888223 |
| AICDA | Homo sapiens activation-induced cytidine deaminase (AICDA), mRNA [NM_020661] | 1.453955 | 2.311856 | -0.8579 | 0.888223 |
| PPP2R1A | Homo sapiens protein phosphatase 2, regulatory subunit A, alpha (PPP2R1A), transcript variant 1, mRNA [NM_014225] | 2.684938 | 3.540596 | -0.85566 | 0.888223 |
| FABP7 | Homo sapiens fatty acid binding protein 7, brain (FABP7), mRNA [NM_001446] | 1.807877 | 2.661599 | -0.85372 | 0.888246 |
| RAC2 | Homo sapiens ras-related C3 botulinum toxin substrate 2 (rho family, small GTP binding protein Rac2) (RAC2), mRNA [NM_002872] | 1.71394 | 2.565895 | -0.85195 | 0.888223 |
| CLCN2 | Homo sapiens chloride channel 2 (CLCN2), transcript variant 1, mRNA [NM_004366] | 2.762311 | 3.612146 | -0.84983 | 0.888223 |
| RHOB | Homo sapiens ras homolog gene family, member B (RHOB), mRNA [NM_004040] | 1.432719 | 2.2816 | -0.84888 | 0.888223 |
| PTPRC | Homo sapiens protein tyrosine phosphatase, receptor type, C (PTPRC), transcript variant 1, mRNA [NM_002838] | 10.35864 | 11.20655 | -0.8479 | 0.888223 |
| CITED2 | Homo sapiens Cbp/p300-interacting transactivator, with Glu/Asp-rich carboxy-terminal domain, 2 (CITED2), transcript variant 1, mRNA [NM_006079] | 2.350884 | 3.198608 | -0.84772 | 0.888223 |
| PGR | Homo sapiens progesterone receptor (PGR), transcript variant 2, mRNA [NM_000926] | 1.51895 | 2.360018 | -0.84107 | 0.888223 |
| ADAM15 | Homo sapiens ADAM metallopeptidase domain 15 (ADAM15), transcript variant 1, mRNA [NM_207191] | 1.905871 | 2.745718 | -0.83985 | 0.89325 |
| UHMK1 | Homo sapiens U2AF homology motif (UHM) kinase 1 (UHMK1), transcript variant 1, mRNA [NM_175866] | 2.500956 | 3.338551 | -0.8376 | 0.888223 |
| CNTN1 | Homo sapiens contactin 1 (CNTN1), transcript variant 1, mRNA [NM_001843] | 2.23402 | 3.068722 | -0.8347 | 0.888223 |
| SOX15 | Homo sapiens SRY (sex determining region Y)-box 15 (SOX15), mRNA [NM_006942] | 2.569015 | 3.396438 | -0.82742 | 0.888223 |
| SNIP | Homo sapiens SRC kinase signaling inhibitor 1 (SRCIN1), mRNA [NM_025248] | 1.99279 | 2.819698 | -0.82691 | 0.888223 |
| BMP6 | Homo sapiens bone morphogenetic protein 6 (BMP6), mRNA [NM_001718] | 2.277179 | 3.099466 | -0.82229 | 0.888223 |
| B4GALT1 | Homo sapiens UDP-Gal:betaGlcNAc beta 1,4- galactosyltransferase, polypeptide 1 (B4GALT1), mRNA [NM_001497] | 1.611094 | 2.429408 | -0.81831 | 0.888223 |
| NKX2-6 | Homo sapiens NK2 homeobox 6 (NKX2-6), mRNA [NM_001136271] | 2.358364 | 3.175189 | -0.81682 | 0.89938 |
| BCL3 | Homo sapiens B-cell CLL/lymphoma 3 (BCL3), mRNA [NM_005178] | 5.054336 | 5.865263 | -0.81093 | 0.910748 |
| VAV2 | Homo sapiens cDNA FLJ42277 fis, clone TLIVE2002338. [AK124271] | 1.857434 | 2.659932 | -0.8025 | 0.888223 |
| PTPRR | Homo sapiens protein tyrosine phosphatase, receptor type, R (PTPRR), transcript variant 1, mRNA [NM_002849] | 2.385127 | 3.184081 | -0.79895 | 0.888223 |
| BMPR2 | Homo sapiens bone morphogenetic protein receptor, type II (serine/threonine kinase) (BMPR2), mRNA [NM_001204] | 1.735279 | 2.533609 | -0.79833 | 0.888223 |
| A_33_P3332997 | forkhead box A1 [Source:HGNC Symbol;Acc:5021] [ENST00000557418] | 3.577055 | 4.375328 | -0.79827 | 0.888223 |
| LMNA | Homo sapiens lamin A/C (LMNA), transcript variant 2, mRNA [NM_005572] | 4.341494 | 5.13922 | -0.79773 | 0.888223 |
| DOCK7 | Homo sapiens dedicator of cytokinesis 7 (DOCK7), mRNA [NM_033407] | 1.363596 | 2.156538 | -0.79294 | 0.888223 |
| C20orf39 | Homo sapiens synapse differentiation inducing 1 (SYNDIG1), mRNA [NM_024893] | 1.737534 | 2.529725 | -0.79219 | 0.888223 |
| AACS | Homo sapiens acetoacetyl-CoA synthetase (AACS), mRNA [NM_023928] | 2.582273 | 3.365675 | -0.7834 | 0.888223 |
| AMIGO1 | Homo sapiens adhesion molecule with Ig-like domain 1 (AMIGO1), mRNA [NM_020703] | 1.5096 | 2.286602 | -0.777 | 0.888223 |
| PLXNC1 | Homo sapiens plexin C1 (PLXNC1), transcript variant 1, mRNA [NM_005761] | 1.505642 | 2.281811 | -0.77617 | 0.888223 |
| T | Homo sapiens T, brachyury homolog (mouse) (T), mRNA [NM_003181] | 2.029386 | 2.804496 | -0.77511 | 0.888223 |
| STMN2 | Homo sapiens stathmin-like 2 (STMN2), transcript variant 2, mRNA [NM_007029] | 3.668895 | 4.443703 | -0.77481 | 0.888223 |
| RBP4 | Homo sapiens retinol binding protein 4, plasma (RBP4), mRNA [NM_006744] | 2.884123 | 3.658251 | -0.77413 | 0.888223 |
| ABLIM3 | Homo sapiens actin binding LIM protein family, member 3 (ABLIM3), mRNA [NM_014945] | 1.299879 | 2.07359 | -0.77371 | 0.888223 |
| RFX3 | Homo sapiens regulatory factor X, 3 (influences HLA class II expression) (RFX3), transcript variant 2, mRNA [NM_134428] | 3.023358 | 3.793997 | -0.77064 | 0.888223 |
| TP73 | Homo sapiens tumor protein p73 (TP73), transcript variant 1, mRNA [NM_005427] | 3.70596 | 4.471772 | -0.76581 | 0.888223 |
| XRCC2 | Homo sapiens X-ray repair complementing defective repair in Chinese hamster cells 2 (XRCC2), mRNA [NM_005431] | 10.51553 | 11.28026 | -0.76473 | 0.888223 |
| ENST00000360087 | leukocyte receptor tyrosine kinase [Source:HGNC Symbol;Acc:6721] [ENST00000360087] | 1.464319 | 2.226361 | -0.76204 | 0.888223 |
| FOXO4 | Homo sapiens forkhead box O4 (FOXO4), transcript variant 1, mRNA [NM_005938] | 1.43918 | 2.200804 | -0.76162 | 0.888223 |
| HSPA2 | Homo sapiens heat shock 70kDa protein 2 (HSPA2), mRNA [NM_021979] | 1.659385 | 2.419432 | -0.76005 | 0.888223 |
| KITLG | Homo sapiens KIT ligand (KITLG), transcript variant b, mRNA [NM_000899] | 3.427424 | 4.180256 | -0.75283 | 0.888223 |
| KLF7 | Homo sapiens Kruppel-like factor 7 (ubiquitous) (KLF7), mRNA [NM_003709] | 1.394235 | 2.144159 | -0.74992 | 0.888223 |
| HIPK2 | Homo sapiens homeodomain interacting protein kinase 2 (HIPK2), transcript variant 2, mRNA [NM_001113239] | 2.846078 | 3.594492 | -0.74841 | 0.888223 |
| LTBP4 | Homo sapiens latent transforming growth factor beta binding protein 4 (LTBP4), transcript variant 1, mRNA [NM_001042544] | 1.973104 | 2.719181 | -0.74608 | 0.888223 |
| SOAT1 | Homo sapiens sterol O-acyltransferase 1 (SOAT1), nuclear gene encoding mitochondrial protein, transcript variant 688113, mRNA [NM_003101] | 1.871877 | 2.612237 | -0.74036 | 0.888223 |
| QKI | Homo sapiens QKI, KH domain containing, RNA binding (QKI), transcript variant 1, mRNA [NM_006775] | 3.128202 | 3.861813 | -0.73361 | 0.888223 |
| UNC5A | Homo sapiens unc-5 homolog A (C. elegans) (UNC5A), mRNA [NM_133369] | 1.434514 | 2.167159 | -0.73265 | 0.888223 |
| LCE3B | Homo sapiens late cornified envelope 3B (LCE3B), mRNA [NM_178433] | 3.101984 | 3.834047 | -0.73206 | 0.888223 |
| ZEB1 | Homo sapiens zinc finger E-box binding homeobox 1 (ZEB1), transcript variant 1, mRNA [NM_001128128] | 1.470658 | 2.201811 | -0.73115 | 0.888223 |
| CD8A | Homo sapiens CD8a molecule (CD8A), transcript variant 1, mRNA [NM_001768] | 1.398848 | 2.12299 | -0.72414 | 0.888223 |
| PLCB1 | Homo sapiens phospholipase C, beta 1 (phosphoinositide-specific) (PLCB1), transcript variant 2, mRNA [NM_182734] | 2.261536 | 2.985508 | -0.72397 | 0.888223 |
| PAFAH1B1 | Homo sapiens platelet-activating factor acetylhydrolase 1b, regulatory subunit 1 (45kDa) (PAFAH1B1), mRNA [NM_000430] | 1.620106 | 2.343838 | -0.72373 | 0.888223 |
| KLF10 | Homo sapiens Kruppel-like factor 10 (KLF10), transcript variant 1, mRNA [NM_005655] | 1.980964 | 2.704402 | -0.72344 | 0.888223 |
| KCNQ2 | Homo sapiens potassium voltage-gated channel, KQT-like subfamily, member 2 (KCNQ2), transcript variant 5, mRNA [NM_172109] | 5.056465 | 5.778338 | -0.72187 | 0.888223 |
| MAST2 | Homo sapiens microtubule associated serine/threonine kinase 2 (MAST2), mRNA [NM_015112] | 1.436871 | 2.158304 | -0.72143 | 0.888223 |
| MAPK9 | Homo sapiens mitogen-activated protein kinase 9 (MAPK9), transcript variant JNK2-a2, mRNA [NM_002752] | 1.598186 | 2.315397 | -0.71721 | 0.888223 |
| PDE1B | Homo sapiens phosphodiesterase 1B, calmodulin-dependent (PDE1B), transcript variant 1, mRNA [NM_000924] | 1.521392 | 2.234922 | -0.71353 | 0.888223 |
| VAX2 | Homo sapiens ventral anterior homeobox 2 (VAX2), mRNA [NM_012476] | 11.68748 | 12.39918 | -0.7117 | 0.900741 |
| CHRNA1 | Homo sapiens cholinergic receptor, nicotinic, alpha 1 (muscle) (CHRNA1), transcript variant 1, mRNA [NM_001039523] | 1.682455 | 2.392359 | -0.7099 | 0.888223 |
| XRCC5 | Homo sapiens X-ray repair complementing defective repair in Chinese hamster cells 5 (double-strand-break rejoining) (XRCC5), mRNA [NM_021141] | 1.403257 | 2.110943 | -0.70769 | 0.888223 |
| ENST00000376376 | phosphoribosyl transferase domain containing 1 [Source:HGNC Symbol;Acc:23333] [ENST00000376376] | 1.706724 | 2.411394 | -0.70467 | 0.888223 |
| XRCC4 | Homo sapiens X-ray repair complementing defective repair in Chinese hamster cells 4 (XRCC4), transcript variant 3, mRNA [NM_022550] | 1.475256 | 2.176441 | -0.70118 | 0.888223 |
| IFI16 | Homo sapiens interferon, gamma-inducible protein 16 (IFI16), transcript variant 2, mRNA [NM_005531] | 1.620096 | 2.320947 | -0.70085 | 0.888223 |
| SEPT4 | Homo sapiens septin 4 (SEPT4), transcript variant 1, mRNA [NM_004574] | 1.409281 | 2.10866 | -0.69938 | 0.888223 |
| TMSB4X | Homo sapiens thymosin beta 4, X-linked (TMSB4X), mRNA [NM_021109] | 1.623451 | 2.320399 | -0.69695 | 0.888223 |
| S100A7 | Homo sapiens S100 calcium binding protein A7 (S100A7), mRNA [NM_002963] | 3.580945 | 4.273742 | -0.6928 | 0.888223 |
| MYOG | Homo sapiens myogenin (myogenic factor 4) (MYOG), mRNA [NM_002479] | 2.198061 | 2.887331 | -0.68927 | 0.925728 |
| MALT1 | Homo sapiens mucosa associated lymphoid tissue lymphoma translocation gene 1 (MALT1), transcript variant 1, mRNA [NM_006785] | 1.370688 | 2.05854 | -0.68785 | 0.888223 |
| CACNA1I | Homo sapiens calcium channel, voltage-dependent, T type, alpha 1I subunit (CACNA1I), transcript variant 1, mRNA [NM_021096] | 2.70166 | 3.384717 | -0.68306 | 0.908164 |
| FGF18 | Homo sapiens fibroblast growth factor 18 (FGF18), mRNA [NM_003862] | 1.37885 | 2.05923 | -0.68038 | 0.888223 |
| FOXA3 | Homo sapiens forkhead box A3 (FOXA3), mRNA [NM_004497] | 6.263037 | 6.941077 | -0.67804 | 0.94122 |
| IL17A | Homo sapiens interleukin 17A (IL17A), mRNA [NM_002190] | 1.289333 | 1.962838 | -0.6735 | 0.888223 |
| MMP19 | Homo sapiens matrix metallopeptidase 19 (MMP19), transcript variant 1, mRNA [NM_002429] | 5.223363 | 5.896645 | -0.67328 | 0.888223 |
| MDFI | Homo sapiens MyoD family inhibitor (MDFI), mRNA [NM_005586] | 11.26731 | 11.93971 | -0.6724 | 0.888223 |
| F2RL1 | Homo sapiens coagulation factor II (thrombin) receptor-like 1 (F2RL1), mRNA [NM_005242] | 1.505336 | 2.176136 | -0.6708 | 0.888223 |
| MAML1 | mastermind-like 1 (Drosophila) [Source:HGNC Symbol;Acc:13632] [ENST00000503050] | 7.85843 | 8.528962 | -0.67053 | 0.888223 |
| ONECUT1 | Homo sapiens one cut homeobox 1 (ONECUT1), mRNA [NM_004498] | 1.709296 | 2.377328 | -0.66803 | 0.888223 |
| CD86 | Homo sapiens CD86 molecule (CD86), transcript variant 2, mRNA [NM_006889] | 10.33651 | 11.00265 | -0.66614 | 0.888223 |
| SRGAP2 | Homo sapiens SLIT-ROBO Rho GTPase activating protein 2 (SRGAP2), transcript variant 1, mRNA [NM_015326] | 1.743631 | 2.408562 | -0.66493 | 0.888223 |
| LAMA4 | Homo sapiens laminin, alpha 4 (LAMA4), transcript variant 1, mRNA [NM_001105206] | 1.570395 | 2.23321 | -0.66281 | 0.888223 |
| LFNG | Homo sapiens LFNG O-fucosylpeptide 3-beta-N-acetylglucosaminyltransferase (LFNG), transcript variant 2, mRNA [NM_001040168] | 2.517666 | 3.179255 | -0.66159 | 0.888223 |
| ACTB | Homo sapiens actin, beta (ACTB), mRNA [NM_001101] | 1.828666 | 2.489067 | -0.6604 | 0.888223 |
| BCCIP | Homo sapiens BRCA2 and CDKN1A interacting protein (BCCIP), transcript variant C, mRNA [NM_078469] | 1.296709 | 1.956705 | -0.66 | 0.888223 |
| DSP | Homo sapiens desmoplakin (DSP), transcript variant 1, mRNA [NM_004415] | 2.468166 | 3.125503 | -0.65734 | 0.888223 |
| EPHB2 | Homo sapiens EPH receptor B2 (EPHB2), transcript variant 2, mRNA [NM_004442] | 3.251247 | 3.899435 | -0.64819 | 0.895503 |
| ST8SIA4 | Homo sapiens ST8 alpha-N-acetyl-neuraminide alpha-2,8-sialyltransferase 4 (ST8SIA4), transcript variant 2, mRNA [NM_175052] | 1.48385 | 2.130457 | -0.64661 | 0.888223 |
| TRPV1 | Homo sapiens transient receptor potential cation channel, subfamily V, member 1 (TRPV1), transcript variant 3, mRNA [NM_080706] | 2.240878 | 2.886881 | -0.646 | 0.935877 |
| CAPRIN1 | Homo sapiens cell cycle associated protein 1 (CAPRIN1), transcript variant 2, mRNA [NM_203364] | 2.302201 | 2.946182 | -0.64398 | 0.888223 |
| SMO | Homo sapiens smoothened, frizzled family receptor (SMO), mRNA [NM_005631] | 1.346335 | 1.989524 | -0.64319 | 0.888223 |
| FN1 | Homo sapiens fibronectin 1 (FN1), transcript variant 7, mRNA [NM_054034] | 2.108295 | 2.749118 | -0.64082 | 0.888223 |
| ACSL6 | Homo sapiens acyl-CoA synthetase long-chain family member 6 (ACSL6), transcript variant 2, mRNA [NM_001009185] | 3.025426 | 3.664283 | -0.63886 | 0.888223 |
| NEUROG3 | Homo sapiens neurogenin 3 (NEUROG3), mRNA [NM_020999] | 5.055274 | 5.693069 | -0.63779 | 0.888223 |
| LMX1A | Homo sapiens LIM homeobox transcription factor 1, alpha (LMX1A), transcript variant 1, mRNA [NM_177398] | 2.352378 | 2.989791 | -0.63741 | 0.888223 |
| STRA8 | Homo sapiens stimulated by retinoic acid gene 8 homolog (mouse) (STRA8), mRNA [NM_182489] | 1.942282 | 2.578581 | -0.6363 | 0.892771 |
| TSSK6 | Homo sapiens testis-specific serine kinase 6 (TSSK6), mRNA [NM_032037] | 1.880176 | 2.516411 | -0.63623 | 0.888223 |
| POU6F2 | Homo sapiens POU class 6 homeobox 2 (POU6F2), transcript variant 1, mRNA [NM_007252] | 1.60539 | 2.240845 | -0.63545 | 0.888223 |
| YWHAE | Homo sapiens cDNA FLJ53559 complete cds. [AK296555] | 3.830217 | 4.465399 | -0.63518 | 0.909824 |
| SCN1B | Homo sapiens sodium channel, voltage-gated, type I, beta (SCN1B), transcript variant b, mRNA [NM_199037] | 1.855431 | 2.486874 | -0.63144 | 0.902337 |
| ENST00000392193 | microtubule-associated protein 2 [Source:HGNC Symbol;Acc:6839] [ENST00000392193] | 1.699338 | 2.32966 | -0.63032 | 0.888223 |
| ITGA3 | Homo sapiens integrin, alpha 3 (antigen CD49C, alpha 3 subunit of VLA-3 receptor) (ITGA3), transcript variant a, mRNA [NM_002204] | 1.513527 | 2.143411 | -0.62988 | 0.888223 |
| IL6 | interleukin 6 (interferon, beta 2) [Source:HGNC Symbol;Acc:6018] [ENST00000420258] | 1.36489 | 1.991364 | -0.62647 | 0.888223 |
| LCE1C | Homo sapiens late cornified envelope 1C (LCE1C), mRNA [NM_178351] | 5.252562 | 5.877571 | -0.62501 | 0.888223 |
| CLPTM1 | Homo sapiens cleft lip and palate associated transmembrane protein 1 (CLPTM1), transcript variant 2, mRNA [NM_001294] | 1.39838 | 2.022997 | -0.62462 | 0.888223 |
| ABLIM1 | Homo sapiens actin binding LIM protein 1 (ABLIM1), transcript variant 3, mRNA [NM_001003408] | 1.342776 | 1.966067 | -0.62329 | 0.888223 |
| RPS6KA1 | Homo sapiens ribosomal protein S6 kinase, 90kDa, polypeptide 1 (RPS6KA1), transcript variant 1, mRNA [NM_002953] | 2.719532 | 3.341057 | -0.62152 | 0.888223 |
| VSX1 | Homo sapiens visual system homeobox 1 (VSX1), transcript variant 2, mRNA [NM_199425] | 1.597608 | 2.218756 | -0.62115 | 0.888223 |
| TNC | Homo sapiens tenascin C (TNC), mRNA [NM_002160] | 3.101811 | 3.72095 | -0.61914 | 0.888223 |
| CACNA1C | Homo sapiens calcium channel, voltage-dependent, L type, alpha 1C subunit (CACNA1C), transcript variant 18, mRNA [NM_000719] | 2.264916 | 2.883352 | -0.61844 | 0.888223 |
| PTCH1 | patched 1 [Source:HGNC Symbol;Acc:9585] [ENST00000375290] | 5.091437 | 5.708231 | -0.61679 | 0.888223 |
| CCK | Homo sapiens cholecystokinin (CCK), transcript variant 1, mRNA [NM_000729] | 1.806449 | 2.422485 | -0.61604 | 0.919861 |
| HMGB1L1 | Homo sapiens high mobility group box 1 (HMGB1), mRNA [NM_002128] | 1.477081 | 2.092148 | -0.61507 | 0.888223 |
| EID2B | Homo sapiens EP300 interacting inhibitor of differentiation 2B (EID2B), mRNA [NM_152361] | 1.381183 | 1.994862 | -0.61368 | 0.888223 |
| ACSBG1 | acyl-CoA synthetase bubblegum family member 1 [Source:HGNC Symbol;Acc:29567] [ENST00000258873] | 1.352431 | 1.964248 | -0.61182 | 0.888223 |
| MAP2K1 | Homo sapiens mitogen-activated protein kinase kinase 1 (MAP2K1), mRNA [NM_002755] | 1.76386 | 2.374968 | -0.61111 | 0.888223 |
| GLMN | Homo sapiens glomulin, FKBP associated protein (GLMN), mRNA [NM_053274] | 1.303948 | 1.914764 | -0.61082 | 0.888223 |
| KCNQ2 | Q7T267_TETFL (Q7T267) STAT6, partial (3%) [THC2682747] | 1.494267 | 2.104421 | -0.61015 | 0.888223 |
| SOCS3 | Homo sapiens suppressor of cytokine signaling 3 (SOCS3), mRNA [NM_003955] | 1.31524 | 1.923832 | -0.60859 | 0.888223 |
| NFASC | Homo sapiens neurofascin (NFASC), transcript variant 1, mRNA [NM_001005388] | 8.540627 | 9.147881 | -0.60725 | 0.909677 |
| FBXO40 | Homo sapiens F-box protein 40 (FBXO40), mRNA [NM_016298] | 1.91146 | 2.518334 | -0.60687 | 0.888223 |
| ASB1 | Homo sapiens ankyrin repeat and SOCS box containing 1 (ASB1), mRNA [NM_001040445] | 2.092257 | 2.695975 | -0.60372 | 0.888223 |
| KRAS | Homo sapiens v-Ki-ras2 Kirsten rat sarcoma viral oncogene homolog (KRAS), transcript variant a, mRNA [NM_033360] | 1.441546 | 2.044315 | -0.60277 | 0.888223 |
| TNN | Homo sapiens tenascin N (TNN), mRNA [NM_022093] | 2.792 | 3.393348 | -0.60135 | 0.888223 |
| ATP2B2 | Homo sapiens ATPase, Ca++ transporting, plasma membrane 2 (ATP2B2), transcript variant 1, mRNA [NM_001001331] | 1.429857 | 2.030655 | -0.6008 | 0.888223 |
| WASL | Homo sapiens Wiskott-Aldrich syndrome-like (WASL), mRNA [NM_003941] | 1.580772 | 2.181395 | -0.60062 | 0.888223 |
| AFG3L2 | Homo sapiens AFG3 ATPase family gene 3-like 2 (S. cerevisiae) (AFG3L2), nuclear gene encoding mitochondrial protein, mRNA [NM_006796] | 3.034224 | 3.634199 | -0.59997 | 0.888223 |
| PTPRR | Homo sapiens protein tyrosine phosphatase, receptor type, R (PTPRR), transcript variant 1, mRNA [NM_002849] | 1.298773 | 1.897338 | -0.59857 | 0.888223 |
| INPP5E | Homo sapiens inositol polyphosphate-5-phosphatase, 72 kDa (INPP5E), mRNA [NM_019892] | 2.052013 | 2.648707 | -0.59669 | 0.888223 |
| DLK2 | Homo sapiens delta-like 2 homolog (Drosophila) (DLK2), transcript variant 2, mRNA [NM_206539] | 3.767752 | 4.363153 | -0.5954 | 0.888223 |
| CTLA4 | Homo sapiens cytotoxic T-lymphocyte-associated protein 4 (CTLA4), transcript variant 1, mRNA [NM_005214] | 4.271873 | 4.864728 | -0.59286 | 0.913505 |
| CDK5RAP2 | Homo sapiens CDK5 regulatory subunit associated protein 2 (CDK5RAP2), transcript variant 1, mRNA [NM_018249] | 2.94243 | 3.534191 | -0.59176 | 0.941568 |
| EVPL | Homo sapiens envoplakin (EVPL), mRNA [NM_001988] | 2.413668 | 3.004111 | -0.59044 | 0.888223 |
| MLL5 | Homo sapiens myeloid/lymphoid or mixed-lineage leukemia 5 (trithorax homolog, Drosophila) (MLL5), transcript variant 1, mRNA [NM_182931] | 1.454292 | 2.04135 | -0.58706 | 0.888223 |
| THPO | Homo sapiens thrombopoietin (THPO), transcript variant 1, mRNA [NM_000460] | 1.615814 | 2.20012 | -0.58431 | 0.888223 |
| ACIN1 | Homo sapiens apoptotic chromatin condensation inducer 1 (ACIN1), transcript variant 1, mRNA [NM_014977] | 2.363564 | 2.943003 | -0.57944 | 0.888223 |
| CLCN2 | Homo sapiens chloride channel 2 (CLCN2), transcript variant 1, mRNA [NM_004366] | 1.48193 | 2.060033 | -0.5781 | 0.888223 |
| ZNF488 | Homo sapiens zinc finger protein 488 (ZNF488), mRNA [NM_153034] | 1.513203 | 2.09105 | -0.57785 | 0.888223 |
| ITGA5 | Homo sapiens integrin, alpha 5 (fibronectin receptor, alpha polypeptide) (ITGA5), mRNA [NM_002205] | 2.172282 | 2.747514 | -0.57523 | 0.888223 |
| ITM2C | Homo sapiens integral membrane protein 2C (ITM2C), transcript variant 1, mRNA [NM_030926] | 1.543686 | 2.11751 | -0.57382 | 0.888223 |
| ILK | Homo sapiens integrin-linked kinase (ILK), transcript variant 3, mRNA [NM_001014795] | 1.326349 | 1.898425 | -0.57208 | 0.888223 |
| INHA | Homo sapiens inhibin, alpha (INHA), mRNA [NM_002191] | 1.345351 | 1.915946 | -0.57059 | 0.888223 |
| DMD | Homo sapiens dystrophin (DMD), transcript variant Dp40, mRNA [NM_004019] | 1.53251 | 2.102988 | -0.57048 | 0.888223 |
| DYRK3 | Homo sapiens dual-specificity tyrosine-(Y)-phosphorylation regulated kinase 3 (DYRK3), transcript variant 2, mRNA [NM_001004023] | 1.524048 | 2.091264 | -0.56722 | 0.888223 |
| ENST00000394848 | slingshot homolog 2 (Drosophila) [Source:HGNC Symbol;Acc:30580] [ENST00000394848] | 1.370974 | 1.936573 | -0.5656 | 0.888223 |
| KRT3 | Homo sapiens keratin 3 (KRT3), mRNA [NM_057088] | 2.302935 | 2.868431 | -0.5655 | 0.956255 |
| GSK3B | Homo sapiens glycogen synthase kinase 3 beta (GSK3B), transcript variant 1, mRNA [NM_002093] | 2.211512 | 2.774048 | -0.56254 | 0.888223 |
| NEUROD1 | Homo sapiens neurogenic differentiation 1 (NEUROD1), mRNA [NM_002500] | 1.362897 | 1.92516 | -0.56226 | 0.888223 |
| CDK5 | Homo sapiens cyclin-dependent kinase 5 (CDK5), transcript variant 2, mRNA [NM_001164410] | 3.518207 | 4.071615 | -0.55341 | 0.888223 |
| AP2A2 | Homo sapiens adaptor-related protein complex 2, alpha 2 subunit (AP2A2), transcript variant 2, mRNA [NM_012305] | 1.517683 | 2.070988 | -0.5533 | 0.888223 |
| CACNB1 | Homo sapiens calcium channel, voltage-dependent, beta 1 subunit (CACNB1), transcript variant 3, mRNA [NM_199248] | 1.493872 | 2.047072 | -0.5532 | 0.888223 |
| PLCG2 | Homo sapiens phospholipase C, gamma 2 (phosphatidylinositol-specific) (PLCG2), mRNA [NM_002661] | 2.514043 | 3.064338 | -0.55029 | 0.888223 |
| CD40LG | Homo sapiens CD40 ligand (CD40LG), mRNA [NM_000074] | 1.469332 | 2.019376 | -0.55004 | 0.888223 |
| LZTS1 | Homo sapiens leucine zipper, putative tumor suppressor 1 (LZTS1), mRNA [NM_021020] | 2.648856 | 3.196097 | -0.54724 | 0.889741 |
| HDAC7 | histone deacetylase 7 [Source:HGNC Symbol;Acc:14067] [ENST00000459625] | 1.776132 | 2.31925 | -0.54312 | 0.888223 |
| A_33_P3415336 | amyotrophic lateral sclerosis 2 (juvenile) [Source:HGNC Symbol;Acc:443] [ENST00000462747] | 1.368802 | 1.909977 | -0.54117 | 0.888223 |
| TEAD4 | Homo sapiens TEA domain family member 4 (TEAD4), transcript variant 1, mRNA [NM_003213] | 1.385154 | 1.925812 | -0.54066 | 0.888223 |
| CTNNB1 | Homo sapiens catenin (cadherin-associated protein), beta 1, 88kDa (CTNNB1), transcript variant 3, mRNA [NM_001098210] | 1.796987 | 2.335888 | -0.5389 | 0.888223 |
| CLIP3 | Homo sapiens CAP-GLY domain containing linker protein 3 (CLIP3), transcript variant 2, mRNA [NM_015526] | 1.392123 | 1.929242 | -0.53712 | 0.888223 |
| RUNX3 | Homo sapiens runt-related transcription factor 3 (RUNX3), transcript variant 1, mRNA [NM_001031680] | 1.321189 | 1.856016 | -0.53483 | 0.888223 |
| FOXO3 | Homo sapiens forkhead box O3 (FOXO3), transcript variant 1, mRNA [NM_001455] | 1.570457 | 2.10029 | -0.52983 | 0.888223 |
| LAMB2 | Homo sapiens laminin, beta 2 (laminin S) (LAMB2), mRNA [NM_002292] | 2.169722 | 2.698341 | -0.52862 | 0.888223 |
| SLC11A2 | Homo sapiens solute carrier family 11 (proton-coupled divalent metal ion transporters), member 2 (SLC11A2), transcript variant 4, mRNA [NM_000617] | 2.328615 | 2.855403 | -0.52679 | 0.894891 |
| AKT2 | Homo sapiens v-akt murine thymoma viral oncogene homolog 2 (AKT2), transcript variant 1, mRNA [NM_001626] | 1.329161 | 1.854521 | -0.52536 | 0.888223 |
| SCARF1 | Homo sapiens scavenger receptor class F, member 1 (SCARF1), transcript variant 5, mRNA [NM_145352] | 1.304096 | 1.828185 | -0.52409 | 0.888223 |
| CTDSP1 | Homo sapiens CTD (carboxy-terminal domain, RNA polymerase II, polypeptide A) small phosphatase 1 (CTDSP1), transcript variant 1, mRNA [NM_021198] | 1.417455 | 1.940751 | -0.5233 | 0.888223 |
| DLL3 | Homo sapiens delta-like 3 (Drosophila) (DLL3), transcript variant 2, mRNA [NM_203486] | 2.47464 | 2.997176 | -0.52254 | 0.888223 |
| DLD | Homo sapiens dihydrolipoamide dehydrogenase (DLD), mRNA [NM_000108] | 1.337753 | 1.857046 | -0.51929 | 0.888223 |
| WDR62 | Homo sapiens WD repeat domain 62 (WDR62), transcript variant 2, mRNA [NM_173636] | 1.571302 | 2.090333 | -0.51903 | 0.888223 |
| NGEF | neuronal guanine nucleotide exchange factor [Source:HGNC Symbol;Acc:7807] [ENST00000409079] | 1.605541 | 2.123379 | -0.51784 | 0.888223 |
| HOXA1 | Homo sapiens homeobox A1 (HOXA1), transcript variant 1, mRNA [NM_005522] | 1.604817 | 2.119711 | -0.51489 | 0.888223 |
| NDFIP1 | Homo sapiens Nedd4 family interacting protein 1 (NDFIP1), mRNA [NM_030571] | 1.357159 | 1.870398 | -0.51324 | 0.888223 |
| GATA4 | Homo sapiens GATA binding protein 4 (GATA4), mRNA [NM_002052] | 4.441614 | 4.953718 | -0.5121 | 0.888223 |
| CD79A | Homo sapiens CD79a molecule, immunoglobulin-associated alpha (CD79A), transcript variant 1, mRNA [NM_001783] | 3.67303 | 4.184698 | -0.51167 | 0.925029 |
| PLEKHA1 | Homo sapiens pleckstrin homology domain containing, family A (phosphoinositide binding specific) member 1 (PLEKHA1), transcript variant 2, mRNA [NM_001001974] | 1.733586 | 2.244485 | -0.5109 | 0.888223 |
| HHIP | PREDICTED: Homo sapiens hypothetical LOC100505491 (LOC100505491), miscRNA [XR_109840] | 2.09228 | 2.599208 | -0.50693 | 0.888223 |
| HIST2H4B | Homo sapiens histone cluster 2, H4b (HIST2H4B), mRNA [NM_001034077] | 1.694362 | 2.198863 | -0.5045 | 0.888223 |
| NRP1 | Homo sapiens neuropilin 1 (NRP1), transcript variant 3, mRNA [NM_001024629] | 2.432027 | 2.93329 | -0.50126 | 0.910074 |
| RET | Homo sapiens ret proto-oncogene (RET), transcript variant 4, mRNA [NM_020630] | 2.362957 | 2.862665 | -0.49971 | 0.888223 |
| LAMC3 | Homo sapiens laminin, gamma 3 (LAMC3), mRNA [NM_006059] | 2.971436 | 3.470763 | -0.49933 | 0.897441 |
| SART1 | Homo sapiens squamous cell carcinoma antigen recognized by T cells (SART1), mRNA [NM_005146] | 2.8785 | 3.377167 | -0.49867 | 0.888223 |
| HHIP | Homo sapiens hedgehog interacting protein (HHIP), mRNA [NM_022475] | 2.314521 | 2.81193 | -0.49741 | 0.940889 |
| PTPRQ | Homo sapiens protein tyrosine phosphatase, receptor type, Q (PTPRQ), mRNA [NM_001145026] | 1.35823 | 1.854042 | -0.49581 | 0.888223 |
| BCL2 | Homo sapiens B-cell CLL/lymphoma 2 (BCL2), nuclear gene encoding mitochondrial protein, transcript variant beta, mRNA [NM_000657] | 6.71355 | 7.208424 | -0.49487 | 0.888223 |
| ZFHX3 | Homo sapiens zinc finger homeobox 3 (ZFHX3), transcript variant A, mRNA [NM_006885] | 2.03848 | 2.53215 | -0.49367 | 0.888223 |
| PLA2G2A | Homo sapiens phospholipase A2, group IIA (platelets, synovial fluid) (PLA2G2A), transcript variant 1, mRNA [NM_000300] | 1.459892 | 1.952123 | -0.49223 | 0.888223 |
| LHX9 | Homo sapiens LIM homeobox 9 (LHX9), transcript variant 1, mRNA [NM_020204] | 2.458957 | 2.951085 | -0.49213 | 0.888223 |
| GDF11 | Homo sapiens growth differentiation factor 11 (GDF11), mRNA [NM_005811] | 1.597565 | 2.084415 | -0.48685 | 0.888223 |
| NFAM1 | Homo sapiens NFAT activating protein with ITAM motif 1 (NFAM1), mRNA [NM_145912] | 1.618218 | 2.101747 | -0.48353 | 0.888223 |
| ADAM22 | Homo sapiens ADAM metallopeptidase domain 22 (ADAM22), transcript variant 5, mRNA [NM_021721] | 1.272359 | 1.755202 | -0.48284 | 0.888223 |
| CASP6 | Homo sapiens caspase 6, apoptosis-related cysteine peptidase (CASP6), transcript variant alpha, mRNA [NM_001226] | 2.357577 | 2.84018 | -0.4826 | 0.898433 |
| FGF3 | Homo sapiens fibroblast growth factor 3 (FGF3), mRNA [NM_005247] | 6.272329 | 6.754622 | -0.48229 | 0.888223 |
| PDLIM5 | Homo sapiens PDZ and LIM domain 5 (PDLIM5), transcript variant 5, mRNA [NM_001011516] | 1.904849 | 2.387043 | -0.48219 | 0.933862 |
| THC2570780 | TGFB-induced factor homeobox 1 [Source:HGNC Symbol;Acc:11776] [ENST00000551557] | 3.445594 | 3.919688 | -0.47409 | 0.91424 |
| STAT3 | Homo sapiens signal transducer and activator of transcription 3 (acute-phase response factor) (STAT3), transcript variant 3, mRNA [NM_213662] | 1.290446 | 1.764405 | -0.47396 | 0.888223 |
| FOXF1 | Homo sapiens forkhead box F1 (FOXF1), mRNA [NM_001451] | 1.382895 | 1.855435 | -0.47254 | 0.888223 |
| AGGF1 | Homo sapiens angiogenic factor with G patch and FHA domains 1 (AGGF1), mRNA [NM_018046] | 1.409233 | 1.88059 | -0.47136 | 0.888223 |
| CLIC5 | Homo sapiens chloride intracellular channel 5 (CLIC5), nuclear gene encoding mitochondrial protein, transcript variant 1, mRNA [NM_001114086] | 4.345805 | 4.816041 | -0.47024 | 0.888223 |
| ENST00000399256 | Homo sapiens apolipoprotein B (including Ag(x) antigen), mRNA (cDNA clone IMAGE:6270815), complete cds. [BC051278] | 1.534288 | 2.000942 | -0.46665 | 0.888223 |
| ACVR2B | Homo sapiens activin A receptor, type IIB (ACVR2B), mRNA [NM_001106] | 1.279704 | 1.744918 | -0.46521 | 0.888223 |
| VAX1 | Homo sapiens ventral anterior homeobox 1 (VAX1), transcript variant 1, mRNA [NM_001112704] | 1.838607 | 2.302202 | -0.46359 | 0.888223 |
| PPARD | Homo sapiens peroxisome proliferator-activated receptor delta (PPARD), transcript variant 1, mRNA [NM_006238] | 1.787622 | 2.251144 | -0.46352 | 0.888223 |
| DRD1 | Homo sapiens dopamine receptor D1 (DRD1), mRNA [NM_000794] | 1.9225 | 2.383343 | -0.46084 | 0.907746 |
| ARL6 | Homo sapiens ADP-ribosylation factor-like 6 (ARL6), transcript variant 1, mRNA [NM_032146] | 1.29514 | 1.754388 | -0.45925 | 0.888223 |
| GNAO1 | Homo sapiens guanine nucleotide binding protein (G protein), alpha activating activity polypeptide O (GNAO1), transcript variant 2, mRNA [NM_138736] | 1.488913 | 1.947656 | -0.45874 | 0.889199 |
| TTN | Homo sapiens titin (TTN), transcript variant novex-3, mRNA [NM_133379] | 2.267602 | 2.724537 | -0.45693 | 0.888223 |
| RASGRF1 | Homo sapiens Ras protein-specific guanine nucleotide-releasing factor 1 (RASGRF1), transcript variant 1, mRNA [NM_002891] | 1.506045 | 1.962329 | -0.45628 | 0.888223 |
| RBMY1B | Homo sapiens RNA binding motif protein, Y-linked, family 1, member B (RBMY1B), mRNA [NM_001006121] | 1.843077 | 2.299254 | -0.45618 | 0.888223 |
| CALCA | Homo sapiens calcitonin-related polypeptide alpha (CALCA), transcript variant 3, mRNA [NM_001033953] | 1.422096 | 1.877894 | -0.4558 | 0.888223 |
| NP | Homo sapiens purine nucleoside phosphorylase (PNP), mRNA [NM_000270] | 1.501503 | 1.957294 | -0.45579 | 0.888223 |
| BC021296 | tripartite motif containing 16 [Source:HGNC Symbol;Acc:17241] [ENST00000494759] | 1.29752 | 1.752093 | -0.45457 | 0.888223 |
| ERCC2 | Homo sapiens excision repair cross-complementing rodent repair deficiency, complementation group 2 (ERCC2), transcript variant 1, mRNA [NM_000400] | 1.767852 | 2.221833 | -0.45398 | 0.888223 |
| ISL1 | Homo sapiens ISL LIM homeobox 1 (ISL1), mRNA [NM_002202] | 1.678641 | 2.132538 | -0.4539 | 0.888223 |
| TPM1 | Homo sapiens tropomyosin 1 (alpha) (TPM1), transcript variant 3, mRNA [NM_001018004] | 1.475326 | 1.928652 | -0.45333 | 0.888223 |
| CACNA1D | Homo sapiens calcium channel, voltage-dependent, L type, alpha 1D subunit (CACNA1D), transcript variant 1, mRNA [NM_000720] | 2.048945 | 2.501917 | -0.45297 | 0.888223 |
| CHL1 | Homo sapiens cell adhesion molecule with homology to L1CAM (close homolog of L1) (CHL1), mRNA [NM_006614] | 1.288131 | 1.739345 | -0.45121 | 0.888223 |
| NTM | Homo sapiens neurotrimin (NTM), transcript variant 4, mRNA [NM_001144059] | 1.750884 | 2.201643 | -0.45076 | 0.888223 |
| GHRL | Homo sapiens ghrelin/obestatin prepropeptide (GHRL), transcript variant 1, mRNA [NM_016362] | 1.508512 | 1.959259 | -0.45075 | 0.888223 |
| ANK1 | Homo sapiens ankyrin 1, erythrocytic (ANK1), transcript variant 2, mRNA [NM_020477] | 2.210474 | 2.659725 | -0.44925 | 0.923908 |
| C19orf46 | Homo sapiens chromosome 19 open reading frame 46 (C19orf46), mRNA [NM_001039876] | 1.555318 | 2.00314 | -0.44782 | 0.897968 |
| GRIN2A | Homo sapiens glutamate receptor, ionotropic, N-methyl D-aspartate 2A (GRIN2A), transcript variant 1, mRNA [NM_001134407] | 3.485894 | 3.928707 | -0.44281 | 0.888223 |
| MAG | Homo sapiens myelin associated glycoprotein (MAG), transcript variant 1, mRNA [NM_002361] | 3.339099 | 3.781685 | -0.44259 | 0.907895 |
| COL6A3 | Homo sapiens collagen, type VI, alpha 3 (COL6A3), transcript variant 1, mRNA [NM_004369] | 3.292148 | 3.733516 | -0.44137 | 0.888223 |
| CD72 | CD72 molecule [Source:HGNC Symbol;Acc:1696] [ENST00000378431] | 1.311735 | 1.752449 | -0.44071 | 0.888223 |
| CDKN2C | Homo sapiens cyclin-dependent kinase inhibitor 2C (p18, inhibits CDK4) (CDKN2C), transcript variant 2, mRNA [NM_078626] | 1.477411 | 1.918097 | -0.44069 | 0.888223 |
| NTNG2 | Homo sapiens netrin G2 (NTNG2), mRNA [NM_032536] | 3.6068 | 4.046982 | -0.44018 | 0.889199 |
| ABT1 | Homo sapiens activator of basal transcription 1 (ABT1), mRNA [NM_013375] | 2.217702 | 2.657591 | -0.43989 | 0.888223 |
| SPTBN1 | Homo sapiens spectrin, beta, non-erythrocytic 1 (SPTBN1), transcript variant 2, mRNA [NM_178313] | 1.508598 | 1.946468 | -0.43787 | 0.888223 |
| SOX10 | Homo sapiens SRY (sex determining region Y)-box 10 (SOX10), mRNA [NM_006941] | 1.358424 | 1.794666 | -0.43624 | 0.888223 |
| KLK6 | Homo sapiens kallikrein-related peptidase 6 (KLK6), transcript variant B, mRNA [NM_001012964] | 2.566851 | 3.002821 | -0.43597 | 0.888223 |
| BASP1 | Homo sapiens brain abundant, membrane attached signal protein 1 (BASP1), mRNA [NM_006317] | 1.661179 | 2.094483 | -0.4333 | 0.888223 |
| RPS6KB1 | Homo sapiens ribosomal protein S6 kinase, 70kDa, polypeptide 1 (RPS6KB1), mRNA [NM_003161] | 1.410485 | 1.843656 | -0.43317 | 0.888223 |
| HDAC4 | Homo sapiens histone deacetylase 4 (HDAC4), mRNA [NM_006037] | 5.984587 | 6.416694 | -0.43211 | 0.933458 |
| THC2678181 | calcium channel, voltage-dependent, beta 2 subunit [Source:HGNC Symbol;Acc:1402] [ENST00000498816] | 1.351947 | 1.781783 | -0.42984 | 0.888223 |
| KNDC1 | Homo sapiens kinase non-catalytic C-lobe domain (KIND) containing 1 (KNDC1), transcript variant 1, mRNA [NM_152643] | 1.574852 | 2.00342 | -0.42857 | 0.888223 |
| IGF1R | Homo sapiens insulin-like growth factor 1 receptor (IGF1R), mRNA [NM_000875] | 6.753008 | 7.180399 | -0.42739 | 0.905284 |
| PALM | Homo sapiens paralemmin (PALM), transcript variant 1, mRNA [NM_002579] | 1.302812 | 1.729358 | -0.42655 | 0.888223 |
| FYN | Homo sapiens FYN oncogene related to SRC, FGR, YES (FYN), transcript variant 1, mRNA [NM_002037] | 1.572515 | 1.998169 | -0.42565 | 0.888223 |
| C11orf74 | Homo sapiens recombination activating gene 2 (RAG2), transcript variant 1, mRNA [NM_000536] | 1.528428 | 1.953892 | -0.42546 | 0.888223 |
| CBFA2T2 | Homo sapiens core-binding factor, runt domain, alpha subunit 2; translocated to, 2 (CBFA2T2), transcript variant 2, mRNA [NM_005093] | 2.24662 | 2.671772 | -0.42515 | 0.888223 |
| CNTN2 | Homo sapiens contactin 2 (axonal) (CNTN2), mRNA [NM_005076] | 1.551933 | 1.973945 | -0.42201 | 0.888223 |
| SRD5A2 | Homo sapiens steroid-5-alpha-reductase, alpha polypeptide 2 (3-oxo-5 alpha-steroid delta 4-dehydrogenase alpha 2) (SRD5A2), mRNA [NM_000348] | 1.95499 | 2.372282 | -0.41729 | 0.888223 |
| SCN8A | Homo sapiens sodium channel, voltage gated, type VIII, alpha subunit (SCN8A), transcript variant 1, mRNA [NM_014191] | 1.913893 | 2.328724 | -0.41483 | 0.888223 |
| CLASP1 | Homo sapiens cytoplasmic linker associated protein 1 (CLASP1), transcript variant 1, mRNA [NM_015282] | 1.718316 | 2.132867 | -0.41455 | 0.888223 |
| MAFG | Homo sapiens v-maf musculoaponeurotic fibrosarcoma oncogene homolog G (avian) (MAFG), transcript variant 1, mRNA [NM_002359] | 3.24909 | 3.663347 | -0.41426 | 0.925523 |
| IL2RA | Homo sapiens interleukin 2 receptor, alpha (IL2RA), mRNA [NM_000417] | 1.46494 | 1.878369 | -0.41343 | 0.888223 |
| SRF | Homo sapiens serum response factor (c-fos serum response element-binding transcription factor) (SRF), mRNA [NM_003131] | 1.315325 | 1.727768 | -0.41244 | 0.888223 |
| TLN1 | talin 1 [Source:HGNC Symbol;Acc:11845] [ENST00000378192] | 1.429834 | 1.841182 | -0.41135 | 0.888223 |
| PPHLN1 | Homo sapiens periphilin 1 (PPHLN1), transcript variant 5, mRNA [NM_201438] | 3.478614 | 3.888883 | -0.41027 | 0.893492 |
| WNT5B | Homo sapiens wingless-type MMTV integration site family, member 5B (WNT5B), transcript variant 2, mRNA [NM_030775] | 1.966245 | 2.375946 | -0.4097 | 0.888223 |
| TNIK | Homo sapiens TRAF2 and NCK interacting kinase (TNIK), transcript variant 1, mRNA [NM_015028] | 1.355402 | 1.764595 | -0.40919 | 0.888223 |
| NFIB | Homo sapiens nuclear factor I/B (NFIB), transcript variant 3, mRNA [NM_005596] | 1.415014 | 1.824056 | -0.40904 | 0.888223 |
| COL9A1 | Homo sapiens collagen, type IX, alpha 1 (COL9A1), transcript variant 1, mRNA [NM_001851] | 1.703024 | 2.108634 | -0.40561 | 0.896309 |
| RPL24 | Homo sapiens ribosomal protein L24 (RPL24), mRNA [NM_000986] | 1.561118 | 1.965744 | -0.40463 | 0.889718 |
| STK24 | Homo sapiens serine/threonine kinase 24 (STK24), transcript variant 2, mRNA [NM_001032296] | 1.276466 | 1.680189 | -0.40372 | 0.888223 |
| NKX2-2 | Homo sapiens NK2 homeobox 2 (NKX2-2), mRNA [NM_002509] | 1.291609 | 1.694981 | -0.40337 | 0.888223 |
| AGER | Homo sapiens advanced glycosylation end product-specific receptor (AGER), transcript variant 1, mRNA [NM_001136] | 1.309545 | 1.712061 | -0.40252 | 0.888223 |
| HGF | Homo sapiens hepatocyte growth factor (hepapoietin A; scatter factor) (HGF), transcript variant 2, mRNA [NM_001010931] | 1.315463 | 1.716234 | -0.40077 | 0.888223 |
| IL15RA | Homo sapiens interleukin 15 receptor, alpha (IL15RA), transcript variant 2, mRNA [NM_172200] | 2.546956 | 2.944907 | -0.39795 | 0.907874 |
| CHST3 | Homo sapiens carbohydrate (chondroitin 6) sulfotransferase 3 (CHST3), mRNA [NM_004273] | 1.443421 | 1.840918 | -0.3975 | 0.888223 |
| ZCCHC11 | Homo sapiens zinc finger, CCHC domain containing 11 (ZCCHC11), transcript variant 1, mRNA [NM_001009881] | 1.295923 | 1.692598 | -0.39667 | 0.888223 |
| ERCC1 | Homo sapiens excision repair cross-complementing rodent repair deficiency, complementation group 1 (includes overlapping antisense sequence) (ERCC1), transcript variant 1, mRNA [NM_202001] | 1.617517 | 2.013396 | -0.39588 | 0.888223 |
| PLXNA4 | Homo sapiens plexin A4 (PLXNA4), transcript variant 2, mRNA [NM_181775] | 1.30067 | 1.696498 | -0.39583 | 0.888223 |
| HPS1 | Homo sapiens Hermansky-Pudlak syndrome 1 (HPS1), transcript variant 1, mRNA [NM_000195] | 3.854749 | 4.249822 | -0.39507 | 0.888223 |
| CDH23 | Homo sapiens cadherin-related 23 (CDH23), transcript variant 2, mRNA [NM_052836] | 2.714318 | 3.108625 | -0.39431 | 0.916701 |
| CTNND1 | Homo sapiens catenin (cadherin-associated protein), delta 1 (CTNND1), transcript variant 3, mRNA [NM_001331] | 1.548677 | 1.942787 | -0.39411 | 0.888223 |
| ADRA2B | Homo sapiens adrenergic, alpha-2B-, receptor (ADRA2B), mRNA [NM_000682] | 4.110107 | 4.504032 | -0.39392 | 0.919136 |
| NCK2 | Homo sapiens NCK adaptor protein 2 (NCK2), transcript variant 1, mRNA [NM_003581] | 1.463477 | 1.856943 | -0.39347 | 0.888223 |
| MEN1 | Homo sapiens multiple endocrine neoplasia I (MEN1), transcript variant e1E, mRNA [NM_130803] | 1.923492 | 2.31634 | -0.39285 | 0.888223 |
| ERBB3 | Homo sapiens v-erb-b2 erythroblastic leukemia viral oncogene homolog 3 (avian) (ERBB3), transcript variant 1, mRNA [NM_001982] | 5.003441 | 5.39475 | -0.39131 | 0.888223 |
| ABLIM2 | Homo sapiens actin binding LIM protein family, member 2 (ABLIM2), transcript variant 1, mRNA [NM_001130083] | 1.320111 | 1.710754 | -0.39064 | 0.888223 |
| SPAG6 | BC030585 sperm associated antigen 6, isoform 2 {Homo sapiens} (exp=-1; wgp=0; cg=0), partial (31%) [THC2537936] | 1.327901 | 1.718544 | -0.39064 | 0.888223 |
| NOTCH1 | Homo sapiens notch 1 (NOTCH1), mRNA [NM_017617] | 5.366593 | 5.756734 | -0.39014 | 0.888223 |
| FAS | Homo sapiens Fas (TNF receptor superfamily, member 6) (FAS), transcript variant 1, mRNA [NM_000043] | 2.501944 | 2.889999 | -0.38805 | 0.888223 |
| GRIN2A | Homo sapiens glutamate receptor, ionotropic, N-methyl D-aspartate 2A (GRIN2A), transcript variant 3, mRNA [NM_001134408] | 1.620318 | 2.008029 | -0.38771 | 0.888223 |
| INPP5D | Homo sapiens inositol polyphosphate-5-phosphatase, 145kDa (INPP5D), transcript variant 1, mRNA [NM_001017915] | 1.888414 | 2.276047 | -0.38763 | 0.888223 |
| CAV3 | Homo sapiens caveolin 3 (CAV3), transcript variant 2, mRNA [NM_001234] | 1.634249 | 2.021787 | -0.38754 | 0.888223 |
| GFRA3 | Homo sapiens GDNF family receptor alpha 3 (GFRA3), mRNA [NM_001496] | 1.873519 | 2.260229 | -0.38671 | 0.888223 |
| SEP15 | Homo sapiens 15 kDa selenoprotein (SEP15), transcript variant 1, mRNA [NM_004261] | 1.414994 | 1.799338 | -0.38434 | 0.888223 |
| ENST00000399010 | tetratricopeptide repeat domain 3 [Source:HGNC Symbol;Acc:12393] [ENST00000399010] | 1.540609 | 1.924788 | -0.38418 | 0.888223 |
| KLF1 | Homo sapiens Kruppel-like factor 1 (erythroid) (KLF1), mRNA [NM_006563] | 1.326134 | 1.709466 | -0.38333 | 0.888223 |
| DPYSL5 | Homo sapiens dihydropyrimidinase-like 5 (DPYSL5), mRNA [NM_020134] | 2.190125 | 2.57256 | -0.38244 | 0.888223 |
| DND1 | Homo sapiens dead end homolog 1 (zebrafish) (DND1), mRNA [NM_194249] | 1.505906 | 1.888325 | -0.38242 | 0.888223 |
| KRT14 | Homo sapiens keratin 14 (KRT14), mRNA [NM_000526] | 1.801981 | 2.183849 | -0.38187 | 0.894216 |
| EFHD1 | Homo sapiens EF-hand domain family, member D1 (EFHD1), transcript variant 1, mRNA [NM_025202] | 3.362449 | 3.744271 | -0.38182 | 0.888223 |
| AR | Homo sapiens androgen receptor (AR), transcript variant 1, mRNA [NM_000044] | 2.674891 | 3.055633 | -0.38074 | 0.893318 |
| EPHA8 | Homo sapiens EPH receptor A8 (EPHA8), transcript variant 1, mRNA [NM_020526] | 1.483106 | 1.861813 | -0.37871 | 0.888223 |
| TRIM10 | Homo sapiens tripartite motif containing 10 (TRIM10), transcript variant 2, mRNA [NM_052828] | 1.30679 | 1.683019 | -0.37623 | 0.888223 |
| TGIF2 | Homo sapiens TGFB-induced factor homeobox 2 (TGIF2), transcript variant 2, mRNA [NM_021809] | 1.486312 | 1.86173 | -0.37542 | 0.888223 |
| VWC2 | Homo sapiens von Willebrand factor C domain containing 2 (VWC2), mRNA [NM_198570] | 1.405469 | 1.780772 | -0.3753 | 0.888223 |
| EGFR | Homo sapiens epidermal growth factor receptor (EGFR), transcript variant 2, mRNA [NM_201282] | 3.219906 | 3.593745 | -0.37384 | 0.89287 |
| MAPK8IP2 | Homo sapiens mitogen-activated protein kinase 8 interacting protein 2 (MAPK8IP2), transcript variant 1, mRNA [NM_012324] | 2.52966 | 2.903178 | -0.37352 | 0.895701 |
| GFI1 | Homo sapiens growth factor independent 1 transcription repressor (GFI1), transcript variant 1, mRNA [NM_005263] | 1.594107 | 1.967587 | -0.37348 | 0.888223 |
| ITGA1 | Homo sapiens integrin, alpha 1 (ITGA1), mRNA [NM_181501] | 1.415654 | 1.789125 | -0.37347 | 0.888223 |
| PTPRM | Homo sapiens protein tyrosine phosphatase, receptor type, M (PTPRM), transcript variant 2, mRNA [NM_002845] | 1.509878 | 1.883299 | -0.37342 | 0.888223 |
| CASP8 | Homo sapiens caspase 8, apoptosis-related cysteine peptidase (CASP8), transcript variant B, mRNA [NM_033355] | 1.334566 | 1.707872 | -0.37331 | 0.888223 |
| WNT4 | Homo sapiens wingless-type MMTV integration site family, member 4 (WNT4), mRNA [NM_030761] | 1.447262 | 1.818326 | -0.37106 | 0.888223 |
| BMP7 | Homo sapiens bone morphogenetic protein 7 (BMP7), mRNA [NM_001719] | 1.313782 | 1.683236 | -0.36945 | 0.888223 |
| CAPRIN1 | Homo sapiens cell cycle associated protein 1 (CAPRIN1), transcript variant 1, mRNA [NM_005898] | 1.412956 | 1.779773 | -0.36682 | 0.888223 |
| F11R | Homo sapiens F11 receptor (F11R), mRNA [NM_016946] | 1.343927 | 1.710708 | -0.36678 | 0.888223 |
| GREM1 | Homo sapiens gremlin 1 (GREM1), transcript variant 2, mRNA [NM_001191323] | 2.266083 | 2.63271 | -0.36663 | 0.932263 |
| C12orf52 | Homo sapiens chromosome 12 open reading frame 52 (C12orf52), mRNA [NM_032848] | 4.955345 | 5.317998 | -0.36265 | 0.910985 |
| KRT19 | Homo sapiens keratin 19 (KRT19), mRNA [NM_002276] | 5.892959 | 6.254596 | -0.36164 | 0.966947 |
| LAMB1 | Homo sapiens laminin, beta 1, mRNA (cDNA clone IMAGE:4889995), containing frame-shift errors. [BC044633] | 2.408301 | 2.768026 | -0.35973 | 0.888223 |
| FGFR1 | Homo sapiens fibroblast growth factor receptor 1 (FGFR1), transcript variant 1, mRNA [NM_023110] | 1.331425 | 1.690991 | -0.35957 | 0.888223 |
| LRRK2 | Homo sapiens leucine-rich repeat kinase 2 (LRRK2), mRNA [NM_198578] | 1.304766 | 1.6639 | -0.35913 | 0.888223 |
| KNDC1 | Homo sapiens kinase non-catalytic C-lobe domain (KIND) containing 1 (KNDC1), transcript variant 1, mRNA [NM_152643] | 1.283769 | 1.641892 | -0.35812 | 0.888223 |
| PAX2 | Homo sapiens paired box 2 (PAX2), transcript variant c, mRNA [NM_003988] | 1.342243 | 1.700033 | -0.35779 | 0.888223 |
| DLG2 | Homo sapiens cDNA FLJ45207 fis, clone BRCAN2010665, highly similar to Channel associated protein of synapse-110. [AK127150] | 1.342234 | 1.699995 | -0.35776 | 0.888223 |
| ASCL1 | Homo sapiens achaete-scute complex homolog 1 (Drosophila) (ASCL1), mRNA [NM_004316] | 1.903352 | 2.261031 | -0.35768 | 0.889199 |
| FLG | Homo sapiens filaggrin (FLG), mRNA [NM_002016] | 2.964139 | 3.320767 | -0.35663 | 0.891385 |
| MAPK12 | Homo sapiens mitogen-activated protein kinase 12 (MAPK12), mRNA [NM_002969] | 1.318372 | 1.673197 | -0.35482 | 0.888223 |
| ENST00000369477 | CD2 molecule [Source:HGNC Symbol;Acc:1639] [ENST00000369477] | 2.658269 | 3.013066 | -0.3548 | 0.916333 |
| DCX | Homo sapiens doublecortin (DCX), transcript variant 5, mRNA [NM_001195553] | 1.458745 | 1.812382 | -0.35364 | 0.888223 |
| HBZ | Homo sapiens hemoglobin, zeta (HBZ), mRNA [NM_005332] | 1.56142 | 1.914293 | -0.35287 | 0.909677 |
| DPYSL2 | Homo sapiens dihydropyrimidinase-like 2 (DPYSL2), transcript variant 2, mRNA [NM_001386] | 1.322566 | 1.675187 | -0.35262 | 0.888223 |
| FGF9 | Homo sapiens fibroblast growth factor 9 (glia-activating factor) (FGF9), mRNA [NM_002010] | 2.088354 | 2.439318 | -0.35096 | 0.918333 |
| FOXD1 | Homo sapiens forkhead box D1 (FOXD1), mRNA [NM_004472] | 1.342697 | 1.693525 | -0.35083 | 0.888223 |
| COL5A2 | Homo sapiens collagen, type V, alpha 2 (COL5A2), mRNA [NM_000393] | 1.509927 | 1.860594 | -0.35067 | 0.889199 |
| ALAS2 | Homo sapiens aminolevulinate, delta-, synthase 2 (ALAS2), nuclear gene encoding mitochondrial protein, transcript variant 1, mRNA [NM_000032] | 2.115564 | 2.466198 | -0.35063 | 0.899613 |
| CTLA4 | Homo sapiens cytotoxic T-lymphocyte-associated protein 4 (CTLA4), transcript variant 1, mRNA [NM_005214] | 1.359782 | 1.709891 | -0.35011 | 0.888223 |
| NF1 | Homo sapiens neurofibromin 1 (NF1), transcript variant 2, mRNA [NM_000267] | 1.333133 | 1.680298 | -0.34716 | 0.888223 |
| PLXNA4 | Homo sapiens plexin A4 (PLXNA4), transcript variant 1, mRNA [NM_020911] | 1.336377 | 1.683064 | -0.34669 | 0.888223 |
| ABL2 | Homo sapiens v-abl Abelson murine leukemia viral oncogene homolog 2 (ABL2), transcript variant e, mRNA [NM_001136001] | 3.166402 | 3.512115 | -0.34571 | 0.940802 |
| ENST00000378430 | CD72 molecule [Source:HGNC Symbol;Acc:1696] [ENST00000378430] | 5.880641 | 6.225103 | -0.34446 | 0.938174 |
| PRDM1 | Homo sapiens PR domain containing 1, with ZNF domain (PRDM1), transcript variant 1, mRNA [NM_001198] | 1.836704 | 2.18051 | -0.34381 | 0.888223 |
| NCOR1 | Homo sapiens nuclear receptor corepressor 1 (NCOR1), transcript variant 1, mRNA [NM_006311] | 1.828408 | 2.17215 | -0.34374 | 0.922215 |
| BBS4 | Homo sapiens Bardet-Biedl syndrome 4 (BBS4), mRNA [NM_033028] | 1.280775 | 1.6235 | -0.34273 | 0.888223 |
| OCA2 | Homo sapiens oculocutaneous albinism II (OCA2), mRNA [NM_000275] | 1.507465 | 1.849845 | -0.34238 | 0.888251 |
| PCSK1 | Homo sapiens proprotein convertase subtilisin/kexin type 1 (PCSK1), transcript variant 1, mRNA [NM_000439] | 1.737627 | 2.07939 | -0.34176 | 0.902577 |
| NRXN1 | Homo sapiens neurexin 1 (NRXN1), transcript variant alpha1, mRNA [NM_004801] | 1.580293 | 1.921862 | -0.34157 | 0.888223 |
| CDC42 | Homo sapiens cell division cycle 42 (GTP binding protein, 25kDa) (CDC42), transcript variant 3, mRNA [NM_001039802] | 1.394704 | 1.736035 | -0.34133 | 0.888223 |
| POU4F1 | Homo sapiens POU class 4 homeobox 1 (POU4F1), mRNA [NM_006237] | 2.227827 | 2.568704 | -0.34088 | 0.964566 |
| MAPK3 | Homo sapiens mitogen-activated protein kinase 3 (MAPK3), transcript variant 1, mRNA [NM_002746] | 2.219903 | 2.560523 | -0.34062 | 0.888223 |
| EPB41L5 | Homo sapiens erythrocyte membrane protein band 4.1 like 5 (EPB41L5), transcript variant 1, mRNA [NM_020909] | 1.385433 | 1.725919 | -0.34049 | 0.888223 |
| MB | Homo sapiens myoglobin (MB), transcript variant 2, mRNA [NM_203377] | 1.411659 | 1.751796 | -0.34014 | 0.888223 |
| ARIH2 | Homo sapiens ariadne homolog 2 (Drosophila) (ARIH2), mRNA [NM_006321] | 1.282358 | 1.621319 | -0.33896 | 0.888223 |
| CUX1 | Homo sapiens cut-like homeobox 1 (CUX1), transcript variant 1, mRNA [NM_181552] | 1.43309 | 1.770356 | -0.33727 | 0.888223 |
| DPY19L2 | Homo sapiens dpy-19-like 2 (C. elegans) (DPY19L2), mRNA [NM_173812] | 2.612377 | 2.947235 | -0.33486 | 0.888223 |
| EGFR | Homo sapiens epidermal growth factor receptor (EGFR), transcript variant 3, mRNA [NM_201283] | 1.469394 | 1.803099 | -0.3337 | 0.888223 |
| GLG1 | Homo sapiens golgi glycoprotein 1 (GLG1), transcript variant 1, mRNA [NM_012201] | 1.580986 | 1.91468 | -0.33369 | 0.907842 |
| PSAP | Homo sapiens prosaposin (PSAP), transcript variant 2, mRNA [NM_001042465] | 2.45721 | 2.790889 | -0.33368 | 0.907845 |
| PTK2B | Homo sapiens cDNA FLJ46514 fis, clone THYMU3032798, highly similar to Focal adhesion kinase 2 (EC 2.7.1.112). [AK128371] | 3.166782 | 3.500313 | -0.33353 | 0.888223 |
| HDAC10 | Homo sapiens histone deacetylase 10 (HDAC10), transcript variant 1, mRNA [NM_032019] | 1.709551 | 2.04175 | -0.3322 | 0.888223 |
| ENST00000359354 | fibroblast growth factor receptor 2 [Source:HGNC Symbol;Acc:3689] [ENST00000359354] | 3.835434 | 4.167515 | -0.33208 | 0.888223 |
| SEMA4D | Homo sapiens sema domain, immunoglobulin domain (Ig), transmembrane domain (TM) and short cytoplasmic domain, (semaphorin) 4D (SEMA4D), transcript variant 2, mRNA [NM_001142287] | 1.663047 | 1.994446 | -0.3314 | 0.911311 |
| RPS6KA1 | Homo sapiens ribosomal protein S6 kinase, 90kDa, polypeptide 1 (RPS6KA1), transcript variant 1, mRNA [NM_002953] | 2.669298 | 3.000016 | -0.33072 | 0.928543 |
| NPHP3 | Homo sapiens nephronophthisis 3 (adolescent) (NPHP3), mRNA [NM_153240] | 1.458178 | 1.788212 | -0.33003 | 0.888223 |
| NRXN1 | Homo sapiens neurexin 1 (NRXN1), transcript variant alpha1, mRNA [NM_004801] | 2.992176 | 3.321291 | -0.32911 | 0.910877 |
| CSNK2B | Homo sapiens casein kinase 2, beta polypeptide (CSNK2B), mRNA [NM_001320] | 1.766082 | 2.093717 | -0.32763 | 0.934861 |
| APOLD1 | Homo sapiens apolipoprotein L domain containing 1 (APOLD1), transcript variant 1, mRNA [NM_001130415] | 1.786762 | 2.112088 | -0.32533 | 0.930933 |
| MEF2A | Homo sapiens myocyte enhancer factor 2A (MEF2A), transcript variant 5, mRNA [NM_001171894] | 1.331382 | 1.655508 | -0.32413 | 0.888223 |
| IFNB1 | Homo sapiens interferon, beta 1, fibroblast (IFNB1), mRNA [NM_002176] | 1.284667 | 1.608427 | -0.32376 | 0.888223 |
| SEPT2 | Homo sapiens septin 2 (SEPT2), transcript variant 1, mRNA [NM_001008491] | 1.39175 | 1.715203 | -0.32345 | 0.888223 |
| C11orf9 | Homo sapiens chromosome 11 open reading frame 9 (C11orf9), transcript variant 1, mRNA [NM_013279] | 1.37295 | 1.695862 | -0.32291 | 0.888223 |
| CNGA3 | Homo sapiens cyclic nucleotide gated channel alpha 3 (CNGA3), transcript variant 1, mRNA [NM_001298] | 1.851302 | 2.173556 | -0.32225 | 0.914676 |
| STRA6 | Homo sapiens stimulated by retinoic acid gene 6 homolog (mouse) (STRA6), transcript variant 8, mRNA [NM_001199042] | 1.661971 | 1.983001 | -0.32103 | 0.911311 |
| CELA1 | Homo sapiens chymotrypsin-like elastase family, member 1 (CELA1), mRNA [NM_001971] | 1.535562 | 1.855821 | -0.32026 | 0.888223 |
| DCLRE1C | Homo sapiens DNA cross-link repair 1C (DCLRE1C), transcript variant c, mRNA [NM_001033858] | 1.455629 | 1.775192 | -0.31956 | 0.888223 |
| NTNG1 | netrin G1 [Source:HGNC Symbol;Acc:23319] [ENST00000370068] | 1.417174 | 1.736189 | -0.31901 | 0.888223 |
| BCL6 | Homo sapiens B-cell CLL/lymphoma 6 (BCL6), transcript variant 2, mRNA [NM_001130845] | 1.433038 | 1.750929 | -0.31789 | 0.888223 |
| DLG1 | Homo sapiens discs, large homolog 1 (Drosophila) (DLG1), transcript variant 1, mRNA [NM_001098424] | 1.305639 | 1.623164 | -0.31752 | 0.888223 |
| RDH10 | Homo sapiens retinol dehydrogenase 10 (all-trans) (RDH10), mRNA [NM_172037] | 4.36 | 4.6765 | -0.3165 | 0.888223 |
| RELA | Homo sapiens v-rel reticuloendotheliosis viral oncogene homolog A (avian) (RELA), transcript variant 1, mRNA [NM_021975] | 1.572953 | 1.889129 | -0.31618 | 0.888223 |
| CCDC64 | Homo sapiens coiled-coil domain containing 64 (CCDC64), mRNA [NM_207311] | 1.831297 | 2.147334 | -0.31604 | 0.92235 |
| OMP | Homo sapiens olfactory marker protein (OMP), mRNA [NM_006189] | 2.123744 | 2.439051 | -0.31531 | 0.888223 |
| ESR1 | Homo sapiens estrogen receptor 1 (ESR1), transcript variant 1, mRNA [NM_000125] | 3.922099 | 4.237208 | -0.31511 | 0.888223 |
| NAV1 | Homo sapiens neuron navigator 1 (NAV1), transcript variant 1, mRNA [NM_020443] | 1.403947 | 1.718853 | -0.31491 | 0.888223 |
| CAMK1D | Homo sapiens calcium/calmodulin-dependent protein kinase ID (CAMK1D), transcript variant 1, mRNA [NM_020397] | 1.517365 | 1.831916 | -0.31455 | 0.899333 |
| CLIC4 | Homo sapiens chloride intracellular channel 4 (CLIC4), nuclear gene encoding mitochondrial protein, mRNA [NM_013943] | 1.469605 | 1.783298 | -0.31369 | 0.888223 |
| EIF2AK3 | Homo sapiens eukaryotic translation initiation factor 2-alpha kinase 3 (EIF2AK3), mRNA [NM_004836] | 1.302216 | 1.615816 | -0.3136 | 0.888223 |
| DISC1 | Homo sapiens disrupted in schizophrenia 1 (DISC1), transcript variant S, mRNA [NM_001012959] | 1.338116 | 1.650598 | -0.31248 | 0.888223 |
| NCS1 | Homo sapiens neuronal calcium sensor 1 (NCS1), transcript variant 1, mRNA [NM_014286] | 1.418418 | 1.730399 | -0.31198 | 0.888223 |
| IL1RAPL1 | Homo sapiens interleukin 1 receptor accessory protein-like 1 (IL1RAPL1), mRNA [NM_014271] | 2.799594 | 3.110997 | -0.3114 | 0.894828 |
| NKX2-1 | Homo sapiens NK2 homeobox 1 (NKX2-1), transcript variant 2, mRNA [NM_003317] | 1.400619 | 1.711862 | -0.31124 | 0.888223 |
| COL4A4 | Homo sapiens collagen, type IV, alpha 4 (COL4A4), mRNA [NM_000092] | 1.333864 | 1.644489 | -0.31063 | 0.888223 |
| WNT8A | Homo sapiens wingless-type MMTV integration site family, member 8A (WNT8A), mRNA [NM_058244] | 3.68975 | 3.999844 | -0.31009 | 0.910883 |
| CHRNB1 | Homo sapiens cholinergic receptor, nicotinic, beta 1 (muscle) (CHRNB1), mRNA [NM_000747] | 3.287156 | 3.596158 | -0.309 | 0.888223 |
| HMGA2 | Homo sapiens high mobility group AT-hook 2 (HMGA2), transcript variant 2, mRNA [NM_003484] | 3.931348 | 4.24016 | -0.30881 | 0.928933 |
| NPTN | Homo sapiens neuroplastin (NPTN), transcript variant b, mRNA [NM_012428] | 1.444806 | 1.752876 | -0.30807 | 0.888223 |
| INHBA | Homo sapiens inhibin, beta A (INHBA), mRNA [NM_002192] | 4.171056 | 4.476807 | -0.30575 | 0.888223 |
| PAX2 | Homo sapiens paired box 2 (PAX2), transcript variant e, mRNA [NM_003990] | 3.718193 | 4.023511 | -0.30532 | 0.902508 |
| DTNBP1 | Homo sapiens dystrobrevin binding protein 1 (DTNBP1), transcript variant 2, mRNA [NM_183040] | 1.518823 | 1.824132 | -0.30531 | 0.888223 |
| SEMA4F | Homo sapiens sema domain, immunoglobulin domain (Ig), transmembrane domain (TM) and short cytoplasmic domain, (semaphorin) 4F (SEMA4F), mRNA [NM_004263] | 2.559266 | 2.863847 | -0.30458 | 0.914628 |
| COL11A1 | Homo sapiens collagen, type XI, alpha 1 (COL11A1), transcript variant B, mRNA [NM_080629] | 1.450627 | 1.749731 | -0.2991 | 0.888223 |
| FOXI2 | Homo sapiens forkhead box I2 (FOXI2), mRNA [NM_207426] | 16.90538 | 17.20431 | -0.29893 | 0.888223 |
| SIPA1L1 | Homo sapiens signal-induced proliferation-associated 1 like 1 (SIPA1L1), mRNA [NM_015556] | 2.605366 | 2.903892 | -0.29853 | 0.913827 |
| LMO2 | Homo sapiens LIM domain only 2 (rhombotin-like 1) (LMO2), transcript variant 1, mRNA [NM_005574] | 1.38749 | 1.685608 | -0.29812 | 0.888223 |
| HMGCR | Homo sapiens 3-hydroxy-3-methylglutaryl-CoA reductase (HMGCR), transcript variant 1, mRNA [NM_000859] | 3.293248 | 3.591362 | -0.29811 | 0.926601 |
| FGFR2 | Homo sapiens fibroblast growth factor receptor 2 (FGFR2), transcript variant 2, mRNA [NM_022970] | 1.334285 | 1.631667 | -0.29738 | 0.888223 |
| SLITRK6 | Homo sapiens SLIT and NTRK-like family, member 6 (SLITRK6), mRNA [NM_032229] | 1.418493 | 1.715557 | -0.29706 | 0.888223 |
| APBB1 | Homo sapiens amyloid beta (A4) precursor protein-binding, family B, member 1 (Fe65) (APBB1), transcript variant 1, mRNA [NM_001164] | 1.804146 | 2.096858 | -0.29271 | 0.888223 |
| IL6ST | Homo sapiens interleukin 6 signal transducer (gp130, oncostatin M receptor) (IL6ST), transcript variant 3, mRNA [NM_001190981] | 1.514627 | 1.807153 | -0.29253 | 0.888223 |
| THY1 | Homo sapiens Thy-1 cell surface antigen (THY1), mRNA [NM_006288] | 1.527832 | 1.817377 | -0.28954 | 0.888223 |
| XRCC6 | Homo sapiens X-ray repair complementing defective repair in Chinese hamster cells 6 (XRCC6), mRNA [NM_001469] | 3.496118 | 3.784349 | -0.28823 | 0.908507 |
| SIAH1 | Homo sapiens seven in absentia homolog 1 (Drosophila) (SIAH1), transcript variant 1, mRNA [NM_003031] | 1.799383 | 2.087331 | -0.28795 | 0.933216 |
| COL6A3 | Homo sapiens collagen, type VI, alpha 3 (COL6A3), transcript variant 3, mRNA [NM_057165] | 1.480615 | 1.76731 | -0.2867 | 0.888223 |
| SHBG | Homo sapiens sex hormone-binding globulin (SHBG), transcript variant 4, mRNA [NM_001146281] | 1.455976 | 1.741558 | -0.28558 | 0.888223 |
| FGFR3 | Homo sapiens fibroblast growth factor receptor 3 (FGFR3), transcript variant 1, mRNA [NM_000142] | 1.821635 | 2.106921 | -0.28529 | 0.888223 |
| MSH4 | Homo sapiens mutS homolog 4 (E. coli) (MSH4), mRNA [NM_002440] | 1.475505 | 1.760783 | -0.28528 | 0.888223 |
| LILRB4 | Homo sapiens leukocyte immunoglobulin-like receptor, subfamily B (with TM and ITIM domains), member 4 (LILRB4), transcript variant 1, mRNA [NM_006847] | 2.085739 | 2.368498 | -0.28276 | 0.940216 |
| RHOC | Homo sapiens ras homolog gene family, member C (RHOC), transcript variant 1, mRNA [NM_175744] | 1.562718 | 1.845033 | -0.28232 | 0.89091 |
| MYD88 | Homo sapiens myeloid differentiation primary response gene (88) (MYD88), transcript variant 2, mRNA [NM_002468] | 1.32099 | 1.602828 | -0.28184 | 0.888223 |
| GDF6 | Homo sapiens growth differentiation factor 6 (GDF6), mRNA [NM_001001557] | 1.287232 | 1.568781 | -0.28155 | 0.888223 |
| IL12RB1 | Homo sapiens interleukin 12 receptor, beta 1 (IL12RB1), transcript variant 2, mRNA [NM_153701] | 1.635311 | 1.916849 | -0.28154 | 0.888223 |
| COL6A2 | Homo sapiens collagen, type VI, alpha 2 (COL6A2), transcript variant 2C2a', mRNA [NM_058175] | 3.674583 | 3.955556 | -0.28097 | 0.913853 |
| DUSP10 | Homo sapiens dual specificity phosphatase 10 (DUSP10), transcript variant 1, mRNA [NM_007207] | 1.490361 | 1.770927 | -0.28057 | 0.888223 |
| VEGFA | Homo sapiens vascular endothelial growth factor A (VEGFA), transcript variant 1, mRNA [NM_001025366] | 1.400642 | 1.681199 | -0.28056 | 0.888223 |
| ENST00000339879 | serine/threonine kinase 3 [Source:HGNC Symbol;Acc:11406] [ENST00000424861] | 1.759972 | 2.039616 | -0.27964 | 0.888223 |
| FZR1 | Homo sapiens fizzy/cell division cycle 20 related 1 (Drosophila) (FZR1), transcript variant 2, mRNA [NM_016263] | 1.431745 | 1.711161 | -0.27942 | 0.888223 |
| ARHGEF2 | Homo sapiens Rho/Rac guanine nucleotide exchange factor (GEF) 2 (ARHGEF2), transcript variant 1, mRNA [NM_001162383] | 1.748177 | 2.026108 | -0.27793 | 0.908164 |
| BVES | Homo sapiens blood vessel epicardial substance (BVES), transcript variant B, mRNA [NM_147147] | 1.35204 | 1.629464 | -0.27742 | 0.888223 |
| ZFYVE27 | Homo sapiens zinc finger, FYVE domain containing 27 (ZFYVE27), transcript variant 1, mRNA [NM_001002261] | 1.310321 | 1.586732 | -0.27641 | 0.888223 |
| ZGLP1 | Homo sapiens zinc finger, GATA-like protein 1 (ZGLP1), mRNA [NM_001103167] | 3.348705 | 3.624173 | -0.27547 | 0.951631 |
| TMEM90A | Homo sapiens synapse differentiation inducing 1-like (SYNDIG1L), mRNA [NM_001105579] | 1.667721 | 1.942963 | -0.27524 | 0.905587 |
| ITGB1BP3 | Homo sapiens integrin beta 1 binding protein 3 (ITGB1BP3), mRNA [NM_170678] | 1.433212 | 1.707829 | -0.27462 | 0.888223 |
| BCL11B | Homo sapiens B-cell CLL/lymphoma 11B (zinc finger protein) (BCL11B), transcript variant 1, mRNA [NM_138576] | 2.041419 | 2.315937 | -0.27452 | 0.917204 |
| IL10 | Homo sapiens interleukin 10 (IL10), mRNA [NM_000572] | 1.731492 | 2.005825 | -0.27433 | 0.888223 |
| IL20 | Homo sapiens interleukin 20 (IL20), mRNA [NM_018724] | 4.271298 | 4.544458 | -0.27316 | 0.946291 |
| SGPL1 | Homo sapiens sphingosine-1-phosphate lyase 1 (SGPL1), mRNA [NM_003901] | 1.392999 | 1.666044 | -0.27304 | 0.888223 |
| HAND2 | Homo sapiens heart and neural crest derivatives expressed 2 (HAND2), mRNA [NM_021973] | 1.861907 | 2.134763 | -0.27286 | 0.934467 |
| RAB11A | Homo sapiens RAB11A, member RAS oncogene family (RAB11A), transcript variant 1, mRNA [NM_004663] | 1.342489 | 1.615077 | -0.27259 | 0.888223 |
| PLXNB2 | Homo sapiens plexin B2 (PLXNB2), mRNA [NM_012401] | 1.658875 | 1.92961 | -0.27073 | 0.891623 |
| SLITRK1 | Homo sapiens SLIT and NTRK-like family, member 1 (SLITRK1), mRNA [NM_052910] | 3.107128 | 3.377771 | -0.27064 | 0.894645 |
| LHX6 | Homo sapiens LIM homeobox 6 (LHX6), transcript variant 1, mRNA [NM_014368] | 1.448188 | 1.717788 | -0.2696 | 0.888223 |
| GDF3 | Homo sapiens growth differentiation factor 3 (GDF3), mRNA [NM_020634] | 2.017155 | 2.28667 | -0.26952 | 0.950677 |
| KAL1 | Homo sapiens Kallmann syndrome 1 sequence (KAL1), mRNA [NM_000216] | 1.795126 | 2.063712 | -0.26859 | 0.920561 |
| CHAT | Homo sapiens choline O-acetyltransferase (CHAT), transcript variant M, mRNA [NM_020549] | 1.385395 | 1.653869 | -0.26847 | 0.888223 |
| DMRT2 | Homo sapiens doublesex and mab-3 related transcription factor 2 (DMRT2), transcript variant 1, mRNA [NM_006557] | 1.491977 | 1.760088 | -0.26811 | 0.890447 |
| CD2 | Homo sapiens cDNA, FLJ17718. [AK310676] | 1.34378 | 1.611291 | -0.26751 | 0.888223 |
| EPAS1 | Homo sapiens endothelial PAS domain protein 1 (EPAS1), mRNA [NM_001430] | 1.321919 | 1.588976 | -0.26706 | 0.888223 |
| CSNK2B | Homo sapiens casein kinase 2, beta polypeptide (CSNK2B), mRNA [NM_001320] | 1.743235 | 2.010128 | -0.26689 | 0.92693 |
| FRMD7 | Homo sapiens FERM domain containing 7 (FRMD7), mRNA [NM_194277] | 3.038679 | 3.303303 | -0.26462 | 0.943542 |
| DVL1 | Homo sapiens dishevelled, dsh homolog 1 (Drosophila) (DVL1), mRNA [NM_004421] | 1.48811 | 1.751682 | -0.26357 | 0.888223 |
| FANCC | Homo sapiens Fanconi anemia, complementation group C (FANCC), transcript variant 3, mRNA [NM_001243744] | 1.306569 | 1.570073 | -0.2635 | 0.888223 |
| SRC | Homo sapiens v-src sarcoma (Schmidt-Ruppin A-2) viral oncogene homolog (avian) (SRC), transcript variant 1, mRNA [NM_005417] | 2.09557 | 2.357408 | -0.26184 | 0.888223 |
| NTRK3 | Homo sapiens neurotrophic tyrosine kinase, receptor, type 3 (NTRK3), transcript variant 1, mRNA [NM_001012338] | 1.341473 | 1.601542 | -0.26007 | 0.888223 |
| SPTBN2 | Homo sapiens spectrin, beta, non-erythrocytic 2 (SPTBN2), mRNA [NM_006946] | 2.141797 | 2.401062 | -0.25927 | 0.908164 |
| MYL2 | Homo sapiens myosin, light chain 2, regulatory, cardiac, slow (MYL2), mRNA [NM_000432] | 1.467833 | 1.726675 | -0.25884 | 0.909953 |
| TLX2 | Homo sapiens T-cell leukemia homeobox 2 (TLX2), mRNA [NM_016170] | 1.609269 | 1.867541 | -0.25827 | 0.888223 |
| CLIC4 | Homo sapiens chloride intracellular channel 4 (CLIC4), nuclear gene encoding mitochondrial protein, mRNA [NM_013943] | 1.625433 | 1.883616 | -0.25818 | 0.904078 |
| CCND2 | Homo sapiens cyclin D2 (CCND2), mRNA [NM_001759] | 1.524771 | 1.781771 | -0.257 | 0.888223 |
| SP7 | Homo sapiens Sp7 transcription factor (SP7), transcript variant 1, mRNA [NM_001173467] | 4.568955 | 4.825589 | -0.25663 | 0.954723 |
| QKI | Homo sapiens QKI, KH domain containing, RNA binding (QKI), transcript variant 1, mRNA [NM_006775] | 1.790316 | 2.046503 | -0.25619 | 0.950469 |
| BBS9 | Homo sapiens Bardet-Biedl syndrome 9 (BBS9), transcript variant 1, mRNA [NM_014451] | 1.544179 | 1.800247 | -0.25607 | 0.891708 |
| INHBB | Homo sapiens inhibin, beta B (INHBB), mRNA [NM_002193] | 1.331869 | 1.587842 | -0.25597 | 0.888223 |
| AMELX | Homo sapiens amelogenin, X-linked (AMELX), transcript variant 2, mRNA [NM_182681] | 1.345209 | 1.601036 | -0.25583 | 0.888223 |
| RNF17 | Homo sapiens ring finger protein 17 (RNF17), transcript variant 1, mRNA [NM_031277] | 1.439487 | 1.694733 | -0.25525 | 0.888223 |
| SH3PXD2B | Homo sapiens SH3 and PX domains 2B (SH3PXD2B), mRNA [NM_001017995] | 1.381887 | 1.636476 | -0.25459 | 0.888223 |
| TFE3 | Homo sapiens transcription factor binding to IGHM enhancer 3 (TFE3), mRNA [NM_006521] | 7.630269 | 7.882565 | -0.2523 | 0.888223 |
| LYL1 | Homo sapiens lymphoblastic leukemia derived sequence 1 (LYL1), mRNA [NM_005583] | 1.625338 | 1.877631 | -0.25229 | 0.90051 |
| RAG1 | Homo sapiens recombination activating gene 1 (RAG1), mRNA [NM_000448] | 2.015085 | 2.266962 | -0.25188 | 0.946513 |
| PDLIM7 | Homo sapiens PDZ and LIM domain 7 (enigma) (PDLIM7), transcript variant 1, mRNA [NM_005451] | 1.334524 | 1.58626 | -0.25174 | 0.888223 |
| CCDC85B | Homo sapiens coiled-coil domain containing 85B (CCDC85B), mRNA [NM_006848] | 1.780525 | 2.032207 | -0.25168 | 0.953954 |
| SPTBN5 | Homo sapiens spectrin, beta, non-erythrocytic 5 (SPTBN5), mRNA [NM_016642] | 2.201651 | 2.452937 | -0.25129 | 0.930259 |
| MEN1 | Homo sapiens multiple endocrine neoplasia I (MEN1), transcript variant e1E, mRNA [NM_130803] | 1.611249 | 1.861723 | -0.25047 | 0.888223 |
| PRICKLE1 | Homo sapiens prickle homolog 1 (Drosophila) (PRICKLE1), transcript variant 1, mRNA [NM_153026] | 1.678615 | 1.927774 | -0.24916 | 0.888223 |
| CSNK2A1 | Homo sapiens casein kinase 2, alpha 1 polypeptide (CSNK2A1), transcript variant 1, mRNA [NM_177559] | 3.99938 | 4.24806 | -0.24868 | 0.894216 |
| MKKS | Homo sapiens McKusick-Kaufman syndrome (MKKS), transcript variant 2, mRNA [NM_170784] | 1.301379 | 1.550033 | -0.24865 | 0.888223 |
| TDRD7 | Homo sapiens tudor domain containing 7 (TDRD7), mRNA [NM_014290] | 3.62422 | 3.872211 | -0.24799 | 0.888223 |
| SEMA3A | Homo sapiens sema domain, immunoglobulin domain (Ig), short basic domain, secreted, (semaphorin) 3A (SEMA3A), mRNA [NM_006080] | 1.289826 | 1.537518 | -0.24769 | 0.888223 |
| FOXI3 | Homo sapiens forkhead box I3 (FOXI3), mRNA [NM_001135649] | 2.620219 | 2.866983 | -0.24676 | 0.92693 |
| SGK269 | Homo sapiens NKF3 kinase family member (PEAK1), mRNA [NM_024776] | 1.383762 | 1.630275 | -0.24651 | 0.888223 |
| TWIST1 | Homo sapiens twist homolog 1 (Drosophila) (TWIST1), mRNA [NM_000474] | 1.395935 | 1.641653 | -0.24572 | 0.888223 |
| NDEL1 | Homo sapiens nudE nuclear distribution gene E homolog (A. nidulans)-like 1 (NDEL1), transcript variant 2, mRNA [NM_030808] | 3.361225 | 3.606516 | -0.24529 | 0.932415 |
| TGFBI | Homo sapiens transforming growth factor, beta-induced, 68kDa (TGFBI), mRNA [NM_000358] | 1.339366 | 1.583106 | -0.24374 | 0.888223 |
| PAK3 | Homo sapiens p21 protein (Cdc42/Rac)-activated kinase 3 (PAK3), transcript variant 2, mRNA [NM_002578] | 1.750985 | 1.992932 | -0.24195 | 0.914216 |
| HMGB2 | Homo sapiens high mobility group box 2 (HMGB2), transcript variant 1, mRNA [NM_002129] | 1.34317 | 1.583807 | -0.24064 | 0.888223 |
| LTBP3 | Homo sapiens latent transforming growth factor beta binding protein 3 (LTBP3), transcript variant 2, mRNA [NM_021070] | 1.346893 | 1.587397 | -0.2405 | 0.888223 |
| GDF2 | Homo sapiens growth differentiation factor 2 (GDF2), mRNA [NM_016204] | 1.888273 | 2.128429 | -0.24016 | 0.934671 |
| NOTCH3 | Homo sapiens notch 3 (NOTCH3), mRNA [NM_000435] | 1.468681 | 1.708516 | -0.23983 | 0.888223 |
| CRHR1 | Homo sapiens corticotropin releasing hormone receptor 1 (CRHR1), transcript variant 1, mRNA [NM_001145146] | 7.28131 | 7.519446 | -0.23814 | 0.960822 |
| CRYAA | Homo sapiens crystallin, alpha A (CRYAA), mRNA [NM_000394] | 1.443881 | 1.681212 | -0.23733 | 0.888223 |
| UCN | Homo sapiens urocortin (UCN), mRNA [NM_003353] | 1.527306 | 1.764269 | -0.23696 | 0.910559 |
| NF2 | Homo sapiens neurofibromin 2 (merlin) (NF2), transcript variant 13, mRNA [NM_181831] | 1.810685 | 2.046476 | -0.23579 | 0.888223 |
| LFNG | Homo sapiens LFNG O-fucosylpeptide 3-beta-N-acetylglucosaminyltransferase (LFNG), transcript variant 1, mRNA [NM_001040167] | 4.6256 | 4.860952 | -0.23535 | 0.920798 |
| MYADML | Homo sapiens myeloid-associated differentiation marker-like (MYADML), non-coding RNA [NR_003143] | 1.355125 | 1.590362 | -0.23524 | 0.888223 |
| FGFR1 | fibroblast growth factor receptor 1 [Source:HGNC Symbol;Acc:3688] [ENST00000496296] | 1.359521 | 1.594066 | -0.23455 | 0.888223 |
| SPTAN1 | Homo sapiens spectrin, alpha, non-erythrocytic 1 (alpha-fodrin) (SPTAN1), transcript variant 1, mRNA [NM_001130438] | 1.499263 | 1.733 | -0.23374 | 0.888223 |
| SIK1 | Homo sapiens salt-inducible kinase 1 (SIK1), mRNA [NM_173354] | 1.564985 | 1.797456 | -0.23247 | 0.888223 |
| LHX2 | Homo sapiens LIM homeobox 2 (LHX2), mRNA [NM_004789] | 1.3998 | 1.631253 | -0.23145 | 0.888223 |
| DNASE2 | Homo sapiens deoxyribonuclease II, lysosomal (DNASE2), mRNA [NM_001375] | 1.409503 | 1.640104 | -0.2306 | 0.888223 |
| PRMT2 | Homo sapiens protein arginine methyltransferase 2 (PRMT2), transcript variant 1, mRNA [NM_206962] | 1.426921 | 1.655944 | -0.22902 | 0.888223 |
| EN2 | Homo sapiens engrailed homeobox 2 (EN2), mRNA [NM_001427] | 7.931569 | 8.159738 | -0.22817 | 0.926925 |
| NUMB | BROAD Institute lincRNA (XLOC_011068), lincRNA [TCONS_00022790] | 1.666891 | 1.894423 | -0.22753 | 0.888223 |
| FHL2 | Homo sapiens four and a half LIM domains 2 (FHL2), transcript variant 5, mRNA [NM_001039492] | 1.397405 | 1.623641 | -0.22624 | 0.888223 |
| SFRP2 | Homo sapiens secreted frizzled-related protein 2 (SFRP2), mRNA [NM_003013] | 2.551505 | 2.777285 | -0.22578 | 0.941588 |
| WNT7B | Homo sapiens wingless-type MMTV integration site family, member 7B (WNT7B), mRNA [NM_058238] | 1.409994 | 1.635631 | -0.22564 | 0.888223 |
| ISL2 | Homo sapiens ISL LIM homeobox 2 (ISL2), mRNA [NM_145805] | 1.28211 | 1.507468 | -0.22536 | 0.888223 |
| LIF | Homo sapiens leukemia inhibitory factor (cholinergic differentiation factor) (LIF), mRNA [NM_002309] | 1.422027 | 1.647302 | -0.22527 | 0.888223 |
| EDN1 | Homo sapiens endothelin 1 (EDN1), transcript variant 1, mRNA [NM_001955] | 1.647651 | 1.872774 | -0.22512 | 0.888223 |
| UPK2 | Homo sapiens uroplakin 2 (UPK2), mRNA [NM_006760] | 1.319682 | 1.544604 | -0.22492 | 0.888223 |
| NCOR1 | Homo sapiens nuclear receptor corepressor 1 (NCOR1), transcript variant 2, mRNA [NM_001190438] | 1.616733 | 1.841096 | -0.22436 | 0.922171 |
| PSEN1 | presenilin 1 [Source:HGNC Symbol;Acc:9508] [ENST00000394157] | 1.292309 | 1.51619 | -0.22388 | 0.888223 |
| CLIC4 | Homo sapiens chloride intracellular channel 4 (CLIC4), nuclear gene encoding mitochondrial protein, mRNA [NM_013943] | 1.344551 | 1.568316 | -0.22376 | 0.888223 |
| RBM38 | Homo sapiens RNA binding motif protein 38 (RBM38), transcript variant 1, mRNA [NM_017495] | 1.287097 | 1.510118 | -0.22302 | 0.888223 |
| LPIN1 | Homo sapiens lipin 1 (LPIN1), mRNA [NM_145693] | 1.343239 | 1.566025 | -0.22279 | 0.888223 |
| WNT7A | Homo sapiens wingless-type MMTV integration site family, member 7A (WNT7A), mRNA [NM_004625] | 1.907815 | 2.129741 | -0.22193 | 0.964566 |
| RPS6KA2 | Homo sapiens ribosomal protein S6 kinase, 90kDa, polypeptide 2 (RPS6KA2), transcript variant 1, mRNA [NM_021135] | 1.42819 | 1.650018 | -0.22183 | 0.888223 |
| NF1 | Homo sapiens neurofibromin 1 (NF1), transcript variant 2, mRNA [NM_000267] | 1.328114 | 1.547688 | -0.21957 | 0.888223 |
| SLIT3 | Homo sapiens slit homolog 3 (Drosophila) (SLIT3), mRNA [NM_003062] | 2.131439 | 2.35068 | -0.21924 | 0.917204 |
| ULK2 | Homo sapiens unc-51-like kinase 2 (C. elegans) (ULK2), transcript variant 1, mRNA [NM_014683] | 1.494778 | 1.712715 | -0.21794 | 0.888223 |
| APOB | Homo sapiens apolipoprotein B (including Ag(x) antigen) (APOB), mRNA [NM_000384] | 1.620766 | 1.838414 | -0.21765 | 0.888223 |
| THEMIS | Homo sapiens thymocyte selection associated (THEMIS), transcript variant 1, mRNA [NM_001164685] | 1.437553 | 1.653251 | -0.2157 | 0.888223 |
| NTNG1 | Homo sapiens netrin G1 (NTNG1), transcript variant 1, mRNA [NM_001113226] | 1.548761 | 1.764131 | -0.21537 | 0.888223 |
| ETV1 | Homo sapiens ets variant 1 (ETV1), transcript variant 1, mRNA [NM_004956] | 1.492355 | 1.706873 | -0.21452 | 0.921462 |
| TUBB | Homo sapiens tubulin, beta class I (TUBB), mRNA [NM_178014] | 1.316995 | 1.531483 | -0.21449 | 0.888223 |
| ATG7 | Homo sapiens ATG7 autophagy related 7 homolog (S. cerevisiae) (ATG7), transcript variant 1, mRNA [NM_006395] | 1.410877 | 1.624528 | -0.21365 | 0.888223 |
| NUDT7 | Homo sapiens nudix (nucleoside diphosphate linked moiety X)-type motif 7 (NUDT7), transcript variant 3, mRNA [NM_001243657] | 1.585632 | 1.798727 | -0.2131 | 0.890447 |
| FAS | Homo sapiens Fas (TNF receptor superfamily, member 6) (FAS), transcript variant 1, mRNA [NM_000043] | 1.681278 | 1.893673 | -0.21239 | 0.888223 |
| NF1 | Homo sapiens neurofibromin 1 (NF1), transcript variant 2, mRNA [NM_000267] | 1.410919 | 1.623145 | -0.21223 | 0.888223 |
| FGFR2 | Homo sapiens fibroblast growth factor receptor 2 (FGFR2), transcript variant 2, mRNA [NM_022970] | 1.604242 | 1.81562 | -0.21138 | 0.888223 |
| TCEA1 | Homo sapiens transcription elongation factor A (SII), 1 (TCEA1), transcript variant 1, mRNA [NM_006756] | 1.301293 | 1.512617 | -0.21132 | 0.888223 |
| MYLK3 | Homo sapiens myosin light chain kinase 3 (MYLK3), mRNA [NM_182493] | 4.202567 | 4.412852 | -0.21029 | 0.91362 |
| CDC42 | Homo sapiens cell division cycle 42 (GTP binding protein, 25kDa) (CDC42), transcript variant 2, mRNA [NM_044472] | 1.546875 | 1.757039 | -0.21016 | 0.888223 |
| SMAD4 | Homo sapiens SMAD family member 4 (SMAD4), mRNA [NM_005359] | 1.570424 | 1.779894 | -0.20947 | 0.888956 |
| EPHA3 | Homo sapiens EPH receptor A3 (EPHA3), transcript variant 1, mRNA [NM_005233] | 1.378817 | 1.587961 | -0.20914 | 0.888223 |
| HOXB13 | Homo sapiens homeobox B13 (HOXB13), mRNA [NM_006361] | 1.695502 | 1.901151 | -0.20565 | 0.92693 |
| RTN4RL1 | Homo sapiens reticulon 4 receptor-like 1 (RTN4RL1), mRNA [NM_178568] | 2.101376 | 2.30585 | -0.20447 | 0.935319 |
| ANK2 | Homo sapiens ankyrin 2, neuronal (ANK2), transcript variant 1, mRNA [NM_001148] | 1.504631 | 1.704906 | -0.20027 | 0.888223 |
| FEZ1 | Homo sapiens fasciculation and elongation protein zeta 1 (zygin I) (FEZ1), transcript variant 1, mRNA [NM_005103] | 1.572009 | 1.770152 | -0.19814 | 0.888223 |
| PLXNB1 | Homo sapiens plexin B1 (PLXNB1), transcript variant 1, mRNA [NM_002673] | 1.71458 | 1.912463 | -0.19788 | 0.924609 |
| ZMIZ1 | Homo sapiens zinc finger, MIZ-type containing 1 (ZMIZ1), mRNA [NM_020338] | 1.411686 | 1.609268 | -0.19758 | 0.888223 |
| AP3B1 | Homo sapiens adaptor-related protein complex 3, beta 1 subunit (AP3B1), mRNA [NM_003664] | 1.354804 | 1.551615 | -0.19681 | 0.888223 |
| SHROOM3 | Homo sapiens shroom family member 3 (SHROOM3), mRNA [NM_020859] | 1.502947 | 1.698717 | -0.19577 | 0.893423 |
| FNDC3A | Homo sapiens fibronectin type III domain containing 3A (FNDC3A), transcript variant 1, mRNA [NM_001079673] | 1.652918 | 1.848347 | -0.19543 | 0.942008 |
| FST | Homo sapiens follistatin (FST), transcript variant FST344, mRNA [NM_013409] | 1.411294 | 1.6067 | -0.19541 | 0.888223 |
| SPRY2 | Homo sapiens sprouty homolog 2 (Drosophila) (SPRY2), mRNA [NM_005842] | 1.464493 | 1.658055 | -0.19356 | 0.888223 |
| HDAC11 | Homo sapiens histone deacetylase 11 (HDAC11), transcript variant 1, mRNA [NM_024827] | 1.57662 | 1.770172 | -0.19355 | 0.930259 |
| LCE1A | Homo sapiens late cornified envelope 1A (LCE1A), mRNA [NM_178348] | 4.253262 | 4.443841 | -0.19058 | 0.936601 |
| EID2 | Homo sapiens EP300 interacting inhibitor of differentiation 2 (EID2), mRNA [NM_153232] | 2.030122 | 2.220611 | -0.19049 | 0.955824 |
| UQCRQ | Homo sapiens ubiquinol-cytochrome c reductase, complex III subunit VII, 9.5kDa (UQCRQ), nuclear gene encoding mitochondrial protein, mRNA [NM_014402] | 1.609974 | 1.799605 | -0.18963 | 0.9238 |
| RHOA | Homo sapiens ras homolog gene family, member A (RHOA), mRNA [NM_001664] | 1.488819 | 1.677591 | -0.18877 | 0.888223 |
| ESR2 | Homo sapiens estrogen receptor 2 (ER beta) (ESR2), transcript variant a, mRNA [NM_001437] | 1.379425 | 1.567611 | -0.18819 | 0.888223 |
| COL6A1 | Homo sapiens collagen, type VI, alpha 1 (COL6A1), mRNA [NM_001848] | 1.742701 | 1.929061 | -0.18636 | 0.888223 |
| ABCA1 | Homo sapiens ATP-binding cassette, sub-family A (ABC1), member 1 (ABCA1), mRNA [NM_005502] | 1.322736 | 1.508873 | -0.18614 | 0.888223 |
| NRIP1 | Homo sapiens nuclear receptor interacting protein 1 (NRIP1), mRNA [NM_003489] | 1.492021 | 1.677393 | -0.18537 | 0.888223 |
| MDGA1 | MAM domain containing glycosylphosphatidylinositol anchor 1 [Source:HGNC Symbol;Acc:19267] [ENST00000373401] | 2.370581 | 2.55588 | -0.1853 | 0.974502 |
| LIN28 | Homo sapiens lin-28 homolog A (C. elegans) (LIN28A), mRNA [NM_024674] | 2.981958 | 3.166517 | -0.18456 | 0.971463 |
| PROX1 | Homo sapiens prospero homeobox 1 (PROX1), mRNA [NM_002763] | 1.342193 | 1.525748 | -0.18355 | 0.888223 |
| KIAA1161 | Homo sapiens KIAA1161 (KIAA1161), mRNA [NM_020702] | 2.014074 | 2.197292 | -0.18322 | 0.954202 |
| CACNA1C | Homo sapiens calcium channel, voltage-dependent, L type, alpha 1C subunit (CACNA1C), transcript variant 1, mRNA [NM_199460] | 1.323953 | 1.5065 | -0.18255 | 0.888223 |
| WNT3A | Homo sapiens wingless-type MMTV integration site family, member 3A (WNT3A), mRNA [NM_033131] | 1.978704 | 2.160577 | -0.18187 | 0.936547 |
| STIP1 | Homo sapiens stress-induced-phosphoprotein 1 (STIP1), mRNA [NM_006819] | 1.350863 | 1.532729 | -0.18187 | 0.888223 |
| FGF6 | Homo sapiens fibroblast growth factor 6 (FGF6), mRNA [NM_020996] | 1.452316 | 1.634144 | -0.18183 | 0.888223 |
| CYP1A1 | Homo sapiens cytochrome P450, family 1, subfamily A, polypeptide 1 (CYP1A1), mRNA [NM_000499] | 2.310563 | 2.491656 | -0.18109 | 0.935544 |
| EPHA5 | Homo sapiens EPH receptor A5 (EPHA5), transcript variant 1, mRNA [NM_004439] | 1.492978 | 1.673875 | -0.1809 | 0.888223 |
| EID2 | Homo sapiens EP300 interacting inhibitor of differentiation 2 (EID2), mRNA [NM_153232] | 1.810097 | 1.989783 | -0.17969 | 0.935544 |
| RUNX2 | Homo sapiens runt-related transcription factor 2 (RUNX2), transcript variant 2, mRNA [NM_001015051] | 1.453198 | 1.632577 | -0.17938 | 0.888223 |
| SPEG | Q9EQJ5_MOUSE (Q9EQJ5) Striated muscle-specific serine/threonine protein kinase, partial (7%) [THC2520003] | 2.507382 | 2.686302 | -0.17892 | 0.950049 |
| NKX2-3 | Homo sapiens NK2 homeobox 3 (NKX2-3), mRNA [NM_145285] | 3.98989 | 4.16876 | -0.17887 | 0.935408 |
| SYNE1 | Homo sapiens spectrin repeat containing, nuclear envelope 1 (SYNE1), transcript variant 2, mRNA [NM_033071] | 2.342214 | 2.52055 | -0.17834 | 0.974092 |
| RGMA | Homo sapiens RGM domain family, member A (RGMA), transcript variant 4, mRNA [NM_020211] | 1.479048 | 1.657157 | -0.17811 | 0.910559 |
| RPS6KA2 | Homo sapiens ribosomal protein S6 kinase, 90kDa, polypeptide 2 (RPS6KA2), transcript variant 1, mRNA [NM_021135] | 1.305895 | 1.483958 | -0.17806 | 0.888223 |
| LY6D | Homo sapiens lymphocyte antigen 6 complex, locus D (LY6D), mRNA [NM_003695] | 1.968549 | 2.146218 | -0.17767 | 0.964562 |
| SYK | Homo sapiens spleen tyrosine kinase (SYK), transcript variant 1, mRNA [NM_003177] | 1.382237 | 1.559592 | -0.17735 | 0.888223 |
| DHCR24 | Homo sapiens 24-dehydrocholesterol reductase (DHCR24), mRNA [NM_014762] | 1.307009 | 1.483904 | -0.17689 | 0.888223 |
| PTPRA | Homo sapiens protein tyrosine phosphatase, receptor type, A (PTPRA), transcript variant 1, mRNA [NM_002836] | 1.50438 | 1.680106 | -0.17573 | 0.888223 |
| FKBP4 | Homo sapiens FK506 binding protein 4, 59kDa (FKBP4), mRNA [NM_002014] | 1.44169 | 1.617004 | -0.17531 | 0.888223 |
| BTK | Homo sapiens Bruton agammaglobulinemia tyrosine kinase (BTK), mRNA [NM_000061] | 6.799813 | 6.97471 | -0.1749 | 0.888223 |
| BCL11A | Homo sapiens B-cell CLL/lymphoma 11A (zinc finger protein) (BCL11A), transcript variant 2, mRNA [NM_018014] | 1.656061 | 1.830791 | -0.17473 | 0.901693 |
| SPP1 | Homo sapiens secreted phosphoprotein 1 (SPP1), transcript variant 1, mRNA [NM_001040058] | 1.509332 | 1.684044 | -0.17471 | 0.888223 |
| PLXNA4 | Homo sapiens plexin A4 (PLXNA4), transcript variant 1, mRNA [NM_020911] | 1.515715 | 1.689734 | -0.17402 | 0.912409 |
| ENAH | Homo sapiens enabled homolog (Drosophila) (ENAH), transcript variant 1, mRNA [NM_001008493] | 1.486995 | 1.66081 | -0.17382 | 0.888223 |
| CD28 | Homo sapiens CD28 molecule (CD28), transcript variant 1, mRNA [NM_006139] | 1.30827 | 1.481902 | -0.17363 | 0.888223 |
| WNT10B | Homo sapiens wingless-type MMTV integration site family, member 10B (WNT10B), mRNA [NM_003394] | 1.584724 | 1.755008 | -0.17028 | 0.934349 |
| UNC5C | Homo sapiens unc-5 homolog C (C. elegans) (UNC5C), mRNA [NM_003728] | 3.506957 | 3.675928 | -0.16897 | 0.972274 |
| MAMLD1 | Homo sapiens mastermind-like domain containing 1 (MAMLD1), transcript variant 2, mRNA [NM_005491] | 3.662602 | 3.831185 | -0.16858 | 0.948997 |
| PTPRC | Homo sapiens protein tyrosine phosphatase, receptor type, C (PTPRC), transcript variant 1, mRNA [NM_002838] | 1.524212 | 1.692667 | -0.16846 | 0.925616 |
| HELT | Homo sapiens helt bHLH transcription factor (HELT), mRNA [NM_001029887] | 3.094162 | 3.262534 | -0.16837 | 0.967619 |
| NFKB2 | Homo sapiens nuclear factor of kappa light polypeptide gene enhancer in B-cells 2 (p49/p100) (NFKB2), transcript variant 3, mRNA [NM_001077493] | 3.08393 | 3.250421 | -0.16649 | 0.970182 |
| TYRP1 | Homo sapiens tyrosinase-related protein 1 (TYRP1), mRNA [NM_000550] | 1.388064 | 1.552956 | -0.16489 | 0.888223 |
| SMAD7 | Homo sapiens SMAD family member 7 (SMAD7), transcript variant 1, mRNA [NM_005904] | 2.578324 | 2.743081 | -0.16476 | 0.956255 |
| FOXL2 | Homo sapiens forkhead box L2 (FOXL2), mRNA [NM_023067] | 1.689207 | 1.853519 | -0.16431 | 0.889644 |
| NOX4 | Homo sapiens NADPH oxidase 4 (NOX4), transcript variant 3, mRNA [NM_001143837] | 1.39646 | 1.559263 | -0.1628 | 0.888223 |
| FAS | Homo sapiens Fas (TNF receptor superfamily, member 6) (FAS), transcript variant 1, mRNA [NM_000043] | 1.325273 | 1.487445 | -0.16217 | 0.888223 |
| C10orf58 | Homo sapiens chromosome 10 open reading frame 58 (C10orf58), transcript variant 1, mRNA [NM_032333] | 1.447859 | 1.609979 | -0.16212 | 0.888223 |
| SPEG | Homo sapiens SPEG complex locus (SPEG), transcript variant 4, mRNA [NM_001173476] | 1.460809 | 1.622016 | -0.16121 | 0.888223 |
| S100B | S100 calcium binding protein B [Source:HGNC Symbol;Acc:10500] [ENST00000397648] | 1.707589 | 1.868383 | -0.16079 | 0.936226 |
| RAPGEF4 | Homo sapiens Rap guanine nucleotide exchange factor (GEF) 4 (RAPGEF4), transcript variant 1, mRNA [NM_007023] | 1.399213 | 1.559029 | -0.15982 | 0.888223 |
| YBX2 | Homo sapiens Y box binding protein 2 (YBX2), mRNA [NM_015982] | 2.255829 | 2.414948 | -0.15912 | 0.965597 |
| SOD1 | Homo sapiens superoxide dismutase 1, soluble (SOD1), mRNA [NM_000454] | 1.422943 | 1.581714 | -0.15877 | 0.888223 |
| PDE4D | Homo sapiens phosphodiesterase 4D, cAMP-specific (PDE4D), transcript variant 3, mRNA [NM_001165899] | 1.308934 | 1.467275 | -0.15834 | 0.888223 |
| KRT14 | Homo sapiens keratin 14 (KRT14), mRNA [NM_000526] | 2.41757 | 2.575814 | -0.15824 | 0.974633 |
| NOS1 | Homo sapiens nitric oxide synthase 1 (neuronal) (NOS1), transcript variant 2, mRNA [NM_001204218] | 1.444751 | 1.602469 | -0.15772 | 0.894341 |
| HDAC5 | Homo sapiens histone deacetylase 5 (HDAC5), transcript variant 3, mRNA [NM_001015053] | 2.100792 | 2.257901 | -0.15711 | 0.972274 |
| LAMA4 | Homo sapiens laminin, alpha 4 (LAMA4), transcript variant 1, mRNA [NM_001105206] | 3.718494 | 3.875247 | -0.15675 | 0.970097 |
| LRTOMT | Homo sapiens leucine rich transmembrane and 0-methyltransferase domain containing (LRTOMT), transcript variant 4, mRNA [NM_001145308] | 3.303503 | 3.460146 | -0.15664 | 0.949585 |
| WNT7B | Homo sapiens wingless-type MMTV integration site family, member 7B (WNT7B), mRNA [NM_058238] | 3.791404 | 3.948011 | -0.15661 | 0.961564 |
| NANOS2 | Homo sapiens nanos homolog 2 (Drosophila) (NANOS2), mRNA [NM_001029861] | 1.39065 | 1.547116 | -0.15647 | 0.888223 |
| ENST00000373348 | adipogenin [Source:HGNC Symbol;Acc:28606] [ENST00000373348] | 1.388432 | 1.544345 | -0.15591 | 0.894891 |
| BCL2L1 | Homo sapiens BCL2-like 1 (BCL2L1), nuclear gene encoding mitochondrial protein, transcript variant 1, mRNA [NM_138578] | 1.693911 | 1.849555 | -0.15564 | 0.888223 |
| PRDX2 | Homo sapiens peroxiredoxin 2 (PRDX2), nuclear gene encoding mitochondrial protein, transcript variant 3, mRNA [NM_181738] | 1.507193 | 1.66273 | -0.15554 | 0.894216 |
| ARHGEF2 | Homo sapiens Rho/Rac guanine nucleotide exchange factor (GEF) 2 (ARHGEF2), transcript variant 3, mRNA [NM_004723] | 1.382112 | 1.536021 | -0.15391 | 0.888223 |
| BCL9L | Homo sapiens B-cell CLL/lymphoma 9-like (BCL9L), mRNA [NM_182557] | 1.910621 | 2.064233 | -0.15361 | 0.960719 |
| NFIB | nuclear factor I/B [Source:HGNC Symbol;Acc:7785] [ENST00000380924] | 1.49645 | 1.650026 | -0.15358 | 0.888223 |
| NR2F1 | Homo sapiens nuclear receptor subfamily 2, group F, member 1 (NR2F1), mRNA [NM_005654] | 1.281669 | 1.434808 | -0.15314 | 0.888223 |
| ENG | Homo sapiens endoglin (ENG), transcript variant 2, mRNA [NM_000118] | 1.470934 | 1.623656 | -0.15272 | 0.891708 |
| NRCAM | Homo sapiens neuronal cell adhesion molecule (NRCAM), transcript variant 1, mRNA [NM_001037132] | 1.448779 | 1.599873 | -0.15109 | 0.888223 |
| CDSN | Homo sapiens corneodesmosin (CDSN), mRNA [NM_001264] | 1.382409 | 1.532892 | -0.15048 | 0.888223 |
| KIF5C | Homo sapiens kinesin family member 5C (KIF5C), mRNA [NM_004522] | 1.475799 | 1.625604 | -0.1498 | 0.888223 |
| TM7SF4 | Homo sapiens transmembrane 7 superfamily member 4 (TM7SF4), mRNA [NM_030788] | 1.684374 | 1.834063 | -0.14969 | 0.966905 |
| PEBP1 | Homo sapiens phosphatidylethanolamine binding protein 1 (PEBP1), mRNA [NM_002567] | 1.741263 | 1.890828 | -0.14957 | 0.913011 |
| ZGLP1 | Homo sapiens zinc finger, GATA-like protein 1 (ZGLP1), mRNA [NM_001103167] | 2.334208 | 2.48342 | -0.14921 | 0.967907 |
| GATA1 | Homo sapiens GATA binding protein 1 (globin transcription factor 1) (GATA1), mRNA [NM_002049] | 1.29057 | 1.437435 | -0.14687 | 0.888223 |
| MMD | Homo sapiens monocyte to macrophage differentiation-associated (MMD), mRNA [NM_012329] | 2.025435 | 2.17215 | -0.14671 | 0.962127 |
| GNAQ | Homo sapiens guanine nucleotide binding protein (G protein), q polypeptide (GNAQ), mRNA [NM_002072] | 1.282475 | 1.429103 | -0.14663 | 0.888223 |
| SPRR2B | Homo sapiens small proline-rich protein 2B (SPRR2B), mRNA [NM_001017418] | 1.328398 | 1.474537 | -0.14614 | 0.888223 |
| MCL1 | Homo sapiens myeloid cell leukemia sequence 1 (BCL2-related) (MCL1), nuclear gene encoding mitochondrial protein, transcript variant 1, mRNA [NM_021960] | 1.299929 | 1.445366 | -0.14544 | 0.888223 |
| TRIM10 | Homo sapiens tripartite motif containing 10 (TRIM10), transcript variant 1, mRNA [NM_006778] | 2.560269 | 2.704425 | -0.14416 | 0.94498 |
| EFNA1 | Homo sapiens ephrin-A1 (EFNA1), transcript variant 1, mRNA [NM_004428] | 1.614075 | 1.757766 | -0.14369 | 0.888223 |
| RET | Homo sapiens ret proto-oncogene (RET), transcript variant 2, mRNA [NM_020975] | 1.425528 | 1.569146 | -0.14362 | 0.888223 |
| SIAH2 | Homo sapiens seven in absentia homolog 2 (Drosophila) (SIAH2), mRNA [NM_005067] | 2.536099 | 2.679626 | -0.14353 | 0.971474 |
| COL29A1 | Homo sapiens collagen, type VI, alpha 5 (COL6A5), mRNA [NM_153264] | 1.692899 | 1.836086 | -0.14319 | 0.961613 |
| GATA4 | Homo sapiens GATA binding protein 4 (GATA4), mRNA [NM_002052] | 4.072311 | 4.215232 | -0.14292 | 0.95095 |
| WWTR1 | Homo sapiens WW domain containing transcription regulator 1 (WWTR1), transcript variant 1, mRNA [NM_015472] | 1.386672 | 1.528986 | -0.14231 | 0.888223 |
| LILRB1 | Homo sapiens leukocyte immunoglobulin-like receptor, subfamily B (with TM and ITIM domains), member 1 (LILRB1), transcript variant 1, mRNA [NM_006669] | 1.386022 | 1.528102 | -0.14208 | 0.888223 |
| PCSK4 | Homo sapiens proprotein convertase subtilisin/kexin type 4 (PCSK4), mRNA [NM_017573] | 1.428277 | 1.569673 | -0.1414 | 0.888223 |
| STX3 | Homo sapiens syntaxin 3 (STX3), transcript variant 1, mRNA [NM_004177] | 1.399775 | 1.540646 | -0.14087 | 0.888223 |
| MYB | Homo sapiens v-myb myeloblastosis viral oncogene homolog (avian) (MYB), transcript variant 2, mRNA [NM_005375] | 1.47074 | 1.611527 | -0.14079 | 0.888223 |
| IL17C | Homo sapiens interleukin 17C (IL17C), mRNA [NM_013278] | 1.312461 | 1.453123 | -0.14066 | 0.888223 |
| HRAS | Homo sapiens v-Ha-ras Harvey rat sarcoma viral oncogene homolog (HRAS), transcript variant 1, mRNA [NM_005343] | 2.843377 | 2.98293 | -0.13955 | 0.941503 |
| PITX1 | Homo sapiens paired-like homeodomain 1 (PITX1), mRNA [NM_002653] | 2.81692 | 2.954861 | -0.13794 | 0.979252 |
| MRAP | Homo sapiens melanocortin 2 receptor accessory protein (MRAP), transcript variant 2, mRNA [NM_206898] | 1.346478 | 1.483951 | -0.13747 | 0.888223 |
| AGAP2 | Homo sapiens ArfGAP with GTPase domain, ankyrin repeat and PH domain 2 (AGAP2), transcript variant 1, mRNA [NM_001122772] | 4.996475 | 5.132563 | -0.13609 | 0.960782 |
| EPO | Homo sapiens erythropoietin (EPO), mRNA [NM_000799] | 1.355987 | 1.492031 | -0.13604 | 0.888223 |
| KIF4A | Homo sapiens kinesin family member 4A (KIF4A), mRNA [NM_012310] | 1.373202 | 1.509232 | -0.13603 | 0.888223 |
| PML | Homo sapiens promyelocytic leukemia (PML), transcript variant 5, mRNA [NM_033244] | 2.372022 | 2.507972 | -0.13595 | 0.941461 |
| RAC2 | Homo sapiens ras-related C3 botulinum toxin substrate 2 (rho family, small GTP binding protein Rac2) (RAC2), mRNA [NM_002872] | 1.798494 | 1.933952 | -0.13546 | 0.960719 |
| TP73 | Homo sapiens tumor protein p73 (TP73), transcript variant 10, mRNA [NM_001204186] | 3.710736 | 3.846042 | -0.13531 | 0.956522 |
| PURB | Homo sapiens purine-rich element binding protein B (PURB), mRNA [NM_033224] | 1.363528 | 1.498621 | -0.13509 | 0.888223 |
| FSHR | Homo sapiens follicle stimulating hormone receptor (FSHR), transcript variant 1, mRNA [NM_000145] | 1.638876 | 1.77248 | -0.1336 | 0.888223 |
| CELSR2 | Homo sapiens cadherin, EGF LAG seven-pass G-type receptor 2 (flamingo homolog, Drosophila) (CELSR2), mRNA [NM_001408] | 1.553263 | 1.686822 | -0.13356 | 0.89648 |
| EPHB2 | Homo sapiens EPH receptor B2 (EPHB2), transcript variant 2, mRNA [NM_004442] | 4.970749 | 5.103659 | -0.13291 | 0.972766 |
| PLD2 | Homo sapiens phospholipase D2 (PLD2), transcript variant 1, mRNA [NM_002663] | 1.45247 | 1.58474 | -0.13227 | 0.910991 |
| CRHR1 | Homo sapiens corticotropin releasing hormone receptor 1 (CRHR1), transcript variant 4, mRNA [NM_001145148] | 3.103243 | 3.235396 | -0.13215 | 0.978384 |
| EPHA8 | Homo sapiens EPH receptor A8 (EPHA8), transcript variant 2, mRNA [NM_001006943] | 1.427915 | 1.560039 | -0.13212 | 0.904908 |
| ARHGEF10 | Rho guanine nucleotide exchange factor (GEF) 10 [Source:HGNC Symbol;Acc:14103] [ENST00000523711] | 1.673563 | 1.804984 | -0.13142 | 0.958715 |
| CRYAA | Homo sapiens crystallin, alpha A (CRYAA), mRNA [NM_000394] | 2.006199 | 2.13761 | -0.13141 | 0.961971 |
| ACHE | Homo sapiens acetylcholinesterase (ACHE), transcript variant E4-E6, mRNA [NM_000665] | 1.402554 | 1.533819 | -0.13126 | 0.888223 |
| PAX6 | Homo sapiens paired box 6 (PAX6), transcript variant 1, mRNA [NM_000280] | 1.335042 | 1.465942 | -0.1309 | 0.888223 |
| VEGFA | Homo sapiens vascular endothelial growth factor A (VEGFA), transcript variant 6, mRNA [NM_001025370] | 2.230582 | 2.361398 | -0.13082 | 0.953954 |
| AMHR2 | Homo sapiens anti-Mullerian hormone receptor, type II (AMHR2), transcript variant 1, mRNA [NM_020547] | 1.916343 | 2.046881 | -0.13054 | 0.971087 |
| AGER | Homo sapiens advanced glycosylation end product-specific receptor (AGER), transcript variant 8, mRNA [NM_001206954] | 1.853097 | 1.983594 | -0.1305 | 0.969085 |
| MCOLN3 | Homo sapiens mucolipin 3 (MCOLN3), mRNA [NM_018298] | 1.419198 | 1.54946 | -0.13026 | 0.888223 |
| ITK | Homo sapiens IL2-inducible T-cell kinase (ITK), mRNA [NM_005546] | 1.310737 | 1.440682 | -0.12995 | 0.888223 |
| CCR7 | Homo sapiens chemokine (C-C motif) receptor 7 (CCR7), mRNA [NM_001838] | 3.423454 | 3.553351 | -0.1299 | 0.982431 |
| VAMP3 | Homo sapiens vesicle-associated membrane protein 3 (cellubrevin) (VAMP3), mRNA [NM_004781] | 1.284717 | 1.414461 | -0.12974 | 0.888223 |
| FOXN1 | Homo sapiens forkhead box N1 (FOXN1), mRNA [NM_003593] | 4.728564 | 4.857891 | -0.12933 | 0.9929 |
| SPINK5 | Homo sapiens serine peptidase inhibitor, Kazal type 5 (SPINK5), transcript variant 1, mRNA [NM_001127698] | 1.743976 | 1.872936 | -0.12896 | 0.967619 |
| PRKACG | Homo sapiens protein kinase, cAMP-dependent, catalytic, gamma (PRKACG), mRNA [NM_002732] | 1.532079 | 1.660886 | -0.12881 | 0.951697 |
| ANKRD54 | Homo sapiens ankyrin repeat domain 54 (ANKRD54), transcript variant 1, mRNA [NM_138797] | 1.382428 | 1.510694 | -0.12827 | 0.888223 |
| STK11 | Homo sapiens serine/threonine kinase 11 (STK11), mRNA [NM_000455] | 2.044275 | 2.171609 | -0.12733 | 0.979472 |
| LOC401021 | homeobox D3 [Source:HGNC Symbol;Acc:5137] [ENST00000459979] | 1.293188 | 1.419834 | -0.12665 | 0.888223 |
| CDH23 | Homo sapiens cadherin-related 23 (CDH23), transcript variant 1, mRNA [NM_022124] | 1.758881 | 1.885337 | -0.12646 | 0.966539 |
| LECT1 | Homo sapiens leukocyte cell derived chemotaxin 1 (LECT1), transcript variant 1, mRNA [NM_007015] | 4.254972 | 4.381279 | -0.12631 | 0.934687 |
| SORT1 | Homo sapiens sortilin 1 (SORT1), transcript variant 1, mRNA [NM_002959] | 1.317957 | 1.444236 | -0.12628 | 0.888223 |
| MMD2 | Homo sapiens monocyte to macrophage differentiation-associated 2 (MMD2), transcript variant 2, mRNA [NM_198403] | 1.32968 | 1.455878 | -0.1262 | 0.888223 |
| NRP1 | Homo sapiens neuropilin 1 (NRP1), transcript variant 1, mRNA [NM_003873] | 1.354307 | 1.48033 | -0.12602 | 0.888223 |
| IFNG | Homo sapiens interferon, gamma (IFNG), mRNA [NM_000619] | 1.605487 | 1.731191 | -0.1257 | 0.888223 |
| WNT9B | Homo sapiens wingless-type MMTV integration site family, member 9B (WNT9B), mRNA [NM_003396] | 1.848623 | 1.974268 | -0.12565 | 0.971789 |
| CSF3 | Homo sapiens colony stimulating factor 3 (granulocyte) (CSF3), transcript variant 1, mRNA [NM_000759] | 1.960064 | 2.085216 | -0.12515 | 0.916669 |
| SMAD2 | Homo sapiens SMAD family member 2 (SMAD2), transcript variant 2, mRNA [NM_001003652] | 1.407528 | 1.531835 | -0.12431 | 0.888223 |
| CD72 | Homo sapiens CD72 molecule (CD72), mRNA [NM_001782] | 1.694116 | 1.818066 | -0.12395 | 0.972274 |
| ADRA2C | Homo sapiens adrenergic, alpha-2C-, receptor (ADRA2C), mRNA [NM_000683] | 5.98636 | 6.109863 | -0.1235 | 0.949415 |
| ESRRA | Homo sapiens estrogen-related receptor alpha (ESRRA), mRNA [NM_004451] | 1.296893 | 1.42013 | -0.12324 | 0.888223 |
| NTN4 | Homo sapiens netrin 4 (NTN4), mRNA [NM_021229] | 2.589411 | 2.710267 | -0.12086 | 0.977542 |
| FES | Homo sapiens feline sarcoma oncogene (FES), transcript variant 1, mRNA [NM_002005] | 1.313064 | 1.433521 | -0.12046 | 0.888223 |
| WNT6 | Homo sapiens wingless-type MMTV integration site family, member 6 (WNT6), mRNA [NM_006522] | 1.411032 | 1.530865 | -0.11983 | 0.911222 |
| YWHAG | Homo sapiens tyrosine 3-monooxygenase/tryptophan 5-monooxygenase activation protein, gamma polypeptide (YWHAG), mRNA [NM_012479] | 1.729419 | 1.847782 | -0.11836 | 0.965932 |
| PTPRC | Homo sapiens protein tyrosine phosphatase, receptor type, C (PTPRC), transcript variant 1, mRNA [NM_002838] | 4.679803 | 4.796339 | -0.11654 | 0.943026 |
| SLC1A3 | Homo sapiens solute carrier family 1 (glial high affinity glutamate transporter), member 3 (SLC1A3), transcript variant 3, mRNA [NM_001166696] | 1.334244 | 1.450748 | -0.1165 | 0.888223 |
| STK3 | Homo sapiens serine/threonine kinase 3 (STK3), mRNA [NM_006281] | 1.395026 | 1.509949 | -0.11492 | 0.888223 |
| TRPC4 | Homo sapiens transient receptor potential cation channel, subfamily C, member 4 (TRPC4), transcript variant alpha, mRNA [NM_016179] | 1.533891 | 1.648618 | -0.11473 | 0.916582 |
| ATXN2 | Homo sapiens ataxin 2 (ATXN2), mRNA [NM_002973] | 1.741668 | 1.855915 | -0.11425 | 0.974655 |
| TRIP13 | Homo sapiens thyroid hormone receptor interactor 13 (TRIP13), transcript variant 1, mRNA [NM_004237] | 1.597038 | 1.710802 | -0.11376 | 0.929879 |
| CYTL1 | Homo sapiens cytokine-like 1 (CYTL1), mRNA [NM_018659] | 1.403845 | 1.515678 | -0.11183 | 0.888223 |
| TRIM28 | Homo sapiens tripartite motif containing 28 (TRIM28), mRNA [NM_005762] | 1.446359 | 1.556544 | -0.11018 | 0.888223 |
| CREB3L2 | Homo sapiens cAMP responsive element binding protein 3-like 2 (CREB3L2), mRNA [NM_194071] | 3.798545 | 3.907859 | -0.10931 | 0.977044 |
| CHRD | Homo sapiens chordin (CHRD), mRNA [NM_003741] | 2.187515 | 2.296537 | -0.10902 | 0.979887 |
| BMP4 | Homo sapiens bone morphogenetic protein 4 (BMP4), transcript variant 1, mRNA [NM_001202] | 1.578998 | 1.687903 | -0.10891 | 0.888559 |
| TRIB3 | Homo sapiens tribbles homolog 3 (Drosophila) (TRIB3), mRNA [NM_021158] | 1.419024 | 1.527256 | -0.10823 | 0.888223 |
| CABYR | Homo sapiens calcium binding tyrosine-(Y)-phosphorylation regulated (CABYR), transcript variant 1, mRNA [NM_012189] | 1.604441 | 1.712651 | -0.10821 | 0.949588 |
| PLEK | Homo sapiens pleckstrin (PLEK), mRNA [NM_002664] | 3.213623 | 3.321421 | -0.1078 | 0.972357 |
| ERBB3 | Homo sapiens v-erb-b2 erythroblastic leukemia viral oncogene homolog 3 (avian) (ERBB3), transcript variant 1, mRNA [NM_001982] | 1.493623 | 1.601178 | -0.10756 | 0.916701 |
| LGALS1 | Homo sapiens lectin, galactoside-binding, soluble, 1 (LGALS1), mRNA [NM_002305] | 1.925721 | 2.033238 | -0.10752 | 0.965408 |
| NGFR | Homo sapiens nerve growth factor receptor (NGFR), mRNA [NM_002507] | 4.56301 | 4.670302 | -0.10729 | 0.958232 |
| ZFP36L1 | Homo sapiens zinc finger protein 36, C3H type-like 1 (ZFP36L1), transcript variant 1, mRNA [NM_004926] | 1.471636 | 1.578532 | -0.1069 | 0.888223 |
| MLF1 | Homo sapiens myeloid leukemia factor 1 (MLF1), transcript variant 4, mRNA [NM_001195432] | 1.326182 | 1.432488 | -0.10631 | 0.888223 |
| PLP1 | Homo sapiens proteolipid protein 1 (PLP1), transcript variant 3, mRNA [NM_001128834] | 4.946556 | 5.052421 | -0.10587 | 0.98166 |
| LCE1D | Homo sapiens late cornified envelope 1D (LCE1D), mRNA [NM_178352] | 7.023506 | 7.129359 | -0.10585 | 0.984785 |
| NBR1 | neighbor of BRCA1 gene 1 [Source:HGNC Symbol;Acc:6746] [ENST00000389311] | 1.848183 | 1.953937 | -0.10575 | 0.970921 |
| FIGNL1 | Homo sapiens fidgetin-like 1 (FIGNL1), transcript variant 1, mRNA [NM_001042762] | 2.749569 | 2.85532 | -0.10575 | 0.977832 |
| SASH3 | Homo sapiens SAM and SH3 domain containing 3 (SASH3), mRNA [NM_018990] | 1.377096 | 1.482824 | -0.10573 | 0.888223 |
| BCL11A | Homo sapiens B-cell CLL/lymphoma 11A (zinc finger protein) (BCL11A), transcript variant 1, mRNA [NM_022893] | 2.737876 | 2.843296 | -0.10542 | 0.958232 |
| MAPK1 | Homo sapiens mitogen-activated protein kinase 1 (MAPK1), transcript variant 2, mRNA [NM_138957] | 2.18085 | 2.286174 | -0.10532 | 0.96766 |
| NR0B1 | Homo sapiens nuclear receptor subfamily 0, group B, member 1 (NR0B1), mRNA [NM_000475] | 1.424897 | 1.530172 | -0.10528 | 0.917204 |
| DISC1 | Homo sapiens disrupted in schizophrenia 1 (DISC1), transcript variant b, mRNA [NM_001164538] | 1.513114 | 1.618257 | -0.10514 | 0.893606 |
| DPY19L2 | Homo sapiens dpy-19-like 2 (C. elegans) (DPY19L2), mRNA [NM_173812] | 2.164531 | 2.269205 | -0.10467 | 0.976074 |
| RPS6KA4 | Homo sapiens ribosomal protein S6 kinase, 90kDa, polypeptide 4 (RPS6KA4), transcript variant 1, mRNA [NM_003942] | 1.82821 | 1.932257 | -0.10405 | 0.977227 |
| NRG1 | Homo sapiens neuregulin 1 (NRG1), transcript variant HRG-gamma, mRNA [NM_004495] | 6.176346 | 6.280224 | -0.10388 | 0.943856 |
| MTSS1 | Homo sapiens metastasis suppressor 1 (MTSS1), mRNA [NM_014751] | 1.343855 | 1.447188 | -0.10333 | 0.888223 |
| DTNBP1 | Homo sapiens dystrobrevin binding protein 1 (DTNBP1), transcript variant 1, mRNA [NM_032122] | 2.6377 | 2.740785 | -0.10309 | 0.98968 |
| LMNA | Homo sapiens lamin A/C (LMNA), transcript variant 1, mRNA [NM_170707] | 1.484864 | 1.586858 | -0.10199 | 0.944206 |
| UNC5D | Homo sapiens unc-5 homolog D (C. elegans) (UNC5D), mRNA [NM_080872] | 1.34338 | 1.445036 | -0.10166 | 0.888223 |
| CCNB1 | Homo sapiens cyclin B1 (CCNB1), mRNA [NM_031966] | 2.989037 | 3.08986 | -0.10082 | 0.978231 |
| ENST00000423322 | Homo sapiens MSTP131 (MST131) mRNA, complete cds. [AF176921] | 1.588961 | 1.689104 | -0.10014 | 0.96766 |
| MYH14 | Homo sapiens myosin, heavy chain 14, non-muscle (MYH14), transcript variant 1, mRNA [NM_001077186] | 3.89842 | 3.998293 | -0.09987 | 0.982548 |
| PDLIM5 | Homo sapiens PDZ and LIM domain 5 (PDLIM5), transcript variant 1, mRNA [NM_006457] | 2.189272 | 2.28885 | -0.09958 | 0.981208 |
| SPTBN1 | Homo sapiens spectrin, beta, non-erythrocytic 1 (SPTBN1), transcript variant 1, mRNA [NM_003128] | 1.46314 | 1.562269 | -0.09913 | 0.912507 |
| CDC25B | Homo sapiens cell division cycle 25 homolog B (S. pombe) (CDC25B), transcript variant 1, mRNA [NM_021873] | 2.654367 | 2.753205 | -0.09884 | 0.974595 |
| LEP | Homo sapiens leptin (LEP), mRNA [NM_000230] | 1.367594 | 1.466289 | -0.09869 | 0.888223 |
| LCE1C | Homo sapiens late cornified envelope 1C (LCE1C), mRNA [NM_178351] | 1.593691 | 1.692235 | -0.09854 | 0.911451 |
| PDE3A | Homo sapiens phosphodiesterase 3A, cGMP-inhibited (PDE3A), transcript variant 1, mRNA [NM_000921] | 1.585154 | 1.682664 | -0.09751 | 0.958031 |
| BLNK | Homo sapiens B-cell linker (BLNK), transcript variant 1, mRNA [NM_013314] | 1.409517 | 1.505288 | -0.09577 | 0.888223 |
| ATP7A | Homo sapiens ATPase, Cu++ transporting, alpha polypeptide (ATP7A), mRNA [NM_000052] | 3.056661 | 3.150965 | -0.0943 | 0.981083 |
| ERAP1 | Homo sapiens endoplasmic reticulum aminopeptidase 1 (ERAP1), transcript variant 1, mRNA [NM_016442] | 1.630493 | 1.724731 | -0.09424 | 0.923348 |
| NFKBID | Homo sapiens nuclear factor of kappa light polypeptide gene enhancer in B-cells inhibitor, delta (NFKBID), mRNA [NM_139239] | 1.309965 | 1.403979 | -0.09401 | 0.888223 |
| FZD9 | Homo sapiens frizzled family receptor 9 (FZD9), mRNA [NM_003508] | 7.432516 | 7.526496 | -0.09398 | 0.973966 |
| PPP3CA | Homo sapiens protein phosphatase 3, catalytic subunit, alpha isozyme (PPP3CA), transcript variant 1, mRNA [NM_000944] | 3.294268 | 3.387721 | -0.09345 | 0.981083 |
| SMARCA4 | Homo sapiens SWI/SNF related, matrix associated, actin dependent regulator of chromatin, subfamily a, member 4 (SMARCA4), transcript variant 3, mRNA [NM_003072] | 7.784726 | 7.876628 | -0.0919 | 0.979887 |
| BRSK2 | Homo sapiens BR serine/threonine kinase 2 (BRSK2), mRNA [NM_003957] | 1.77478 | 1.86668 | -0.0919 | 0.955578 |
| ABL2 | Homo sapiens v-abl Abelson murine leukemia viral oncogene homolog 2 (ABL2), transcript variant b, mRNA [NM_007314] | 1.438459 | 1.529932 | -0.09147 | 0.888223 |
| ENST00000329335 | retinitis pigmentosa 1-like 1 [Source:HGNC Symbol;Acc:15946] [ENST00000329335] | 1.99736 | 2.088774 | -0.09141 | 0.985989 |
| A_19_P00321851 | Homo sapiens homeodomain interacting protein kinase 2 (HIPK2), transcript variant 1, mRNA [NM_022740] | 6.879737 | 6.971093 | -0.09136 | 0.951452 |
| NPHS1 | Homo sapiens nephrosis 1, congenital, Finnish type (nephrin) (NPHS1), mRNA [NM_004646] | 1.446602 | 1.537753 | -0.09115 | 0.888223 |
| ENST00000405331 | tribbles homolog 2 (Drosophila) [Source:HGNC Symbol;Acc:30809] [ENST00000405331] | 2.201629 | 2.292634 | -0.091 | 0.971789 |
| CHN2 | Homo sapiens chimerin (chimaerin) 2 (CHN2), transcript variant 2, mRNA [NM_004067] | 1.363679 | 1.45464 | -0.09096 | 0.888223 |
| NODAL | Homo sapiens nodal homolog (mouse) (NODAL), mRNA [NM_018055] | 1.571455 | 1.662085 | -0.09063 | 0.888223 |
| ARHGDIA | Homo sapiens Rho GDP dissociation inhibitor (GDI) alpha (ARHGDIA), transcript variant 2, mRNA [NM_004309] | 1.634403 | 1.724607 | -0.0902 | 0.922171 |
| SHH | Homo sapiens sonic hedgehog (SHH), mRNA [NM_000193] | 1.528443 | 1.618188 | -0.08975 | 0.888223 |
| NCAM1 | Homo sapiens neural cell adhesion molecule 1 (NCAM1), transcript variant 5, mRNA [NM_001242607] | 1.362675 | 1.452229 | -0.08955 | 0.888223 |
| HOXA13 | Homo sapiens homeobox A13 (HOXA13), mRNA [NM_000522] | 1.418172 | 1.507268 | -0.0891 | 0.888223 |
| HIF1A | Homo sapiens hypoxia inducible factor 1, alpha subunit (basic helix-loop-helix transcription factor) (HIF1A), transcript variant 2, mRNA [NM_181054] | 1.315994 | 1.404161 | -0.08817 | 0.888223 |
| AP2B1 | Homo sapiens adaptor-related protein complex 2, beta 1 subunit (AP2B1), transcript variant 1, mRNA [NM_001030006] | 1.443482 | 1.531308 | -0.08783 | 0.905923 |
| PPP3R1 | Homo sapiens protein phosphatase 3, regulatory subunit B, alpha (PPP3R1), mRNA [NM_000945] | 3.514916 | 3.60177 | -0.08685 | 0.972357 |
| MTSS1 | Homo sapiens metastasis suppressor 1 (MTSS1), mRNA [NM_014751] | 1.671673 | 1.757941 | -0.08627 | 0.955576 |
| LPL | Homo sapiens lipoprotein lipase (LPL), mRNA [NM_000237] | 1.769994 | 1.856141 | -0.08615 | 0.973794 |
| PAFAH1B1 | Homo sapiens platelet-activating factor acetylhydrolase 1b, regulatory subunit 1 (45kDa) (PAFAH1B1), mRNA [NM_000430] | 1.779736 | 1.865773 | -0.08604 | 0.979887 |
| UCP1 | Homo sapiens uncoupling protein 1 (mitochondrial, proton carrier) (UCP1), nuclear gene encoding mitochondrial protein, mRNA [NM_021833] | 1.469462 | 1.555022 | -0.08556 | 0.888223 |
| CTBP1 | Homo sapiens C-terminal binding protein 1 (CTBP1), transcript variant 2, mRNA [NM_001012614] | 1.465479 | 1.550749 | -0.08527 | 0.951631 |
| NOC3L | Homo sapiens nucleolar complex associated 3 homolog (S. cerevisiae) (NOC3L), mRNA [NM_022451] | 1.353668 | 1.438918 | -0.08525 | 0.888223 |
| EID1 | Homo sapiens EP300 interacting inhibitor of differentiation 1 (EID1), mRNA [NM_014335] | 1.440878 | 1.524059 | -0.08318 | 0.888223 |
| CNTNAP2 | Homo sapiens contactin associated protein-like 2 (CNTNAP2), mRNA [NM_014141] | 1.554202 | 1.637151 | -0.08295 | 0.952288 |
| LPPR4 | Homo sapiens lipid phosphate phosphatase-related protein type 4 (LPPR4), transcript variant 1, mRNA [NM_014839] | 1.765968 | 1.84867 | -0.0827 | 0.971602 |
| VEGFC | Homo sapiens vascular endothelial growth factor C (VEGFC), mRNA [NM_005429] | 1.329687 | 1.410361 | -0.08067 | 0.888223 |
| PRNP | Homo sapiens prion protein (PRNP), transcript variant 1, mRNA [NM_000311] | 1.44883 | 1.529095 | -0.08026 | 0.888223 |
| MED1 | Homo sapiens mediator complex subunit 1 (MED1), mRNA [NM_004774] | 1.527438 | 1.607655 | -0.08022 | 0.95461 |
| NLGN1 | Homo sapiens neuroligin 1 (NLGN1), mRNA [NM_014932] | 1.331873 | 1.411067 | -0.07919 | 0.888223 |
| EPHA4 | Homo sapiens EPH receptor A4 (EPHA4), mRNA [NM_004438] | 1.759529 | 1.838554 | -0.07902 | 0.935544 |
| RAB3A | Homo sapiens RAB3A, member RAS oncogene family (RAB3A), mRNA [NM_002866] | 2.436944 | 2.514695 | -0.07775 | 0.98593 |
| A_19_P00316200 | Homo sapiens striatin, calmodulin binding protein (STRN), mRNA [NM_003162] | 1.29118 | 1.368678 | -0.0775 | 0.888223 |
| FZD4 | Homo sapiens frizzled family receptor 4 (FZD4), mRNA [NM_012193] | 1.357369 | 1.434795 | -0.07743 | 0.888223 |
| CLN8 | Homo sapiens ceroid-lipofuscinosis, neuronal 8 (epilepsy, progressive with mental retardation) (CLN8), mRNA [NM_018941] | 1.282775 | 1.359759 | -0.07698 | 0.888223 |
| LHX4 | Homo sapiens LIM homeobox 4 (LHX4), mRNA [NM_033343] | 1.457883 | 1.534054 | -0.07617 | 0.888223 |
| PPT1 | Homo sapiens palmitoyl-protein thioesterase 1 (PPT1), transcript variant 1, mRNA [NM_000310] | 1.43012 | 1.50524 | -0.07512 | 0.888223 |
| HES5 | Homo sapiens hairy and enhancer of split 5 (Drosophila) (HES5), mRNA [NM_001010926] | 1.645547 | 1.720507 | -0.07496 | 0.973782 |
| AP3D1 | Homo sapiens adaptor-related protein complex 3, delta 1 subunit (AP3D1), transcript variant 2, mRNA [NM_003938] | 1.475702 | 1.550383 | -0.07468 | 0.899333 |
| FOXC1 | Homo sapiens forkhead box C1 (FOXC1), mRNA [NM_001453] | 1.316297 | 1.390128 | -0.07383 | 0.888223 |
| BMP7 | Homo sapiens bone morphogenetic protein 7 (BMP7), mRNA [NM_001719] | 1.573823 | 1.647065 | -0.07324 | 0.888223 |
| ASPM | Homo sapiens asp (abnormal spindle) homolog, microcephaly associated (Drosophila) (ASPM), transcript variant 1, mRNA [NM_018136] | 1.364296 | 1.43731 | -0.07301 | 0.888223 |
| CAT | Homo sapiens catalase (CAT), mRNA [NM_001752] | 1.406648 | 1.477122 | -0.07047 | 0.888223 |
| SCN8A | Homo sapiens sodium channel, voltage gated, type VIII, alpha subunit (SCN8A), transcript variant 1, mRNA [NM_014191] | 1.558075 | 1.628266 | -0.07019 | 0.941501 |
| ENST00000290863 | angiotensin I converting enzyme (peptidyl-dipeptidase A) 1 [Source:HGNC Symbol;Acc:2707] [ENST00000490216] | 1.34601 | 1.414149 | -0.06814 | 0.888223 |
| FEZ2 | Homo sapiens fasciculation and elongation protein zeta 2 (zygin II) (FEZ2), transcript variant 2, mRNA [NM_001042548] | 6.235358 | 6.302665 | -0.06731 | 0.99011 |
| SPTBN4 | Homo sapiens spectrin, beta, non-erythrocytic 4 (SPTBN4), transcript variant sigma1, mRNA [NM_020971] | 3.640075 | 3.707214 | -0.06714 | 0.977328 |
| BTG1 | Homo sapiens B-cell translocation gene 1, anti-proliferative (BTG1), mRNA [NM_001731] | 2.425528 | 2.492486 | -0.06696 | 0.982548 |
| RORA | Homo sapiens RAR-related orphan receptor A (RORA), transcript variant 2, mRNA [NM_134260] | 1.302161 | 1.368514 | -0.06635 | 0.888223 |
| LIG4 | Homo sapiens ligase IV, DNA, ATP-dependent (LIG4), transcript variant 1, mRNA [NM_002312] | 1.395895 | 1.461997 | -0.0661 | 0.888223 |
| CXCL12 | Homo sapiens chemokine (C-X-C motif) ligand 12 (CXCL12), transcript variant 3, mRNA [NM_001033886] | 2.394967 | 2.459788 | -0.06482 | 0.989695 |
| MYO5A | Homo sapiens myosin VA (heavy chain 12, myoxin) (MYO5A), transcript variant 1, mRNA [NM_000259] | 1.401193 | 1.465874 | -0.06468 | 0.888223 |
| LIMK1 | Homo sapiens LIM domain kinase 1 (LIMK1), transcript variant 1, mRNA [NM_002314] | 1.492453 | 1.556374 | -0.06392 | 0.888223 |
| PDE5A | Homo sapiens phosphodiesterase 5A, cGMP-specific (PDE5A), transcript variant 1, mRNA [NM_001083] | 1.483796 | 1.547595 | -0.0638 | 0.943795 |
| CTNND1 | Homo sapiens catenin (cadherin-associated protein), delta 1 (CTNND1), transcript variant 16, mRNA [NM_001206885] | 3.412223 | 3.475491 | -0.06327 | 0.974551 |
| WWOX | Homo sapiens WW domain containing oxidoreductase (WWOX), transcript variant 2, mRNA [NM_130791] | 1.799061 | 1.861419 | -0.06236 | 0.979957 |
| COL4A2 | Homo sapiens collagen, type IV, alpha 2 (COL4A2), mRNA [NM_001846] | 2.50655 | 2.568724 | -0.06217 | 0.979274 |
| EDNRA | Homo sapiens endothelin receptor type A (EDNRA), transcript variant 1, mRNA [NM_001957] | 1.84031 | 1.902157 | -0.06185 | 0.984934 |
| TTN | titin [Source:HGNC Symbol;Acc:12403] [ENST00000392423] | 1.989465 | 2.051281 | -0.06182 | 0.974763 |
| PF4 | Homo sapiens platelet factor 4 (PF4), mRNA [NM_002619] | 2.479935 | 2.541109 | -0.06117 | 0.98732 |
| ZNF703 | Homo sapiens zinc finger protein 703 (ZNF703), mRNA [NM_025069] | 1.503699 | 1.564138 | -0.06044 | 0.971328 |
| RASA1 | Homo sapiens RAS p21 protein activator (GTPase activating protein) 1 (RASA1), transcript variant 1, mRNA [NM_002890] | 1.433796 | 1.493989 | -0.06019 | 0.888223 |
| RNF6 | Homo sapiens ring finger protein (C3H2C3 type) 6 (RNF6), transcript variant 1, mRNA [NM_005977] | 1.540634 | 1.600356 | -0.05972 | 0.952152 |
| SPANXB2 | Homo sapiens SPANX family, member B2 (SPANXB2), mRNA [NM_145664] | 5.352349 | 5.411961 | -0.05961 | 0.985302 |
| NR2F6 | Homo sapiens nuclear receptor subfamily 2, group F, member 6 (NR2F6), mRNA [NM_005234] | 1.573356 | 1.631632 | -0.05828 | 0.978216 |
| PIAS2 | Homo sapiens protein inhibitor of activated STAT, 2 (PIAS2), transcript variant beta, mRNA [NM_004671] | 3.266067 | 3.3235 | -0.05743 | 0.977633 |
| HOXA5 | Homo sapiens homeobox A5 (HOXA5), mRNA [NM_019102] | 2.693604 | 2.750964 | -0.05736 | 0.987798 |
| TBX3 | Homo sapiens T-box 3 (TBX3), transcript variant 2, mRNA [NM_016569] | 1.367528 | 1.424595 | -0.05707 | 0.888223 |
| ACVR2A | Homo sapiens activin A receptor, type IIA (ACVR2A), mRNA [NM_001616] | 1.410435 | 1.467266 | -0.05683 | 0.914733 |
| ARHGEF11 | Homo sapiens Rho guanine nucleotide exchange factor (GEF) 11 (ARHGEF11), transcript variant 2, mRNA [NM_198236] | 1.507481 | 1.563191 | -0.05571 | 0.972541 |
| IMPAD1 | Homo sapiens inositol monophosphatase domain containing 1 (IMPAD1), mRNA [NM_017813] | 2.250541 | 2.306171 | -0.05563 | 0.984984 |
| NPHP3 | Homo sapiens nephronophthisis 3 (adolescent) (NPHP3), mRNA [NM_153240] | 1.423169 | 1.478716 | -0.05555 | 0.916701 |
| FOXM1 | Homo sapiens forkhead box M1 (FOXM1), transcript variant 1, mRNA [NM_202002] | 2.374852 | 2.42982 | -0.05497 | 0.987843 |
| ERCC2 | Homo sapiens excision repair cross-complementing rodent repair deficiency, complementation group 2 (ERCC2), transcript variant 1, mRNA [NM_000400] | 3.987783 | 4.042742 | -0.05496 | 0.993502 |
| AGRN | Homo sapiens agrin (AGRN), mRNA [NM_198576] | 3.639467 | 3.694341 | -0.05487 | 0.989695 |
| ROBO2 | Homo sapiens roundabout, axon guidance receptor, homolog 2 (Drosophila) (ROBO2), transcript variant 2, mRNA [NM_002942] | 2.229383 | 2.283737 | -0.05435 | 0.983382 |
| AVIL | Homo sapiens advillin (AVIL), mRNA [NM_006576] | 2.020061 | 2.07426 | -0.0542 | 0.987843 |
| HPRT1 | Homo sapiens hypoxanthine phosphoribosyltransferase 1 (HPRT1), mRNA [NM_000194] | 1.741487 | 1.795348 | -0.05386 | 0.987965 |
| CNTN6 | Homo sapiens contactin 6 (CNTN6), mRNA [NM_014461] | 1.72875 | 1.781933 | -0.05318 | 0.984844 |
| MAPT | Homo sapiens microtubule-associated protein tau (MAPT), transcript variant 1, mRNA [NM_016835] | 1.313294 | 1.366023 | -0.05273 | 0.888223 |
| ENST00000326470 | Homo sapiens slingshot homolog 1 (Drosophila) (SSH1), transcript variant 3, mRNA [NM_001161331] | 1.419716 | 1.471738 | -0.05202 | 0.957912 |
| PBX3 | Homo sapiens pre-B-cell leukemia homeobox 3 (PBX3), transcript variant 1, mRNA [NM_006195] | 1.364706 | 1.416406 | -0.0517 | 0.89938 |
| SPON2 | Homo sapiens spondin 2, extracellular matrix protein (SPON2), transcript variant 1, mRNA [NM_012445] | 1.515206 | 1.566434 | -0.05123 | 0.945624 |
| PAX6 | Homo sapiens paired box 6 (PAX6), transcript variant 1, mRNA [NM_000280] | 1.479876 | 1.530925 | -0.05105 | 0.971936 |
| KLF4 | Homo sapiens Kruppel-like factor 4 (gut) (KLF4), mRNA [NM_004235] | 1.553657 | 1.604571 | -0.05091 | 0.923937 |
| ID4 | Homo sapiens inhibitor of DNA binding 4, dominant negative helix-loop-helix protein (ID4), mRNA [NM_001546] | 1.290365 | 1.341154 | -0.05079 | 0.888223 |
| COL4A3 | Homo sapiens collagen, type IV, alpha 3 (Goodpasture antigen) (COL4A3), mRNA [NM_000091] | 1.566879 | 1.617102 | -0.05022 | 0.951646 |
| NDEL1 | Homo sapiens nudE nuclear distribution gene E homolog (A. nidulans)-like 1 (NDEL1), transcript variant 1, mRNA [NM_001025579] | 1.691521 | 1.740734 | -0.04921 | 0.97805 |
| ZEB1 | Homo sapiens zinc finger E-box binding homeobox 1 (ZEB1), transcript variant 1, mRNA [NM_001128128] | 1.409646 | 1.458719 | -0.04907 | 0.888223 |
| YWHAH | Homo sapiens tyrosine 3-monooxygenase/tryptophan 5-monooxygenase activation protein, eta polypeptide (YWHAH), mRNA [NM_003405] | 1.409788 | 1.458587 | -0.0488 | 0.910985 |
| FANCA | Homo sapiens Fanconi anemia, complementation group A (FANCA), transcript variant 2, mRNA [NM_001018112] | 1.343746 | 1.392534 | -0.04879 | 0.894841 |
| KIAA1598 | Homo sapiens KIAA1598 (KIAA1598), transcript variant 2, mRNA [NM_018330] | 2.511822 | 2.560351 | -0.04853 | 0.98732 |
| MPP5 | Homo sapiens membrane protein, palmitoylated 5 (MAGUK p55 subfamily member 5) (MPP5), mRNA [NM_022474] | 1.425304 | 1.473673 | -0.04837 | 0.888223 |
| LTA4H | Homo sapiens leukotriene A4 hydrolase (LTA4H), mRNA [NM_000895] | 1.754578 | 1.802916 | -0.04834 | 0.982578 |
| GRM5 | Homo sapiens glutamate receptor, metabotropic 5 (GRM5), transcript variant b, mRNA [NM_000842] | 1.43746 | 1.484774 | -0.04731 | 0.888223 |
| KRT2 | Homo sapiens keratin 2 (KRT2), mRNA [NM_000423] | 1.401231 | 1.44849 | -0.04726 | 0.888223 |
| PTGS2 | Homo sapiens prostaglandin-endoperoxide synthase 2 (prostaglandin G/H synthase and cyclooxygenase) (PTGS2), mRNA [NM_000963] | 1.396557 | 1.443723 | -0.04717 | 0.888223 |
| CYP24A1 | Homo sapiens cytochrome P450, family 24, subfamily A, polypeptide 1 (CYP24A1), nuclear gene encoding mitochondrial protein, transcript variant 1, mRNA [NM_000782] | 1.33486 | 1.381011 | -0.04615 | 0.888223 |
| NRIP1 | Homo sapiens nuclear receptor interacting protein 1 (NRIP1), mRNA [NM_003489] | 1.301158 | 1.347009 | -0.04585 | 0.888223 |
| NPM2 | Homo sapiens nucleophosmin/nucleoplasmin 2 (NPM2), mRNA [NM_182795] | 1.673646 | 1.719408 | -0.04576 | 0.983817 |
| HSD17B3 | Homo sapiens hydroxysteroid (17-beta) dehydrogenase 3 (HSD17B3), mRNA [NM_000197] | 1.370022 | 1.41461 | -0.04459 | 0.908164 |
| NRG1 | Homo sapiens neuregulin 1 (NRG1), transcript variant HRG-gamma, mRNA [NM_004495] | 2.884921 | 2.92861 | -0.04369 | 0.988697 |
| SEMA4A | Homo sapiens sema domain, immunoglobulin domain (Ig), transmembrane domain (TM) and short cytoplasmic domain, (semaphorin) 4A (SEMA4A), transcript variant 1, mRNA [NM_022367] | 2.192623 | 2.23559 | -0.04297 | 0.986704 |
| HOXB5 | Homo sapiens homeobox B5 (HOXB5), mRNA [NM_002147] | 2.177048 | 2.219291 | -0.04224 | 0.991368 |
| NTF3 | Homo sapiens neurotrophin 3 (NTF3), transcript variant 2, mRNA [NM_002527] | 2.600393 | 2.642608 | -0.04221 | 0.988432 |
| MRAP | Homo sapiens melanocortin 2 receptor accessory protein (MRAP), transcript variant 1, mRNA [NM_178817] | 1.446798 | 1.488618 | -0.04182 | 0.888223 |
| STIP1 | Homo sapiens stress-induced-phosphoprotein 1 (STIP1), mRNA [NM_006819] | 2.401642 | 2.442914 | -0.04127 | 0.985113 |
| OLIG2 | Homo sapiens oligodendrocyte lineage transcription factor 2 (OLIG2), mRNA [NM_005806] | 1.653742 | 1.694526 | -0.04078 | 0.987843 |
| PRDM1 | Homo sapiens PR domain containing 1, with ZNF domain (PRDM1), transcript variant 1, mRNA [NM_001198] | 1.353034 | 1.393379 | -0.04035 | 0.888223 |
| UTF1 | Homo sapiens undifferentiated embryonic cell transcription factor 1 (UTF1), mRNA [NM_003577] | 5.430795 | 5.470896 | -0.0401 | 0.990727 |
| FOXD4 | Homo sapiens forkhead box D4 (FOXD4), mRNA [NM_207305] | 1.643148 | 1.683184 | -0.04004 | 0.984687 |
| CENPI | centromere protein I [Source:HGNC Symbol;Acc:3968] [ENST00000403304] | 1.334332 | 1.37316 | -0.03883 | 0.888223 |
| ENST00000370442 | small ArfGAP 1 [Source:HGNC Symbol;Acc:19651] [ENST00000370442] | 1.395863 | 1.434111 | -0.03825 | 0.888223 |
| KCNQ3 | Homo sapiens potassium voltage-gated channel, KQT-like subfamily, member 3 (KCNQ3), transcript variant 1, mRNA [NM_004519] | 1.411345 | 1.449221 | -0.03788 | 0.888223 |
| FOXP1 | Homo sapiens forkhead box P1 (FOXP1), transcript variant 3, mRNA [NM_001244808] | 12.96467 | 13.00145 | -0.03678 | 0.990024 |
| RUNX1 | Homo sapiens runt-related transcription factor 1 (RUNX1), transcript variant 2, mRNA [NM_001001890] | 1.34714 | 1.383671 | -0.03653 | 0.888223 |
| CETP | Homo sapiens cholesteryl ester transfer protein, plasma (CETP), mRNA [NM_000078] | 1.541983 | 1.578261 | -0.03628 | 0.976503 |
| SGCZ | Homo sapiens sarcoglycan, zeta (SGCZ), mRNA [NM_139167] | 1.340655 | 1.376853 | -0.0362 | 0.888223 |
| MAPK8IP3 | Homo sapiens mitogen-activated protein kinase 8 interacting protein 3 (MAPK8IP3), transcript variant 1, mRNA [NM_015133] | 1.572167 | 1.608325 | -0.03616 | 0.945168 |
| ETV1 | Homo sapiens ets variant 1 (ETV1), transcript variant 1, mRNA [NM_004956] | 2.60314 | 2.63924 | -0.0361 | 0.99283 |
| CYP26C1 | Homo sapiens mRNA for FLJ00329 protein. [AK131099] | 1.72757 | 1.763135 | -0.03556 | 0.98359 |
| HSP90AA1 | Homo sapiens heat shock protein 90kDa alpha (cytosolic), class A member 1 (HSP90AA1), transcript variant 2, mRNA [NM_005348] | 1.42225 | 1.457424 | -0.03517 | 0.888223 |
| UPK1A | Homo sapiens uroplakin 1A (UPK1A), mRNA [NM_007000] | 2.246454 | 2.281435 | -0.03498 | 0.991086 |
| CACYBP | Homo sapiens calcyclin binding protein (CACYBP), transcript variant 1, mRNA [NM_014412] | 1.664032 | 1.698334 | -0.0343 | 0.980555 |
| ESRRB | Homo sapiens estrogen-related receptor beta (ESRRB), mRNA [NM_004452] | 1.633245 | 1.66753 | -0.03428 | 0.988266 |
| SOS2 | Homo sapiens son of sevenless homolog 2 (Drosophila) (SOS2), mRNA [NM_006939] | 1.3391 | 1.373254 | -0.03415 | 0.888223 |
| DICER1 | Homo sapiens dicer 1, ribonuclease type III (DICER1), transcript variant 3, mRNA [NM_001195573] | 1.685939 | 1.719072 | -0.03313 | 0.983382 |
| MKKS | Homo sapiens McKusick-Kaufman syndrome (MKKS), transcript variant 2, mRNA [NM_170784] | 1.581058 | 1.613968 | -0.03291 | 0.98166 |
| CBX2 | Homo sapiens chromobox homolog 2 (CBX2), transcript variant 1, mRNA [NM_005189] | 1.518849 | 1.551474 | -0.03263 | 0.963794 |
| HPS4 | Homo sapiens Hermansky-Pudlak syndrome 4 (HPS4), transcript variant 1, mRNA [NM_022081] | 1.379276 | 1.411448 | -0.03217 | 0.888223 |
| HFE2 | Homo sapiens hemochromatosis type 2 (juvenile) (HFE2), transcript variant a, mRNA [NM_213653] | 1.929809 | 1.960754 | -0.03095 | 0.991586 |
| IL18 | Homo sapiens interleukin 18 (interferon-gamma-inducing factor) (IL18), transcript variant 1, mRNA [NM_001562] | 1.393664 | 1.424407 | -0.03074 | 0.919184 |
| HOPX | Homo sapiens HOP homeobox (HOPX), transcript variant 5, mRNA [NM_001145460] | 1.413539 | 1.444277 | -0.03074 | 0.888223 |
| KDR | Homo sapiens kinase insert domain receptor (a type III receptor tyrosine kinase) (KDR), mRNA [NM_002253] | 3.02238 | 3.052927 | -0.03055 | 0.981032 |
| KIAA0319 | Homo sapiens KIAA0319 (KIAA0319), transcript variant 1, mRNA [NM_014809] | 1.487832 | 1.517971 | -0.03014 | 0.888223 |
| CXCL12 | Homo sapiens chemokine (C-X-C motif) ligand 12 (CXCL12), transcript variant 2, mRNA [NM_000609] | 1.463619 | 1.493727 | -0.03011 | 0.888223 |
| MAP1B | Homo sapiens microtubule-associated protein 1B (MAP1B), mRNA [NM_005909] | 1.354643 | 1.38458 | -0.02994 | 0.888223 |
| LHB | Homo sapiens luteinizing hormone beta polypeptide (LHB), mRNA [NM_000894] | 2.642943 | 2.672201 | -0.02926 | 0.993004 |
| TGFBR2 | Homo sapiens transforming growth factor, beta receptor II (70/80kDa) (TGFBR2), transcript variant 1, mRNA [NM_001024847] | 1.530356 | 1.559491 | -0.02914 | 0.98443 |
| IL2 | Homo sapiens interleukin 2 (IL2), mRNA [NM_000586] | 1.490553 | 1.519601 | -0.02905 | 0.970182 |
| CYP24A1 | Homo sapiens cytochrome P450, family 24, subfamily A, polypeptide 1 (CYP24A1), nuclear gene encoding mitochondrial protein, transcript variant 1, mRNA [NM_000782] | 1.558084 | 1.586962 | -0.02888 | 0.977444 |
| CBY1 | Homo sapiens chibby homolog 1 (Drosophila) (CBY1), transcript variant 2, mRNA [NM_001002880] | 1.627925 | 1.656687 | -0.02876 | 0.987366 |
| CDH1 | Homo sapiens cadherin 1, type 1, E-cadherin (epithelial) (CDH1), mRNA [NM_004360] | 1.36674 | 1.394579 | -0.02784 | 0.888223 |
| DTX1 | Homo sapiens deltex homolog 1 (Drosophila) (DTX1), mRNA [NM_004416] | 1.402631 | 1.430032 | -0.0274 | 0.888223 |
| WNT5A | Homo sapiens wingless-type MMTV integration site family, member 5A (WNT5A), mRNA [NM_003392] | 1.397506 | 1.424797 | -0.02729 | 0.888223 |
| NOS1 | Homo sapiens nitric oxide synthase 1 (neuronal) (NOS1), transcript variant 1, mRNA [NM_000620] | 1.563286 | 1.590466 | -0.02718 | 0.96846 |
| TIPARP | Homo sapiens TCDD-inducible poly(ADP-ribose) polymerase (TIPARP), transcript variant 1, mRNA [NM_001184717] | 1.457618 | 1.484649 | -0.02703 | 0.888223 |
| SOX18 | Homo sapiens SRY (sex determining region Y)-box 18 (SOX18), mRNA [NM_018419] | 1.395478 | 1.421838 | -0.02636 | 0.888223 |
| MNDA | Homo sapiens myeloid cell nuclear differentiation antigen (MNDA), mRNA [NM_002432] | 3.265529 | 3.291856 | -0.02633 | 0.997124 |
| ADAMTS1 | Homo sapiens ADAM metallopeptidase with thrombospondin type 1 motif, 1 (ADAMTS1), mRNA [NM_006988] | 1.502831 | 1.528812 | -0.02598 | 0.894891 |
| GLI1 | Homo sapiens GLI family zinc finger 1 (GLI1), transcript variant 1, mRNA [NM_005269] | 1.39951 | 1.425373 | -0.02586 | 0.888223 |
| NKX3-2 | Homo sapiens NK3 homeobox 2 (NKX3-2), mRNA [NM_001189] | 1.582703 | 1.608426 | -0.02572 | 0.984687 |
| NPDC1 | Homo sapiens neural proliferation, differentiation and control, 1 (NPDC1), mRNA [NM_015392] | 2.951487 | 2.977205 | -0.02572 | 0.994893 |
| FBN2 | Homo sapiens fibrillin 2 (FBN2), mRNA [NM_001999] | 2.658527 | 2.684106 | -0.02558 | 0.994071 |
| ECT2 | Homo sapiens epithelial cell transforming sequence 2 oncogene (ECT2), mRNA [NM_018098] | 1.704483 | 1.7299 | -0.02542 | 0.984729 |
| SHC1 | Homo sapiens SHC (Src homology 2 domain containing) transforming protein 1 (SHC1), transcript variant 2, mRNA [NM_003029] | 1.382614 | 1.407952 | -0.02534 | 0.906114 |
| MAL | Homo sapiens mal, T-cell differentiation protein (MAL), transcript variant a, mRNA [NM_002371] | 1.418451 | 1.443785 | -0.02533 | 0.888223 |
| BRUNOL4 | Homo sapiens CUGBP, Elav-like family member 4 (CELF4), transcript variant 1, mRNA [NM_020180] | 1.366963 | 1.392221 | -0.02526 | 0.888223 |
| TTN | Homo sapiens titin (TTN), transcript variant novex-3, mRNA [NM_133379] | 1.343194 | 1.368199 | -0.02501 | 0.894891 |
| CTNNBIP1 | Homo sapiens catenin, beta interacting protein 1 (CTNNBIP1), transcript variant 1, mRNA [NM_020248] | 1.419289 | 1.443809 | -0.02452 | 0.888223 |
| CYB5R4 | Homo sapiens cytochrome b5 reductase 4 (CYB5R4), mRNA [NM_016230] | 1.518108 | 1.542597 | -0.02449 | 0.927004 |
| ST8SIA2 | Homo sapiens ST8 alpha-N-acetyl-neuraminide alpha-2,8-sialyltransferase 2 (ST8SIA2), mRNA [NM_006011] | 1.392161 | 1.41643 | -0.02427 | 0.888223 |
| GDF7 | Homo sapiens growth differentiation factor 7 (GDF7), mRNA [NM_182828] | 4.697004 | 4.721211 | -0.02421 | 0.995026 |
| RELA | Homo sapiens v-rel reticuloendotheliosis viral oncogene homolog A (avian) (RELA), transcript variant 1, mRNA [NM_021975] | 2.234287 | 2.258062 | -0.02377 | 0.990926 |
| PPARA | Homo sapiens peroxisome proliferator-activated receptor alpha (PPARA), transcript variant 5, mRNA [NM_005036] | 1.406784 | 1.43007 | -0.02329 | 0.888223 |
| BAD | BCL2-associated agonist of cell death [Source:HGNC Symbol;Acc:936] [ENST00000544271] | 1.574585 | 1.597766 | -0.02318 | 0.972274 |
| SEMA5A | Homo sapiens sema domain, seven thrombospondin repeats (type 1 and type 1-like), transmembrane domain (TM) and short cytoplasmic domain, (semaphorin) 5A (SEMA5A), mRNA [NM_003966] | 5.30018 | 5.323289 | -0.02311 | 0.989698 |
| IGFBP3 | Homo sapiens insulin-like growth factor binding protein 3 (IGFBP3), transcript variant 1, mRNA [NM_001013398] | 1.398464 | 1.421558 | -0.02309 | 0.888223 |
| TNMD | Homo sapiens tenomodulin (TNMD), mRNA [NM_022144] | 1.454228 | 1.476695 | -0.02247 | 0.888223 |
| CD36 | Homo sapiens CD36 molecule (thrombospondin receptor) (CD36), transcript variant 2, mRNA [NM_001001547] | 2.072124 | 2.093994 | -0.02187 | 0.98593 |
| ABL2 | Homo sapiens v-abl Abelson murine leukemia viral oncogene homolog 2 (ABL2), transcript variant b, mRNA [NM_007314] | 1.76179 | 1.783588 | -0.0218 | 0.992944 |
| CYP19A1 | Homo sapiens cytochrome P450, family 19, subfamily A, polypeptide 1 (CYP19A1), transcript variant 2, mRNA [NM_031226] | 1.451779 | 1.473342 | -0.02156 | 0.888223 |
| CYP27B1 | Homo sapiens cytochrome P450, family 27, subfamily B, polypeptide 1 (CYP27B1), nuclear gene encoding mitochondrial protein, mRNA [NM_000785] | 1.383885 | 1.405403 | -0.02152 | 0.888223 |
| MYO1E | Homo sapiens myosin IE (MYO1E), mRNA [NM_004998] | 1.463039 | 1.48427 | -0.02123 | 0.888223 |
| MYH9 | Homo sapiens myosin, heavy chain 9, non-muscle (MYH9), mRNA [NM_002473] | 1.414144 | 1.435236 | -0.02109 | 0.888223 |
| A_19_P00812250 | Homo sapiens SMAD family member 4 (SMAD4), mRNA [NM_005359] | 1.426488 | 1.447521 | -0.02103 | 0.888223 |
| FGF23 | Homo sapiens fibroblast growth factor 23 (FGF23), mRNA [NM_020638] | 1.46422 | 1.485172 | -0.02095 | 0.888223 |
| CASP2 | Homo sapiens caspase 2, apoptosis-related cysteine peptidase (CASP2), transcript variant 1, mRNA [NM_032982] | 4.168223 | 4.189115 | -0.02089 | 0.994142 |
| NTRK3 | Homo sapiens neurotrophic tyrosine kinase, receptor, type 3 (NTRK3), transcript variant 2, mRNA [NM_002530] | 1.59616 | 1.616614 | -0.02045 | 0.990298 |
| JUN | Homo sapiens jun proto-oncogene (JUN), mRNA [NM_002228] | 1.388307 | 1.408746 | -0.02044 | 0.888223 |
| ARHGEF10 | Homo sapiens Rho guanine nucleotide exchange factor (GEF) 10 (ARHGEF10), mRNA [NM_014629] | 1.537828 | 1.558237 | -0.02041 | 0.990792 |
| RC3H1 | Homo sapiens ring finger and CCCH-type domains 1 (RC3H1), mRNA [NM_172071] | 1.426002 | 1.446359 | -0.02036 | 0.895701 |
| TYMS | Homo sapiens thymidylate synthetase (TYMS), mRNA [NM_001071] | 1.636477 | 1.656827 | -0.02035 | 0.990926 |
| ADAD1 | Homo sapiens adenosine deaminase domain containing 1 (testis-specific) (ADAD1), transcript variant 1, mRNA [NM_139243] | 1.440563 | 1.460707 | -0.02014 | 0.903673 |
| OGT | Homo sapiens O-linked N-acetylglucosamine (GlcNAc) transferase (UDP-N-acetylglucosamine:polypeptide-N-acetylglucosaminyl transferase) (OGT), transcript variant 1, mRNA [NM_181672] | 1.450387 | 1.470506 | -0.02012 | 0.888223 |
| SFRP5 | Homo sapiens secreted frizzled-related protein 5 (SFRP5), mRNA [NM_003015] | 1.420054 | 1.439907 | -0.01985 | 0.888223 |
| DCLRE1C | Homo sapiens DNA cross-link repair 1C (DCLRE1C), transcript variant c, mRNA [NM_001033858] | 1.37465 | 1.394427 | -0.01978 | 0.888223 |
| ANGPT2 | Homo sapiens angiopoietin 2 (ANGPT2), transcript variant 1, mRNA [NM_001147] | 1.361389 | 1.381091 | -0.0197 | 0.921551 |
| KLHL1 | Homo sapiens kelch-like 1 (Drosophila) (KLHL1), mRNA [NM_020866] | 1.40229 | 1.421831 | -0.01954 | 0.888223 |
| RIPK2 | Homo sapiens receptor-interacting serine-threonine kinase 2 (RIPK2), mRNA [NM_003821] | 3.002929 | 3.022195 | -0.01927 | 0.992107 |
| DISC1 | Homo sapiens disrupted in schizophrenia 1 (DISC1), transcript variant n, mRNA [NM_001164551] | 1.45946 | 1.478639 | -0.01918 | 0.888223 |
| LGI1 | Homo sapiens leucine-rich, glioma inactivated 1 (LGI1), mRNA [NM_005097] | 1.44935 | 1.46849 | -0.01914 | 0.896588 |
| DYNLL2 | Homo sapiens dynein, light chain, LC8-type 2 (DYNLL2), mRNA [NM_080677] | 1.423342 | 1.442353 | -0.01901 | 0.888559 |
| DLX2 | Homo sapiens distal-less homeobox 2 (DLX2), mRNA [NM_004405] | 1.36698 | 1.385979 | -0.019 | 0.888223 |
| CAP2 | Homo sapiens CAP, adenylate cyclase-associated protein, 2 (yeast) (CAP2), mRNA [NM_006366] | 1.438515 | 1.45736 | -0.01884 | 0.888223 |
| GIMAP5 | Homo sapiens GTPase, IMAP family member 5 (GIMAP5), mRNA [NM_018384] | 1.350045 | 1.368574 | -0.01853 | 0.91885 |
| NR1H3 | Homo sapiens nuclear receptor subfamily 1, group H, member 3 (NR1H3), transcript variant 1, mRNA [NM_005693] | 1.446254 | 1.464587 | -0.01833 | 0.933545 |
| ACVR1 | Homo sapiens activin A receptor, type I (ACVR1), transcript variant 1, mRNA [NM_001105] | 1.366128 | 1.38443 | -0.0183 | 0.888223 |
| GDAP1L1 | Homo sapiens ganglioside-induced differentiation-associated protein 1-like 1 (GDAP1L1), mRNA [NM_024034] | 1.327881 | 1.345951 | -0.01807 | 0.888223 |
| APP | Homo sapiens amyloid beta (A4) precursor protein (APP), transcript variant 1, mRNA [NM_000484] | 1.361038 | 1.378727 | -0.01769 | 0.888223 |
| BTG4 | Homo sapiens B-cell translocation gene 4 (BTG4), mRNA [NM_017589] | 1.326687 | 1.344338 | -0.01765 | 0.888223 |
| RXRA | Homo sapiens retinoid X receptor, alpha (RXRA), mRNA [NM_002957] | 1.460407 | 1.478033 | -0.01763 | 0.888223 |
| FRS2 | Homo sapiens fibroblast growth factor receptor substrate 2 (FRS2), transcript variant 1, mRNA [NM_006654] | 1.406537 | 1.423979 | -0.01744 | 0.888223 |
| TAF8 | TAF8 RNA polymerase II, TATA box binding protein (TBP)-associated factor, 43kDa [Source:HGNC Symbol;Acc:17300] [ENST00000372978] | 1.324634 | 1.342061 | -0.01743 | 0.888223 |
| PTCHD3 | Homo sapiens patched domain containing 3 (PTCHD3), mRNA [NM_001034842] | 1.429892 | 1.447277 | -0.01739 | 0.888223 |
| BLM | Homo sapiens Bloom syndrome, RecQ helicase-like (BLM), mRNA [NM_000057] | 1.352891 | 1.36991 | -0.01702 | 0.888223 |
| MSI2 | Homo sapiens musashi homolog 2 (Drosophila) (MSI2), transcript variant 2, mRNA [NM_170721] | 2.40275 | 2.419751 | -0.017 | 0.993282 |
| RPS6KA5 | Homo sapiens ribosomal protein S6 kinase, 90kDa, polypeptide 5 (RPS6KA5), transcript variant 1, mRNA [NM_004755] | 3.459502 | 3.476414 | -0.01691 | 0.991676 |
| FANCC | Homo sapiens Fanconi anemia, complementation group C (FANCC), transcript variant 1, mRNA [NM_000136] | 1.348623 | 1.365259 | -0.01664 | 0.890271 |
| ABL1 | Homo sapiens c-abl oncogene 1, non-receptor tyrosine kinase (ABL1), transcript variant b, mRNA [NM_007313] | 1.359445 | 1.376009 | -0.01656 | 0.890271 |
| BMPR1A | Homo sapiens bone morphogenetic protein receptor, type IA (BMPR1A), mRNA [NM_004329] | 1.414998 | 1.431546 | -0.01655 | 0.888223 |
| SDC2 | Homo sapiens syndecan 2 (SDC2), mRNA [NM_002998] | 1.361815 | 1.378338 | -0.01652 | 0.888223 |
| NME2 | Homo sapiens non-metastatic cells 2, protein (NM23B) expressed in (NME2), transcript variant 1, mRNA [NM_002512] | 1.356913 | 1.373418 | -0.01651 | 0.888223 |
| DMRT1 | Homo sapiens doublesex and mab-3 related transcription factor 1 (DMRT1), mRNA [NM_021951] | 1.328345 | 1.344776 | -0.01643 | 0.888223 |
| KCNJ10 | Homo sapiens potassium inwardly-rectifying channel, subfamily J, member 10 (KCNJ10), mRNA [NM_002241] | 1.522144 | 1.538346 | -0.0162 | 0.941921 |
| ESR1 | Homo sapiens estrogen receptor 1 (ESR1), transcript variant 1, mRNA [NM_000125] | 1.389556 | 1.405754 | -0.0162 | 0.902599 |
| ENST00000455749 | neuropilin 1 [Source:HGNC Symbol;Acc:8004] [ENST00000374818] | 1.430629 | 1.446706 | -0.01608 | 0.924837 |
| JAM3 | Homo sapiens junctional adhesion molecule 3 (JAM3), transcript variant 1, mRNA [NM_032801] | 1.372666 | 1.388551 | -0.01589 | 0.894194 |
| MSX2 | Homo sapiens msh homeobox 2 (MSX2), mRNA [NM_002449] | 1.435656 | 1.451422 | -0.01577 | 0.909677 |
| HDAC9 | Homo sapiens histone deacetylase 9 (HDAC9), transcript variant 3, mRNA [NM_014707] | 1.406181 | 1.421923 | -0.01574 | 0.888223 |
| E2F4 | Homo sapiens E2F transcription factor 4, p107/p130-binding (E2F4), mRNA [NM_001950] | 1.3255 | 1.341217 | -0.01572 | 0.888223 |
| MSR1 | BROAD Institute lincRNA (XLOC_007013), lincRNA [TCONS_00014939] | 1.31617 | 1.331805 | -0.01563 | 0.888223 |
| SCN3B | Homo sapiens sodium channel, voltage-gated, type III, beta (SCN3B), transcript variant 1, mRNA [NM_018400] | 1.405468 | 1.421101 | -0.01563 | 0.913827 |
| FGF5 | Homo sapiens fibroblast growth factor 5 (FGF5), transcript variant 1, mRNA [NM_004464] | 1.334322 | 1.34994 | -0.01562 | 0.897441 |
| RTN1 | Homo sapiens reticulon 1 (RTN1), transcript variant 1, mRNA [NM_021136] | 1.403596 | 1.419141 | -0.01554 | 0.889049 |
| NRTN | Homo sapiens neurturin (NRTN), mRNA [NM_004558] | 1.353314 | 1.368703 | -0.01539 | 0.894891 |
| SPO11 | Homo sapiens SPO11 meiotic protein covalently bound to DSB homolog (S. cerevisiae) (SPO11), transcript variant 1, mRNA [NM_012444] | 1.317928 | 1.333078 | -0.01515 | 0.888223 |
| HNF1B | Homo sapiens HNF1 homeobox B (HNF1B), transcript variant 1, mRNA [NM_000458] | 2.923684 | 2.938819 | -0.01513 | 0.996578 |
| CUGBP1 | Homo sapiens CUGBP, Elav-like family member 1 (CELF1), transcript variant 2, mRNA [NM_198700] | 1.645278 | 1.660408 | -0.01513 | 0.991519 |
| KCNH1 | Homo sapiens potassium voltage-gated channel, subfamily H (eag-related), member 1 (KCNH1), transcript variant 1, mRNA [NM_172362] | 1.347981 | 1.363089 | -0.01511 | 0.901506 |
| ALDH1A2 | Homo sapiens aldehyde dehydrogenase 1 family, member A2 (ALDH1A2), transcript variant 3, mRNA [NM_170697] | 1.35301 | 1.368114 | -0.0151 | 0.897701 |
| HOXC8 | Homo sapiens homeobox C8 (HOXC8), mRNA [NM_022658] | 1.319223 | 1.334304 | -0.01508 | 0.888223 |
| RGMB | Homo sapiens RGM domain family, member B (RGMB), mRNA [NM_001012761] | 1.383763 | 1.398806 | -0.01504 | 0.897838 |
| MLPH | Homo sapiens melanophilin (MLPH), transcript variant 1, mRNA [NM_024101] | 1.59613 | 1.61116 | -0.01503 | 0.989698 |
| LPIN1 | Homo sapiens lipin 1 (LPIN1), mRNA [NM_145693] | 1.447871 | 1.462898 | -0.01503 | 0.888223 |
| ALS2 | Homo sapiens amyotrophic lateral sclerosis 2 (juvenile) (ALS2), transcript variant 2, mRNA [NM_001135745] | 1.505888 | 1.520836 | -0.01495 | 0.943111 |
| CHD7 | Homo sapiens chromodomain helicase DNA binding protein 7 (CHD7), mRNA [NM_017780] | 1.349639 | 1.364404 | -0.01476 | 0.888223 |
| ENST00000370496 | collagen, type IX, alpha 1 [Source:HGNC Symbol;Acc:2217] [ENST00000370496] | 1.366856 | 1.381582 | -0.01473 | 0.888223 |
| RHOH | Homo sapiens ras homolog gene family, member H (RHOH), mRNA [NM_004310] | 1.353748 | 1.368466 | -0.01472 | 0.912409 |
| FGFR3 | Homo sapiens fibroblast growth factor receptor 3 (FGFR3), transcript variant 1, mRNA [NM_000142] | 1.316664 | 1.33138 | -0.01472 | 0.888223 |
| DNER | Homo sapiens delta/notch-like EGF repeat containing (DNER), mRNA [NM_139072] | 1.322838 | 1.337494 | -0.01466 | 0.888223 |
| NEDD4 | Homo sapiens neural precursor cell expressed, developmentally down-regulated 4 (NEDD4), transcript variant 2, mRNA [NM_198400] | 2.016302 | 2.03089 | -0.01459 | 0.99395 |
| CCKAR | Homo sapiens cholecystokinin A receptor (CCKAR), mRNA [NM_000730] | 1.326863 | 1.341299 | -0.01444 | 0.892172 |
| LEF1 | Homo sapiens lymphoid enhancer-binding factor 1 (LEF1), transcript variant 1, mRNA [NM_016269] | 1.346834 | 1.361224 | -0.01439 | 0.904481 |
| ENST00000374441 | muscle, skeletal, receptor tyrosine kinase [Source:HGNC Symbol;Acc:7525] [ENST00000374441] | 1.379467 | 1.393817 | -0.01435 | 0.902607 |
| SOCS5 | Homo sapiens suppressor of cytokine signaling 5 (SOCS5), transcript variant 2, mRNA [NM_144949] | 1.387012 | 1.401304 | -0.01429 | 0.914628 |
| SOX2 | Homo sapiens SRY (sex determining region Y)-box 2 (SOX2), mRNA [NM_003106] | 1.376348 | 1.390515 | -0.01417 | 0.912315 |
| QKI | Homo sapiens QKI, KH domain containing, RNA binding (QKI), transcript variant 1, mRNA [NM_006775] | 1.32575 | 1.339839 | -0.01409 | 0.908164 |
| ABL2 | Homo sapiens v-abl Abelson murine leukemia viral oncogene homolog 2 (ABL2), transcript variant b, mRNA [NM_007314] | 1.363086 | 1.377166 | -0.01408 | 0.909677 |
| C5orf13 | Homo sapiens chromosome 5 open reading frame 13 (C5orf13), transcript variant 11, mRNA [NM_001142483] | 1.33931 | 1.353273 | -0.01396 | 0.888223 |
| CD80 | Homo sapiens CD80 molecule (CD80), mRNA [NM_005191] | 1.446872 | 1.460829 | -0.01396 | 0.889199 |
| TBR1 | Homo sapiens T-box, brain, 1 (TBR1), mRNA [NM_006593] | 1.426955 | 1.440879 | -0.01392 | 0.911258 |
| IL15RA | Homo sapiens interleukin 15 receptor, alpha (IL15RA), transcript variant 2, mRNA [NM_172200] | 1.474769 | 1.488599 | -0.01383 | 0.915089 |
| BNIP2 | BCL2/adenovirus E1B 19kDa interacting protein 2 [Source:HGNC Symbol;Acc:1083] [ENST00000267859] | 1.351895 | 1.365624 | -0.01373 | 0.888223 |
| CACNA1I | Homo sapiens calcium channel, voltage-dependent, T type, alpha 1I subunit (CACNA1I), transcript variant 1, mRNA [NM_021096] | 1.31926 | 1.332876 | -0.01362 | 0.888223 |
| GPR183 | Homo sapiens G protein-coupled receptor 183 (GPR183), mRNA [NM_004951] | 1.399399 | 1.412898 | -0.0135 | 0.910559 |
| MARK2 | Homo sapiens MAP/microtubule affinity-regulating kinase 2 (MARK2), transcript variant 3, mRNA [NM_004954] | 1.367108 | 1.380485 | -0.01338 | 0.912316 |
| EID3 | Homo sapiens EP300 interacting inhibitor of differentiation 3 (EID3), mRNA [NM_001008394] | 1.322508 | 1.335859 | -0.01335 | 0.888223 |
| ROCK2 | Homo sapiens Rho-associated, coiled-coil containing protein kinase 2 (ROCK2), mRNA [NM_004850] | 1.355488 | 1.368784 | -0.0133 | 0.912409 |
| UCHL1 | Homo sapiens ubiquitin carboxyl-terminal esterase L1 (ubiquitin thiolesterase) (UCHL1), mRNA [NM_004181] | 1.294644 | 1.307844 | -0.0132 | 0.891623 |
| TFRC | Homo sapiens transferrin receptor (p90, CD71) (TFRC), transcript variant 1, mRNA [NM_003234] | 1.393868 | 1.40704 | -0.01317 | 0.888223 |
| NKAP | Homo sapiens NFKB activating protein (NKAP), mRNA [NM_024528] | 1.711182 | 1.724327 | -0.01314 | 0.993031 |
| FOXN4 | Homo sapiens forkhead box N4 (FOXN4), mRNA [NM_213596] | 1.373175 | 1.386232 | -0.01306 | 0.917497 |
| C22orf28 | Homo sapiens chromosome 22 open reading frame 28 (C22orf28), mRNA [NM_014306] | 1.331178 | 1.344198 | -0.01302 | 0.889696 |
| SLC25A38 | Homo sapiens solute carrier family 25, member 38 (SLC25A38), nuclear gene encoding mitochondrial protein, mRNA [NM_017875] | 1.407672 | 1.42067 | -0.013 | 0.911311 |
| ITGB1 | Homo sapiens integrin, beta 1 (fibronectin receptor, beta polypeptide, antigen CD29 includes MDF2, MSK12) (ITGB1), transcript variant 1E, mRNA [NM_133376] | 1.455097 | 1.468092 | -0.01299 | 0.980205 |
| DFNA5 | Homo sapiens deafness, autosomal dominant 5 (DFNA5), transcript variant 1, mRNA [NM_004403] | 1.354113 | 1.367044 | -0.01293 | 0.888223 |
| ALS2 | Homo sapiens amyotrophic lateral sclerosis 2 (juvenile) (ALS2), transcript variant 1, mRNA [NM_020919] | 1.525397 | 1.538318 | -0.01292 | 0.952469 |
| ENST00000396039 | par-6 partitioning defective 6 homolog beta (C. elegans) [Source:HGNC Symbol;Acc:16245] [ENST00000396039] | 1.380512 | 1.393267 | -0.01275 | 0.923908 |
| TFE3 | Homo sapiens transcription factor binding to IGHM enhancer 3 (TFE3), mRNA [NM_006521] | 1.315836 | 1.328513 | -0.01268 | 0.888223 |
| CTSL2 | Homo sapiens cathepsin L2 (CTSL2), transcript variant 1, mRNA [NM_001333] | 1.430361 | 1.443021 | -0.01266 | 0.988184 |
| FOXC2 | Homo sapiens forkhead box C2 (MFH-1, mesenchyme forkhead 1) (FOXC2), mRNA [NM_005251] | 1.407179 | 1.419805 | -0.01263 | 0.93567 |
| CUX1 | Homo sapiens cut-like homeobox 1 (CUX1), transcript variant 2, mRNA [NM_001913] | 1.479481 | 1.492106 | -0.01263 | 0.896841 |
| TTPA | Homo sapiens tocopherol (alpha) transfer protein (TTPA), mRNA [NM_000370] | 1.381571 | 1.393982 | -0.01241 | 0.927386 |
| NEUROD6 | Homo sapiens neurogenic differentiation 6 (NEUROD6), mRNA [NM_022728] | 1.346268 | 1.358644 | -0.01238 | 0.918781 |
| PDZRN3 | Homo sapiens PDZ domain containing ring finger 3 (PDZRN3), mRNA [NM_015009] | 1.390509 | 1.402831 | -0.01232 | 0.932009 |
| NRAS | Homo sapiens neuroblastoma RAS viral (v-ras) oncogene homolog (NRAS), mRNA [NM_002524] | 1.358278 | 1.370506 | -0.01223 | 0.920732 |
| A_19_P00805548 | Homo sapiens bone morphogenetic protein receptor, type IA (BMPR1A), mRNA [NM_004329] | 1.336424 | 1.348606 | -0.01218 | 0.924895 |
| IL31RA | Homo sapiens interleukin 31 receptor A (IL31RA), transcript variant 4, mRNA [NM_001242638] | 1.33799 | 1.350053 | -0.01206 | 0.917204 |
| FOXD2 | Homo sapiens forkhead box D2 (FOXD2), mRNA [NM_004474] | 1.371206 | 1.383135 | -0.01193 | 0.888223 |
| EHF | Homo sapiens ets homologous factor (EHF), transcript variant 2, mRNA [NM_012153] | 1.312881 | 1.324767 | -0.01189 | 0.888223 |
| FEZF2 | Homo sapiens FEZ family zinc finger 2 (FEZF2), mRNA [NM_018008] | 1.328354 | 1.340235 | -0.01188 | 0.908164 |
| PAK2 | Homo sapiens p21 protein (Cdc42/Rac)-activated kinase 2 (PAK2), mRNA [NM_002577] | 1.401746 | 1.413577 | -0.01183 | 0.939445 |
| CDK5RAP2 | Homo sapiens CDK5 regulatory subunit associated protein 2 (CDK5RAP2), transcript variant 1, mRNA [NM_018249] | 1.356698 | 1.368465 | -0.01177 | 0.888223 |
| CDK5RAP1 | Homo sapiens CDK5 regulatory subunit associated protein 1 (CDK5RAP1), transcript variant 2, mRNA [NM_016082] | 1.418025 | 1.429745 | -0.01172 | 0.933381 |
| HOOK3 | Homo sapiens hook homolog 3 (Drosophila) (HOOK3), mRNA [NM_032410] | 1.419192 | 1.430891 | -0.0117 | 0.936398 |
| RC3H1 | Homo sapiens ring finger and CCCH-type domains 1 (RC3H1), mRNA [NM_172071] | 1.32165 | 1.333217 | -0.01157 | 0.897441 |
| RTN4 | Homo sapiens reticulon 4 (RTN4), transcript variant 1, mRNA [NM_020532] | 1.321352 | 1.332703 | -0.01135 | 0.898834 |
| WNT16 | Homo sapiens wingless-type MMTV integration site family, member 16 (WNT16), transcript variant 1, mRNA [NM_057168] | 1.417831 | 1.429077 | -0.01125 | 0.933545 |
| HHEX | Homo sapiens hematopoietically expressed homeobox (HHEX), mRNA [NM_002729] | 1.410722 | 1.421844 | -0.01112 | 0.946291 |
| FGF20 | Homo sapiens fibroblast growth factor 20 (FGF20), mRNA [NM_019851] | 1.377448 | 1.388537 | -0.01109 | 0.92693 |
| SOX21 | Homo sapiens SRY (sex determining region Y)-box 21 (SOX21), mRNA [NM_007084] | 1.317203 | 1.328288 | -0.01108 | 0.914567 |
| SSH2 | Homo sapiens slingshot homolog 2 (Drosophila) (SSH2), mRNA [NM_033389] | 1.359685 | 1.37067 | -0.01099 | 0.89938 |
| RQCD1 | Homo sapiens RCD1 required for cell differentiation1 homolog (S. pombe) (RQCD1), mRNA [NM_005444] | 1.306613 | 1.317584 | -0.01097 | 0.888223 |
| ALK | Homo sapiens anaplastic lymphoma receptor tyrosine kinase (ALK), mRNA [NM_004304] | 1.382795 | 1.393733 | -0.01094 | 0.938226 |
| SPI1 | Homo sapiens spleen focus forming virus (SFFV) proviral integration oncogene spi1 (SPI1), transcript variant 1, mRNA [NM_001080547] | 1.399989 | 1.410912 | -0.01092 | 0.944559 |
| MREG | Homo sapiens melanoregulin (MREG), mRNA [NM_018000] | 1.317692 | 1.328602 | -0.01091 | 0.92103 |
| UBE4B | Homo sapiens ubiquitination factor E4B (UBE4B), transcript variant 2, mRNA [NM_006048] | 1.345637 | 1.356501 | -0.01086 | 0.908164 |
| ID2 | Homo sapiens inhibitor of DNA binding 2, dominant negative helix-loop-helix protein (ID2), mRNA [NM_002166] | 1.375671 | 1.386486 | -0.01081 | 0.888223 |
| MSH2 | Homo sapiens mutS homolog 2, colon cancer, nonpolyposis type 1 (E. coli) (MSH2), mRNA [NM_000251] | 1.364069 | 1.374789 | -0.01072 | 0.888223 |
| CCM2 | Homo sapiens cerebral cavernous malformation 2 (CCM2), transcript variant 2, mRNA [NM_031443] | 1.365562 | 1.376185 | -0.01062 | 0.933216 |
| NTNG1 | Homo sapiens netrin G1 (NTNG1), transcript variant 3, mRNA [NM_014917] | 1.393819 | 1.404441 | -0.01062 | 0.896801 |
| PAK1 | Homo sapiens p21 protein (Cdc42/Rac)-activated kinase 1 (PAK1), transcript variant 1, mRNA [NM_001128620] | 1.454344 | 1.464801 | -0.01046 | 0.916588 |
| CASC5 | Homo sapiens cancer susceptibility candidate 5 (CASC5), transcript variant 1, mRNA [NM_170589] | 1.386476 | 1.396899 | -0.01042 | 0.928887 |
| ERBB4 | Homo sapiens v-erb-a erythroblastic leukemia viral oncogene homolog 4 (avian) (ERBB4), transcript variant JM-a/CVT-1, mRNA [NM_005235] | 1.451665 | 1.462059 | -0.01039 | 0.908164 |
| FGF1 | Homo sapiens fibroblast growth factor 1 (acidic) (FGF1), transcript variant 1, mRNA [NM_000800] | 1.341032 | 1.351376 | -0.01034 | 0.949585 |
| ATL1 | Homo sapiens atlastin GTPase 1 (ATL1), transcript variant 2, mRNA [NM_181598] | 1.426346 | 1.436688 | -0.01034 | 0.900585 |
| EIF2B3 | Homo sapiens eukaryotic translation initiation factor 2B, subunit 3 gamma, 58kDa (EIF2B3), transcript variant 1, mRNA [NM_020365] | 1.421869 | 1.432156 | -0.01029 | 0.947138 |
| PPARD | Homo sapiens peroxisome proliferator-activated receptor delta (PPARD), transcript variant 1, mRNA [NM_006238] | 1.456846 | 1.467129 | -0.01028 | 0.895437 |
| RGS14 | Homo sapiens regulator of G-protein signaling 14 (RGS14), mRNA [NM_006480] | 1.422833 | 1.433056 | -0.01022 | 0.943317 |
| SLC2A4 | Homo sapiens solute carrier family 2 (facilitated glucose transporter), member 4 (SLC2A4), mRNA [NM_001042] | 1.358812 | 1.368825 | -0.01001 | 0.934467 |
| SIAH1 | Homo sapiens seven in absentia homolog 1 (Drosophila) (SIAH1), transcript variant 1, mRNA [NM_003031] | 1.394542 | 1.404542 | -0.01 | 0.943621 |
| ETV4 | Homo sapiens ets variant 4 (ETV4), transcript variant 2, mRNA [NM_001079675] | 1.372749 | 1.382732 | -0.00998 | 0.888223 |
| WNK1 | Homo sapiens WNK lysine deficient protein kinase 1 (WNK1), transcript variant 4, mRNA [NM_001184985] | 1.323336 | 1.3333 | -0.00996 | 0.892771 |
| TWIST1 | Homo sapiens twist homolog 1 (Drosophila) (TWIST1), mRNA [NM_000474] | 2.596919 | 2.606743 | -0.00982 | 0.998132 |
| DGKG | Homo sapiens diacylglycerol kinase, gamma 90kDa (DGKG), transcript variant 1, mRNA [NM_001346] | 1.393292 | 1.403082 | -0.00979 | 0.923413 |
| FEM1B | Homo sapiens fem-1 homolog b (C. elegans) (FEM1B), mRNA [NM_015322] | 1.304173 | 1.313871 | -0.0097 | 0.888223 |
| RHOG | Homo sapiens ras homolog gene family, member G (rho G) (RHOG), mRNA [NM_001665] | 1.398414 | 1.408092 | -0.00968 | 0.951804 |
| TET2 | Homo sapiens tet methylcytosine dioxygenase 2 (TET2), transcript variant 1, mRNA [NM_001127208] | 1.394943 | 1.404586 | -0.00964 | 0.951646 |
| ROBO1 | Homo sapiens roundabout, axon guidance receptor, homolog 1 (Drosophila) (ROBO1), transcript variant 2, mRNA [NM_133631] | 1.49889 | 1.508497 | -0.00961 | 0.9423 |
| HMGCS1 | Homo sapiens 3-hydroxy-3-methylglutaryl-CoA synthase 1 (soluble) (HMGCS1), transcript variant 2, mRNA [NM_002130] | 1.373007 | 1.382513 | -0.00951 | 0.910412 |
| PTH1R | Homo sapiens parathyroid hormone 1 receptor (PTH1R), transcript variant 1, mRNA [NM_000316] | 1.362965 | 1.372333 | -0.00937 | 0.909665 |
| REC8 | Homo sapiens REC8 homolog (yeast) (REC8), transcript variant 2, mRNA [NM_001048205] | 1.477329 | 1.48664 | -0.00931 | 0.902241 |
| ENST00000409550 | catenin (cadherin-associated protein), alpha 2 [Source:HGNC Symbol;Acc:2510] [ENST00000409550] | 1.392889 | 1.402115 | -0.00923 | 0.953378 |
| CDK5RAP3 | Homo sapiens CDK5 regulatory subunit associated protein 3 (CDK5RAP3), mRNA [NM_176096] | 1.321357 | 1.3305 | -0.00914 | 0.9423 |
| KEAP1 | Homo sapiens kelch-like ECH-associated protein 1 (KEAP1), transcript variant 1, mRNA [NM_203500] | 1.434944 | 1.444065 | -0.00912 | 0.979887 |
| ACVRL1 | Homo sapiens activin A receptor type II-like 1 (ACVRL1), transcript variant 1, mRNA [NM_000020] | 1.399926 | 1.409041 | -0.00911 | 0.92344 |
| TRIB2 | Homo sapiens tribbles homolog 2 (Drosophila) (TRIB2), transcript variant 1, mRNA [NM_021643] | 1.355222 | 1.364334 | -0.00911 | 0.888223 |
| LRTOMT | Homo sapiens leucine rich transmembrane and 0-methyltransferase domain containing (LRTOMT), transcript variant 1, mRNA [NM_145309] | 1.313235 | 1.322236 | -0.009 | 0.927485 |
| PTPRJ | Homo sapiens protein tyrosine phosphatase, receptor type, J (PTPRJ), transcript variant 1, mRNA [NM_002843] | 1.452113 | 1.461101 | -0.00899 | 0.932178 |
| RPL22 | Homo sapiens ribosomal protein L22 (RPL22), mRNA [NM_000983] | 1.321645 | 1.330603 | -0.00896 | 0.904649 |
| GPX1 | Homo sapiens glutathione peroxidase 1 (GPX1), transcript variant 2, mRNA [NM_201397] | 1.490704 | 1.499587 | -0.00888 | 0.912552 |
| GNAS | Homo sapiens GNAS complex locus (GNAS), transcript variant 4, mRNA [NM_016592] | 1.462893 | 1.471752 | -0.00886 | 0.960363 |
| JAG1 | Homo sapiens jagged 1 (JAG1), mRNA [NM_000214] | 1.351958 | 1.360604 | -0.00865 | 0.945624 |
| PKNOX1 | Homo sapiens PBX/knotted 1 homeobox 1 (PKNOX1), mRNA [NM_004571] | 1.313296 | 1.321922 | -0.00863 | 0.933681 |
| TLX3 | Homo sapiens T-cell leukemia homeobox 3 (TLX3), mRNA [NM_021025] | 1.394658 | 1.403206 | -0.00855 | 0.958173 |
| YBX1 | Homo sapiens Y box binding protein 1 (YBX1), mRNA [NM_004559] | 1.423835 | 1.432317 | -0.00848 | 0.942389 |
| DLX1 | distal-less homeobox 1 [Source:HGNC Symbol;Acc:2914] [ENST00000409492] | 1.31659 | 1.325023 | -0.00843 | 0.912409 |
| CNTN1 | Homo sapiens contactin 1 (CNTN1), transcript variant 1, mRNA [NM_001843] | 1.438476 | 1.446879 | -0.0084 | 0.960515 |
| EFNB3 | Homo sapiens ephrin-B3 (EFNB3), mRNA [NM_001406] | 1.31757 | 1.325966 | -0.0084 | 0.912409 |
| IL1A | Homo sapiens interleukin 1, alpha (IL1A), mRNA [NM_000575] | 1.382657 | 1.390983 | -0.00833 | 0.888223 |
| RPS6KA3 | Homo sapiens ribosomal protein S6 kinase, 90kDa, polypeptide 3 (RPS6KA3), mRNA [NM_004586] | 1.293168 | 1.301441 | -0.00827 | 0.89938 |
| A_19_P00322910 | Homo sapiens twist homolog 2 (Drosophila) (TWIST2), mRNA [NM_057179] | 1.341374 | 1.349629 | -0.00825 | 0.959883 |
| DLG1 | Homo sapiens discs, large homolog 1 (Drosophila) (DLG1), transcript variant 2, mRNA [NM_004087] | 1.395647 | 1.403787 | -0.00814 | 0.929687 |
| ENST00000398133 | H.sapiens alpha-A crystallin gene exon 1,2 and pseudoexon. [X14789] | 1.329247 | 1.337151 | -0.0079 | 0.954043 |
| BHLHE22 | Homo sapiens basic helix-loop-helix family, member e22 (BHLHE22), mRNA [NM_152414] | 1.394507 | 1.402406 | -0.0079 | 0.928887 |
| SPAG9 | Homo sapiens sperm associated antigen 9 (SPAG9), transcript variant 3, mRNA [NM_003971] | 1.384822 | 1.392691 | -0.00787 | 0.959231 |
| PPP3CB | protein phosphatase 3, catalytic subunit, beta isozyme [Source:HGNC Symbol;Acc:9315] [ENST00000394822] | 1.355919 | 1.363697 | -0.00778 | 0.951646 |
| LAMA1 | Homo sapiens laminin, alpha 1 (LAMA1), mRNA [NM_005559] | 1.298571 | 1.306337 | -0.00777 | 0.892771 |
| SUZ12 | Homo sapiens suppressor of zeste 12 homolog (Drosophila) (SUZ12), mRNA [NM_015355] | 1.34355 | 1.35128 | -0.00773 | 0.888223 |
| DNMT3B | Homo sapiens DNA (cytosine-5-)-methyltransferase 3 beta (DNMT3B), transcript variant 6, mRNA [NM_175850] | 1.40397 | 1.411587 | -0.00762 | 0.964566 |
| SEMA6A | Homo sapiens sema domain, transmembrane domain (TM), and cytoplasmic domain, (semaphorin) 6A (SEMA6A), mRNA [NM_020796] | 1.464645 | 1.472169 | -0.00752 | 0.889741 |
| ANTXR1 | Homo sapiens anthrax toxin receptor 1 (ANTXR1), transcript variant 2, mRNA [NM_053034] | 2.784118 | 2.79162 | -0.0075 | 0.998495 |
| NME1 | Homo sapiens non-metastatic cells 1, protein (NM23A) expressed in (NME1), transcript variant 1, mRNA [NM_198175] | 1.405197 | 1.412649 | -0.00745 | 0.945204 |
| PAX7 | Homo sapiens paired box 7 (PAX7), transcript variant 3, mRNA [NM_001135254] | 1.347575 | 1.354991 | -0.00742 | 0.888223 |
| BAIAP2 | Homo sapiens BAI1-associated protein 2 (BAIAP2), transcript variant 1, mRNA [NM_017450] | 1.425213 | 1.432621 | -0.00741 | 0.963794 |
| ZNF536 | Homo sapiens zinc finger protein 536 (ZNF536), mRNA [NM_014717] | 1.348397 | 1.355518 | -0.00712 | 0.954723 |
| SFXN1 | Homo sapiens sideroflexin 1 (SFXN1), mRNA [NM_022754] | 1.489853 | 1.496862 | -0.00701 | 0.956522 |
| WNT1 | Homo sapiens wingless-type MMTV integration site family, member 1 (WNT1), mRNA [NM_005430] | 1.363038 | 1.370039 | -0.007 | 0.927468 |
| WNT7B | Homo sapiens wingless-type MMTV integration site family, member 7B (WNT7B), mRNA [NM_058238] | 1.328822 | 1.335642 | -0.00682 | 0.922215 |
| HOMER1 | Homo sapiens homer homolog 1 (Drosophila) (HOMER1), mRNA [NM_004272] | 1.313217 | 1.320011 | -0.00679 | 0.923008 |
| SOX11 | Homo sapiens SRY (sex determining region Y)-box 11 (SOX11), mRNA [NM_003108] | 1.420058 | 1.426802 | -0.00674 | 0.971973 |
| S100B | Homo sapiens S100 calcium binding protein B (S100B), mRNA [NM_006272] | 1.406919 | 1.413662 | -0.00674 | 0.959796 |
| A_19_P00804767 | Homo sapiens bone morphogenetic protein receptor, type IA (BMPR1A), mRNA [NM_004329] | 1.332009 | 1.338699 | -0.00669 | 0.943365 |
| UNC5D | Homo sapiens unc-5 homolog D (C. elegans) (UNC5D), mRNA [NM_080872] | 1.432377 | 1.43898 | -0.0066 | 0.957155 |
| IGFBP5 | Homo sapiens insulin-like growth factor binding protein 5 (IGFBP5), mRNA [NM_000599] | 1.3251 | 1.331663 | -0.00656 | 0.960822 |
| HEYL | Homo sapiens hairy/enhancer-of-split related with YRPW motif-like (HEYL), mRNA [NM_014571] | 1.422268 | 1.428714 | -0.00645 | 0.960822 |
| RBMY1B | Homo sapiens RNA binding motif protein, Y-linked, family 1, member B (RBMY1B), mRNA [NM_001006121] | 1.313274 | 1.319718 | -0.00644 | 0.922171 |
| SALL1 | Homo sapiens sal-like 1 (Drosophila) (SALL1), transcript variant 1, mRNA [NM_002968] | 1.417678 | 1.424033 | -0.00635 | 0.966948 |
| CDK1 | Homo sapiens cyclin-dependent kinase 1 (CDK1), transcript variant 4, mRNA [NM_001170406] | 1.33398 | 1.340319 | -0.00634 | 0.913505 |
| SEMA3E | Homo sapiens sema domain, immunoglobulin domain (Ig), short basic domain, secreted, (semaphorin) 3E (SEMA3E), transcript variant 1, mRNA [NM_012431] | 1.387058 | 1.39331 | -0.00625 | 0.965365 |
| SCUBE1 | Homo sapiens signal peptide, CUB domain, EGF-like 1 (SCUBE1), mRNA [NM_173050] | 1.454139 | 1.460363 | -0.00622 | 0.946563 |
| PPP3CB | Homo sapiens protein phosphatase 3, catalytic subunit, beta isozyme (PPP3CB), transcript variant 2, mRNA [NM_021132] | 1.436458 | 1.442673 | -0.00622 | 0.956328 |
| ID1 | Homo sapiens inhibitor of DNA binding 1, dominant negative helix-loop-helix protein (ID1), transcript variant 1, mRNA [NM_002165] | 1.308454 | 1.314608 | -0.00615 | 0.951804 |
| AGT | Homo sapiens angiotensinogen (serpin peptidase inhibitor, clade A, member 8) (AGT), mRNA [NM_000029] | 1.404395 | 1.410507 | -0.00611 | 0.944435 |
| DOK7 | Homo sapiens docking protein 7 (DOK7), transcript variant 1, mRNA [NM_173660] | 1.331834 | 1.337807 | -0.00597 | 0.916669 |
| SPAG6 | Homo sapiens sperm associated antigen 6 (SPAG6), transcript variant 2, mRNA [NM_172242] | 1.39733 | 1.403016 | -0.00569 | 0.961061 |
| ABL1 | Homo sapiens c-abl oncogene 1, non-receptor tyrosine kinase (ABL1), transcript variant a, mRNA [NM_005157] | 1.432387 | 1.438003 | -0.00562 | 0.943542 |
| ZNF358 | Homo sapiens zinc finger protein 358 (ZNF358), mRNA [NM_018083] | 2.358692 | 2.364247 | -0.00555 | 0.998856 |
| CACNB2 | Homo sapiens calcium channel, voltage-dependent, beta 2 subunit (CACNB2), transcript variant 1, mRNA [NM_000724] | 1.374311 | 1.379779 | -0.00547 | 0.972357 |
| A_21_P0003042 | Q6T424_HUMAN (Q6T424) Tumor differentiation factor, partial (13%) [THC2606489] | 1.342577 | 1.347944 | -0.00537 | 0.921098 |
| TCEA1 | Homo sapiens transcription elongation factor A (SII), 1 (TCEA1), transcript variant 1, mRNA [NM_006756] | 1.319377 | 1.324424 | -0.00505 | 0.930477 |
| AFF4 | Homo sapiens AF4/FMR2 family, member 4 (AFF4), mRNA [NM_014423] | 1.381644 | 1.386631 | -0.00499 | 0.94934 |
| CACNB4 | Homo sapiens calcium channel, voltage-dependent, beta 4 subunit (CACNB4), transcript variant 1, mRNA [NM_001005747] | 1.374252 | 1.379201 | -0.00495 | 0.951513 |
| ENST00000344201 | core-binding factor, runt domain, alpha subunit 2; translocated to, 2 [Source:HGNC Symbol;Acc:1536] [ENST00000397798] | 1.314856 | 1.319805 | -0.00495 | 0.939445 |
| CGA | Homo sapiens glycoprotein hormones, alpha polypeptide (CGA), transcript variant 2, mRNA [NM_000735] | 1.447707 | 1.452653 | -0.00495 | 0.964674 |
| DMD | Homo sapiens dystrophin, mRNA (cDNA clone IMAGE:5274415), complete cds. [BC036103] | 1.381948 | 1.386831 | -0.00488 | 0.973936 |
| NCAM1 | Homo sapiens neural cell adhesion molecule 1 (NCAM1), transcript variant 4, mRNA [NM_001242608] | 1.421578 | 1.426455 | -0.00488 | 0.943111 |
| ZNF703 | Homo sapiens zinc finger protein 703 (ZNF703), mRNA [NM_025069] | 1.508615 | 1.513448 | -0.00483 | 0.974502 |
| NCOA4 | Homo sapiens nuclear receptor coactivator 4 (NCOA4), transcript variant 1, mRNA [NM_001145260] | 1.293873 | 1.298699 | -0.00483 | 0.913011 |
| HRNR | Homo sapiens hornerin (HRNR), mRNA [NM_001009931] | 1.35707 | 1.361889 | -0.00482 | 0.971474 |
| PICALM | Homo sapiens phosphatidylinositol binding clathrin assembly protein (PICALM), transcript variant 1, mRNA [NM_007166] | 1.423381 | 1.428188 | -0.00481 | 0.979568 |
| ROD1 | Homo sapiens ROD1 regulator of differentiation 1 (S. pombe) (ROD1), transcript variant 5, mRNA [NM_001244897] | 1.405722 | 1.410493 | -0.00477 | 0.958232 |
| ACTA1 | Homo sapiens actin, alpha 1, skeletal muscle (ACTA1), mRNA [NM_001100] | 1.374234 | 1.378948 | -0.00471 | 0.939769 |
| FZD1 | Homo sapiens frizzled family receptor 1 (FZD1), mRNA [NM_003505] | 1.292756 | 1.297455 | -0.0047 | 0.916701 |
| LAMA4 | Homo sapiens laminin, alpha 4 (LAMA4), transcript variant 5, mRNA [NM_001105209] | 1.424137 | 1.42883 | -0.00469 | 0.979887 |
| CD1D | Homo sapiens CD1d molecule (CD1D), mRNA [NM_001766] | 1.371649 | 1.376329 | -0.00468 | 0.94749 |
| OSTM1 | Homo sapiens osteopetrosis associated transmembrane protein 1 (OSTM1), mRNA [NM_014028] | 1.499399 | 1.504075 | -0.00468 | 0.972274 |
| EXOC4 | Homo sapiens exocyst complex component 4 (EXOC4), transcript variant 2, mRNA [NM_001037126] | 1.371419 | 1.376007 | -0.00459 | 0.951359 |
| ANG | Homo sapiens angiogenin, ribonuclease, RNase A family, 5 (ANG), transcript variant 1, mRNA [NM_001145] | 1.307131 | 1.311683 | -0.00455 | 0.924783 |
| RALA | Homo sapiens v-ral simian leukemia viral oncogene homolog A (ras related) (RALA), mRNA [NM_005402] | 1.505314 | 1.509846 | -0.00453 | 0.970992 |
| SPTB | Homo sapiens spectrin, beta, erythrocytic (SPTB), transcript variant 2, mRNA [NM_000347] | 1.484944 | 1.489468 | -0.00452 | 0.95204 |
| PTK2 | Homo sapiens PTK2 protein tyrosine kinase 2 (PTK2), transcript variant 1, mRNA [NM_153831] | 1.429664 | 1.433919 | -0.00425 | 0.982853 |
| FGF2 | Homo sapiens fibroblast growth factor 2 (basic) (FGF2), mRNA [NM_002006] | 1.305195 | 1.3094 | -0.00421 | 0.928543 |
| ABCA5 | Homo sapiens ATP-binding cassette, sub-family A (ABC1), member 5 (ABCA5), transcript variant 1, mRNA [NM_018672] | 1.416745 | 1.420927 | -0.00418 | 0.949119 |
| GAPDHS | Homo sapiens glyceraldehyde-3-phosphate dehydrogenase, spermatogenic (GAPDHS), mRNA [NM_014364] | 1.456269 | 1.460447 | -0.00418 | 0.958128 |
| DLX5 | Homo sapiens distal-less homeobox 5 (DLX5), mRNA [NM_005221] | 1.29778 | 1.301951 | -0.00417 | 0.931869 |
| WEE1 | Homo sapiens WEE1 homolog (S. pombe) (WEE1), transcript variant 1, mRNA [NM_003390] | 1.423095 | 1.427251 | -0.00416 | 0.981032 |
| DOCK1 | Homo sapiens dedicator of cytokinesis 1 (DOCK1), mRNA [NM_001380] | 1.411457 | 1.41561 | -0.00415 | 0.96515 |
| SP3 | Homo sapiens Sp3 transcription factor (SP3), transcript variant 1, mRNA [NM_003111] | 1.31842 | 1.322542 | -0.00412 | 0.951583 |
| SRD5A1 | Homo sapiens steroid-5-alpha-reductase, alpha polypeptide 1 (3-oxo-5 alpha-steroid delta 4-dehydrogenase alpha 1) (SRD5A1), mRNA [NM_001047] | 1.396812 | 1.400844 | -0.00403 | 0.974872 |
| INSR | Homo sapiens insulin receptor (INSR), transcript variant 1, mRNA [NM_000208] | 1.451015 | 1.455045 | -0.00403 | 0.972852 |
| PRMT1 | Homo sapiens protein arginine methyltransferase 1 (PRMT1), transcript variant 1, mRNA [NM_001536] | 1.342606 | 1.34655 | -0.00394 | 0.972274 |
| DSCAM | Homo sapiens Down syndrome cell adhesion molecule (DSCAM), transcript variant 1, mRNA [NM_001389] | 1.485257 | 1.48918 | -0.00392 | 0.96199 |
| PDE4D | Homo sapiens phosphodiesterase 4D, cAMP-specific (PDE4D), transcript variant 3, mRNA [NM_001165899] | 1.327165 | 1.331073 | -0.00391 | 0.888223 |
| OTX2 | Homo sapiens orthodenticle homeobox 2 (OTX2), transcript variant 1, mRNA [NM_021728] | 1.50645 | 1.510289 | -0.00384 | 0.976755 |
| PTBP1 | Homo sapiens polypyrimidine tract binding protein 1 (PTBP1), transcript variant 1, mRNA [NM_002819] | 1.391294 | 1.395071 | -0.00378 | 0.980555 |
| CDK6 | Homo sapiens cyclin-dependent kinase 6 (CDK6), transcript variant 1, mRNA [NM_001259] | 1.348338 | 1.352075 | -0.00374 | 0.959324 |
| GDF5 | Homo sapiens growth differentiation factor 5 (GDF5), mRNA [NM_000557] | 1.335096 | 1.338603 | -0.00351 | 0.969442 |
| HCLS1 | Homo sapiens hematopoietic cell-specific Lyn substrate 1 (HCLS1), mRNA [NM_005335] | 1.424152 | 1.42758 | -0.00343 | 0.956364 |
| ATXN10 | Homo sapiens ataxin 10 (ATXN10), transcript variant 1, mRNA [NM_013236] | 1.338341 | 1.341709 | -0.00337 | 0.979652 |
| UPK1B | Homo sapiens uroplakin 1B (UPK1B), mRNA [NM_006952] | 1.432353 | 1.435695 | -0.00334 | 0.986069 |
| KLF2 | Homo sapiens Kruppel-like factor 2 (lung) (KLF2), mRNA [NM_016270] | 1.404265 | 1.407446 | -0.00318 | 0.987798 |
| RFX6 | Homo sapiens regulatory factor X, 6 (RFX6), mRNA [NM_173560] | 1.307301 | 1.310477 | -0.00318 | 0.955793 |
| ATF1 | Homo sapiens activating transcription factor 1 (ATF1), mRNA [NM_005171] | 1.436146 | 1.439311 | -0.00316 | 0.972274 |
| PRMT2 | Homo sapiens protein arginine methyltransferase 2 (PRMT2), transcript variant 1, mRNA [NM_206962] | 1.298623 | 1.301765 | -0.00314 | 0.969347 |
| MNX1 | Homo sapiens motor neuron and pancreas homeobox 1 (MNX1), transcript variant 1, mRNA [NM_005515] | 1.398051 | 1.401148 | -0.0031 | 0.98249 |
| LCE2B | Homo sapiens late cornified envelope 2B (LCE2B), mRNA [NM_014357] | 1.332139 | 1.335104 | -0.00297 | 0.981249 |
| AKT2 | Homo sapiens v-akt murine thymoma viral oncogene homolog 2 (AKT2), transcript variant 1, mRNA [NM_001626] | 1.392222 | 1.395104 | -0.00288 | 0.980832 |
| STK24 | Homo sapiens serine/threonine kinase 24 (STK24), transcript variant 2, mRNA [NM_001032296] | 1.382209 | 1.385073 | -0.00286 | 0.983382 |
| ULK2 | Homo sapiens unc-51-like kinase 2 (C. elegans) (ULK2), transcript variant 1, mRNA [NM_014683] | 1.29656 | 1.299379 | -0.00282 | 0.951646 |
| PTPRJ | Homo sapiens protein tyrosine phosphatase, receptor type, J (PTPRJ), transcript variant 2, mRNA [NM_001098503] | 1.361016 | 1.363827 | -0.00281 | 0.976659 |
| IL4 | Homo sapiens interleukin 4 (IL4), transcript variant 1, mRNA [NM_000589] | 1.3075 | 1.310301 | -0.0028 | 0.960822 |
| ESR2 | Homo sapiens estrogen receptor 2 (ER beta) (ESR2), transcript variant e, mRNA [NM_001214903] | 1.428444 | 1.43124 | -0.0028 | 0.967854 |
| SMAD3 | Homo sapiens SMAD family member 3 (SMAD3), transcript variant 1, mRNA [NM_005902] | 1.332907 | 1.335634 | -0.00273 | 0.922746 |
| ATXN10 | Homo sapiens ataxin 10 (ATXN10), transcript variant 1, mRNA [NM_013236] | 1.28876 | 1.291455 | -0.0027 | 0.937642 |
| GLI2 | Homo sapiens GLI family zinc finger 2 (GLI2), mRNA [NM_005270] | 1.329081 | 1.33161 | -0.00253 | 0.963623 |
| A_33_P3353027 | chimerin (chimaerin) 2 [Source:HGNC Symbol;Acc:1944] [ENST00000461824] | 1.341309 | 1.343799 | -0.00249 | 0.973794 |
| TGIF1 | Homo sapiens TGFB-induced factor homeobox 1 (TGIF1), transcript variant 1, mRNA [NM_170695] | 1.52113 | 1.523587 | -0.00246 | 0.985302 |
| ITGA9 | Homo sapiens integrin, alpha 9 (ITGA9), mRNA [NM_002207] | 1.46404 | 1.466493 | -0.00245 | 0.98748 |
| ICAM1 | Homo sapiens intercellular adhesion molecule 1 (ICAM1), mRNA [NM_000201] | 1.470056 | 1.47249 | -0.00243 | 0.99432 |
| C1S | Homo sapiens complement component 1, s subcomponent (C1S), transcript variant 1, mRNA [NM_201442] | 1.299603 | 1.30198 | -0.00238 | 0.977633 |
| EIF2B5 | Homo sapiens eukaryotic translation initiation factor 2B, subunit 5 epsilon, 82kDa (EIF2B5), mRNA [NM_003907] | 1.360691 | 1.363052 | -0.00236 | 0.969848 |
| SAV1 | Homo sapiens salvador homolog 1 (Drosophila) (SAV1), mRNA [NM_021818] | 1.358803 | 1.361152 | -0.00235 | 0.983879 |
| SOX17 | Homo sapiens SRY (sex determining region Y)-box 17 (SOX17), mRNA [NM_022454] | 1.378407 | 1.380735 | -0.00233 | 0.984736 |
| BMPR1A | Homo sapiens bone morphogenetic protein receptor, type IA (BMPR1A), mRNA [NM_004329] | 1.501892 | 1.504172 | -0.00228 | 0.988266 |
| PML | Homo sapiens promyelocytic leukemia (PML), transcript variant 1, mRNA [NM_033238] | 1.285709 | 1.287978 | -0.00227 | 0.888223 |
| RPS14 | ribosomal protein S14 [Source:HGNC Symbol;Acc:10387] [ENST00000401695] | 1.534743 | 1.537005 | -0.00226 | 0.998856 |
| RPS6KB1 | Homo sapiens ribosomal protein S6 kinase, 70kDa, polypeptide 1 (RPS6KB1), mRNA [NM_003161] | 1.388223 | 1.390468 | -0.00224 | 0.984785 |
| SOAT2 | Homo sapiens sterol O-acyltransferase 2 (SOAT2), mRNA [NM_003578] | 1.410613 | 1.412734 | -0.00212 | 0.979887 |
| SMAD1 | Homo sapiens SMAD family member 1 (SMAD1), transcript variant 1, mRNA [NM_005900] | 1.301354 | 1.303337 | -0.00198 | 0.964569 |
| JUNB | Homo sapiens jun B proto-oncogene (JUNB), mRNA [NM_002229] | 1.659054 | 1.66103 | -0.00198 | 0.999337 |
| ABLIM1 | Homo sapiens actin binding LIM protein 1 (ABLIM1), transcript variant 3, mRNA [NM_001003408] | 1.353078 | 1.354993 | -0.00191 | 0.984785 |
| CCR2 | Homo sapiens chemokine (C-C motif) receptor 2 (CCR2), transcript variant A, mRNA [NM_001123041] | 1.3651 | 1.366908 | -0.00181 | 0.988266 |
| TCF7 | Homo sapiens transcription factor 7 (T-cell specific, HMG-box) (TCF7), transcript variant 1, mRNA [NM_003202] | 1.369702 | 1.37151 | -0.00181 | 0.992494 |
| TAF8 | Homo sapiens TAF8 RNA polymerase II, TATA box binding protein (TBP)-associated factor, 43kDa (TAF8), mRNA [NM_138572] | 1.286234 | 1.288039 | -0.00181 | 0.953461 |
| NLGN3 | Homo sapiens neuroligin 3 (NLGN3), transcript variant 2, mRNA [NM_018977] | 1.432322 | 1.434086 | -0.00176 | 0.990198 |
| NRBP2 | Homo sapiens nuclear receptor binding protein 2 (NRBP2), mRNA [NM_178564] | 1.388735 | 1.390462 | -0.00173 | 0.988432 |
| SFRP4 | Homo sapiens secreted frizzled-related protein 4 (SFRP4), mRNA [NM_003014] | 1.454175 | 1.45585 | -0.00168 | 0.984563 |
| CNP | Homo sapiens 2',3'-cyclic nucleotide 3' phosphodiesterase (CNP), mRNA [NM_033133] | 1.373453 | 1.375105 | -0.00165 | 0.983817 |
| ACVR1 | Homo sapiens activin A receptor, type I (ACVR1), transcript variant 1, mRNA [NM_001105] | 1.381831 | 1.383358 | -0.00153 | 0.989415 |
| MYH6 | Homo sapiens myosin, heavy chain 6, cardiac muscle, alpha (MYH6), mRNA [NM_002471] | 1.392616 | 1.394128 | -0.00151 | 0.98748 |
| COL2A1 | Homo sapiens collagen, type II, alpha 1 (COL2A1), transcript variant 1, mRNA [NM_001844] | 1.332879 | 1.334366 | -0.00149 | 0.969536 |
| FLT3 | Homo sapiens fms-related tyrosine kinase 3 (FLT3), mRNA [NM_004119] | 1.41985 | 1.421332 | -0.00148 | 0.992971 |
| ZIC2 | Homo sapiens Zic family member 2 (ZIC2), mRNA [NM_007129] | 1.344606 | 1.345951 | -0.00135 | 0.989741 |
| VSX2 | Homo sapiens visual system homeobox 2 (VSX2), mRNA [NM_182894] | 1.390554 | 1.391889 | -0.00133 | 0.989695 |
| CHUK | Homo sapiens conserved helix-loop-helix ubiquitous kinase (CHUK), mRNA [NM_001278] | 1.329335 | 1.330625 | -0.00129 | 0.984374 |
| TET1 | Homo sapiens tet methylcytosine dioxygenase 1 (TET1), mRNA [NM_030625] | 1.383044 | 1.384282 | -0.00124 | 0.990801 |
| RAC1 | Homo sapiens ras-related C3 botulinum toxin substrate 1 (rho family, small GTP binding protein Rac1) (RAC1), transcript variant Rac1b, mRNA [NM_018890] | 1.374441 | 1.375635 | -0.00119 | 0.96821 |
| PPARG | Homo sapiens peroxisome proliferator-activated receptor gamma (PPARG), transcript variant 3, mRNA [NM_138711] | 1.428105 | 1.429243 | -0.00114 | 0.980555 |
| KRT4 | Homo sapiens keratin 4 (KRT4), mRNA [NM_002272] | 1.33361 | 1.334702 | -0.00109 | 0.984785 |
| AKT2 | Homo sapiens v-akt murine thymoma viral oncogene homolog 2 (AKT2), transcript variant 1, mRNA [NM_001626] | 1.43652 | 1.437475 | -0.00095 | 0.988266 |
| TIPARP | Homo sapiens TCDD-inducible poly(ADP-ribose) polymerase (TIPARP), transcript variant 2, mRNA [NM_015508] | 1.356171 | 1.357061 | -0.00089 | 0.988266 |
| SPTBN4 | Homo sapiens spectrin, beta, non-erythrocytic 4 (SPTBN4), transcript variant sigma1, mRNA [NM_020971] | 1.307075 | 1.307964 | -0.00089 | 0.985987 |
| AKT1 | Homo sapiens v-akt murine thymoma viral oncogene homolog 1 (AKT1), transcript variant 1, mRNA [NM_005163] | 1.332787 | 1.333661 | -0.00087 | 0.9929 |
| TACC2 | Homo sapiens transforming, acidic coiled-coil containing protein 2 (TACC2), transcript variant 1, mRNA [NM_206862] | 1.307048 | 1.307906 | -0.00086 | 0.985987 |
| TMSL3 | Homo sapiens thymosin beta 4, X-linked (TMSB4X), mRNA [NM_021109] | 1.28967 | 1.290474 | -0.0008 | 0.981083 |
| ELF5 | Homo sapiens E74-like factor 5 (ets domain transcription factor) (ELF5), transcript variant 1, mRNA [NM_198381] | 1.443329 | 1.444119 | -0.00079 | 0.991475 |
| TFAP2A | transcription factor AP-2 alpha (activating enhancer binding protein 2 alpha) [Source:HGNC Symbol;Acc:11742] [ENST00000478375] | 1.292742 | 1.293515 | -0.00077 | 0.980693 |
| CSF1 | Homo sapiens colony stimulating factor 1 (macrophage) (CSF1), transcript variant 2, mRNA [NM_172210] | 1.286372 | 1.287078 | -0.00071 | 0.979579 |
| DCDC2 | Homo sapiens doublecortin domain containing 2 (DCDC2), transcript variant 1, mRNA [NM_016356] | 2.383421 | 2.384 | -0.00058 | 0.999927 |
| HGF | Homo sapiens hepatocyte growth factor (hepapoietin A; scatter factor) (HGF), transcript variant 5, mRNA [NM_001010934] | 1.439248 | 1.439763 | -0.00052 | 0.997687 |
| TRPC5 | Homo sapiens transient receptor potential cation channel, subfamily C, member 5 (TRPC5), mRNA [NM_012471] | 1.296194 | 1.296702 | -0.00051 | 0.989695 |
| SRD5A1 | Homo sapiens steroid-5-alpha-reductase, alpha polypeptide 1 (3-oxo-5 alpha-steroid delta 4-dehydrogenase alpha 1) (SRD5A1), mRNA [NM_001047] | 1.330153 | 1.330565 | -0.00041 | 0.987386 |
| BPGM | Homo sapiens 2,3-bisphosphoglycerate mutase (BPGM), transcript variant 2, mRNA [NM_199186] | 1.292691 | 1.293072 | -0.00038 | 0.990801 |
| PEX7 | Homo sapiens peroxisomal biogenesis factor 7 (PEX7), mRNA [NM_000288] | 1.284437 | 1.284722 | -0.00028 | 0.98732 |
| HEXA | Homo sapiens hexosaminidase A (alpha polypeptide) (HEXA), mRNA [NM_000520] | 1.356593 | 1.356843 | -0.00025 | 0.998166 |
| NAB2 | Homo sapiens NGFI-A binding protein 2 (EGR1 binding protein 2) (NAB2), mRNA [NM_005967] | 1.283594 | 1.283767 | -0.00017 | 0.988266 |
| ENST00000366784 | inositol-trisphosphate 3-kinase B [Source:HGNC Symbol;Acc:6179] [ENST00000366784] | 1.290277 | 1.290437 | -0.00016 | 0.99566 |
| FJ169610 | GATA binding protein 4 [Source:HGNC Symbol;Acc:4173] [ENST00000532977] | 1.285073 | 1.28514 | -6.7E-05 | 0.995472 |
| EPHB1 | Homo sapiens EPH receptor B1 (EPHB1), mRNA [NM_004441] | 1.366765 | 1.366791 | -2.6E-05 | 0.999623 |
| ERRFI1 | Homo sapiens ERBB receptor feedback inhibitor 1 (ERRFI1), mRNA [NM_018948] | 1.284438 | 1.284454 | -1.6E-05 | 0.99902 |
| WNT1 | Homo sapiens wingless-type MMTV integration site family, member 1 (WNT1), mRNA [NM_005430] | 1.283839 | 1.283854 | -1.5E-05 | 0.998856 |
| CLTA | Homo sapiens clathrin, light chain A (CLTA), transcript variant 2, mRNA [NM_007096] | 1.287339 | 1.287209 | 0.000131 | 0.995969 |
| OPCML | Homo sapiens opioid binding protein/cell adhesion molecule-like (OPCML), transcript variant 2, mRNA [NM_001012393] | 1.283622 | 1.283411 | 0.000211 | 0.975208 |
| ASB1 | Homo sapiens ankyrin repeat and SOCS box containing 1 (ASB1), mRNA [NM_001040445] | 1.3738 | 1.373547 | 0.000253 | 0.99804 |
| AVPR1A | Homo sapiens arginine vasopressin receptor 1A (AVPR1A), mRNA [NM_000706] | 1.375462 | 1.375159 | 0.000303 | 0.996578 |
| TSHR | Homo sapiens thyroid stimulating hormone receptor (TSHR), transcript variant 2, mRNA [NM_001018036] | 1.342637 | 1.342321 | 0.000316 | 0.997564 |
| HDAC2 | Homo sapiens histone deacetylase 2 (HDAC2), transcript variant 1, mRNA [NM_001527] | 1.290968 | 1.290524 | 0.000443 | 0.98748 |
| SEMA4C | Homo sapiens sema domain, immunoglobulin domain (Ig), transmembrane domain (TM) and short cytoplasmic domain, (semaphorin) 4C (SEMA4C), mRNA [NM_017789] | 1.38326 | 1.382797 | 0.000463 | 0.994557 |
| PDGFRB | Homo sapiens platelet-derived growth factor receptor, beta polypeptide (PDGFRB), mRNA [NM_002609] | 1.445165 | 1.444687 | 0.000478 | 0.9944 |
| ADAMTS9 | Homo sapiens ADAM metallopeptidase with thrombospondin type 1 motif, 9 (ADAMTS9), mRNA [NM_182920] | 1.306643 | 1.306148 | 0.000495 | 0.989344 |
| NFIB | Homo sapiens nuclear factor I/B (NFIB), transcript variant 3, mRNA [NM_005596] | 1.306046 | 1.305543 | 0.000503 | 0.9944 |
| EDN1 | Homo sapiens endothelin 1 (EDN1), transcript variant 1, mRNA [NM_001955] | 1.488855 | 1.48834 | 0.000515 | 0.997669 |
| MESP1 | Homo sapiens mesoderm posterior 1 homolog (mouse) (MESP1), mRNA [NM_018670] | 1.285933 | 1.285292 | 0.000641 | 0.979887 |
| NAB1 | Homo sapiens NGFI-A binding protein 1 (EGR1 binding protein 1) (NAB1), mRNA [NM_005966] | 1.437374 | 1.436642 | 0.000732 | 0.988986 |
| CASP2 | Homo sapiens caspase 2, apoptosis-related cysteine peptidase (CASP2), transcript variant 1, mRNA [NM_032982] | 1.289548 | 1.288799 | 0.000749 | 0.975197 |
| A_19_P00315668 | Homo sapiens homeodomain interacting protein kinase 2 (HIPK2), transcript variant 1, mRNA [NM_022740] | 1.44849 | 1.447685 | 0.000805 | 0.991067 |
| MEF2D | Homo sapiens myocyte enhancer factor 2D (MEF2D), mRNA [NM_005920] | 1.384514 | 1.383698 | 0.000816 | 0.991587 |
| SYNE1 | Homo sapiens spectrin repeat containing, nuclear envelope 1 (SYNE1), transcript variant 1, mRNA [NM_182961] | 1.28829 | 1.287446 | 0.000843 | 0.965168 |
| THC2715000 | enabled homolog (Drosophila) [Source:HGNC Symbol;Acc:18271] [ENST00000498108] | 1.285964 | 1.285103 | 0.000862 | 0.904306 |
| NFIB | Homo sapiens nuclear factor I/B (NFIB), transcript variant 3, mRNA [NM_005596] | 1.311746 | 1.310846 | 0.0009 | 0.984929 |
| PLXNA4 | Homo sapiens plexin A4 (PLXNA4), transcript variant 3, mRNA [NM_001105543] | 1.444059 | 1.443139 | 0.00092 | 0.995184 |
| MYEF2 | Homo sapiens myelin expression factor 2 (MYEF2), mRNA [NM_016132] | 1.287054 | 1.286092 | 0.000962 | 0.973419 |
| IRF4 | Homo sapiens interferon regulatory factor 4 (IRF4), transcript variant 1, mRNA [NM_002460] | 1.292336 | 1.291275 | 0.001061 | 0.969466 |
| PLXNB3 | Homo sapiens plexin B3 (PLXNB3), transcript variant 2, mRNA [NM_001163257] | 1.288923 | 1.287842 | 0.001081 | 0.950128 |
| PAX1 | Homo sapiens paired box 1 (PAX1), mRNA [NM_006192] | 1.307841 | 1.306733 | 0.001108 | 0.979568 |
| EIF2S2 | Homo sapiens eukaryotic translation initiation factor 2, subunit 2 beta, 38kDa (EIF2S2), mRNA [NM_003908] | 1.329852 | 1.328731 | 0.001121 | 0.976897 |
| GABBR1 | Homo sapiens gamma-aminobutyric acid (GABA) B receptor, 1 (GABBR1), transcript variant 1, mRNA [NM_001470] | 1.318659 | 1.317492 | 0.001166 | 0.981249 |
| ENST00000369297 | EPH receptor A7 [Source:HGNC Symbol;Acc:3390] [ENST00000369297] | 1.399208 | 1.398041 | 0.001167 | 0.988266 |
| ITGA2B | Homo sapiens integrin, alpha 2b (platelet glycoprotein IIb of IIb/IIIa complex, antigen CD41) (ITGA2B), mRNA [NM_000419] | 1.47531 | 1.473987 | 0.001323 | 0.993869 |
| PCM1 | Homo sapiens pericentriolar material 1 (PCM1), mRNA [NM_006197] | 1.288525 | 1.28717 | 0.001355 | 0.905507 |
| EGR2 | Homo sapiens early growth response 2 (EGR2), transcript variant 1, mRNA [NM_000399] | 1.327072 | 1.325703 | 0.001369 | 0.982929 |
| PPP3R1 | Homo sapiens protein phosphatase 3, regulatory subunit B, alpha (PPP3R1), mRNA [NM_000945] | 1.306328 | 1.304919 | 0.001408 | 0.98547 |
| MAP7 | Homo sapiens microtubule-associated protein 7 (MAP7), transcript variant 4, mRNA [NM_003980] | 1.306054 | 1.304646 | 0.001409 | 0.987185 |
| PITX1 | Homo sapiens paired-like homeodomain 1 (PITX1), mRNA [NM_002653] | 1.344117 | 1.342704 | 0.001413 | 0.9902 |
| MSR1 | Homo sapiens macrophage scavenger receptor 1 (MSR1), transcript variant SR-AI, mRNA [NM_138715] | 1.323986 | 1.322569 | 0.001418 | 0.987798 |
| FOXA2 | Homo sapiens forkhead box A2 (FOXA2), transcript variant 1, mRNA [NM_021784] | 1.43402 | 1.432538 | 0.001482 | 0.992356 |
| SLITRK5 | Homo sapiens SLIT and NTRK-like family, member 5 (SLITRK5), mRNA [NM_015567] | 1.398548 | 1.397025 | 0.001523 | 0.984785 |
| PCYT1B | Homo sapiens phosphate cytidylyltransferase 1, choline, beta (PCYT1B), transcript variant 1, mRNA [NM_004845] | 1.289663 | 1.28809 | 0.001573 | 0.90528 |
| L3MBTL | Homo sapiens l(3)mbt-like 1 (Drosophila) (L3MBTL1), transcript variant II, mRNA [NM_032107] | 1.288958 | 1.287327 | 0.001632 | 0.948009 |
| DPYSL5 | Homo sapiens dihydropyrimidinase-like 5 (DPYSL5), mRNA [NM_020134] | 1.329008 | 1.327351 | 0.001658 | 0.966905 |
| NCDN | Homo sapiens neurochondrin (NCDN), transcript variant 3, mRNA [NM_014284] | 1.28804 | 1.286371 | 0.00167 | 0.911103 |
| ATP7A | Homo sapiens ATPase, Cu++ transporting, alpha polypeptide (ATP7A), mRNA [NM_000052] | 1.340527 | 1.338845 | 0.001682 | 0.989695 |
| LCE2C | Homo sapiens late cornified envelope 2C (LCE2C), mRNA [NM_178429] | 1.291816 | 1.290109 | 0.001707 | 0.92497 |
| TNFSF11 | Homo sapiens tumor necrosis factor (ligand) superfamily, member 11 (TNFSF11), transcript variant 2, mRNA [NM_033012] | 1.361471 | 1.359741 | 0.001729 | 0.982276 |
| A_19_P00316199 | Homo sapiens striatin, calmodulin binding protein (STRN), mRNA [NM_003162] | 1.292971 | 1.291171 | 0.0018 | 0.93472 |
| LZTS1 | Homo sapiens leucine zipper, putative tumor suppressor 1 (LZTS1), mRNA [NM_021020] | 1.766873 | 1.765071 | 0.001802 | 0.999273 |
| ATG5 | Homo sapiens ATG5 autophagy related 5 homolog (S. cerevisiae) (ATG5), mRNA [NM_004849] | 1.353351 | 1.351495 | 0.001856 | 0.955578 |
| FZD2 | Homo sapiens frizzled family receptor 2 (FZD2), mRNA [NM_001466] | 1.292856 | 1.290995 | 0.00186 | 0.923908 |
| VASP | Homo sapiens vasodilator-stimulated phosphoprotein (VASP), mRNA [NM_003370] | 1.398648 | 1.39676 | 0.001888 | 0.974137 |
| GOPC | Homo sapiens golgi-associated PDZ and coiled-coil motif containing (GOPC), transcript variant 1, mRNA [NM_020399] | 1.30508 | 1.303164 | 0.001916 | 0.951646 |
| BAD | Homo sapiens BCL2-associated agonist of cell death (BAD), transcript variant 1, mRNA [NM_004322] | 1.351362 | 1.349445 | 0.001916 | 0.984339 |
| HSP90AB1 | Homo sapiens heat shock protein 90kDa alpha (cytosolic), class B member 1 (HSP90AB1), mRNA [NM_007355] | 1.329314 | 1.32739 | 0.001924 | 0.950656 |
| SPRR4 | Homo sapiens small proline-rich protein 4 (SPRR4), mRNA [NM_173080] | 3.236853 | 3.234928 | 0.001926 | 0.999337 |
| ASPA | Homo sapiens aspartoacylase (ASPA), transcript variant 1, mRNA [NM_000049] | 1.294017 | 1.291902 | 0.002115 | 0.916588 |
| ENST00000373976 | protein kinase, cGMP-dependent, type I [Source:HGNC Symbol;Acc:9414] [ENST00000373976] | 1.29997 | 1.297844 | 0.002126 | 0.924628 |
| CCL21 | Homo sapiens chemokine (C-C motif) ligand 21 (CCL21), mRNA [NM_002989] | 1.389365 | 1.387237 | 0.002129 | 0.982811 |
| A_19_P00806490 | Homo sapiens tet methylcytosine dioxygenase 1 (TET1), mRNA [NM_030625] | 1.312936 | 1.310791 | 0.002145 | 0.960534 |
| CDON | Homo sapiens Cdon homolog (mouse) (CDON), transcript variant 2, mRNA [NM_016952] | 1.296991 | 1.294779 | 0.002211 | 0.935319 |
| SPTAN1 | Homo sapiens spectrin, alpha, non-erythrocytic 1 (alpha-fodrin) (SPTAN1), transcript variant 2, mRNA [NM_003127] | 2.701418 | 2.699174 | 0.002243 | 0.999491 |
| EGFL7 | Homo sapiens EGF-like-domain, multiple 7 (EGFL7), transcript variant 2, mRNA [NM_201446] | 2.302457 | 2.300148 | 0.002309 | 0.999487 |
| PTPRC | Homo sapiens protein tyrosine phosphatase, receptor type, C (PTPRC), transcript variant 1, mRNA [NM_002838] | 1.447679 | 1.445335 | 0.002345 | 0.972526 |
| PTPRQ | Homo sapiens protein tyrosine phosphatase, receptor type, Q (PTPRQ), mRNA [NM_001145026] | 1.536791 | 1.534401 | 0.002389 | 0.988266 |
| SLIT1 | Homo sapiens slit homolog 1 (Drosophila) (SLIT1), mRNA [NM_003061] | 1.457399 | 1.454894 | 0.002505 | 0.986638 |
| RNF6 | Homo sapiens ring finger protein (C3H2C3 type) 6 (RNF6), transcript variant 1, mRNA [NM_005977] | 1.553008 | 1.550465 | 0.002543 | 0.992036 |
| PGR | Homo sapiens progesterone receptor (PGR), transcript variant 2, mRNA [NM_000926] | 1.315218 | 1.312654 | 0.002565 | 0.948019 |
| AGTPBP1 | Homo sapiens ATP/GTP binding protein 1 (AGTPBP1), mRNA [NM_015239] | 1.391434 | 1.388858 | 0.002576 | 0.977749 |
| KLF15 | Homo sapiens Kruppel-like factor 15 (KLF15), mRNA [NM_014079] | 1.4204 | 1.417783 | 0.002617 | 0.973465 |
| BTG2 | Homo sapiens BTG family, member 2 (BTG2), mRNA [NM_006763] | 1.444806 | 1.442044 | 0.002762 | 0.965933 |
| RACGAP1 | Homo sapiens Rac GTPase activating protein 1 (RACGAP1), transcript variant 1, mRNA [NM_013277] | 1.317198 | 1.314401 | 0.002797 | 0.944508 |
| CASP3 | Homo sapiens caspase 3, apoptosis-related cysteine peptidase (CASP3), transcript variant alpha, mRNA [NM_004346] | 1.592699 | 1.589877 | 0.002822 | 0.996482 |
| SEMA3C | Homo sapiens sema domain, immunoglobulin domain (Ig), short basic domain, secreted, (semaphorin) 3C (SEMA3C), mRNA [NM_006379] | 1.297591 | 1.294765 | 0.002825 | 0.888223 |
| LOC728137 | Homo sapiens testis specific protein, Y-linked 3 (TSPY3), mRNA [NM_001077697] | 1.447668 | 1.444828 | 0.00284 | 0.987843 |
| EPHA3 | Homo sapiens EPH receptor A3 (EPHA3), transcript variant 2, mRNA [NM_182644] | 1.461969 | 1.459043 | 0.002927 | 0.98732 |
| WDR77 | Homo sapiens WD repeat domain 77 (WDR77), mRNA [NM_024102] | 1.435614 | 1.432665 | 0.002949 | 0.966905 |
| CCR2 | Homo sapiens chemokine (C-C motif) receptor 2 (CCR2), transcript variant B, mRNA [NM_001123396] | 1.324941 | 1.321701 | 0.00324 | 0.928887 |
| HPS4 | Homo sapiens Hermansky-Pudlak syndrome 4 (HPS4), transcript variant 1, mRNA [NM_022081] | 1.388962 | 1.385663 | 0.003299 | 0.970869 |
| RND1 | Homo sapiens Rho family GTPase 1 (RND1), mRNA [NM_014470] | 1.558676 | 1.555352 | 0.003324 | 0.997943 |
| LAMB2 | Homo sapiens laminin, beta 2 (laminin S) (LAMB2), mRNA [NM_002292] | 4.092429 | 4.089049 | 0.00338 | 0.998856 |
| C12orf29 | Homo sapiens centrosomal protein 290kDa (CEP290), mRNA [NM_025114] | 1.299141 | 1.295758 | 0.003383 | 0.890447 |
| OTP | Homo sapiens orthopedia homeobox (OTP), mRNA [NM_032109] | 1.314713 | 1.311293 | 0.00342 | 0.961978 |
| LMNA | Homo sapiens lamin A/C (LMNA), transcript variant 2, mRNA [NM_005572] | 3.158383 | 3.154901 | 0.003482 | 0.999368 |
| A_19_P00801576 | Homo sapiens CUGBP, Elav-like family member 1 (CELF1), transcript variant 5, mRNA [NM_001172640] | 1.301047 | 1.297484 | 0.003562 | 0.893606 |
| GRB2 | Homo sapiens growth factor receptor-bound protein 2 (GRB2), transcript variant 1, mRNA [NM_002086] | 1.311335 | 1.307694 | 0.003641 | 0.916669 |
| GSK3A | Homo sapiens glycogen synthase kinase 3 alpha (GSK3A), mRNA [NM_019884] | 1.320362 | 1.316638 | 0.003725 | 0.959324 |
| TET2 | Homo sapiens tet methylcytosine dioxygenase 2 (TET2), transcript variant 2, mRNA [NM_017628] | 1.316663 | 1.312915 | 0.003748 | 0.92103 |
| BARX2 | Homo sapiens BARX homeobox 2 (BARX2), mRNA [NM_003658] | 1.31011 | 1.305949 | 0.004161 | 0.937776 |
| LOC284912 | myoglobin [Source:HGNC Symbol;Acc:6915] [ENST00000472240] | 1.406651 | 1.402426 | 0.004225 | 0.973546 |
| SPINK5 | Homo sapiens serine peptidase inhibitor, Kazal type 5 (SPINK5), transcript variant 3, mRNA [NM_001127699] | 1.305889 | 1.301654 | 0.004234 | 0.888223 |
| KGFLP2 | Homo sapiens keratinocyte growth factor-like protein 2 (KGFLP2), non-coding RNA [NR_003670] | 1.780632 | 1.776382 | 0.00425 | 0.999001 |
| RB1 | Homo sapiens retinoblastoma 1 (RB1), mRNA [NM_000321] | 1.406262 | 1.402011 | 0.004251 | 0.959324 |
| ALMS1 | Homo sapiens Alstrom syndrome 1 (ALMS1), mRNA [NM_015120] | 1.307028 | 1.302767 | 0.004261 | 0.894538 |
| FGF5 | Homo sapiens fibroblast growth factor 5 (FGF5), transcript variant 1, mRNA [NM_004464] | 1.329064 | 1.324791 | 0.004274 | 0.943914 |
| SHBG | Homo sapiens sex hormone-binding globulin (SHBG), transcript variant 5, non-coding RNA [NR_027462] | 1.346654 | 1.342301 | 0.004353 | 0.935618 |
| SPRY1 | Homo sapiens sprouty homolog 1, antagonist of FGF signaling (Drosophila) (SPRY1), transcript variant 2, mRNA [NM_199327] | 1.314365 | 1.309917 | 0.004448 | 0.89938 |
| STEAP4 | Homo sapiens STEAP family member 4 (STEAP4), transcript variant 2, mRNA [NM_001205315] | 1.318906 | 1.314434 | 0.004472 | 0.940447 |
| BCL11A | Homo sapiens B-cell CLL/lymphoma 11A (zinc finger protein) (BCL11A), transcript variant 1, mRNA [NM_022893] | 1.324548 | 1.320064 | 0.004484 | 0.941105 |
| MSI1 | Homo sapiens musashi homolog 1 (Drosophila) (MSI1), mRNA [NM_002442] | 2.241804 | 2.237267 | 0.004536 | 0.998856 |
| GLI2 | Homo sapiens GLI family zinc finger 2 (GLI2), mRNA [NM_005270] | 2.33684 | 2.332278 | 0.004562 | 0.998514 |
| ASPM | Homo sapiens asp (abnormal spindle) homolog, microcephaly associated (Drosophila) (ASPM), transcript variant 1, mRNA [NM_018136] | 1.309031 | 1.304437 | 0.004594 | 0.88974 |
| BFSP2 | Homo sapiens beaded filament structural protein 2, phakinin (BFSP2), mRNA [NM_003571] | 1.314126 | 1.309251 | 0.004876 | 0.891708 |
| ACVR1B | Homo sapiens activin A receptor, type IB (ACVR1B), transcript variant 3, mRNA [NM_020328] | 1.317721 | 1.312761 | 0.00496 | 0.9238 |
| CHST11 | Homo sapiens carbohydrate (chondroitin 4) sulfotransferase 11 (CHST11), transcript variant 1, mRNA [NM_018413] | 1.314399 | 1.309383 | 0.005016 | 0.889667 |
| FGF7 | Homo sapiens fibroblast growth factor 7 (FGF7), mRNA [NM_002009] | 1.337847 | 1.332696 | 0.005151 | 0.908164 |
| NF2 | Homo sapiens neurofibromin 2 (merlin) (NF2), transcript variant 8, mRNA [NM_181832] | 1.355955 | 1.350766 | 0.00519 | 0.930648 |
| PRMT2 | Homo sapiens protein arginine methyltransferase 2 (PRMT2), transcript variant 1, mRNA [NM_206962] | 1.359882 | 1.354642 | 0.00524 | 0.937131 |
| MAPK12 | Homo sapiens cDNA, FLJ98809. [AK308768] | 1.320756 | 1.315506 | 0.00525 | 0.894553 |
| TSPY3 | Homo sapiens testis specific protein, Y-linked 2 (TSPY2), mRNA [NM_022573] | 1.309662 | 1.304398 | 0.005264 | 0.888223 |
| CNTF | Homo sapiens ciliary neurotrophic factor (CNTF), mRNA [NM_000614] | 1.315627 | 1.310343 | 0.005284 | 0.888223 |
| SH3GL2 | Homo sapiens SH3-domain GRB2-like 2 (SH3GL2), mRNA [NM_003026] | 1.318901 | 1.313601 | 0.0053 | 0.889199 |
| SIRT1 | Homo sapiens sirtuin 1 (SIRT1), transcript variant 1, mRNA [NM_012238] | 1.384854 | 1.379467 | 0.005388 | 0.951513 |
| STEAP4 | Homo sapiens STEAP family member 4 (STEAP4), transcript variant 2, mRNA [NM_001205315] | 1.304499 | 1.299085 | 0.005414 | 0.888223 |
| NRL | Homo sapiens neural retina leucine zipper (NRL), mRNA [NM_006177] | 1.35475 | 1.349258 | 0.005492 | 0.915657 |
| RPL22 | Homo sapiens ribosomal protein L22 (RPL22), mRNA [NM_000983] | 1.358482 | 1.352948 | 0.005535 | 0.922171 |
| NOTCH2 | Homo sapiens notch 2 (NOTCH2), transcript variant 1, mRNA [NM_024408] | 1.360702 | 1.355145 | 0.005556 | 0.923413 |
| CASP3 | Homo sapiens caspase 3, apoptosis-related cysteine peptidase (CASP3), transcript variant alpha, mRNA [NM_004346] | 1.382742 | 1.377174 | 0.005568 | 0.914216 |
| GRIN2A | Homo sapiens glutamate receptor, ionotropic, N-methyl D-aspartate 2A (GRIN2A), transcript variant 2, mRNA [NM_000833] | 1.545013 | 1.539398 | 0.005615 | 0.977408 |
| SHANK3 | Homo sapiens SH3 and multiple ankyrin repeat domains 3 (SHANK3), mRNA [NM_001080420] | 1.618618 | 1.61297 | 0.005649 | 0.997137 |
| USP33 | Homo sapiens ubiquitin specific peptidase 33 (USP33), transcript variant 3, mRNA [NM_201626] | 1.305871 | 1.300219 | 0.005651 | 0.888223 |
| AMOT | Homo sapiens angiomotin (AMOT), transcript variant 2, mRNA [NM_133265] | 1.347057 | 1.341384 | 0.005672 | 0.921444 |
| BMP15 | Homo sapiens bone morphogenetic protein 15 (BMP15), mRNA [NM_005448] | 1.321101 | 1.31539 | 0.005711 | 0.900741 |
| NR4A3 | Homo sapiens nuclear receptor subfamily 4, group A, member 3 (NR4A3), transcript variant 4, mRNA [NM_173199] | 1.387955 | 1.382217 | 0.005738 | 0.94947 |
| TGM3 | Homo sapiens transglutaminase 3 (E polypeptide, protein-glutamine-gamma-glutamyltransferase) (TGM3), mRNA [NM_003245] | 1.322977 | 1.317206 | 0.005771 | 0.888223 |
| HIF1A | Homo sapiens hypoxia inducible factor 1, alpha subunit (basic helix-loop-helix transcription factor) (HIF1A), transcript variant 2, mRNA [NM_181054] | 1.393657 | 1.387875 | 0.005782 | 0.888223 |
| CYP19A1 | Homo sapiens cytochrome P450, family 19, subfamily A, polypeptide 1, mRNA (cDNA clone IMAGE:5588077), complete cds. [BC035714] | 1.307261 | 1.301461 | 0.005799 | 0.888223 |
| TRPV1 | Homo sapiens transient receptor potential cation channel, subfamily V, member 1 (TRPV1), transcript variant 3, mRNA [NM_080706] | 1.307101 | 1.301221 | 0.00588 | 0.888223 |
| KRTDAP | Homo sapiens keratinocyte differentiation-associated protein (KRTDAP), transcript variant 1, mRNA [NM_207392] | 1.341976 | 1.336036 | 0.00594 | 0.91439 |
| HOPX | Homo sapiens HOP homeobox (HOPX), transcript variant 2, mRNA [NM_139211] | 1.30827 | 1.302314 | 0.005956 | 0.888223 |
| ZFP42 | Homo sapiens zinc finger protein 42 homolog (mouse) (ZFP42), mRNA [NM_174900] | 1.361957 | 1.355986 | 0.005972 | 0.924821 |
| PEX13 | Homo sapiens peroxisomal biogenesis factor 13 (PEX13), mRNA [NM_002618] | 1.312958 | 1.306951 | 0.006007 | 0.888223 |
| EID2B | Homo sapiens EP300 interacting inhibitor of differentiation 2B (EID2B), mRNA [NM_152361] | 1.34444 | 1.338414 | 0.006026 | 0.89938 |
| CEBPG | Homo sapiens CCAAT/enhancer binding protein (C/EBP), gamma (CEBPG), transcript variant 1, mRNA [NM_001806] | 1.345708 | 1.339603 | 0.006105 | 0.916588 |
| SEMA6D | Homo sapiens sema domain, transmembrane domain (TM), and cytoplasmic domain, (semaphorin) 6D (SEMA6D), transcript variant 6, mRNA [NM_024966] | 1.325553 | 1.319319 | 0.006234 | 0.888223 |
| WARS2 | Homo sapiens tryptophanyl tRNA synthetase 2, mitochondrial (WARS2), nuclear gene encoding mitochondrial protein, transcript variant 2, mRNA [NM_201263] | 1.429512 | 1.423249 | 0.006263 | 0.972598 |
| AX746595 | Mov10l1, Moloney leukemia virus 10-like 1, homolog (mouse) [Source:HGNC Symbol;Acc:7201] [ENST00000354853] | 1.337976 | 1.331638 | 0.006338 | 0.888223 |
| GDAP1 | Homo sapiens ganglioside-induced differentiation-associated protein 1 (GDAP1), transcript variant 1, mRNA [NM_018972] | 1.315415 | 1.308979 | 0.006437 | 0.888223 |
| SHROOM3 | Homo sapiens shroom family member 3 (SHROOM3), mRNA [NM_020859] | 1.756937 | 1.750456 | 0.00648 | 0.997741 |
| NF1 | Homo sapiens neurofibromin 1 (NF1), transcript variant 1, mRNA [NM_001042492] | 1.34456 | 1.338069 | 0.006491 | 0.928887 |
| TREM2 | Homo sapiens triggering receptor expressed on myeloid cells 2 (TREM2), mRNA [NM_018965] | 1.319371 | 1.312871 | 0.0065 | 0.888223 |
| DACH2 | Homo sapiens dachshund homolog 2 (Drosophila) (DACH2), transcript variant 1, mRNA [NM_053281] | 1.342913 | 1.336406 | 0.006506 | 0.953461 |
| SYCP2 | Homo sapiens synaptonemal complex protein 2 (SYCP2), mRNA [NM_014258] | 1.428187 | 1.421577 | 0.006609 | 0.991832 |
| DSCAML1 | Homo sapiens Down syndrome cell adhesion molecule like 1 (DSCAML1), mRNA [NM_020693] | 1.316647 | 1.309993 | 0.006654 | 0.888223 |
| DPYSL4 | Homo sapiens dihydropyrimidinase-like 4 (DPYSL4), mRNA [NM_006426] | 1.491764 | 1.484984 | 0.00678 | 0.981083 |
| DST | Homo sapiens dystonin (DST), transcript variant 1eA, mRNA [NM_015548] | 1.329135 | 1.322176 | 0.006959 | 0.888223 |
| KCNQ2 | Homo sapiens potassium voltage-gated channel, KQT-like subfamily, member 2 (KCNQ2), transcript variant 3, mRNA [NM_004518] | 1.343592 | 1.33663 | 0.006962 | 0.942418 |
| HOOK1 | Homo sapiens hook homolog 1 (Drosophila) (HOOK1), mRNA [NM_015888] | 2.687539 | 2.680555 | 0.006983 | 0.99472 |
| RP1 | Homo sapiens retinitis pigmentosa 1 (autosomal dominant) (RP1), mRNA [NM_006269] | 1.329746 | 1.322712 | 0.007034 | 0.888223 |
| STAT5A | Homo sapiens signal transducer and activator of transcription 5A (STAT5A), mRNA [NM_003152] | 1.326735 | 1.319674 | 0.00706 | 0.888223 |
| TRIM11 | Homo sapiens tripartite motif containing 11 (TRIM11), mRNA [NM_145214] | 1.342891 | 1.335821 | 0.007069 | 0.905107 |
| SRY | Homo sapiens sex determining region Y (SRY), mRNA [NM_003140] | 1.321085 | 1.313966 | 0.007119 | 0.888223 |
| TDRD1 | Homo sapiens tudor domain containing 1 (TDRD1), mRNA [NM_198795] | 1.328798 | 1.321654 | 0.007145 | 0.888223 |
| GDAP2 | Homo sapiens ganglioside induced differentiation associated protein 2 (GDAP2), transcript variant 1, mRNA [NM_017686] | 1.415844 | 1.408668 | 0.007176 | 0.888223 |
| IL7R | Homo sapiens interleukin 7 receptor (IL7R), mRNA [NM_002185] | 1.508614 | 1.501379 | 0.007236 | 0.970992 |
| PDGFRA | Homo sapiens platelet-derived growth factor receptor, alpha polypeptide (PDGFRA), mRNA [NM_006206] | 1.345462 | 1.338192 | 0.00727 | 0.89938 |
| RELN | Homo sapiens reelin (RELN), transcript variant 1, mRNA [NM_005045] | 1.317877 | 1.310595 | 0.007283 | 0.888223 |
| VEZF1 | Homo sapiens vascular endothelial zinc finger 1 (VEZF1), mRNA [NM_007146] | 1.368937 | 1.361595 | 0.007342 | 0.900515 |
| ANTXR1 | Homo sapiens anthrax toxin receptor 1 (ANTXR1), transcript variant 1, mRNA [NM_032208] | 1.319791 | 1.312392 | 0.007399 | 0.888223 |
| EIF2S2 | Homo sapiens eukaryotic translation initiation factor 2, subunit 2 beta, 38kDa (EIF2S2), mRNA [NM_003908] | 1.292543 | 1.285093 | 0.00745 | 0.888223 |
| SEMA7A | Homo sapiens semaphorin 7A, GPI membrane anchor (John Milton Hagen blood group) (SEMA7A), transcript variant 1, mRNA [NM_003612] | 1.429954 | 1.422471 | 0.007483 | 0.90773 |
| MYH9 | Homo sapiens myosin, heavy chain 9, non-muscle (MYH9), mRNA [NM_002473] | 1.338842 | 1.331319 | 0.007523 | 0.888223 |
| ATOH1 | Homo sapiens atonal homolog 1 (Drosophila) (ATOH1), mRNA [NM_005172] | 1.483019 | 1.47548 | 0.007539 | 0.974551 |
| TBC1D24 | Homo sapiens TBC1 domain family, member 24 (TBC1D24), transcript variant 2, mRNA [NM_020705] | 1.397135 | 1.389595 | 0.00754 | 0.888559 |
| BARX1 | Homo sapiens BARX homeobox 1 (BARX1), mRNA [NM_021570] | 1.322428 | 1.314668 | 0.00776 | 0.888223 |
| GDF5OS | growth differentiation factor 5 opposite strand [Source:HGNC Symbol;Acc:33435] [ENST00000374375] | 4.018424 | 4.01051 | 0.007914 | 0.997652 |
| ITGAV | Homo sapiens integrin, alpha V (vitronectin receptor, alpha polypeptide, antigen CD51) (ITGAV), transcript variant 1, mRNA [NM_002210] | 1.48961 | 1.481549 | 0.00806 | 0.982171 |
| GLP1R | Homo sapiens glucagon-like peptide 1 receptor (GLP1R), mRNA [NM_002062] | 1.394487 | 1.386422 | 0.008064 | 0.932317 |
| LOR | Homo sapiens loricrin (LOR), mRNA [NM_000427] | 1.414092 | 1.406003 | 0.008089 | 0.9514 |
| IL6R | Homo sapiens interleukin 6 receptor (IL6R), transcript variant 1, mRNA [NM_000565] | 1.397169 | 1.388962 | 0.008207 | 0.96505 |
| DAZL | Homo sapiens deleted in azoospermia-like (DAZL), transcript variant 2, mRNA [NM_001351] | 1.336077 | 1.327804 | 0.008273 | 0.888223 |
| ADIG | Homo sapiens adipogenin (ADIG), mRNA [NM_001018082] | 1.438171 | 1.429857 | 0.008314 | 0.962759 |
| CDC42 | Homo sapiens cell division cycle 42 (GTP binding protein, 25kDa) (CDC42), transcript variant 3, mRNA [NM_001039802] | 1.388138 | 1.37972 | 0.008418 | 0.888223 |
| PLK4 | Homo sapiens polo-like kinase 4 (PLK4), transcript variant 1, mRNA [NM_014264] | 1.335453 | 1.327019 | 0.008434 | 0.888223 |
| EIF2B4 | Homo sapiens eukaryotic translation initiation factor 2B, subunit 4 delta, 67kDa (EIF2B4), transcript variant 2, mRNA [NM_001034116] | 1.327504 | 1.319062 | 0.008441 | 0.888223 |
| RBMY1B | Homo sapiens RNA binding motif protein, Y-linked, family 1, member B (RBMY1B), mRNA [NM_001006121] | 1.321903 | 1.313435 | 0.008468 | 0.888223 |
| PRICKLE2 | Homo sapiens prickle homolog 2 (Drosophila) (PRICKLE2), mRNA [NM_198859] | 1.323685 | 1.315098 | 0.008587 | 0.888223 |
| TET2 | Homo sapiens tet methylcytosine dioxygenase 2 (TET2), transcript variant 1, mRNA [NM_001127208] | 1.324473 | 1.315789 | 0.008684 | 0.888223 |
| FANCA | Homo sapiens Fanconi anemia, complementation group A (FANCA), transcript variant 1, mRNA [NM_000135] | 1.380272 | 1.371346 | 0.008927 | 0.888223 |
| CTHRC1 | Homo sapiens collagen triple helix repeat containing 1 (CTHRC1), mRNA [NM_138455] | 1.540808 | 1.531827 | 0.00898 | 0.986008 |
| NFKB1 | Homo sapiens nuclear factor of kappa light polypeptide gene enhancer in B-cells 1 (NFKB1), transcript variant 1, mRNA [NM_003998] | 1.378374 | 1.369368 | 0.009006 | 0.897347 |
| SMAD9 | Homo sapiens SMAD family member 9 (SMAD9), transcript variant b, mRNA [NM_005905] | 1.733837 | 1.7248 | 0.009037 | 0.995797 |
| FGF2 | Homo sapiens fibroblast growth factor 2 (basic) (FGF2), mRNA [NM_002006] | 1.34059 | 1.331458 | 0.009132 | 0.888223 |
| TNFSF4 | Homo sapiens tumor necrosis factor (ligand) superfamily, member 4 (TNFSF4), mRNA [NM_003326] | 1.421873 | 1.412621 | 0.009253 | 0.888223 |
| LDB2 | Homo sapiens LIM domain binding 2 (LDB2), transcript variant 1, mRNA [NM_001290] | 1.331112 | 1.321769 | 0.009342 | 0.888223 |
| POU3F4 | Homo sapiens POU class 3 homeobox 4 (POU3F4), mRNA [NM_000307] | 1.711932 | 1.702583 | 0.00935 | 0.996752 |
| EIF2B3 | Homo sapiens eukaryotic translation initiation factor 2B, subunit 3 gamma, 58kDa (EIF2B3), transcript variant 1, mRNA [NM_020365] | 1.485253 | 1.475902 | 0.009351 | 0.969741 |
| RCAN1 | Homo sapiens regulator of calcineurin 1 (RCAN1), transcript variant 1, mRNA [NM_004414] | 1.340493 | 1.330876 | 0.009617 | 0.888223 |
| SATB2 | Homo sapiens SATB homeobox 2 (SATB2), transcript variant 2, mRNA [NM_015265] | 1.458648 | 1.448965 | 0.009683 | 0.965302 |
| CYP26A1 | Homo sapiens cytochrome P450, family 26, subfamily A, polypeptide 1 (CYP26A1), transcript variant 2, mRNA [NM_057157] | 1.490058 | 1.480281 | 0.009777 | 0.965932 |
| OPHN1 | Homo sapiens oligophrenin 1 (OPHN1), mRNA [NM_002547] | 1.330301 | 1.320444 | 0.009857 | 0.888223 |
| NBR1 | Homo sapiens neighbor of BRCA1 gene 1 (NBR1), transcript variant 2, mRNA [NM_031858] | 1.430532 | 1.420645 | 0.009887 | 0.888223 |
| FNDC3B | Homo sapiens fibronectin type III domain containing 3B (FNDC3B), transcript variant 1, mRNA [NM_022763] | 1.334368 | 1.324365 | 0.010003 | 0.888223 |
| ENST00000381984 | regulatory factor X, 3 (influences HLA class II expression) [Source:HGNC Symbol;Acc:9984] [ENST00000381984] | 1.335174 | 1.32511 | 0.010064 | 0.888223 |
| FZD8 | Homo sapiens frizzled family receptor 8 (FZD8), mRNA [NM_031866] | 1.330359 | 1.320248 | 0.010111 | 0.888223 |
| IL4R | interleukin 4 receptor [Source:HGNC Symbol;Acc:6015] [ENST00000380922] | 1.449776 | 1.439603 | 0.010173 | 0.959324 |
| TIMP1 | Homo sapiens TIMP metallopeptidase inhibitor 1 (TIMP1), mRNA [NM_003254] | 1.43782 | 1.427515 | 0.010305 | 0.956298 |
| CEP57 | Homo sapiens centrosomal protein 57kDa (CEP57), transcript variant 1, mRNA [NM_014679] | 1.332077 | 1.321487 | 0.01059 | 0.888223 |
| CAPN2 | Homo sapiens calpain 2, (m/II) large subunit (CAPN2), transcript variant 1, mRNA [NM_001748] | 1.332905 | 1.322224 | 0.010681 | 0.888223 |
| CRB1 | Homo sapiens crumbs homolog 1 (Drosophila) (CRB1), transcript variant 1, mRNA [NM_201253] | 1.332502 | 1.321812 | 0.01069 | 0.888223 |
| TUBB2B | Homo sapiens tubulin, beta 2B class IIb (TUBB2B), mRNA [NM_178012] | 1.481846 | 1.471139 | 0.010707 | 0.970182 |
| HCN1 | Homo sapiens hyperpolarization activated cyclic nucleotide-gated potassium channel 1 (HCN1), mRNA [NM_021072] | 1.343763 | 1.333049 | 0.010714 | 0.917661 |
| CLASP1 | Homo sapiens cytoplasmic linker associated protein 1 (CLASP1), transcript variant 1, mRNA [NM_015282] | 1.460037 | 1.448915 | 0.011122 | 0.959324 |
| PTCD2 | Homo sapiens pentatricopeptide repeat domain 2 (PTCD2), mRNA [NM_024754] | 1.419894 | 1.408769 | 0.011125 | 0.935544 |
| MT1G | Homo sapiens metallothionein 1G (MT1G), mRNA [NM_005950] | 1.343187 | 1.332025 | 0.011162 | 0.924131 |
| LCE3D | Homo sapiens late cornified envelope 3D (LCE3D), mRNA [NM_032563] | 1.422526 | 1.411244 | 0.011282 | 0.918242 |
| NOX4 | Homo sapiens NADPH oxidase 4 (NOX4), transcript variant 2, mRNA [NM_001143836] | 1.358722 | 1.347385 | 0.011337 | 0.888223 |
| GPR98 | Homo sapiens G protein-coupled receptor 98 (GPR98), transcript variant 1, mRNA [NM_032119] | 1.329669 | 1.318242 | 0.011427 | 0.917939 |
| LRP6 | Homo sapiens low density lipoprotein receptor-related protein 6 (LRP6), mRNA [NM_002336] | 1.340881 | 1.329443 | 0.011438 | 0.888223 |
| CAV2 | Homo sapiens caveolin 2 (CAV2), transcript variant 1, mRNA [NM_001233] | 1.397645 | 1.385845 | 0.0118 | 0.916333 |
| PHOX2A | Homo sapiens paired-like homeobox 2a (PHOX2A), mRNA [NM_005169] | 1.39411 | 1.382085 | 0.012025 | 0.899681 |
| PRTN3 | Homo sapiens proteinase 3 (PRTN3), mRNA [NM_002777] | 1.392787 | 1.380659 | 0.012128 | 0.898499 |
| NTRK3 | neurotrophic tyrosine kinase, receptor, type 3 [Source:HGNC Symbol;Acc:8033] [ENST00000394480] | 1.362429 | 1.350248 | 0.012181 | 0.888223 |
| TRPS1 | Homo sapiens trichorhinophalangeal syndrome I (TRPS1), mRNA [NM_014112] | 1.452302 | 1.439881 | 0.012422 | 0.92497 |
| GNAT1 | Homo sapiens guanine nucleotide binding protein (G protein), alpha transducing activity polypeptide 1 (GNAT1), transcript variant 2, mRNA [NM_000172] | 1.667199 | 1.654738 | 0.012461 | 0.994697 |
| ZEB2 | Homo sapiens zinc finger E-box binding homeobox 2 (ZEB2), transcript variant 1, mRNA [NM_014795] | 1.336451 | 1.323846 | 0.012605 | 0.888223 |
| FZD7 | Homo sapiens frizzled family receptor 7 (FZD7), mRNA [NM_003507] | 1.34469 | 1.332071 | 0.012619 | 0.888223 |
| EVL | Homo sapiens Enah/Vasp-like (EVL), mRNA [NM_016337] | 1.421276 | 1.408631 | 0.012645 | 0.926845 |
| DMD | Homo sapiens dystrophin (DMD), transcript variant Dp427p2, mRNA [NM_004010] | 1.509033 | 1.496381 | 0.012652 | 0.949287 |
| FGFR1 | Homo sapiens fibroblast growth factor receptor 1 (FGFR1), transcript variant 13, mRNA [NM_001174066] | 1.341352 | 1.328637 | 0.012715 | 0.888223 |
| NRD1 | Homo sapiens nardilysin (N-arginine dibasic convertase) (NRD1), transcript variant 1, mRNA [NM_002525] | 1.339932 | 1.327137 | 0.012795 | 0.888223 |
| GDAP2 | Homo sapiens ganglioside induced differentiation associated protein 2 (GDAP2), transcript variant 1, mRNA [NM_017686] | 1.417193 | 1.40397 | 0.013223 | 0.917845 |
| ANK2 | Homo sapiens ankyrin 2, neuronal (ANK2), transcript variant 1, mRNA [NM_001148] | 1.552904 | 1.53958 | 0.013324 | 0.935662 |
| MORF4L2 | Homo sapiens mortality factor 4 like 2 (MORF4L2), transcript variant 2, mRNA [NM_012286] | 1.39526 | 1.38191 | 0.01335 | 0.888223 |
| PPARGC1A | Homo sapiens peroxisome proliferator-activated receptor gamma, coactivator 1 alpha (PPARGC1A), mRNA [NM_013261] | 1.433082 | 1.419614 | 0.013469 | 0.934679 |
| CEP120 | Homo sapiens centrosomal protein 120kDa (CEP120), transcript variant 1, mRNA [NM_153223] | 1.529057 | 1.515573 | 0.013484 | 0.92693 |
| TBX1 | Homo sapiens T-box 1 (TBX1), transcript variant A, mRNA [NM_080646] | 1.414139 | 1.400654 | 0.013485 | 0.913827 |
| PDLIM7 | Homo sapiens PDZ and LIM domain 7 (enigma) (PDLIM7), transcript variant 1, mRNA [NM_005451] | 1.515929 | 1.502249 | 0.01368 | 0.991067 |
| ULK1 | Homo sapiens unc-51-like kinase 1 (C. elegans) (ULK1), mRNA [NM_003565] | 1.479653 | 1.465972 | 0.01368 | 0.957178 |
| PARD6B | Homo sapiens par-6 partitioning defective 6 homolog beta (C. elegans) (PARD6B), mRNA [NM_032521] | 1.38422 | 1.370375 | 0.013845 | 0.888223 |
| RAF1 | Homo sapiens v-raf-1 murine leukemia viral oncogene homolog 1 (RAF1), mRNA [NM_002880] | 3.755534 | 3.74162 | 0.013914 | 0.995161 |
| GJC1 | Homo sapiens gap junction protein, gamma 1, 45kDa (GJC1), transcript variant 1, mRNA [NM_005497] | 1.35763 | 1.343645 | 0.013986 | 0.888223 |
| PLXNA4 | Homo sapiens plexin A4 (PLXNA4), transcript variant 1, mRNA [NM_020911] | 1.508005 | 1.49365 | 0.014355 | 0.943986 |
| PLXNA1 | Homo sapiens plexin A1 (PLXNA1), mRNA [NM_032242] | 1.346415 | 1.331765 | 0.01465 | 0.888223 |
| NTNG1 | Homo sapiens netrin G1 (NTNG1), transcript variant 2, mRNA [NM_001113228] | 1.369482 | 1.354694 | 0.014788 | 0.968147 |
| PTPRC | Homo sapiens protein tyrosine phosphatase, receptor type, C (PTPRC), transcript variant 4, mRNA [NM_080923] | 1.347182 | 1.332065 | 0.015116 | 0.888223 |
| CCR4 | Homo sapiens chemokine (C-C motif) receptor 4 (CCR4), mRNA [NM_005508] | 1.523912 | 1.508591 | 0.015321 | 0.927473 |
| PLA2G4A | Homo sapiens phospholipase A2, group IVA (cytosolic, calcium-dependent) (PLA2G4A), mRNA [NM_024420] | 1.598198 | 1.582703 | 0.015495 | 0.984844 |
| MLPH | Homo sapiens melanophilin (MLPH), transcript variant 1, mRNA [NM_024101] | 1.452821 | 1.437239 | 0.015582 | 0.894442 |
| TGFB1 | Homo sapiens transforming growth factor, beta 1 (TGFB1), mRNA [NM_000660] | 1.434748 | 1.419104 | 0.015644 | 0.888223 |
| TRIM32 | Homo sapiens tripartite motif containing 32 (TRIM32), transcript variant 1, mRNA [NM_012210] | 1.439847 | 1.424182 | 0.015665 | 0.888223 |
| GNGT1 | Homo sapiens guanine nucleotide binding protein (G protein), gamma transducing activity polypeptide 1 (GNGT1), mRNA [NM_021955] | 1.399486 | 1.38356 | 0.015926 | 0.914216 |
| GDNF | Homo sapiens glial cell derived neurotrophic factor (GDNF), transcript variant 1, mRNA [NM_000514] | 1.452253 | 1.436204 | 0.016049 | 0.888241 |
| NCKAP1L | Homo sapiens NCK-associated protein 1-like (NCKAP1L), transcript variant 1, mRNA [NM_005337] | 1.442139 | 1.426074 | 0.016066 | 0.888223 |
| ATP7A | Homo sapiens ATPase, Cu++ transporting, alpha polypeptide (ATP7A), mRNA [NM_000052] | 1.342619 | 1.326325 | 0.016294 | 0.888223 |
| CTNNA1 | Homo sapiens catenin (cadherin-associated protein), alpha 1, 102kDa (CTNNA1), mRNA [NM_001903] | 1.47608 | 1.45955 | 0.01653 | 0.956328 |
| IRF1 | Homo sapiens interferon regulatory factor 1 (IRF1), mRNA [NM_002198] | 1.356296 | 1.339462 | 0.016835 | 0.888223 |
| TFAP2A | Homo sapiens transcription factor AP-2 alpha (activating enhancer binding protein 2 alpha) (TFAP2A), transcript variant 2, mRNA [NM_001032280] | 1.35389 | 1.337031 | 0.016859 | 0.888223 |
| ADCYAP1R1 | Homo sapiens adenylate cyclase activating polypeptide 1 (pituitary) receptor type I (ADCYAP1R1), transcript variant 3, mRNA [NM_001118] | 1.51633 | 1.499431 | 0.016899 | 0.929071 |
| FOXE1 | Homo sapiens forkhead box E1 (thyroid transcription factor 2) (FOXE1), mRNA [NM_004473] | 1.504084 | 1.486781 | 0.017303 | 0.939445 |
| TCF7L2 | Homo sapiens transcription factor 7-like 2 (T-cell specific, HMG-box) (TCF7L2), transcript variant 2, mRNA [NM_030756] | 1.551239 | 1.533767 | 0.017472 | 0.904957 |
| NOTCH1 | Homo sapiens notch 1 (NOTCH1), mRNA [NM_017617] | 1.390082 | 1.372536 | 0.017546 | 0.888223 |
| EP300 | Homo sapiens E1A binding protein p300 (EP300), mRNA [NM_001429] | 1.350136 | 1.332078 | 0.018058 | 0.888223 |
| AHR | Homo sapiens aryl hydrocarbon receptor (AHR), mRNA [NM_001621] | 1.521295 | 1.503174 | 0.018121 | 0.91362 |
| CEBPA | Homo sapiens CCAAT/enhancer binding protein (C/EBP), alpha (CEBPA), mRNA [NM_004364] | 1.360595 | 1.342464 | 0.01813 | 0.888223 |
| RAB27A | Homo sapiens RAB27A, member RAS oncogene family (RAB27A), transcript variant 1, mRNA [NM_004580] | 1.536737 | 1.518539 | 0.018199 | 0.902035 |
| COL3A1 | Homo sapiens collagen, type III, alpha 1 (COL3A1), mRNA [NM_000090] | 1.350244 | 1.331959 | 0.018285 | 0.888223 |
| PMP22 | Homo sapiens peripheral myelin protein 22 (PMP22), transcript variant 1, mRNA [NM_000304] | 1.443908 | 1.425023 | 0.018885 | 0.888223 |
| TLN1 | Homo sapiens talin 1 (TLN1), mRNA [NM_006289] | 1.38317 | 1.364245 | 0.018925 | 0.888223 |
| PCSK9 | Homo sapiens proprotein convertase subtilisin/kexin type 9 (PCSK9), mRNA [NM_174936] | 1.623284 | 1.604354 | 0.01893 | 0.991086 |
| DNAJC19 | Homo sapiens DnaJ (Hsp40) homolog, subfamily C, member 19 (DNAJC19), transcript variant 1, mRNA [NM_145261] | 1.454637 | 1.435594 | 0.019043 | 0.888223 |
| H1FNT | Homo sapiens H1 histone family, member N, testis-specific (H1FNT), mRNA [NM_181788] | 2.587094 | 2.567906 | 0.019188 | 0.993173 |
| MMP19 | Homo sapiens matrix metallopeptidase 19 (MMP19), transcript variant 1, mRNA [NM_002429] | 1.352854 | 1.333663 | 0.019191 | 0.888223 |
| PRKG1 | full-length cDNA clone CS0DB006YH07 of Neuroblastoma Cot 10-normalized of Homo sapiens (human) [CR603327] | 1.357712 | 1.338517 | 0.019195 | 0.888223 |
| LRP4 | Homo sapiens low density lipoprotein receptor-related protein 4 (LRP4), mRNA [NM_002334] | 1.842428 | 1.823112 | 0.019315 | 0.993886 |
| MYO7A | Homo sapiens myosin VIIA (MYO7A), transcript variant 1, mRNA [NM_000260] | 1.527741 | 1.508314 | 0.019427 | 0.914946 |
| VGF | Homo sapiens VGF nerve growth factor inducible (VGF), mRNA [NM_003378] | 1.497971 | 1.478539 | 0.019431 | 0.940216 |
| ARID5B | Homo sapiens AT rich interactive domain 5B (MRF1-like) (ARID5B), transcript variant 1, mRNA [NM_032199] | 1.404054 | 1.384444 | 0.019611 | 0.888223 |
| A_33_P3288609 | p21 protein (Cdc42/Rac)-activated kinase 1 [Source:HGNC Symbol;Acc:8590] [ENST00000526968] | 1.442111 | 1.422202 | 0.019909 | 0.912033 |
| PRDM12 | Homo sapiens PR domain containing 12 (PRDM12), mRNA [NM_021619] | 1.450866 | 1.430806 | 0.02006 | 0.913853 |
| CDK5R1 | Homo sapiens cyclin-dependent kinase 5, regulatory subunit 1 (p35) (CDK5R1), mRNA [NM_003885] | 1.358444 | 1.33834 | 0.020104 | 0.888223 |
| CCNB1 | Homo sapiens cyclin B1 (CCNB1), mRNA [NM_031966] | 2.50558 | 2.485436 | 0.020144 | 0.992944 |
| CHN1 | Homo sapiens chimerin (chimaerin) 1 (CHN1), transcript variant 1, mRNA [NM_001822] | 1.471134 | 1.450904 | 0.02023 | 0.930477 |
| XIRP1 | Homo sapiens xin actin-binding repeat containing 1 (XIRP1), transcript variant 1, mRNA [NM_194293] | 1.364375 | 1.344033 | 0.020343 | 0.943795 |
| LHX5 | Homo sapiens LIM homeobox 5 (LHX5), mRNA [NM_022363] | 1.780943 | 1.760411 | 0.020531 | 0.992356 |
| COL6A2 | Homo sapiens collagen, type VI, alpha 2 (COL6A2), transcript variant 2C2a, mRNA [NM_058174] | 1.450733 | 1.430036 | 0.020698 | 0.888223 |
| DBN1 | Homo sapiens drebrin 1 (DBN1), transcript variant 2, mRNA [NM_080881] | 1.485574 | 1.464457 | 0.021117 | 0.941767 |
| IL25 | Homo sapiens interleukin 25 (IL25), transcript variant 1, mRNA [NM_022789] | 1.407054 | 1.385919 | 0.021134 | 0.888223 |
| NPTX1 | Homo sapiens neuronal pentraxin I (NPTX1), mRNA [NM_002522] | 1.562416 | 1.541003 | 0.021414 | 0.89938 |
| TGFBR1 | Homo sapiens transforming growth factor, beta receptor 1 (TGFBR1), transcript variant 1, mRNA [NM_004612] | 1.387591 | 1.365424 | 0.022167 | 0.888223 |
| ERBB4 | Homo sapiens v-erb-a erythroblastic leukemia viral oncogene homolog 4 (avian) (ERBB4), transcript variant JM-a/CVT-1, mRNA [NM_005235] | 1.789286 | 1.766886 | 0.0224 | 0.992422 |
| MSR1 | Homo sapiens macrophage scavenger receptor 1 (MSR1), transcript variant SR-AII, mRNA [NM_002445] | 1.42391 | 1.401033 | 0.022876 | 0.888223 |
| NPHP1 | Homo sapiens nephronophthisis 1 (juvenile) (NPHP1), transcript variant 1, mRNA [NM_000272] | 1.332212 | 1.309235 | 0.022977 | 0.888223 |
| LYN | Homo sapiens v-yes-1 Yamaguchi sarcoma viral related oncogene homolog (LYN), transcript variant 1, mRNA [NM_002350] | 1.416787 | 1.39374 | 0.023047 | 0.888223 |
| LZTS1 | Homo sapiens leucine zipper, putative tumor suppressor 1 (LZTS1), mRNA [NM_021020] | 1.357069 | 1.333916 | 0.023153 | 0.888223 |
| RSPO2 | Homo sapiens R-spondin 2 (RSPO2), mRNA [NM_178565] | 1.357337 | 1.333855 | 0.023482 | 0.888223 |
| KATNA1 | Homo sapiens katanin p60 (ATPase containing) subunit A 1 (KATNA1), transcript variant 1, mRNA [NM_007044] | 1.420238 | 1.396711 | 0.023527 | 0.888223 |
| C10orf58 | Homo sapiens chromosome 10 open reading frame 58 (C10orf58), transcript variant 4, mRNA [NM_001243780] | 1.356391 | 1.332633 | 0.023758 | 0.888223 |
| GRXCR1 | Homo sapiens glutaredoxin, cysteine rich 1 (GRXCR1), mRNA [NM_001080476] | 1.396749 | 1.372674 | 0.024075 | 0.888223 |
| SLIT2 | Homo sapiens slit homolog 2 (Drosophila) (SLIT2), mRNA [NM_004787] | 1.521091 | 1.496956 | 0.024135 | 0.912409 |
| NCOA3 | Homo sapiens nuclear receptor coactivator 3 (NCOA3), transcript variant 1, mRNA [NM_181659] | 1.400319 | 1.37598 | 0.024339 | 0.888223 |
| ROCK1 | Homo sapiens Rho-associated, coiled-coil containing protein kinase 1 (ROCK1), mRNA [NM_005406] | 1.53294 | 1.5086 | 0.02434 | 0.894891 |
| ADA | Homo sapiens adenosine deaminase (ADA), mRNA [NM_000022] | 1.523708 | 1.499115 | 0.024593 | 0.984808 |
| THC2499666 | ARP3_BOVIN (P61157) Actin-like protein 3 (Actin-related protein 3) (Actin-2), partial (58%) [THC2499666] | 1.701128 | 1.676447 | 0.024682 | 0.990205 |
| NHEJ1 | Homo sapiens nonhomologous end-joining factor 1 (NHEJ1), mRNA [NM_024782] | 1.360011 | 1.335265 | 0.024747 | 0.888223 |
| RORB | Homo sapiens RAR-related orphan receptor B (RORB), mRNA [NM_006914] | 2.406581 | 2.381774 | 0.024808 | 0.994142 |
| NEURL2 | Homo sapiens neuralized homolog 2 (Drosophila) (NEURL2), mRNA [NM_080749] | 1.460733 | 1.435483 | 0.02525 | 0.978128 |
| FGF9 | Homo sapiens fibroblast growth factor 9 (glia-activating factor) (FGF9), mRNA [NM_002010] | 1.464088 | 1.438732 | 0.025357 | 0.925505 |
| ENST00000349937 | transcription factor 7-like 2 (T-cell specific, HMG-box) [Source:HGNC Symbol;Acc:11641] [ENST00000349937] | 5.15441 | 5.128923 | 0.025487 | 0.99225 |
| AFP | Homo sapiens alpha-fetoprotein (AFP), mRNA [NM_001134] | 1.433785 | 1.408096 | 0.025689 | 0.888223 |
| PRDX3 | Homo sapiens peroxiredoxin 3 (PRDX3), nuclear gene encoding mitochondrial protein, transcript variant 1, mRNA [NM_006793] | 1.776339 | 1.750647 | 0.025693 | 0.991397 |
| GDF15 | Homo sapiens growth differentiation factor 15 (GDF15), mRNA [NM_004864] | 6.23131 | 6.204955 | 0.026355 | 0.990972 |
| FRZB | Homo sapiens frizzled-related protein (FRZB), mRNA [NM_001463] | 1.447655 | 1.421099 | 0.026555 | 0.888223 |
| DYX1C1 | Homo sapiens dyslexia susceptibility 1 candidate 1 (DYX1C1), transcript variant 3, mRNA [NM_001033560] | 1.355164 | 1.328593 | 0.026572 | 0.888223 |
| CBX2 | Homo sapiens chromobox homolog 2 (CBX2), transcript variant 2, mRNA [NM_032647] | 3.641848 | 3.615088 | 0.02676 | 0.991374 |
| PDE4D | phosphodiesterase 4D, cAMP-specific [Source:HGNC Symbol;Acc:8783] [ENST00000509355] | 1.35619 | 1.329167 | 0.027023 | 0.888223 |
| TUBB2A | Homo sapiens tubulin, beta 2A class IIa (TUBB2A), mRNA [NM_001069] | 1.364075 | 1.336847 | 0.027227 | 0.888223 |
| PML | Homo sapiens promyelocytic leukemia (PML), transcript variant 8, mRNA [NM_033247] | 1.529075 | 1.501722 | 0.027354 | 0.972852 |
| OMG | Homo sapiens oligodendrocyte myelin glycoprotein (OMG), mRNA [NM_002544] | 1.367202 | 1.339836 | 0.027367 | 0.888223 |
| ZCCHC11 | Homo sapiens zinc finger, CCHC domain containing 11, mRNA (cDNA clone IMAGE:5505348), with apparent retained intron. [BC048301] | 2.257312 | 2.229898 | 0.027414 | 0.990582 |
| ENST00000372179 | netrin G2 [Source:HGNC Symbol;Acc:14288] [ENST00000372179] | 2.297014 | 2.269395 | 0.027619 | 0.994557 |
| GDF9 | Homo sapiens growth differentiation factor 9 (GDF9), mRNA [NM_005260] | 1.383855 | 1.356047 | 0.027809 | 0.888223 |
| BRCA1 | Homo sapiens breast cancer 1, early onset (BRCA1), transcript variant 2, mRNA [NM_007300] | 1.431136 | 1.403177 | 0.027959 | 0.888223 |
| CALR | Homo sapiens calreticulin (CALR), mRNA [NM_004343] | 2.339834 | 2.311172 | 0.028662 | 0.988266 |
| FEZ2 | Homo sapiens fasciculation and elongation protein zeta 2 (zygin II) (FEZ2), transcript variant 2, mRNA [NM_001042548] | 1.478706 | 1.450012 | 0.028694 | 0.888223 |
| DPY19L2 | Homo sapiens dpy-19-like 2 (C. elegans) (DPY19L2), mRNA [NM_173812] | 1.446925 | 1.418224 | 0.028701 | 0.888223 |
| NCOR2 | Homo sapiens nuclear receptor corepressor 2 (NCOR2), transcript variant 1, mRNA [NM_006312] | 3.906993 | 3.878261 | 0.028731 | 0.994893 |
| FANCA | Homo sapiens Fanconi anemia, complementation group A (FANCA), transcript variant 2, mRNA [NM_001018112] | 1.400009 | 1.371132 | 0.028877 | 0.888223 |
| ROBO2 | Homo sapiens roundabout, axon guidance receptor, homolog 2 (Drosophila) (ROBO2), transcript variant 2, mRNA [NM_002942] | 1.673982 | 1.644486 | 0.029496 | 0.989698 |
| TCFL5 | Homo sapiens transcription factor-like 5 (basic helix-loop-helix) (TCFL5), mRNA [NM_006602] | 2.987158 | 2.957293 | 0.029865 | 0.990183 |
| ALKBH1 | Homo sapiens alkB, alkylation repair homolog 1 (E. coli) (ALKBH1), mRNA [NM_006020] | 1.551119 | 1.521168 | 0.029951 | 0.924816 |
| ESR1 | Homo sapiens estrogen receptor 1 (ESR1), transcript variant 4, mRNA [NM_001122742] | 1.405155 | 1.375141 | 0.030014 | 0.946291 |
| DDX25 | Homo sapiens DEAD (Asp-Glu-Ala-Asp) box polypeptide 25 (DDX25), mRNA [NM_013264] | 1.447617 | 1.417423 | 0.030195 | 0.96846 |
| MAML1 | Homo sapiens mastermind-like 1 (Drosophila) (MAML1), mRNA [NM_014757] | 1.491011 | 1.46063 | 0.030381 | 0.930259 |
| VAX1 | Homo sapiens ventral anterior homeobox 1 (VAX1), transcript variant 2, mRNA [NM_199131] | 1.525907 | 1.495513 | 0.030395 | 0.90773 |
| CDK5RAP1 | Homo sapiens CDK5 regulatory subunit associated protein 1 (CDK5RAP1), transcript variant 2, mRNA [NM_016082] | 1.538764 | 1.507992 | 0.030772 | 0.888223 |
| CXCL12 | Homo sapiens chemokine (C-X-C motif) ligand 12 (CXCL12), transcript variant 1, mRNA [NM_199168] | 1.422604 | 1.391195 | 0.031409 | 0.888223 |
| GBX2 | Homo sapiens gastrulation brain homeobox 2 (GBX2), mRNA [NM_001485] | 3.959027 | 3.927537 | 0.03149 | 0.990699 |
| SMAP1 | Homo sapiens small ArfGAP 1 (SMAP1), transcript variant 1, mRNA [NM_001044305] | 1.856597 | 1.824458 | 0.03214 | 0.986232 |
| CACNG2 | Homo sapiens calcium channel, voltage-dependent, gamma subunit 2 (CACNG2), mRNA [NM_006078] | 1.403066 | 1.369976 | 0.03309 | 0.888223 |
| IL2RA | Homo sapiens interleukin 2 receptor, alpha (IL2RA), mRNA [NM_000417] | 1.528008 | 1.494746 | 0.033262 | 0.891184 |
| ENST00000294649 | netrin G1 [Source:HGNC Symbol;Acc:23319] [ENST00000294649] | 1.549635 | 1.515868 | 0.033767 | 0.888223 |
| EFEMP1 | Homo sapiens EGF containing fibulin-like extracellular matrix protein 1 (EFEMP1), transcript variant 2, mRNA [NM_001039348] | 1.679993 | 1.646134 | 0.033859 | 0.984844 |
| IGF1 | Homo sapiens insulin-like growth factor 1 (somatomedin C) (IGF1), transcript variant 4, mRNA [NM_000618] | 1.457248 | 1.422875 | 0.034372 | 0.888223 |
| NBN | Homo sapiens nibrin (NBN), mRNA [NM_002485] | 1.425476 | 1.390988 | 0.034489 | 0.888223 |
| TRIO | Homo sapiens triple functional domain (PTPRF interacting) (TRIO), mRNA [NM_007118] | 1.584187 | 1.549589 | 0.034598 | 0.897441 |
| BNIP2 | Homo sapiens BCL2/adenovirus E1B 19kDa interacting protein 2 (BNIP2), mRNA [NM_004330] | 1.424518 | 1.389363 | 0.035155 | 0.888223 |
| FABP4 | Homo sapiens fatty acid binding protein 4, adipocyte (FABP4), mRNA [NM_001442] | 1.349964 | 1.314651 | 0.035313 | 0.888223 |
| ELF5 | Homo sapiens E74-like factor 5 (ets domain transcription factor) (ELF5), transcript variant 2, mRNA [NM_001422] | 1.391636 | 1.356122 | 0.035513 | 0.888223 |
| PVRL2 | Homo sapiens poliovirus receptor-related 2 (herpesvirus entry mediator B) (PVRL2), transcript variant alpha, mRNA [NM_002856] | 1.371683 | 1.336158 | 0.035524 | 0.888223 |
| ADAM12 | Homo sapiens ADAM metallopeptidase domain 12 (ADAM12), transcript variant 2, mRNA [NM_021641] | 3.434558 | 3.398854 | 0.035704 | 0.987798 |
| NFASC | Homo sapiens neurofascin (NFASC), transcript variant 5, mRNA [NM_001005389] | 2.828027 | 2.792149 | 0.035877 | 0.990842 |
| BDNF | Homo sapiens brain-derived neurotrophic factor (BDNF), transcript variant 1, mRNA [NM_170735] | 1.3578 | 1.321804 | 0.035996 | 0.888223 |
| OCA2 | Homo sapiens oculocutaneous albinism II (OCA2), mRNA [NM_000275] | 1.424412 | 1.388293 | 0.036119 | 0.888223 |
| JAG2 | Homo sapiens jagged 2 (JAG2), transcript variant 1, mRNA [NM_002226] | 1.405501 | 1.369297 | 0.036204 | 0.888223 |
| KAL1 | Homo sapiens Kallmann syndrome 1 sequence (KAL1), mRNA [NM_000216] | 1.369517 | 1.333252 | 0.036265 | 0.888223 |
| CTBP1 | Homo sapiens C-terminal binding protein 1 (CTBP1), transcript variant 2, mRNA [NM_001012614] | 1.391657 | 1.355108 | 0.036549 | 0.888223 |
| HDAC11 | Homo sapiens histone deacetylase 11 (HDAC11), transcript variant 1, mRNA [NM_024827] | 1.323181 | 1.286517 | 0.036664 | 0.888223 |
| NOX4 | Homo sapiens NADPH oxidase 4 (NOX4), transcript variant 1, mRNA [NM_016931] | 1.46218 | 1.424859 | 0.037322 | 0.909953 |
| PIP5K1C | Homo sapiens phosphatidylinositol-4-phosphate 5-kinase, type I, gamma (PIP5K1C), transcript variant 2, mRNA [NM_012398] | 1.659956 | 1.622335 | 0.037621 | 0.985263 |
| NUMBL | Homo sapiens numb homolog (Drosophila)-like (NUMBL), mRNA [NM_004756] | 1.376795 | 1.338628 | 0.038167 | 0.888223 |
| UNC5C | Homo sapiens unc-5 homolog C (C. elegans) (UNC5C), mRNA [NM_003728] | 2.000968 | 1.962368 | 0.0386 | 0.974655 |
| DLG3 | Homo sapiens discs, large homolog 3 (Drosophila) (DLG3), transcript variant 1, mRNA [NM_021120] | 1.54737 | 1.508631 | 0.038739 | 0.888223 |
| UBE3A | ubiquitin protein ligase E3A [Source:HGNC Symbol;Acc:12496] [ENST00000428984] | 1.565116 | 1.526339 | 0.038776 | 0.888223 |
| HLA-DMA | Homo sapiens major histocompatibility complex, class II, DM alpha (HLA-DMA), mRNA [NM_006120] | 1.980724 | 1.941749 | 0.038975 | 0.991067 |
| SMAD9 | Homo sapiens SMAD family member 9 (SMAD9), transcript variant a, mRNA [NM_001127217] | 1.384537 | 1.345154 | 0.039383 | 0.888223 |
| ETV1 | Homo sapiens ets variant 1 (ETV1), transcript variant 1, mRNA [NM_004956] | 1.362359 | 1.322571 | 0.039788 | 0.888223 |
| MYPN | Homo sapiens myopalladin (MYPN), mRNA [NM_032578] | 1.458202 | 1.417994 | 0.040208 | 0.888223 |
| SOX6 | Homo sapiens SRY (sex determining region Y)-box 6 (SOX6), transcript variant 1, mRNA [NM_017508] | 1.907526 | 1.867129 | 0.040397 | 0.984844 |
| IL23R | Homo sapiens interleukin 23 receptor (IL23R), mRNA [NM_144701] | 2.221014 | 2.180356 | 0.040657 | 0.988578 |
| CLIC5 | Homo sapiens chloride intracellular channel 5 (CLIC5), nuclear gene encoding mitochondrial protein, transcript variant 2, mRNA [NM_016929] | 1.471282 | 1.429945 | 0.041337 | 0.888223 |
| GJB2 | Homo sapiens gap junction protein, beta 2, 26kDa (GJB2), mRNA [NM_004004] | 1.442891 | 1.401462 | 0.041429 | 0.888223 |
| LMX1B | Homo sapiens LIM homeobox transcription factor 1, beta (LMX1B), transcript variant 1, mRNA [NM_002316] | 3.253881 | 3.212113 | 0.041767 | 0.989442 |
| RPS19 | Homo sapiens ribosomal protein S19 (RPS19), mRNA [NM_001022] | 1.445282 | 1.403492 | 0.041789 | 0.888223 |
| FGF8 | Homo sapiens fibroblast growth factor 8 (androgen-induced) (FGF8), transcript variant F, mRNA [NM_033163] | 2.284818 | 2.242884 | 0.041934 | 0.979887 |
| PTEN | Homo sapiens phosphatase and tensin homolog (PTEN), mRNA [NM_000314] | 1.565789 | 1.523786 | 0.042003 | 0.888223 |
| EGR1 | Homo sapiens early growth response 1 (EGR1), mRNA [NM_001964] | 1.441596 | 1.399512 | 0.042084 | 0.888223 |
| TOB2 | Homo sapiens transducer of ERBB2, 2 (TOB2), mRNA [NM_016272] | 1.402998 | 1.360893 | 0.042105 | 0.888223 |
| CDKN1C | Homo sapiens cyclin-dependent kinase inhibitor 1C (p57, Kip2) (CDKN1C), transcript variant 1, mRNA [NM_000076] | 1.452598 | 1.409191 | 0.043407 | 0.934134 |
| TCF7 | Homo sapiens transcription factor 7 (T-cell specific, HMG-box) (TCF7), transcript variant 1, mRNA [NM_003202] | 1.496501 | 1.452398 | 0.044103 | 0.888223 |
| FIG4 | Homo sapiens FIG4 homolog, SAC1 lipid phosphatase domain containing (S. cerevisiae) (FIG4), mRNA [NM_014845] | 1.486887 | 1.442362 | 0.044525 | 0.888223 |
| PLA2G10 | Homo sapiens phospholipase A2, group X (PLA2G10), mRNA [NM_003561] | 3.657025 | 3.611279 | 0.045746 | 0.988266 |
| ARHGEF1 | Homo sapiens Rho guanine nucleotide exchange factor (GEF) 1 (ARHGEF1), transcript variant 1, mRNA [NM_199002] | 1.51072 | 1.464905 | 0.045815 | 0.888223 |
| ID2 | Homo sapiens inhibitor of DNA binding 2, dominant negative helix-loop-helix protein (ID2), mRNA [NM_002166] | 1.825963 | 1.779703 | 0.04626 | 0.98748 |
| PRKDC | Homo sapiens protein kinase, DNA-activated, catalytic polypeptide (PRKDC), transcript variant 1, mRNA [NM_006904] | 1.802596 | 1.756303 | 0.046293 | 0.988432 |
| ZEB2 | Homo sapiens zinc finger E-box binding homeobox 2 (ZEB2), transcript variant 1, mRNA [NM_014795] | 2.988091 | 2.941658 | 0.046434 | 0.988432 |
| DSCAM | Homo sapiens Down syndrome cell adhesion molecule (DSCAM), transcript variant 1, mRNA [NM_001389] | 1.635239 | 1.588759 | 0.04648 | 0.973794 |
| SOX5 | Homo sapiens SRY (sex determining region Y)-box 5 (SOX5), transcript variant 2, mRNA [NM_152989] | 1.598512 | 1.551939 | 0.046573 | 0.889644 |
| SOX4 | Homo sapiens SRY (sex determining region Y)-box 4 (SOX4), mRNA [NM_003107] | 1.582315 | 1.535545 | 0.04677 | 0.915089 |
| IRF6 | Homo sapiens interferon regulatory factor 6 (IRF6), transcript variant 1, mRNA [NM_006147] | 1.477278 | 1.430158 | 0.04712 | 0.888223 |
| GNAT2 | Homo sapiens guanine nucleotide binding protein (G protein), alpha transducing activity polypeptide 2 (GNAT2), mRNA [NM_005272] | 1.475584 | 1.428287 | 0.047297 | 0.888223 |
| CAV2 | Homo sapiens caveolin 2 (CAV2), transcript variant 1, mRNA [NM_001233] | 1.573637 | 1.526339 | 0.047298 | 0.888223 |
| LOC440983 | WW domain containing transcription regulator 1 [Source:HGNC Symbol;Acc:24042] [ENST00000474080] | 1.598999 | 1.551679 | 0.047321 | 0.973794 |
| CHRD | Homo sapiens chordin (CHRD), mRNA [NM_003741] | 2.913045 | 2.865563 | 0.047482 | 0.989695 |
| DCLRE1C | Homo sapiens DNA cross-link repair 1C (DCLRE1C), transcript variant c, mRNA [NM_001033858] | 1.408971 | 1.360878 | 0.048093 | 0.888223 |
| SLITRK3 | Homo sapiens SLIT and NTRK-like family, member 3 (SLITRK3), mRNA [NM_014926] | 1.424144 | 1.375334 | 0.048811 | 0.888223 |
| CASP8 | Homo sapiens caspase 8, apoptosis-related cysteine peptidase (CASP8), transcript variant E, mRNA [NM_033358] | 1.408598 | 1.359765 | 0.048833 | 0.888223 |
| TLX1 | Homo sapiens T-cell leukemia homeobox 1 (TLX1), transcript variant 1, mRNA [NM_005521] | 1.961488 | 1.912498 | 0.048989 | 0.98824 |
| NME5 | Homo sapiens non-metastatic cells 5, protein expressed in (nucleoside-diphosphate kinase) (NME5), mRNA [NM_003551] | 1.487877 | 1.438785 | 0.049092 | 0.888223 |
| CD3D | Homo sapiens CD3d molecule, delta (CD3-TCR complex) (CD3D), transcript variant 1, mRNA [NM_000732] | 1.402563 | 1.353463 | 0.0491 | 0.922215 |
| NR1H2 | Homo sapiens nuclear receptor subfamily 1, group H, member 2 (NR1H2), mRNA [NM_007121] | 1.46012 | 1.410188 | 0.049932 | 0.888223 |
| MLL5 | Homo sapiens myeloid/lymphoid or mixed-lineage leukemia 5 (trithorax homolog, Drosophila) (MLL5), transcript variant 1, mRNA [NM_182931] | 1.699418 | 1.649157 | 0.05026 | 0.977447 |
| NF1 | Homo sapiens neurofibromin 1 (NF1), transcript variant 2, mRNA [NM_000267] | 1.561116 | 1.510113 | 0.051003 | 0.888223 |
| ITGA4 | Homo sapiens integrin, alpha 4 (antigen CD49D, alpha 4 subunit of VLA-4 receptor) (ITGA4), mRNA [NM_000885] | 1.468762 | 1.417499 | 0.051263 | 0.888223 |
| CRYGB | Homo sapiens crystallin, gamma B (CRYGB), mRNA [NM_005210] | 1.569397 | 1.516767 | 0.05263 | 0.888223 |
| C12orf57 | Homo sapiens atrophin 1 (ATN1), transcript variant 1, mRNA [NM_001007026] | 3.221908 | 3.16784 | 0.054067 | 0.984563 |
| GSX1 | Homo sapiens GS homeobox 1 (GSX1), mRNA [NM_145657] | 1.938317 | 1.884195 | 0.054122 | 0.979912 |
| ARHGEF10 | Rho guanine nucleotide exchange factor (GEF) 10 [Source:HGNC Symbol;Acc:14103] [ENST00000382795] | 1.715597 | 1.661329 | 0.054268 | 0.97282 |
| CCL2 | Homo sapiens chemokine (C-C motif) ligand 2 (CCL2), mRNA [NM_002982] | 1.472854 | 1.417875 | 0.05498 | 0.888223 |
| ATF2 | Homo sapiens activating transcription factor 2 (ATF2), mRNA [NM_001880] | 1.472108 | 1.416171 | 0.055937 | 0.888223 |
| BMI1 | Homo sapiens BMI1 polycomb ring finger oncogene (BMI1), mRNA [NM_005180] | 1.413342 | 1.357325 | 0.056017 | 0.888223 |
| MAPT | Homo sapiens microtubule-associated protein tau (MAPT), transcript variant 6, mRNA [NM_001123066] | 3.050231 | 2.994077 | 0.056154 | 0.984785 |
| THOC5 | Homo sapiens THO complex 5 (THOC5), transcript variant 1, mRNA [NM_001002878] | 1.931665 | 1.875473 | 0.056192 | 0.985557 |
| COL1A2 | Homo sapiens collagen, type I, alpha 2 (COL1A2), mRNA [NM_000089] | 1.480177 | 1.423309 | 0.056868 | 0.888223 |
| TFAP2B | Homo sapiens transcription factor AP-2 beta (activating enhancer binding protein 2 beta) (TFAP2B), mRNA [NM_003221] | 1.423962 | 1.365333 | 0.058629 | 0.888223 |
| DPYSL5 | Homo sapiens dihydropyrimidinase-like 5 (DPYSL5), mRNA [NM_020134] | 1.471342 | 1.412443 | 0.058898 | 0.888223 |
| HBZ | Homo sapiens hemoglobin, zeta (HBZ), mRNA [NM_005332] | 1.376157 | 1.316697 | 0.05946 | 0.888223 |
| TSPO | Homo sapiens translocator protein (18kDa) (TSPO), transcript variant PBR, mRNA [NM_000714] | 1.523142 | 1.463634 | 0.059508 | 0.888223 |
| ADAMTS20 | ADAM metallopeptidase with thrombospondin type 1 motif, 20 [Source:HGNC Symbol;Acc:17178] [ENST00000389417] | 1.52109 | 1.461531 | 0.059559 | 0.888223 |
| RARA | Homo sapiens retinoic acid receptor, alpha (RARA), transcript variant 2, mRNA [NM_001024809] | 6.494918 | 6.434783 | 0.060135 | 0.977487 |
| STRBP | Homo sapiens spermatid perinuclear RNA binding protein (STRBP), transcript variant 1, mRNA [NM_018387] | 1.604008 | 1.542859 | 0.061149 | 0.888223 |
| MIB1 | Homo sapiens mindbomb homolog 1 (Drosophila) (MIB1), mRNA [NM_020774] | 1.432277 | 1.370522 | 0.061755 | 0.888223 |
| MAS1 | Homo sapiens MAS1 oncogene (MAS1), mRNA [NM_002377] | 1.45906 | 1.397007 | 0.062052 | 0.888223 |
| SP7 | Homo sapiens Sp7 transcription factor (SP7), transcript variant 2, mRNA [NM_152860] | 1.740601 | 1.678303 | 0.062298 | 0.97954 |
| ENST00000338061 | phospholipase C, beta 1 (phosphoinositide-specific) [Source:HGNC Symbol;Acc:15917] [ENST00000338061] | 1.37095 | 1.30826 | 0.06269 | 0.888223 |
| UHMK1 | Homo sapiens U2AF homology motif (UHM) kinase 1 (UHMK1), transcript variant 1, mRNA [NM_175866] | 1.467598 | 1.404633 | 0.062965 | 0.888223 |
| IL23A | Homo sapiens interleukin 23, alpha subunit p19 (IL23A), mRNA [NM_016584] | 2.831216 | 2.768229 | 0.062987 | 0.964566 |
| DAB2IP | Homo sapiens DAB2 interacting protein (DAB2IP), transcript variant 2, mRNA [NM_138709] | 1.923028 | 1.859744 | 0.063284 | 0.978828 |
| GNAS | Homo sapiens GNAS complex locus (GNAS), transcript variant 2, mRNA [NM_001077490] | 2.467596 | 2.404278 | 0.063318 | 0.979652 |
| BMPR1A | Homo sapiens bone morphogenetic protein receptor, type IA (BMPR1A), mRNA [NM_004329] | 1.422772 | 1.359429 | 0.063343 | 0.888223 |
| ENST00000295663 | X-ray repair complementing defective repair in Chinese hamster cells 5 (double-strand-break rejoining) [Source:HGNC Symbol;Acc:12833] [ENST00000392133] | 1.483602 | 1.419905 | 0.063697 | 0.888223 |
| MERTK | Homo sapiens c-mer proto-oncogene tyrosine kinase (MERTK), mRNA [NM_006343] | 1.503843 | 1.439486 | 0.064357 | 0.888223 |
| SPRR2G | Homo sapiens small proline-rich protein 2G (SPRR2G), mRNA [NM_001014291] | 1.448146 | 1.383127 | 0.065019 | 0.897441 |
| MBNL1 | Homo sapiens muscleblind-like (Drosophila) (MBNL1), transcript variant 1, mRNA [NM_021038] | 1.55289 | 1.487525 | 0.065365 | 0.894194 |
| TXNIP | Homo sapiens thioredoxin interacting protein (TXNIP), mRNA [NM_006472] | 1.451748 | 1.386293 | 0.065455 | 0.888223 |
| PPARG | Homo sapiens peroxisome proliferator-activated receptor gamma (PPARG), transcript variant 3, mRNA [NM_138711] | 1.6027 | 1.535409 | 0.067291 | 0.888223 |
| DISC1 | Homo sapiens disrupted in schizophrenia 1 (DISC1), transcript variant m, mRNA [NM_001164550] | 1.503626 | 1.436294 | 0.067333 | 0.888223 |
| PPP2CA | Homo sapiens protein phosphatase 2, catalytic subunit, alpha isozyme (PPP2CA), mRNA [NM_002715] | 1.465779 | 1.398275 | 0.067504 | 0.888223 |
| SSH1 | Homo sapiens slingshot homolog 1 (Drosophila) (SSH1), transcript variant 1, mRNA [NM_018984] | 1.460793 | 1.393045 | 0.067748 | 0.915145 |
| INSIG1 | Homo sapiens insulin induced gene 1 (INSIG1), transcript variant 1, mRNA [NM_005542] | 1.526184 | 1.458302 | 0.067882 | 0.888223 |
| NFATC3 | Homo sapiens nuclear factor of activated T-cells, cytoplasmic, calcineurin-dependent 3 (NFATC3), transcript variant 1, mRNA [NM_173165] | 1.523973 | 1.45606 | 0.067913 | 0.888223 |
| ENST00000377153 | ubiquitination factor E4B [Source:HGNC Symbol;Acc:12500] [ENST00000377153] | 1.664251 | 1.596177 | 0.068074 | 0.977328 |
| MYF5 | Homo sapiens myogenic factor 5 (MYF5), mRNA [NM_005593] | 2.276693 | 2.208505 | 0.068188 | 0.97445 |
| IL15 | Homo sapiens interleukin 15 (IL15), transcript variant 2, mRNA [NM_172175] | 1.609905 | 1.541655 | 0.06825 | 0.971496 |
| TBX5 | Homo sapiens T-box 5 (TBX5), transcript variant 1, mRNA [NM_000192] | 1.587134 | 1.518739 | 0.068395 | 0.888223 |
| NPTN | Homo sapiens neuroplastin (NPTN), transcript variant b, mRNA [NM_012428] | 1.392395 | 1.323521 | 0.068874 | 0.888223 |
| GABBR1 | Homo sapiens gamma-aminobutyric acid (GABA) B receptor, 1 (GABBR1), transcript variant 1, mRNA [NM_001470] | 1.572736 | 1.503631 | 0.069105 | 0.952152 |
| PLCB1 | HSJ793N16 match: proteins: Q9Z0E5 {Homo sapiens} (exp=0; wgp=1; cg=0), partial (90%) [THC2787210] | 2.105967 | 2.036331 | 0.069635 | 0.982406 |
| YBX1 | Homo sapiens Y box binding protein 1 (YBX1), mRNA [NM_004559] | 1.735682 | 1.665841 | 0.069841 | 0.974763 |
| GJA1 | Homo sapiens gap junction protein, alpha 1, 43kDa (GJA1), mRNA [NM_000165] | 1.493815 | 1.423897 | 0.069918 | 0.89938 |
| C1QC | Homo sapiens complement component 1, q subcomponent, C chain (C1QC), transcript variant 2, mRNA [NM_172369] | 1.589768 | 1.519702 | 0.070066 | 0.888223 |
| PCM1 | Homo sapiens pericentriolar material 1 (PCM1), mRNA [NM_006197] | 3.064952 | 2.99486 | 0.070092 | 0.986707 |
| LCE2D | Homo sapiens late cornified envelope 2D (LCE2D), mRNA [NM_178430] | 1.599233 | 1.528995 | 0.070238 | 0.888223 |
| RAG2 | Homo sapiens recombination activating gene 2 (RAG2), transcript variant 4, mRNA [NM_001243786] | 1.422339 | 1.351267 | 0.071072 | 0.888223 |
| ARHGEF10 | Rho guanine nucleotide exchange factor (GEF) 10 [Source:HGNC Symbol;Acc:14103] [ENST00000398560] | 1.364939 | 1.293752 | 0.071187 | 0.888223 |
| SNRK | Homo sapiens SNF related kinase (SNRK), transcript variant 1, mRNA [NM_017719] | 1.454311 | 1.382878 | 0.071433 | 0.888223 |
| CELSR1 | Homo sapiens cadherin, EGF LAG seven-pass G-type receptor 1 (flamingo homolog, Drosophila) (CELSR1), mRNA [NM_014246] | 1.420636 | 1.349161 | 0.071475 | 0.898834 |
| ENST00000395431 | centrosomal protein 120kDa [Source:HGNC Symbol;Acc:26690] [ENST00000395431] | 1.432919 | 1.361346 | 0.071573 | 0.888223 |
| BDNF | Homo sapiens brain-derived neurotrophic factor (BDNF), transcript variant 1, mRNA [NM_170735] | 3.093067 | 3.02146 | 0.071607 | 0.988266 |
| RUNX2 | Homo sapiens runt-related transcription factor 2 (RUNX2), transcript variant 3, mRNA [NM_004348] | 2.670575 | 2.598686 | 0.071889 | 0.982548 |
| AHR | Homo sapiens aryl hydrocarbon receptor (AHR), mRNA [NM_001621] | 1.413499 | 1.339898 | 0.073601 | 0.888223 |
| SLITRK2 | Homo sapiens SLIT and NTRK-like family, member 2 (SLITRK2), transcript variant 1, mRNA [NM_032539] | 1.919416 | 1.844908 | 0.074507 | 0.973794 |
| SPOCK2 | Homo sapiens sparc/osteonectin, cwcv and kazal-like domains proteoglycan (testican) 2 (SPOCK2), transcript variant 2, mRNA [NM_014767] | 3.665213 | 3.590647 | 0.074566 | 0.985183 |
| CDC20 | Homo sapiens cell division cycle 20 homolog (S. cerevisiae) (CDC20), mRNA [NM_001255] | 3.984031 | 3.90937 | 0.074661 | 0.934395 |
| ANKRD7 | Homo sapiens ankyrin repeat domain 7 (ANKRD7), mRNA [NM_019644] | 1.62502 | 1.550311 | 0.074709 | 0.888223 |
| CHN2 | Homo sapiens chimerin (chimaerin) 2 (CHN2), transcript variant 2, mRNA [NM_004067] | 1.874874 | 1.80013 | 0.074745 | 0.981083 |
| TESC | Homo sapiens tescalcin (TESC), transcript variant 1, mRNA [NM_017899] | 3.599933 | 3.524401 | 0.075531 | 0.98443 |
| MAPK11 | Homo sapiens mitogen-activated protein kinase 11 (MAPK11), mRNA [NM_002751] | 2.951025 | 2.874479 | 0.076546 | 0.979472 |
| YWHAB | Homo sapiens tyrosine 3-monooxygenase/tryptophan 5-monooxygenase activation protein, beta polypeptide (YWHAB), transcript variant 1, mRNA [NM_003404] | 1.475771 | 1.399177 | 0.076593 | 0.888223 |
| SEMA4D | Homo sapiens sema domain, immunoglobulin domain (Ig), transmembrane domain (TM) and short cytoplasmic domain, (semaphorin) 4D (SEMA4D), transcript variant 1, mRNA [NM_006378] | 1.534603 | 1.457894 | 0.076709 | 0.892818 |
| GNAO1 | Homo sapiens guanine nucleotide binding protein (G protein), alpha activating activity polypeptide O (GNAO1), transcript variant 2, mRNA [NM_138736] | 1.624369 | 1.547462 | 0.076907 | 0.955017 |
| RPE65 | Homo sapiens retinal pigment epithelium-specific protein 65kDa (RPE65), mRNA [NM_000329] | 1.518642 | 1.441636 | 0.077006 | 0.888223 |
| CCDC22 | Homo sapiens forkhead box P3 (FOXP3), transcript variant 1, mRNA [NM_014009] | 2.913712 | 2.836703 | 0.077009 | 0.97503 |
| STRN | Homo sapiens striatin, calmodulin binding protein (STRN), mRNA [NM_003162] | 1.49469 | 1.417186 | 0.077504 | 0.888223 |
| SRGAP2 | Homo sapiens SLIT-ROBO Rho GTPase activating protein 2 (SRGAP2), transcript variant 2, mRNA [NM_001042758] | 1.940597 | 1.862864 | 0.077733 | 0.983669 |
| ERCC1 | Homo sapiens excision repair cross-complementing rodent repair deficiency, complementation group 1 (includes overlapping antisense sequence) (ERCC1), transcript variant 1, mRNA [NM_202001] | 1.565803 | 1.487489 | 0.078314 | 0.915987 |
| TRIM15 | Homo sapiens tripartite motif containing 15 (TRIM15), mRNA [NM_033229] | 1.746644 | 1.668002 | 0.078641 | 0.974655 |
| ENST00000313063 | parathyroid hormone 1 receptor [Source:HGNC Symbol;Acc:9608] [ENST00000313063] | 1.615419 | 1.536608 | 0.078811 | 0.888223 |
| UBE3A | Homo sapiens ubiquitin protein ligase E3A (UBE3A), transcript variant 3, mRNA [NM_130839] | 1.427359 | 1.347836 | 0.079523 | 0.888223 |
| ABCG1 | Homo sapiens ATP-binding cassette, sub-family G (WHITE), member 1 (ABCG1), transcript variant 5, mRNA [NM_207627] | 1.468036 | 1.386976 | 0.08106 | 0.888223 |
| XBP1 | Homo sapiens X-box binding protein 1 (XBP1), transcript variant 1, mRNA [NM_005080] | 1.535431 | 1.454096 | 0.081335 | 0.913791 |
| RXFP2 | Homo sapiens relaxin/insulin-like family peptide receptor 2 (RXFP2), transcript variant 1, mRNA [NM_130806] | 1.509268 | 1.427894 | 0.081373 | 0.888223 |
| SKI | Homo sapiens v-ski sarcoma viral oncogene homolog (avian) (SKI), mRNA [NM_003036] | 1.458288 | 1.376699 | 0.081589 | 0.888223 |
| HIPK2 | Homo sapiens homeodomain interacting protein kinase 2 (HIPK2), transcript variant 1, mRNA [NM_022740] | 1.958183 | 1.876334 | 0.081849 | 0.98359 |
| FOXD3 | Homo sapiens forkhead box D3 (FOXD3), mRNA [NM_012183] | 1.471527 | 1.389276 | 0.082251 | 0.888223 |
| REST | Homo sapiens RE1-silencing transcription factor (REST), transcript variant 1, mRNA [NM_005612] | 1.919603 | 1.836852 | 0.082751 | 0.979472 |
| ITGB3 | Homo sapiens integrin, beta 3 (platelet glycoprotein IIIa, antigen CD61) (ITGB3), mRNA [NM_000212] | 1.522416 | 1.439281 | 0.083135 | 0.888223 |
| FBXO5 | Homo sapiens F-box protein 5 (FBXO5), transcript variant 2, mRNA [NM_001142522] | 1.417019 | 1.333684 | 0.083335 | 0.888223 |
| CNGA3 | Homo sapiens cyclic nucleotide gated channel alpha 3 (CNGA3), transcript variant 1, mRNA [NM_001298] | 1.40819 | 1.324472 | 0.083718 | 0.888223 |
| BHLHE41 | Homo sapiens basic helix-loop-helix family, member e41 (BHLHE41), mRNA [NM_030762] | 1.408465 | 1.324233 | 0.084232 | 0.888223 |
| STAT5B | Homo sapiens signal transducer and activator of transcription 5B (STAT5B), mRNA [NM_012448] | 1.624699 | 1.540448 | 0.084252 | 0.946861 |
| MYADM | Homo sapiens myeloid-associated differentiation marker (MYADM), transcript variant 1, mRNA [NM_001020818] | 1.544178 | 1.459686 | 0.084492 | 0.888223 |
| MAP2 | Homo sapiens microtubule-associated protein 2 (MAP2), transcript variant 1, mRNA [NM_002374] | 1.434383 | 1.34968 | 0.084703 | 0.888223 |
| CLEC5A | Homo sapiens C-type lectin domain family 5, member A (CLEC5A), mRNA [NM_013252] | 1.392398 | 1.307351 | 0.085047 | 0.888223 |
| ABLIM2 | Homo sapiens actin binding LIM protein family, member 2 (ABLIM2), transcript variant 6, mRNA [NM_032432] | 1.487705 | 1.402576 | 0.085129 | 0.888223 |
| GNAO1 | Homo sapiens guanine nucleotide binding protein (G protein), alpha activating activity polypeptide O (GNAO1), transcript variant 1, mRNA [NM_020988] | 1.458523 | 1.373328 | 0.085195 | 0.888223 |
| FRAP1 | Homo sapiens mechanistic target of rapamycin (serine/threonine kinase) (MTOR), mRNA [NM_004958] | 1.47207 | 1.386861 | 0.085209 | 0.888223 |
| SMARCA1 | Homo sapiens SWI/SNF related, matrix associated, actin dependent regulator of chromatin, subfamily a, member 1 (SMARCA1), transcript variant 1, mRNA [NM_003069] | 4.382377 | 4.296896 | 0.085481 | 0.988266 |
| WNT3A | Homo sapiens wingless-type MMTV integration site family, member 3A (WNT3A), mRNA [NM_033131] | 4.584299 | 4.498493 | 0.085805 | 0.988432 |
| SEMA4F | Homo sapiens sema domain, immunoglobulin domain (Ig), transmembrane domain (TM) and short cytoplasmic domain, (semaphorin) 4F (SEMA4F), mRNA [NM_004263] | 1.564626 | 1.478609 | 0.086017 | 0.888223 |
| GLI3 | Homo sapiens GLI family zinc finger 3 (GLI3), mRNA [NM_000168] | 1.511261 | 1.424705 | 0.086556 | 0.888223 |
| WDR62 | Homo sapiens WD repeat domain 62 (WDR62), transcript variant 1, mRNA [NM_001083961] | 1.580901 | 1.493683 | 0.087218 | 0.888223 |
| CHRNA1 | Homo sapiens cholinergic receptor, nicotinic, alpha 1 (muscle) (CHRNA1), transcript variant 1, mRNA [NM_001039523] | 1.493718 | 1.406421 | 0.087297 | 0.888223 |
| GABRB2 | Homo sapiens gamma-aminobutyric acid (GABA) A receptor, beta 2 (GABRB2), transcript variant 1, mRNA [NM_021911] | 1.74373 | 1.655548 | 0.088182 | 0.970097 |
| LAMA5 | Homo sapiens laminin, alpha 5 (LAMA5), mRNA [NM_005560] | 3.243026 | 3.154516 | 0.08851 | 0.982853 |
| PRMT1 | Homo sapiens protein arginine methyltransferase 1 (PRMT1), transcript variant 1, mRNA [NM_001536] | 1.830317 | 1.741541 | 0.088776 | 0.965408 |
| LAMC3 | Homo sapiens laminin, gamma 3 (LAMC3), mRNA [NM_006059] | 1.416357 | 1.32743 | 0.088927 | 0.888223 |
| SLC6A4 | solute carrier family 6 (neurotransmitter transporter, serotonin), member 4 [Source:HGNC Symbol;Acc:11050] [ENST00000394821] | 1.428806 | 1.339781 | 0.089025 | 0.888223 |
| TCF4 | Homo sapiens transcription factor 4 (TCF4), transcript variant 2, mRNA [NM_003199] | 1.547567 | 1.458075 | 0.089492 | 0.937804 |
| COL4A5 | Homo sapiens collagen, type IV, alpha 5 (COL4A5), transcript variant 2, mRNA [NM_033380] | 1.41897 | 1.327867 | 0.091103 | 0.888223 |
| TNIK | Homo sapiens TRAF2 and NCK interacting kinase (TNIK), transcript variant 1, mRNA [NM_015028] | 2.545449 | 2.453738 | 0.09171 | 0.977328 |
| DLL1 | Homo sapiens delta-like 1 (Drosophila) (DLL1), mRNA [NM_005618] | 1.514719 | 1.422958 | 0.091761 | 0.888223 |
| DC392227 | ras homolog gene family, member A [Source:HGNC Symbol;Acc:667] [ENST00000265538] | 3.407132 | 3.314743 | 0.092389 | 0.978216 |
| SEP15 | Homo sapiens 15 kDa selenoprotein (SEP15), transcript variant 1, mRNA [NM_004261] | 1.528715 | 1.436253 | 0.092463 | 0.888223 |
| SLC6A4 | Homo sapiens solute carrier family 6 (neurotransmitter transporter, serotonin), member 4 (SLC6A4), mRNA [NM_001045] | 2.449193 | 2.355339 | 0.093854 | 0.969517 |
| AGTR1 | Homo sapiens angiotensin II receptor, type 1 (AGTR1), transcript variant 4, mRNA [NM_031850] | 1.471821 | 1.377304 | 0.094517 | 0.888223 |
| TCHH | Homo sapiens trichohyalin (TCHH), mRNA [NM_007113] | 1.571704 | 1.476953 | 0.09475 | 0.888223 |
| GAS7 | Homo sapiens growth arrest-specific 7 (GAS7), transcript variant c, mRNA [NM_201433] | 1.452926 | 1.358004 | 0.094922 | 0.888223 |
| CITED1 | Homo sapiens Cbp/p300-interacting transactivator, with Glu/Asp-rich carboxy-terminal domain, 1 (CITED1), transcript variant 1, mRNA [NM_004143] | 1.44685 | 1.351725 | 0.095124 | 0.888223 |
| DLX6 | Homo sapiens distal-less homeobox 6 (DLX6), mRNA [NM_005222] | 1.538344 | 1.443102 | 0.095242 | 0.891007 |
| TBX3 | Homo sapiens T-box 3 (TBX3), transcript variant 2, mRNA [NM_016569] | 6.409163 | 6.313402 | 0.095761 | 0.944912 |
| RAC3 | Homo sapiens ras-related C3 botulinum toxin substrate 3 (rho family, small GTP binding protein Rac3) (RAC3), mRNA [NM_005052] | 1.394996 | 1.298838 | 0.096159 | 0.888223 |
| SIX3 | Homo sapiens SIX homeobox 3 (SIX3), mRNA [NM_005413] | 2.896245 | 2.799864 | 0.096381 | 0.978453 |
| DST | Homo sapiens dystonin (DST), transcript variant 1e, mRNA [NM_001723] | 1.448741 | 1.352236 | 0.096505 | 0.888223 |
| SOX9 | Homo sapiens SRY (sex determining region Y)-box 9 (SOX9), mRNA [NM_000346] | 1.530067 | 1.433474 | 0.096592 | 0.888223 |
| TRAPPC4 | Homo sapiens trafficking protein particle complex 4 (TRAPPC4), mRNA [NM_016146] | 1.513964 | 1.417368 | 0.096596 | 0.888223 |
| CLASP2 | Homo sapiens cytoplasmic linker associated protein 2 (CLASP2), transcript variant 1, mRNA [NM_015097] | 4.012152 | 3.914925 | 0.097227 | 0.977137 |
| ID3 | Homo sapiens inhibitor of DNA binding 3, dominant negative helix-loop-helix protein (ID3), mRNA [NM_002167] | 1.483806 | 1.38639 | 0.097415 | 0.888223 |
| MYCBP2 | Homo sapiens MYC binding protein 2 (MYCBP2), mRNA [NM_015057] | 4.658065 | 4.560397 | 0.097668 | 0.941921 |
| CSF3 | Homo sapiens colony stimulating factor 3 (granulocyte) (CSF3), transcript variant 1, mRNA [NM_000759] | 1.508329 | 1.40993 | 0.098399 | 0.888223 |
| CTBP2 | C-terminal binding protein 2 [Source:HGNC Symbol;Acc:2495] [ENST00000530930] | 1.677583 | 1.578963 | 0.09862 | 0.932317 |
| ERCC3 | Homo sapiens excision repair cross-complementing rodent repair deficiency, complementation group 3 (xeroderma pigmentosum group B complementing) (ERCC3), mRNA [NM_000122] | 4.598423 | 4.498778 | 0.099645 | 0.961288 |
| TCF21 | Homo sapiens transcription factor 21 (TCF21), transcript variant 2, mRNA [NM_003206] | 1.391441 | 1.291792 | 0.099649 | 0.888223 |
| RBM15 | Homo sapiens RNA binding motif protein 15 (RBM15), transcript variant 1, mRNA [NM_022768] | 2.671243 | 2.57154 | 0.099703 | 0.964566 |
| GATA6 | Homo sapiens GATA binding protein 6 (GATA6), mRNA [NM_005257] | 1.561505 | 1.461704 | 0.099801 | 0.888223 |
| ENST00000440768 | IKAROS family zinc finger 1 (Ikaros) [Source:HGNC Symbol;Acc:13176] [ENST00000484847] | 1.77281 | 1.672742 | 0.100069 | 0.967391 |
| INSL3 | Homo sapiens insulin-like 3 (Leydig cell) (INSL3), mRNA [NM_005543] | 2.245931 | 2.14561 | 0.100321 | 0.965365 |
| SIX4 | Homo sapiens SIX homeobox 4 (SIX4), mRNA [NM_017420] | 1.505223 | 1.404122 | 0.101101 | 0.888223 |
| FARP2 | Homo sapiens FERM, RhoGEF and pleckstrin domain protein 2 (FARP2), mRNA [NM_014808] | 2.265666 | 2.164125 | 0.101541 | 0.978738 |
| SATB1 | Homo sapiens SATB homeobox 1 (SATB1), transcript variant 1, mRNA [NM_002971] | 1.555808 | 1.453932 | 0.101876 | 0.888223 |
| SPRR2A | Homo sapiens small proline-rich protein 2A (SPRR2A), mRNA [NM_005988] | 1.415596 | 1.313039 | 0.102557 | 0.888223 |
| FOXL2 | Homo sapiens forkhead box L2 (FOXL2), mRNA [NM_023067] | 1.512942 | 1.409897 | 0.103045 | 0.888223 |
| TPM1 | Homo sapiens tropomyosin 1 (alpha) (TPM1), transcript variant 5, mRNA [NM_000366] | 1.562746 | 1.459424 | 0.103322 | 0.888223 |
| NDN | Homo sapiens necdin homolog (mouse) (NDN), mRNA [NM_002487] | 1.465696 | 1.362335 | 0.103361 | 0.888223 |
| DNAJA3 | Homo sapiens DnaJ (Hsp40) homolog, subfamily A, member 3 (DNAJA3), nuclear gene encoding mitochondrial protein, transcript variant 1, mRNA [NM_005147] | 1.643595 | 1.539123 | 0.104473 | 0.888223 |
| PKDCC | Homo sapiens protein kinase domain containing, cytoplasmic homolog (mouse) (PKDCC), mRNA [NM_138370] | 1.470805 | 1.36633 | 0.104476 | 0.888223 |
| ZC3H8 | Homo sapiens zinc finger CCCH-type containing 8 (ZC3H8), mRNA [NM_032494] | 1.942098 | 1.837538 | 0.10456 | 0.976074 |
| KRT84 | Homo sapiens keratin 84 (KRT84), mRNA [NM_033045] | 1.538203 | 1.433594 | 0.104608 | 0.888223 |
| GATA2 | Homo sapiens GATA binding protein 2 (GATA2), transcript variant 1, mRNA [NM_001145661] | 4.151472 | 4.046833 | 0.104639 | 0.928486 |
| PSEN2 | Homo sapiens presenilin 2 (Alzheimer disease 4) (PSEN2), transcript variant 1, mRNA [NM_000447] | 2.029501 | 1.924411 | 0.10509 | 0.929377 |
| CYP19A1 | Homo sapiens cytochrome P450, family 19, subfamily A, polypeptide 1 (CYP19A1), transcript variant 2, mRNA [NM_031226] | 1.574265 | 1.469086 | 0.105178 | 0.888223 |
| CD9 | Homo sapiens CD9 molecule (CD9), mRNA [NM_001769] | 1.434897 | 1.329095 | 0.105802 | 0.888223 |
| CD74 | Homo sapiens CD74 molecule, major histocompatibility complex, class II invariant chain (CD74), transcript variant 3, mRNA [NM_001025158] | 1.524252 | 1.417369 | 0.106883 | 0.888223 |
| TIAM1 | Homo sapiens T-cell lymphoma invasion and metastasis 1 (TIAM1), mRNA [NM_003253] | 1.568933 | 1.461771 | 0.107162 | 0.888223 |
| RPS6KB1 | Homo sapiens ribosomal protein S6 kinase, 70kDa, polypeptide 1 (RPS6KB1), mRNA [NM_003161] | 1.505719 | 1.398343 | 0.107377 | 0.888223 |
| ANKS1A | ankyrin repeat and sterile alpha motif domain containing 1A [Source:HGNC Symbol;Acc:20961] [ENST00000373990] | 3.953637 | 3.845046 | 0.108591 | 0.961131 |
| CSF2 | Homo sapiens colony stimulating factor 2 (granulocyte-macrophage) (CSF2), mRNA [NM_000758] | 1.502388 | 1.39302 | 0.109368 | 0.888223 |
| MAP7 | Homo sapiens microtubule-associated protein 7 (MAP7), transcript variant 4, mRNA [NM_003980] | 1.49076 | 1.381367 | 0.109393 | 0.888223 |
| TRIO | triple functional domain (PTPRF interacting) [Source:HGNC Symbol;Acc:12303] [ENST00000541447] | 1.537506 | 1.428011 | 0.109495 | 0.888223 |
| JAK2 | Homo sapiens Janus kinase 2 (JAK2), mRNA [NM_004972] | 1.465465 | 1.355915 | 0.10955 | 0.888223 |
| HOXA7 | Homo sapiens homeobox A7 (HOXA7), mRNA [NM_006896] | 1.712625 | 1.60244 | 0.110185 | 0.946563 |
| BBS7 | Homo sapiens Bardet-Biedl syndrome 7 (BBS7), transcript variant 2, mRNA [NM_018190] | 1.409482 | 1.299205 | 0.110277 | 0.888223 |
| CSNK2A2 | Homo sapiens casein kinase 2, alpha prime polypeptide (CSNK2A2), mRNA [NM_001896] | 2.060147 | 1.949579 | 0.110568 | 0.97503 |
| TSG101 | Homo sapiens tumor susceptibility gene 101 (TSG101), mRNA [NM_006292] | 1.558909 | 1.448284 | 0.110626 | 0.888223 |
| HOXD3 | Homo sapiens homeobox D3 (HOXD3), mRNA [NM_006898] | 2.318539 | 2.207872 | 0.110666 | 0.973794 |
| PRKCQ | Homo sapiens protein kinase C, theta (PRKCQ), transcript variant 1, mRNA [NM_006257] | 1.482061 | 1.369814 | 0.112247 | 0.888223 |
| A_19_P00316105 | PREDICTED: Homo sapiens hypothetical LOC100505763 (LOC100505763), miscRNA [XR_109604] | 1.626613 | 1.513847 | 0.112766 | 0.89938 |
| MARK2 | Homo sapiens MAP/microtubule affinity-regulating kinase 2 (MARK2), transcript variant 4, mRNA [NM_001039469] | 1.548315 | 1.435287 | 0.113028 | 0.888223 |
| TNFRSF11A | Homo sapiens tumor necrosis factor receptor superfamily, member 11a, NFKB activator (TNFRSF11A), mRNA [NM_003839] | 1.541752 | 1.428309 | 0.113443 | 0.888223 |
| ALOX12 | Homo sapiens arachidonate 12-lipoxygenase (ALOX12), mRNA [NM_000697] | 1.53478 | 1.419869 | 0.114911 | 0.888223 |
| SHC1 | Homo sapiens SHC (Src homology 2 domain containing) transforming protein 1 (SHC1), transcript variant 1, mRNA [NM_183001] | 2.060567 | 1.944915 | 0.115652 | 0.949415 |
| LDB1 | Homo sapiens LIM domain binding 1 (LDB1), transcript variant 3, mRNA [NM_003893] | 2.559736 | 2.443933 | 0.115803 | 0.96669 |
| TYRP1 | Homo sapiens tyrosinase-related protein 1 (TYRP1), mRNA [NM_000550] | 1.609069 | 1.493156 | 0.115912 | 0.888223 |
| TRPC7 | Homo sapiens transient receptor potential cation channel, subfamily C, member 7 (TRPC7), transcript variant 1, mRNA [NM_020389] | 1.720908 | 1.604813 | 0.116095 | 0.930724 |
| APOLD1 | Homo sapiens apolipoprotein L domain containing 1 (APOLD1), transcript variant 2, mRNA [NM_030817] | 1.92584 | 1.808801 | 0.117039 | 0.902337 |
| ATP2B2 | ATPase, Ca++ transporting, plasma membrane 2 [Source:HGNC Symbol;Acc:815] [ENST00000342354] | 1.970594 | 1.853518 | 0.117076 | 0.973011 |
| SPINK5 | Homo sapiens serine peptidase inhibitor, Kazal type 5 (SPINK5), transcript variant 2, mRNA [NM_006846] | 1.498376 | 1.380365 | 0.118012 | 0.888223 |
| GNA11 | Homo sapiens guanine nucleotide binding protein (G protein), alpha 11 (Gq class) (GNA11), mRNA [NM_002067] | 1.555673 | 1.436732 | 0.118941 | 0.888223 |
| ETS1 | Homo sapiens v-ets erythroblastosis virus E26 oncogene homolog 1 (avian) (ETS1), transcript variant 2, mRNA [NM_005238] | 1.643121 | 1.522928 | 0.120193 | 0.894341 |
| SCEL | Homo sapiens sciellin (SCEL), transcript variant 1, mRNA [NM_144777] | 2.888205 | 2.766998 | 0.121206 | 0.975373 |
| A_33_P3260782 | recombination activating gene 2 [Source:HGNC Symbol;Acc:9832] [ENST00000534379] | 1.687385 | 1.565024 | 0.122361 | 0.918333 |
| HOXA2 | Homo sapiens homeobox A2 (HOXA2), mRNA [NM_006735] | 4.788175 | 4.665185 | 0.12299 | 0.977137 |
| RNF17 | Homo sapiens ring finger protein 17 (RNF17), transcript variant 1, mRNA [NM_031277] | 3.465996 | 3.341479 | 0.124517 | 0.977137 |
| ENST00000366699 | tripartite motif containing 11 [Source:HGNC Symbol;Acc:16281] [ENST00000366699] | 3.64758 | 3.522451 | 0.125129 | 0.970563 |
| PTCD2 | Homo sapiens pentatricopeptide repeat domain 2 (PTCD2), mRNA [NM_024754] | 1.445694 | 1.319008 | 0.126686 | 0.888223 |
| CHRNB2 | Homo sapiens cholinergic receptor, nicotinic, beta 2 (neuronal) (CHRNB2), mRNA [NM_000748] | 1.692735 | 1.565993 | 0.126743 | 0.933681 |
| IL21 | Homo sapiens interleukin 21 (IL21), transcript variant 1, mRNA [NM_021803] | 4.329099 | 4.202104 | 0.126995 | 0.941921 |
| HSD17B3 | Homo sapiens hydroxysteroid (17-beta) dehydrogenase 3 (HSD17B3), mRNA [NM_000197] | 2.348819 | 2.22107 | 0.127749 | 0.976308 |
| CYFIP1 | Homo sapiens cytoplasmic FMR1 interacting protein 1 (CYFIP1), transcript variant 1, mRNA [NM_014608] | 2.084027 | 1.955749 | 0.128278 | 0.967391 |
| DMBT1 | Homo sapiens deleted in malignant brain tumors 1 (DMBT1), transcript variant 2, mRNA [NM_007329] | 1.670898 | 1.542168 | 0.12873 | 0.888621 |
| EDN2 | Homo sapiens endothelin 2 (EDN2), mRNA [NM_001956] | 1.565665 | 1.436189 | 0.129476 | 0.888223 |
| BCL11B | Homo sapiens B-cell CLL/lymphoma 11B (zinc finger protein) (BCL11B), transcript variant 1, mRNA [NM_138576] | 1.628941 | 1.498623 | 0.130318 | 0.908164 |
| ENPP1 | Homo sapiens ectonucleotide pyrophosphatase/phosphodiesterase 1 (ENPP1), mRNA [NM_006208] | 4.311454 | 4.180935 | 0.130518 | 0.960822 |
| NRXN3 | Homo sapiens neurexin 3 (NRXN3), transcript variant 1, mRNA [NM_004796] | 1.462042 | 1.331068 | 0.130974 | 0.897899 |
| NGEF | Homo sapiens neuronal guanine nucleotide exchange factor (NGEF), transcript variant 1, mRNA [NM_019850] | 4.587451 | 4.456296 | 0.131155 | 0.888223 |
| THPO | Homo sapiens thrombopoietin (THPO), transcript variant 1, mRNA [NM_000460] | 3.192754 | 3.060927 | 0.131828 | 0.888223 |
| CUL3 | Homo sapiens cullin 3 (CUL3), mRNA [NM_003590] | 1.52447 | 1.392056 | 0.132414 | 0.888223 |
| WNT9A | Homo sapiens wingless-type MMTV integration site family, member 9A (WNT9A), mRNA [NM_003395] | 3.131217 | 2.998524 | 0.132693 | 0.977328 |
| MAMLD1 | Homo sapiens mastermind-like domain containing 1 (MAMLD1), transcript variant 1, mRNA [NM_001177465] | 1.652764 | 1.518941 | 0.133823 | 0.888223 |
| DISC1 | Homo sapiens disrupted in schizophrenia 1 (DISC1), transcript variant f, mRNA [NM_001164542] | 1.4806 | 1.3464 | 0.1342 | 0.888223 |
| CNR1 | Homo sapiens cannabinoid receptor 1 (brain) (CNR1), transcript variant 2, mRNA [NM_033181] | 1.506037 | 1.371774 | 0.134263 | 0.888223 |
| TRPC1 | Homo sapiens transient receptor potential cation channel, subfamily C, member 1 (TRPC1), transcript variant 2, mRNA [NM_003304] | 1.55246 | 1.417304 | 0.135156 | 0.888223 |
| DRD3 | Homo sapiens dopamine receptor D3 (DRD3), transcript variant e, mRNA [NM_033663] | 3.975911 | 3.840274 | 0.135637 | 0.977112 |
| AP2M1 | Homo sapiens adaptor-related protein complex 2, mu 1 subunit (AP2M1), transcript variant 1, mRNA [NM_004068] | 1.552754 | 1.41708 | 0.135674 | 0.888223 |
| BFSP1 | Homo sapiens beaded filament structural protein 1, filensin (BFSP1), transcript variant 1, mRNA [NM_001195] | 1.539485 | 1.403512 | 0.135973 | 0.888223 |
| HTRA2 | Homo sapiens HtrA serine peptidase 2 (HTRA2), nuclear gene encoding mitochondrial protein, transcript variant 2, mRNA [NM_145074] | 1.504516 | 1.368538 | 0.135978 | 0.888223 |
| IHH | Homo sapiens Indian hedgehog (IHH), mRNA [NM_002181] | 2.671613 | 2.535582 | 0.136031 | 0.959231 |
| PRKX | Homo sapiens protein kinase, X-linked (PRKX), mRNA [NM_005044] | 1.533837 | 1.397753 | 0.136084 | 0.888223 |
| CSDA | Homo sapiens cold shock domain protein A (CSDA), transcript variant 1, mRNA [NM_003651] | 4.157332 | 4.017693 | 0.139639 | 0.918853 |
| VPS72 | Homo sapiens vacuolar protein sorting 72 homolog (S. cerevisiae) (VPS72), mRNA [NM_005997] | 1.586326 | 1.446495 | 0.139831 | 0.888223 |
| PTK2B | Homo sapiens PTK2B protein tyrosine kinase 2 beta (PTK2B), transcript variant 1, mRNA [NM_173174] | 1.556759 | 1.416401 | 0.140358 | 0.892228 |
| SNTA1 | Homo sapiens syntrophin, alpha 1 (dystrophin-associated protein A1, 59kDa, acidic component) (SNTA1), mRNA [NM_003098] | 1.498198 | 1.356451 | 0.141747 | 0.888223 |
| LAMB1 | Homo sapiens laminin, beta 1 (LAMB1), mRNA [NM_002291] | 1.524808 | 1.381584 | 0.143224 | 0.888223 |
| HDAC7 | Homo sapiens histone deacetylase 7 (HDAC7), transcript variant 1, mRNA [NM_015401] | 5.211056 | 5.067743 | 0.143313 | 0.94666 |
| APC | Homo sapiens adenomatous polyposis coli (APC), transcript variant 3, mRNA [NM_000038] | 1.568086 | 1.424638 | 0.143448 | 0.888223 |
| RPL24 | Homo sapiens ribosomal protein L24 (RPL24), mRNA [NM_000986] | 2.228463 | 2.085 | 0.143463 | 0.952754 |
| CLTC | Homo sapiens clathrin, heavy chain (Hc), mRNA (cDNA clone MGC:57689 IMAGE:6187185), complete cds. [BC051800] | 1.528783 | 1.384822 | 0.143961 | 0.888223 |
| TBX20 | Homo sapiens T-box 20 (TBX20), transcript variant 2, mRNA [NM_001166220] | 1.466533 | 1.322434 | 0.1441 | 0.888223 |
| LILRB3 | Homo sapiens leukocyte immunoglobulin-like receptor, subfamily B (with TM and ITIM domains), member 3 (LILRB3), transcript variant 2, mRNA [NM_006864] | 1.919526 | 1.774998 | 0.144528 | 0.940219 |
| TGIF1 | Homo sapiens TGFB-induced factor homeobox 1 (TGIF1), transcript variant 1, mRNA [NM_170695] | 1.52366 | 1.377363 | 0.146297 | 0.888223 |
| PRKDC | Homo sapiens protein kinase, DNA-activated, catalytic polypeptide (PRKDC), transcript variant 1, mRNA [NM_006904] | 2.89664 | 2.750328 | 0.146312 | 0.972025 |
| ALCAM | Homo sapiens activated leukocyte cell adhesion molecule (ALCAM), transcript variant 1, mRNA [NM_001627] | 1.487879 | 1.341389 | 0.146491 | 0.888223 |
| ATM | Homo sapiens ataxia telangiectasia mutated (ATM), mRNA [NM_000051] | 3.438313 | 3.291501 | 0.146813 | 0.90314 |
| LPAR1 | Homo sapiens lysophosphatidic acid receptor 1 (LPAR1), transcript variant 2, mRNA [NM_057159] | 4.130295 | 3.982889 | 0.147406 | 0.959376 |
| USH2A | Homo sapiens Usher syndrome 2A (autosomal recessive, mild) (USH2A), transcript variant 1, mRNA [NM_007123] | 1.553983 | 1.406379 | 0.147604 | 0.888223 |
| ABCA1 | Homo sapiens ATP-binding cassette, sub-family A (ABC1), member 1 (ABCA1), mRNA [NM_005502] | 1.449247 | 1.301314 | 0.147933 | 0.888223 |
| UBD | Homo sapiens ubiquitin D (UBD), mRNA [NM_006398] | 6.106246 | 5.958267 | 0.147979 | 0.972357 |
| CCND1 | Homo sapiens cyclin D1 (CCND1), mRNA [NM_053056] | 1.487163 | 1.338364 | 0.148798 | 0.888223 |
| LCE5A | Homo sapiens late cornified envelope 5A (LCE5A), mRNA [NM_178438] | 1.557445 | 1.406882 | 0.150562 | 0.888223 |
| JAK3 | Homo sapiens Janus kinase 3 (JAK3), mRNA [NM_000215] | 1.861618 | 1.709773 | 0.151845 | 0.954601 |
| IGSF9 | Homo sapiens immunoglobulin superfamily, member 9 (IGSF9), transcript variant 2, mRNA [NM_020789] | 4.402168 | 4.250187 | 0.151981 | 0.970554 |
| ACSL6 | Homo sapiens acyl-CoA synthetase long-chain family member 6 (ACSL6), transcript variant 2, mRNA [NM_001009185] | 1.552386 | 1.39972 | 0.152666 | 0.888223 |
| C14orf169 | Homo sapiens chromosome 14 open reading frame 169 (C14orf169), mRNA [NM_024644] | 5.228622 | 5.075936 | 0.152686 | 0.955578 |
| ENST00000371041 | slit homolog 1 (Drosophila) [Source:HGNC Symbol;Acc:11085] [ENST00000371041] | 3.117755 | 2.96455 | 0.153205 | 0.922171 |
| DDR2 | Homo sapiens discoidin domain receptor tyrosine kinase 2 (DDR2), transcript variant 1, mRNA [NM_001014796] | 1.612768 | 1.458977 | 0.153791 | 0.888223 |
| TSHZ3 | Homo sapiens teashirt zinc finger homeobox 3 (TSHZ3), mRNA [NM_020856] | 1.603663 | 1.449276 | 0.154387 | 0.888223 |
| ROD1 | Homo sapiens ROD1 regulator of differentiation 1 (S. pombe) (ROD1), transcript variant 1, mRNA [NM_005156] | 1.647132 | 1.492394 | 0.154739 | 0.888223 |
| LGR4 | Homo sapiens leucine-rich repeat containing G protein-coupled receptor 4 (LGR4), mRNA [NM_018490] | 1.637129 | 1.48198 | 0.155149 | 0.916588 |
| TGFBR3 | Homo sapiens transforming growth factor, beta receptor III (TGFBR3), transcript variant 1, mRNA [NM_003243] | 1.662614 | 1.506616 | 0.155998 | 0.888223 |
| AFF4 | Homo sapiens AF4/FMR2 family, member 4 (AFF4), mRNA [NM_014423] | 1.592267 | 1.434883 | 0.157384 | 0.888223 |
| PLD6 | Homo sapiens phospholipase D family, member 6 (PLD6), mRNA [NM_178836] | 1.531009 | 1.373398 | 0.157611 | 0.888223 |
| MAPK11 | Homo sapiens mitogen-activated protein kinase 11 (MAPK11), mRNA [NM_002751] | 1.739659 | 1.581463 | 0.158197 | 0.942418 |
| ENST00000371958 | angiomotin [Source:HGNC Symbol;Acc:17810] [ENST00000371958] | 1.612059 | 1.453846 | 0.158213 | 0.905148 |
| ENST00000380946 | Kruppel-like factor 6 [Source:HGNC Symbol;Acc:2235] [ENST00000469435] | 1.586849 | 1.428612 | 0.158237 | 0.888223 |
| TIRAP | Homo sapiens toll-interleukin 1 receptor (TIR) domain containing adaptor protein (TIRAP), transcript variant 2, mRNA [NM_148910] | 1.863211 | 1.704844 | 0.158367 | 0.921699 |
| ARF6 | Homo sapiens ADP-ribosylation factor 6 (ARF6), mRNA [NM_001663] | 1.448745 | 1.289898 | 0.158847 | 0.888223 |
| FBXO22 | Homo sapiens F-box protein 22 (FBXO22), transcript variant 1, mRNA [NM_147188] | 1.458269 | 1.299032 | 0.159237 | 0.888223 |
| APBB1 | PREDICTED: Homo sapiens hypothetical LOC100506137 (LOC100506137), miscRNA [XR_109056] | 2.167914 | 2.008221 | 0.159693 | 0.955337 |
| CTDSP1 | Homo sapiens CTD (carboxy-terminal domain, RNA polymerase II, polypeptide A) small phosphatase 1 (CTDSP1), transcript variant 2, mRNA [NM_182642] | 3.602027 | 3.442255 | 0.159772 | 0.935544 |
| TPM1 | Homo sapiens tropomyosin 1 (alpha) (TPM1), transcript variant 1, mRNA [NM_001018005] | 1.963079 | 1.803186 | 0.159892 | 0.952152 |
| PCYT1B | Homo sapiens phosphate cytidylyltransferase 1, choline, beta (PCYT1B), transcript variant 1, mRNA [NM_004845] | 1.610105 | 1.449643 | 0.160462 | 0.888223 |
| FOXO3 | Homo sapiens forkhead box O3 (FOXO3), transcript variant 1, mRNA [NM_001455] | 1.697232 | 1.536268 | 0.160964 | 0.888559 |
| DMBT1 | Homo sapiens deleted in malignant brain tumors 1 (DMBT1), transcript variant 2, mRNA [NM_007329] | 1.58671 | 1.425213 | 0.161496 | 0.888223 |
| SIX2 | Homo sapiens SIX homeobox 2 (SIX2), mRNA [NM_016932] | 1.472241 | 1.309662 | 0.162579 | 0.888223 |
| DST | Homo sapiens dystonin (DST), transcript variant 1e, mRNA [NM_001723] | 1.509334 | 1.3465 | 0.162833 | 0.888223 |
| PDX1 | Homo sapiens pancreatic and duodenal homeobox 1 (PDX1), mRNA [NM_000209] | 1.595676 | 1.432758 | 0.162917 | 0.89325 |
| GANC | Homo sapiens calpain 3, (p94) (CAPN3), transcript variant 1, mRNA [NM_000070] | 1.635226 | 1.47201 | 0.163216 | 0.913498 |
| POU4F1 | Homo sapiens POU class 4 homeobox 1 (POU4F1), mRNA [NM_006237] | 2.714038 | 2.550471 | 0.163567 | 0.97282 |
| FIG4 | Homo sapiens FIG4 homolog, SAC1 lipid phosphatase domain containing (S. cerevisiae) (FIG4), mRNA [NM_014845] | 1.515754 | 1.351165 | 0.164589 | 0.888223 |
| LRRC8A | Homo sapiens leucine rich repeat containing 8 family, member A (LRRC8A), transcript variant 2, mRNA [NM_019594] | 1.952462 | 1.787823 | 0.164639 | 0.924768 |
| MYF6 | Homo sapiens myogenic factor 6 (herculin) (MYF6), mRNA [NM_002469] | 2.643997 | 2.47928 | 0.164717 | 0.946481 |
| AK090448 | Rho guanine nucleotide exchange factor (GEF) 1 [Source:HGNC Symbol;Acc:681] [ENST00000316079] | 7.851748 | 7.685615 | 0.166134 | 0.935333 |
| IL15RA | Homo sapiens interleukin 15 receptor, alpha (IL15RA), transcript variant 3, mRNA [NM_001243539] | 4.407719 | 4.24157 | 0.166149 | 0.92286 |
| STRA6 | Homo sapiens stimulated by retinoic acid gene 6 homolog (mouse) (STRA6), transcript variant 5, mRNA [NM_001142620] | 2.625588 | 2.457981 | 0.167607 | 0.952549 |
| CDH23 | Homo sapiens cadherin-related 23 (CDH23), transcript variant 1, mRNA [NM_022124] | 3.753614 | 3.585998 | 0.167616 | 0.922544 |
| PSD | Homo sapiens pleckstrin and Sec7 domain containing (PSD), mRNA [NM_002779] | 3.240378 | 3.072693 | 0.167684 | 0.967513 |
| SP1 | Homo sapiens Sp1 transcription factor (SP1), transcript variant 1, mRNA [NM_138473] | 1.554095 | 1.386379 | 0.167717 | 0.888223 |
| BBS2 | Homo sapiens Bardet-Biedl syndrome 2 (BBS2), mRNA [NM_031885] | 2.599269 | 2.431054 | 0.168215 | 0.895269 |
| RANBP9 | Homo sapiens RAN binding protein 9 (RANBP9), mRNA [NM_005493] | 1.619767 | 1.450858 | 0.168909 | 0.888223 |
| F11R | Homo sapiens F11 receptor (F11R), mRNA [NM_016946] | 2.442923 | 2.273198 | 0.169725 | 0.972186 |
| POU4F2 | Homo sapiens POU class 4 homeobox 2 (POU4F2), mRNA [NM_004575] | 1.624113 | 1.453783 | 0.17033 | 0.888223 |
| OGDH | Homo sapiens oxoglutarate (alpha-ketoglutarate) dehydrogenase (lipoamide) (OGDH), nuclear gene encoding mitochondrial protein, transcript variant 2, mRNA [NM_001003941] | 1.505334 | 1.334903 | 0.170431 | 0.888223 |
| XYLT1 | Homo sapiens xylosyltransferase I (XYLT1), mRNA [NM_022166] | 1.535562 | 1.364683 | 0.170879 | 0.888223 |
| DKK1 | Homo sapiens dickkopf homolog 1 (Xenopus laevis) (DKK1), mRNA [NM_012242] | 1.615894 | 1.444985 | 0.17091 | 0.888223 |
| FZD6 | Homo sapiens frizzled family receptor 6 (FZD6), transcript variant 1, mRNA [NM_003506] | 1.834684 | 1.660985 | 0.173699 | 0.956522 |
| CAV1 | Homo sapiens caveolin 1, caveolae protein, 22kDa (CAV1), transcript variant 1, mRNA [NM_001753] | 2.228281 | 2.054462 | 0.173819 | 0.953461 |
| FZD10 | Homo sapiens frizzled family receptor 10 (FZD10), mRNA [NM_007197] | 1.609338 | 1.43536 | 0.173978 | 0.895701 |
| KIAA1598 | Homo sapiens mRNA; cDNA DKFZp686A0439 (from clone DKFZp686A0439). [CR749417] | 1.618188 | 1.443507 | 0.174681 | 0.916669 |
| ATG5 | Homo sapiens ATG5 autophagy related 5 homolog (S. cerevisiae) (ATG5), mRNA [NM_004849] | 1.584161 | 1.409427 | 0.174734 | 0.888223 |
| ENST00000394326 | Homo sapiens mRNA for katanin p80 subunit B 1 variant protein. [AB209250] | 2.553683 | 2.378557 | 0.175126 | 0.949687 |
| SEMA3B | Homo sapiens sema domain, immunoglobulin domain (Ig), short basic domain, secreted, (semaphorin) 3B (SEMA3B), transcript variant 1, mRNA [NM_004636] | 6.236628 | 6.060434 | 0.176193 | 0.922185 |
| TWSG1 | Homo sapiens twisted gastrulation homolog 1 (Drosophila) (TWSG1), mRNA [NM_020648] | 1.851935 | 1.674392 | 0.177543 | 0.932415 |
| HOXC10 | Homo sapiens homeobox C10 (HOXC10), mRNA [NM_017409] | 2.174388 | 1.996019 | 0.178369 | 0.948453 |
| ADAM9 | Homo sapiens ADAM metallopeptidase domain 9 (ADAM9), transcript variant 1, mRNA [NM_003816] | 1.630385 | 1.451869 | 0.178516 | 0.894216 |
| PTPRZ1 | Homo sapiens protein tyrosine phosphatase, receptor-type, Z polypeptide 1 (PTPRZ1), transcript variant 1, mRNA [NM_002851] | 1.890707 | 1.712061 | 0.178645 | 0.96199 |
| GATA4 | Homo sapiens GATA binding protein 4 (GATA4), mRNA [NM_002052] | 1.512013 | 1.333112 | 0.178901 | 0.888223 |
| NELL1 | Homo sapiens NEL-like 1 (chicken) (NELL1), transcript variant 1, mRNA [NM_006157] | 1.486992 | 1.307982 | 0.17901 | 0.888223 |
| DNMT3B | DNA (cytosine-5-)-methyltransferase 3 beta [Source:HGNC Symbol;Acc:2979] [ENST00000537219] | 2.059006 | 1.878185 | 0.180822 | 0.935544 |
| PARD3 | Homo sapiens par-3 partitioning defective 3 homolog (C. elegans) (PARD3), transcript variant 9, mRNA [NM_001184792] | 3.392516 | 3.210504 | 0.182012 | 0.964566 |
| NEURL | Homo sapiens neuralized homolog (Drosophila) (NEURL), mRNA [NM_004210] | 2.129743 | 1.946331 | 0.183411 | 0.942153 |
| NANOS3 | Homo sapiens nanos homolog 3 (Drosophila) (NANOS3), mRNA [NM_001098622] | 2.293467 | 2.109503 | 0.183964 | 0.954723 |
| FGF20 | Homo sapiens fibroblast growth factor 20 (FGF20), mRNA [NM_019851] | 1.750058 | 1.564613 | 0.185445 | 0.923937 |
| AXL | Homo sapiens AXL receptor tyrosine kinase (AXL), transcript variant 1, mRNA [NM_021913] | 1.562129 | 1.376124 | 0.186005 | 0.888223 |
| HSD17B4 | Homo sapiens hydroxysteroid (17-beta) dehydrogenase 4 (HSD17B4), transcript variant 2, mRNA [NM_000414] | 2.990284 | 2.804227 | 0.186058 | 0.963623 |
| RPS6KA5 | Homo sapiens ribosomal protein S6 kinase, 90kDa, polypeptide 5 (RPS6KA5), transcript variant 2, mRNA [NM_182398] | 1.64977 | 1.463599 | 0.186171 | 0.888223 |
| XDH | Homo sapiens xanthine dehydrogenase (XDH), mRNA [NM_000379] | 4.07154 | 3.885108 | 0.186432 | 0.940219 |
| PTEN | Homo sapiens phosphatase and tensin homolog (PTEN), mRNA [NM_000314] | 2.348923 | 2.162235 | 0.186688 | 0.888223 |
| PITPNA | Homo sapiens phosphatidylinositol transfer protein, alpha (PITPNA), mRNA [NM_006224] | 1.603227 | 1.415487 | 0.18774 | 0.888223 |
| GLI2 | Homo sapiens GLI family zinc finger 2 (GLI2), mRNA [NM_005270] | 1.55137 | 1.362988 | 0.188382 | 0.888223 |
| TBX1 | Homo sapiens T-box 1 (TBX1), transcript variant C, mRNA [NM_080647] | 1.567574 | 1.37896 | 0.188614 | 0.888223 |
| NANOG | Homo sapiens Nanog homeobox (NANOG), mRNA [NM_024865] | 1.985215 | 1.79658 | 0.188636 | 0.955793 |
| FHL2 | Homo sapiens four and a half LIM domains 2 (FHL2), transcript variant 2, mRNA [NM_201555] | 1.616572 | 1.427526 | 0.189045 | 0.888223 |
| EYA2 | Homo sapiens eyes absent homolog 2 (Drosophila) (EYA2), transcript variant 1, mRNA [NM_005244] | 4.549307 | 4.360112 | 0.189195 | 0.888223 |
| CSNK2A1 | Homo sapiens casein kinase 2, alpha 1 polypeptide (CSNK2A1), transcript variant 1, mRNA [NM_177559] | 1.680928 | 1.491693 | 0.189235 | 0.905818 |
| CELSR1 | Homo sapiens cadherin, EGF LAG seven-pass G-type receptor 1 (flamingo homolog, Drosophila) (CELSR1), mRNA [NM_014246] | 7.106657 | 6.916501 | 0.190156 | 0.984275 |
| MSR1 | Homo sapiens macrophage scavenger receptor 1 (MSR1), transcript variant SR-AI, mRNA [NM_138715] | 3.199238 | 3.008484 | 0.190754 | 0.888223 |
| ARIH2 | Homo sapiens ariadne homolog 2 (Drosophila) (ARIH2), mRNA [NM_006321] | 3.582743 | 3.391708 | 0.191035 | 0.950677 |
| GALR2 | Homo sapiens galanin receptor 2 (GALR2), mRNA [NM_003857] | 2.615755 | 2.424254 | 0.191502 | 0.965408 |
| UBE2B | Homo sapiens ubiquitin-conjugating enzyme E2B (UBE2B), mRNA [NM_003337] | 1.598103 | 1.406168 | 0.191935 | 0.888223 |
| DHRS9 | Homo sapiens dehydrogenase/reductase (SDR family) member 9 (DHRS9), transcript variant 1, mRNA [NM_005771] | 1.679132 | 1.487162 | 0.19197 | 0.888223 |
| ACTG1 | Homo sapiens actin, gamma 1 (ACTG1), transcript variant 2, mRNA [NM_001614] | 1.633877 | 1.441806 | 0.19207 | 0.888223 |
| NRXN1 | Homo sapiens neurexin 1 (NRXN1), transcript variant alpha2, mRNA [NM_001135659] | 2.705404 | 2.513249 | 0.192155 | 0.965932 |
| OSR2 | Homo sapiens odd-skipped related 2 (Drosophila) (OSR2), transcript variant 2, mRNA [NM_053001] | 1.61138 | 1.417687 | 0.193693 | 0.888223 |
| AMH | Homo sapiens anti-Mullerian hormone (AMH), mRNA [NM_000479] | 1.927789 | 1.733834 | 0.193955 | 0.944508 |
| DISC1 | Homo sapiens disrupted in schizophrenia 1 (DISC1), transcript variant l, mRNA [NM_001164549] | 1.656845 | 1.462172 | 0.194672 | 0.888223 |
| CD2 | Homo sapiens CD2 molecule (CD2), mRNA [NM_001767] | 1.753689 | 1.558914 | 0.194775 | 0.943839 |
| IRX5 | Homo sapiens iroquois homeobox 5 (IRX5), transcript variant 1, mRNA [NM_005853] | 1.7001 | 1.505116 | 0.194984 | 0.888223 |
| SNAP25 | Homo sapiens synaptosomal-associated protein, 25kDa (SNAP25), transcript variant 1, mRNA [NM_003081] | 2.67025 | 2.475126 | 0.195124 | 0.9524 |
| PTPN22 | Homo sapiens protein tyrosine phosphatase, non-receptor type 22 (lymphoid) (PTPN22), transcript variant 2, mRNA [NM_012411] | 3.463868 | 3.268646 | 0.195223 | 0.946563 |
| ENST00000370677 | laminin, alpha 5 [Source:HGNC Symbol;Acc:6485] [ENST00000370677] | 12.60394 | 12.40847 | 0.195466 | 0.944599 |
| MAPK9 | Homo sapiens mitogen-activated protein kinase 9 (MAPK9), transcript variant JNK2-g, mRNA [NM_001135044] | 1.636408 | 1.440757 | 0.19565 | 0.888223 |
| NTN3 | Homo sapiens netrin 3 (NTN3), mRNA [NM_006181] | 1.552946 | 1.357155 | 0.195792 | 0.888223 |
| NRP2 | Homo sapiens neuropilin 2 (NRP2), transcript variant 1, mRNA [NM_201266] | 1.577632 | 1.380836 | 0.196796 | 0.888223 |
| SSH2 | slingshot homolog 2 (Drosophila) [Source:HGNC Symbol;Acc:30580] [ENST00000324677] | 1.637523 | 1.440515 | 0.197009 | 0.888223 |
| LIFR | Homo sapiens leukemia inhibitory factor receptor alpha (LIFR), transcript variant 2, mRNA [NM_002310] | 1.493299 | 1.296045 | 0.197254 | 0.888223 |
| TRPV4 | Homo sapiens transient receptor potential cation channel, subfamily V, member 4 (TRPV4), transcript variant 2, mRNA [NM_147204] | 1.625319 | 1.425419 | 0.1999 | 0.888223 |
| MARK1 | Homo sapiens MAP/microtubule affinity-regulating kinase 1 (MARK1), mRNA [NM_018650] | 1.633064 | 1.432991 | 0.200073 | 0.888223 |
| CDKN2A | Homo sapiens cyclin-dependent kinase inhibitor 2A (melanoma, p16, inhibits CDK4) (CDKN2A), transcript variant 3, mRNA [NM_058197] | 1.558261 | 1.357273 | 0.200988 | 0.888223 |
| ERBB2 | Homo sapiens c-ERBB-2 mRNA, 5' UTR. [AB025286] | 5.287532 | 5.085836 | 0.201696 | 0.93885 |
| TP63 | Homo sapiens tumor protein p63 (TP63), transcript variant 1, mRNA [NM_003722] | 3.174982 | 2.972753 | 0.202229 | 0.921399 |
| CXCL12 | Homo sapiens chemokine (C-X-C motif) ligand 12 (CXCL12), transcript variant 3, mRNA [NM_001033886] | 4.283441 | 4.080508 | 0.202933 | 0.888223 |
| CACNB2 | Homo sapiens calcium channel, voltage-dependent, beta 2 subunit (CACNB2), transcript variant 9, mRNA [NM_001167945] | 1.537755 | 1.334192 | 0.203562 | 0.888223 |
| NFIB | Homo sapiens nuclear factor I/B (NFIB), transcript variant 3, mRNA [NM_005596] | 1.565043 | 1.360959 | 0.204083 | 0.888223 |
| MIZF | Homo sapiens histone H4 transcription factor (HINFP), transcript variant 1, mRNA [NM_015517] | 4.663756 | 4.459144 | 0.204612 | 0.911361 |
| CYR61 | Homo sapiens cysteine-rich, angiogenic inducer, 61 (CYR61), mRNA [NM_001554] | 1.686757 | 1.481341 | 0.205416 | 0.897441 |
| CALCA | Homo sapiens calcitonin-related polypeptide alpha (CALCA), transcript variant 2, mRNA [NM_001033952] | 3.194888 | 2.989293 | 0.205595 | 0.954043 |
| F2R | Homo sapiens coagulation factor II (thrombin) receptor (F2R), mRNA [NM_001992] | 1.51151 | 1.305656 | 0.205854 | 0.888223 |
| FIGLA | Homo sapiens folliculogenesis specific basic helix-loop-helix (FIGLA), mRNA [NM_001004311] | 2.288384 | 2.082295 | 0.20609 | 0.945624 |
| RHOB | Homo sapiens ras homolog gene family, member B (RHOB), mRNA [NM_004040] | 1.639011 | 1.432305 | 0.206706 | 0.888223 |
| WNT3 | Homo sapiens wingless-type MMTV integration site family, member 3 (WNT3), mRNA [NM_030753] | 4.323345 | 4.116515 | 0.20683 | 0.933783 |
| PICK1 | Homo sapiens protein interacting with PRKCA 1 (PICK1), transcript variant 1, mRNA [NM_012407] | 5.062335 | 4.855115 | 0.20722 | 0.956131 |
| GABRA5 | Homo sapiens gamma-aminobutyric acid (GABA) A receptor, alpha 5 (GABRA5), transcript variant 1, mRNA [NM_000810] | 1.958026 | 1.749576 | 0.20845 | 0.941944 |
| ST8SIA4 | Homo sapiens ST8 alpha-N-acetyl-neuraminide alpha-2,8-sialyltransferase 4 (ST8SIA4), transcript variant 1, mRNA [NM_005668] | 1.51507 | 1.306415 | 0.208655 | 0.888223 |
| RTN4RL1 | Homo sapiens reticulon 4 receptor-like 1 (RTN4RL1), mRNA [NM_178568] | 2.442205 | 2.233043 | 0.209162 | 0.953461 |
| ENST00000374655 | wingless-type MMTV integration site family, member 4 [Source:HGNC Symbol;Acc:12783] [ENST00000374655] | 1.544867 | 1.334957 | 0.20991 | 0.888223 |
| BCL2 | Homo sapiens B-cell CLL/lymphoma 2 (BCL2), nuclear gene encoding mitochondrial protein, transcript variant alpha, mRNA [NM_000633] | 1.664851 | 1.454162 | 0.210689 | 0.888223 |
| CXADR | Homo sapiens coxsackie virus and adenovirus receptor (CXADR), transcript variant 1, mRNA [NM_001338] | 1.626203 | 1.415072 | 0.21113 | 0.888223 |
| SPIB | Homo sapiens Spi-B transcription factor (Spi-1/PU.1 related) (SPIB), transcript variant 1, mRNA [NM_003121] | 1.658507 | 1.445266 | 0.21324 | 0.888223 |
| ROBO3 | Homo sapiens roundabout, axon guidance receptor, homolog 3 (Drosophila) (ROBO3), mRNA [NM_022370] | 2.418149 | 2.204857 | 0.213292 | 0.958128 |
| TAF4 | Homo sapiens TAF4 RNA polymerase II, TATA box binding protein (TBP)-associated factor, 135kDa (TAF4), mRNA [NM_003185] | 2.693879 | 2.48009 | 0.213789 | 0.963101 |
| TET1 | Homo sapiens tet methylcytosine dioxygenase 1 (TET1), mRNA [NM_030625] | 1.538943 | 1.325119 | 0.213825 | 0.888223 |
| NR4A2 | Homo sapiens nuclear receptor subfamily 4, group A, member 2 (NR4A2), mRNA [NM_006186] | 1.71233 | 1.496804 | 0.215527 | 0.888223 |
| DHRS2 | Homo sapiens dehydrogenase/reductase (SDR family) member 2 (DHRS2), transcript variant 1, mRNA [NM_182908] | 5.617168 | 5.401397 | 0.215771 | 0.979887 |
| HEY2 | Homo sapiens hairy/enhancer-of-split related with YRPW motif 2 (HEY2), mRNA [NM_012259] | 1.668208 | 1.451959 | 0.216249 | 0.888223 |
| OLIG1 | Homo sapiens oligodendrocyte transcription factor 1 (OLIG1), mRNA [NM_138983] | 1.503347 | 1.286355 | 0.216992 | 0.888223 |
| LRP5 | Homo sapiens low density lipoprotein receptor-related protein 5 (LRP5), mRNA [NM_002335] | 2.351989 | 2.134791 | 0.217198 | 0.954723 |
| PPHLN1 | Homo sapiens periphilin 1 (PPHLN1), transcript variant 1, mRNA [NM_016488] | 1.747195 | 1.529783 | 0.217411 | 0.888223 |
| RNF17 | Homo sapiens ring finger protein 17 (RNF17), transcript variant 1, mRNA [NM_031277] | 1.508507 | 1.290923 | 0.217584 | 0.888223 |
| NUMA1 | Homo sapiens nuclear mitotic apparatus protein 1 (NUMA1), mRNA [NM_006185] | 2.049487 | 1.831349 | 0.218138 | 0.93568 |
| PPARGC1B | Homo sapiens peroxisome proliferator-activated receptor gamma, coactivator 1 beta (PPARGC1B), transcript variant 1, mRNA [NM_133263] | 1.62059 | 1.402215 | 0.218375 | 0.888223 |
| AQP3 | Homo sapiens aquaporin 3 (Gill blood group) (AQP3), mRNA [NM_004925] | 3.153509 | 2.935057 | 0.218452 | 0.888223 |
| PPARGC1B | Homo sapiens peroxisome proliferator-activated receptor gamma, coactivator 1 beta (PPARGC1B), transcript variant 1, mRNA [NM_133263] | 2.79122 | 2.570484 | 0.220736 | 0.938226 |
| INSL6 | Homo sapiens insulin-like 6 (INSL6), mRNA [NM_007179] | 1.589037 | 1.367688 | 0.221349 | 0.888223 |
| GRM5 | Homo sapiens glutamate receptor, metabotropic 5 (GRM5), transcript variant b, mRNA [NM_000842] | 1.638361 | 1.415387 | 0.222974 | 0.888683 |
| ERAP1 | Homo sapiens endoplasmic reticulum aminopeptidase 1 (ERAP1), transcript variant 2, mRNA [NM_001040458] | 1.737967 | 1.514956 | 0.223011 | 0.888223 |
| EPHB2 | Homo sapiens EPH receptor B2 (EPHB2), transcript variant 2, mRNA [NM_004442] | 1.703174 | 1.480006 | 0.223167 | 0.888223 |
| SMOC1 | Homo sapiens SPARC related modular calcium binding 1 (SMOC1), transcript variant 1, mRNA [NM_001034852] | 1.724134 | 1.500933 | 0.223201 | 0.895437 |
| PHOX2B | Homo sapiens paired-like homeobox 2b (PHOX2B), mRNA [NM_003924] | 1.88332 | 1.659338 | 0.223982 | 0.941333 |
| SLC7A11 | Homo sapiens solute carrier family 7 (anionic amino acid transporter light chain, xc- system), member 11 (SLC7A11), mRNA [NM_014331] | 2.247847 | 2.022208 | 0.225639 | 0.958232 |
| MITF | Homo sapiens microphthalmia-associated transcription factor (MITF), transcript variant 1, mRNA [NM_198159] | 1.644355 | 1.418322 | 0.226033 | 0.888223 |
| GPX1 | Homo sapiens glutathione peroxidase 1 (GPX1), transcript variant 2, mRNA [NM_201397] | 2.595068 | 2.368979 | 0.226089 | 0.946839 |
| ADNP | activity-dependent neuroprotector homeobox [Source:HGNC Symbol;Acc:15766] [ENST00000349014] | 2.088397 | 1.861195 | 0.227202 | 0.952754 |
| CACNA1G | Homo sapiens calcium channel, voltage-dependent, T type, alpha 1G subunit (CACNA1G), transcript variant 15, mRNA [NM_198397] | 3.891432 | 3.664185 | 0.227247 | 0.951513 |
| SPOCK1 | Homo sapiens sparc/osteonectin, cwcv and kazal-like domains proteoglycan (testican) 1 (SPOCK1), mRNA [NM_004598] | 1.591958 | 1.363149 | 0.228809 | 0.888223 |
| ACTC1 | Homo sapiens actin, alpha, cardiac muscle 1 (ACTC1), mRNA [NM_005159] | 1.636866 | 1.407242 | 0.229624 | 0.888223 |
| L1CAM | Homo sapiens L1 cell adhesion molecule (L1CAM), transcript variant 2, mRNA [NM_024003] | 1.782035 | 1.552392 | 0.229643 | 0.888223 |
| OSR1 | Homo sapiens odd-skipped related 1 (Drosophila) (OSR1), mRNA [NM_145260] | 1.669827 | 1.439788 | 0.230039 | 0.888223 |
| LILRB4 | leukocyte immunoglobulin-like receptor, subfamily B (with TM and ITIM domains), member 4 [Source:HGNC Symbol;Acc:6608] [ENST00000391736] | 1.543978 | 1.312992 | 0.230986 | 0.888223 |
| SOS1 | Homo sapiens son of sevenless homolog 1 (Drosophila) (SOS1), mRNA [NM_005633] | 3.343759 | 3.112707 | 0.231051 | 0.932096 |
| USH2A | Homo sapiens Usher syndrome 2A (autosomal recessive, mild) (USH2A), transcript variant 2, mRNA [NM_206933] | 3.209526 | 2.976947 | 0.232579 | 0.951928 |
| NLGN1 | Homo sapiens neuroligin 1 (NLGN1), mRNA [NM_014932] | 2.716067 | 2.481919 | 0.234148 | 0.956328 |
| VSX1 | Homo sapiens visual system homeobox 1 (VSX1), transcript variant 1, mRNA [NM_014588] | 2.425916 | 2.19167 | 0.234246 | 0.894891 |
| WT1 | Homo sapiens Wilms tumor 1 (WT1), transcript variant D, mRNA [NM_024426] | 1.673031 | 1.438769 | 0.234261 | 0.888223 |
| AQP3 | Homo sapiens aquaporin 3 (Gill blood group) (AQP3), mRNA [NM_004925] | 1.683517 | 1.448305 | 0.235212 | 0.894216 |
| EIF2B2 | Homo sapiens eukaryotic translation initiation factor 2B, subunit 2 beta, 39kDa (EIF2B2), mRNA [NM_014239] | 3.336706 | 3.099251 | 0.237455 | 0.908518 |
| SDC1 | Homo sapiens syndecan 1 (SDC1), transcript variant 1, mRNA [NM_001006946] | 1.683201 | 1.445601 | 0.2376 | 0.888223 |
| NRP2 | Homo sapiens neuropilin 2 (NRP2), transcript variant 6, mRNA [NM_201264] | 1.566843 | 1.32861 | 0.238233 | 0.888223 |
| ITPKB | Homo sapiens inositol-trisphosphate 3-kinase B (ITPKB), mRNA [NM_002221] | 3.106808 | 2.868347 | 0.23846 | 0.957574 |
| CACYBP | Homo sapiens calcyclin binding protein (CACYBP), transcript variant 1, mRNA [NM_014412] | 1.710809 | 1.471872 | 0.238937 | 0.888223 |
| RASGRF1 | Homo sapiens Ras protein-specific guanine nucleotide-releasing factor 1 (RASGRF1), transcript variant 1, mRNA [NM_002891] | 2.072765 | 1.83254 | 0.240225 | 0.924837 |
| GHR | Homo sapiens growth hormone receptor (GHR), transcript variant 1, mRNA [NM_000163] | 1.668528 | 1.427685 | 0.240843 | 0.888223 |
| IL27 | Homo sapiens interleukin 27 (IL27), mRNA [NM_145659] | 1.669758 | 1.427403 | 0.242354 | 0.888223 |
| KIT | Homo sapiens v-kit Hardy-Zuckerman 4 feline sarcoma viral oncogene homolog (KIT), transcript variant 1, mRNA [NM_000222] | 2.107839 | 1.865215 | 0.242624 | 0.888223 |
| HOXD9 | Homo sapiens homeobox D9 (HOXD9), mRNA [NM_014213] | 5.715379 | 5.472432 | 0.242947 | 0.888223 |
| ENST00000374768 | par-3 partitioning defective 3 homolog (C. elegans) [Source:HGNC Symbol;Acc:16051] [ENST00000374768] | 1.561926 | 1.318117 | 0.243809 | 0.888223 |
| CD3E | Homo sapiens CD3e molecule, epsilon (CD3-TCR complex) (CD3E), mRNA [NM_000733] | 3.148464 | 2.904465 | 0.243999 | 0.935544 |
| POU2F3 | Homo sapiens POU class 2 homeobox 3 (POU2F3), transcript variant 1, mRNA [NM_014352] | 1.735959 | 1.491365 | 0.244594 | 0.888223 |
| KGFLP1 | Homo sapiens fibroblast growth factor 7 pseudogene (KGFLP1), non-coding RNA [NR_003674] | 5.017936 | 4.773236 | 0.244699 | 0.888223 |
| NCAN | Homo sapiens neurocan (NCAN), mRNA [NM_004386] | 6.095464 | 5.850198 | 0.245266 | 0.906152 |
| ADAMTS1 | Homo sapiens ADAM metallopeptidase with thrombospondin type 1 motif, 1 (ADAMTS1), mRNA [NM_006988] | 5.312987 | 5.067485 | 0.245502 | 0.888223 |
| ERMP1 | Homo sapiens endoplasmic reticulum metallopeptidase 1 (ERMP1), mRNA [NM_024896] | 6.350466 | 6.104504 | 0.245962 | 0.900741 |
| KLK6 | Homo sapiens kallikrein-related peptidase 6 (KLK6), transcript variant B, mRNA [NM_001012964] | 2.688917 | 2.442824 | 0.246093 | 0.96766 |
| POU5F1 | Homo sapiens POU class 5 homeobox 1 (POU5F1), transcript variant 1, mRNA [NM_002701] | 1.642451 | 1.395791 | 0.246661 | 0.888223 |
| STK4 | Homo sapiens serine/threonine kinase 4 (STK4), mRNA [NM_006282] | 3.538284 | 3.291204 | 0.24708 | 0.902384 |
| RPS14 | Homo sapiens ribosomal protein S14 (RPS14), transcript variant 1, mRNA [NM_001025071] | 3.874288 | 3.626582 | 0.247705 | 0.944912 |
| STAT3 | Homo sapiens signal transducer and activator of transcription 3 (acute-phase response factor) (STAT3), transcript variant 3, mRNA [NM_213662] | 4.311511 | 4.063777 | 0.247734 | 0.919441 |
| IREB2 | Homo sapiens iron-responsive element binding protein 2 (IREB2), mRNA [NM_004136] | 1.701465 | 1.452448 | 0.249017 | 0.888223 |
| ARID5B | Homo sapiens AT rich interactive domain 5B (MRF1-like) (ARID5B), transcript variant 1, mRNA [NM_032199] | 1.719674 | 1.470439 | 0.249235 | 0.888223 |
| A_19_P00804817 | Homo sapiens SMAD family member 4 (SMAD4), mRNA [NM_005359] | 1.994004 | 1.74353 | 0.250474 | 0.921985 |
| RHCG | Homo sapiens Rh family, C glycoprotein (RHCG), mRNA [NM_016321] | 1.576484 | 1.325779 | 0.250705 | 0.888223 |
| HNRNPAB | Homo sapiens heterogeneous nuclear ribonucleoprotein A/B (HNRNPAB), transcript variant 2, mRNA [NM_004499] | 1.941392 | 1.690434 | 0.250958 | 0.906788 |
| POR | Homo sapiens P450 (cytochrome) oxidoreductase (POR), mRNA [NM_000941] | 1.843634 | 1.590445 | 0.253188 | 0.888223 |
| NTRK3 | Homo sapiens neurotrophic tyrosine kinase, receptor, type 3 (NTRK3), transcript variant 3, mRNA [NM_001007156] | 1.740818 | 1.487067 | 0.253751 | 0.888223 |
| SCLT1 | Homo sapiens sodium channel and clathrin linker 1 (SCLT1), mRNA [NM_144643] | 1.5588 | 1.304783 | 0.254017 | 0.888223 |
| RBM24 | Homo sapiens RNA binding motif protein 24 (RBM24), transcript variant 2, mRNA [NM_153020] | 1.664772 | 1.410485 | 0.254287 | 0.888223 |
| CSRP2 | Homo sapiens cysteine and glycine-rich protein 2 (CSRP2), mRNA [NM_001321] | 1.79509 | 1.540639 | 0.254451 | 0.888223 |
| HEY1 | Homo sapiens hairy/enhancer-of-split related with YRPW motif 1 (HEY1), transcript variant 2, mRNA [NM_001040708] | 1.744482 | 1.488895 | 0.255587 | 0.888223 |
| SEMA3F | Homo sapiens sema domain, immunoglobulin domain (Ig), short basic domain, secreted, (semaphorin) 3F (SEMA3F), mRNA [NM_004186] | 3.043664 | 2.787511 | 0.256153 | 0.888223 |
| DMRT2 | Homo sapiens doublesex and mab-3 related transcription factor 2 (DMRT2), transcript variant 1, mRNA [NM_006557] | 1.735388 | 1.479042 | 0.256346 | 0.888223 |
| GREM1 | Homo sapiens gremlin 1 (GREM1), transcript variant 1, mRNA [NM_013372] | 2.023657 | 1.767152 | 0.256505 | 0.888223 |
| DEAF1 | Homo sapiens deformed epidermal autoregulatory factor 1 (Drosophila) (DEAF1), mRNA [NM_021008] | 2.158384 | 1.900903 | 0.25748 | 0.935544 |
| OVOL2 | Homo sapiens ovo-like 2 (Drosophila) (OVOL2), mRNA [NM_021220] | 2.6078 | 2.349679 | 0.258121 | 0.888223 |
| MYO10 | Homo sapiens myosin X (MYO10), mRNA [NM_012334] | 2.283166 | 2.024757 | 0.258409 | 0.92693 |
| ENST00000383765 | transforming growth factor, beta receptor II (70/80kDa) [Source:HGNC Symbol;Acc:11773] [ENST00000383765] | 1.96544 | 1.706725 | 0.258715 | 0.909677 |
| LAMC1 | Homo sapiens laminin, gamma 1 (formerly LAMB2) (LAMC1), mRNA [NM_002293] | 3.01991 | 2.760877 | 0.259033 | 0.951646 |
| GATA3 | Homo sapiens GATA binding protein 3 (GATA3), transcript variant 1, mRNA [NM_001002295] | 1.993206 | 1.733838 | 0.259367 | 0.934619 |
| CDH2 | Homo sapiens cadherin 2, type 1, N-cadherin (neuronal) (CDH2), mRNA [NM_001792] | 6.523726 | 6.263096 | 0.260631 | 0.896749 |
| ABCA2 | Homo sapiens fucosyltransferase 7 (alpha (1,3) fucosyltransferase) (FUT7), mRNA [NM_004479] | 4.112218 | 3.85142 | 0.260798 | 0.933216 |
| PPP2R5B | Homo sapiens protein phosphatase 2, regulatory subunit B', beta (PPP2R5B), mRNA [NM_006244] | 1.604398 | 1.343447 | 0.260951 | 0.888223 |
| PKDCC | protein kinase domain containing, cytoplasmic homolog (mouse) [Source:HGNC Symbol;Acc:25123] [ENST00000492861] | 4.840267 | 4.579141 | 0.261126 | 0.900741 |
| TCF7L2 | Homo sapiens transcription factor 7-like 2 (T-cell specific, HMG-box) (TCF7L2), transcript variant 13, mRNA [NM_001198531] | 2.912489 | 2.650343 | 0.262146 | 0.895437 |
| BBS9 | Homo sapiens Bardet-Biedl syndrome 9 (BBS9), transcript variant 2, mRNA [NM_198428] | 2.924847 | 2.662537 | 0.26231 | 0.92819 |
| LCE1B | Homo sapiens late cornified envelope 1B (LCE1B), mRNA [NM_178349] | 1.820097 | 1.557066 | 0.263032 | 0.904813 |
| WNT8B | Homo sapiens wingless-type MMTV integration site family, member 8B (WNT8B), mRNA [NM_003393] | 2.924383 | 2.661139 | 0.263244 | 0.916701 |
| GRIN3A | Homo sapiens glutamate receptor, ionotropic, N-methyl-D-aspartate 3A (GRIN3A), mRNA [NM_133445] | 1.692794 | 1.428926 | 0.263869 | 0.888223 |
| RBMY1B | Homo sapiens RNA binding motif protein, Y-linked, family 1, member B (RBMY1B), mRNA [NM_001006121] | 2.547645 | 2.283677 | 0.263969 | 0.943621 |
| AXIN1 | Homo sapiens axin 1 (AXIN1), transcript variant 1, mRNA [NM_003502] | 2.973093 | 2.708716 | 0.264377 | 0.93567 |
| BMP5 | Homo sapiens bone morphogenetic protein 5 (BMP5), mRNA [NM_021073] | 1.686695 | 1.421739 | 0.264957 | 0.892866 |
| WNT10A | Homo sapiens wingless-type MMTV integration site family, member 10A (WNT10A), mRNA [NM_025216] | 5.331326 | 5.064591 | 0.266735 | 0.928887 |
| ATP7A | ATPase, Cu++ transporting, alpha polypeptide [Source:HGNC Symbol;Acc:869] [ENST00000355691] | 2.261578 | 1.994301 | 0.267277 | 0.930933 |
| CDKN2A | Homo sapiens cyclin-dependent kinase inhibitor 2A (melanoma, p16, inhibits CDK4) (CDKN2A), transcript variant 1, mRNA [NM_000077] | 5.59231 | 5.324928 | 0.267382 | 0.894891 |
| HEMGN | Homo sapiens hemogen (HEMGN), transcript variant 1, mRNA [NM_018437] | 1.627678 | 1.359538 | 0.26814 | 0.888223 |
| PLAG1 | Homo sapiens pleiomorphic adenoma gene 1 (PLAG1), transcript variant 1, mRNA [NM_002655] | 1.696203 | 1.427688 | 0.268515 | 0.888223 |
| CYTL1 | Homo sapiens cytokine-like 1 (CYTL1), mRNA [NM_018659] | 1.559327 | 1.290345 | 0.268982 | 0.888223 |
| LMX1B | Homo sapiens LIM homeobox transcription factor 1, beta (LMX1B), transcript variant 3, mRNA [NM_001174146] | 6.373942 | 6.104787 | 0.269155 | 0.933632 |
| LRRC4C | Homo sapiens leucine rich repeat containing 4C (LRRC4C), mRNA [NM_020929] | 4.856615 | 4.587353 | 0.269262 | 0.90962 |
| ENST00000370177 | pancreatic progenitor cell differentiation and proliferation factor homolog (zebrafish) [Source:HGNC Symbol;Acc:16142] [ENST00000370177] | 5.634355 | 5.363655 | 0.2707 | 0.937965 |
| TUBB2A | Homo sapiens tubulin, beta 2A class IIa (TUBB2A), mRNA [NM_001069] | 4.159472 | 3.887853 | 0.271619 | 0.919861 |
| DISC1 | Homo sapiens disrupted in schizophrenia 1 (DISC1), transcript variant L, mRNA [NM_018662] | 2.553556 | 2.281909 | 0.271647 | 0.911503 |
| VPS33A | Homo sapiens vacuolar protein sorting 33 homolog A (S. cerevisiae) (VPS33A), mRNA [NM_022916] | 3.58304 | 3.309642 | 0.273399 | 0.933545 |
| ZP3 | Homo sapiens zona pellucida glycoprotein 3 (sperm receptor) (ZP3), transcript variant 2, mRNA [NM_007155] | 2.190043 | 1.916593 | 0.273449 | 0.94749 |
| SLC1A3 | Homo sapiens solute carrier family 1 (glial high affinity glutamate transporter), member 3 (SLC1A3), transcript variant 1, mRNA [NM_004172] | 1.721727 | 1.447847 | 0.273879 | 0.888223 |
| NOG | Homo sapiens noggin (NOG), mRNA [NM_005450] | 3.743009 | 3.468993 | 0.274016 | 0.918173 |
| NEUROG2 | Homo sapiens neurogenin 2 (NEUROG2), mRNA [NM_024019] | 1.641794 | 1.367466 | 0.274328 | 0.888223 |
| KIAA1109 | Homo sapiens KIAA1109 (KIAA1109), mRNA [NM_015312] | 1.687638 | 1.411663 | 0.275974 | 0.888223 |
| MET | Homo sapiens met proto-oncogene (hepatocyte growth factor receptor) (MET), transcript variant 2, mRNA [NM_000245] | 2.861874 | 2.585293 | 0.276581 | 0.888223 |
| ENST00000403284 | c-ros oncogene 1 , receptor tyrosine kinase [Source:HGNC Symbol;Acc:10261] [ENST00000403284] | 1.922692 | 1.644811 | 0.277881 | 0.903186 |
| IL6ST | Homo sapiens interleukin 6 signal transducer (gp130, oncostatin M receptor) (IL6ST), transcript variant 3, mRNA [NM_001190981] | 3.374513 | 3.095883 | 0.27863 | 0.89091 |
| OSTN | Homo sapiens osteocrin (OSTN), mRNA [NM_198184] | 1.727262 | 1.447842 | 0.27942 | 0.888223 |
| TBCE | Homo sapiens tubulin folding cofactor E (TBCE), transcript variant 1, mRNA [NM_001079515] | 2.697327 | 2.417141 | 0.280186 | 0.920846 |
| LTA4H | Homo sapiens leukotriene A4 hydrolase (LTA4H), mRNA [NM_000895] | 4.501046 | 4.220532 | 0.280514 | 0.888223 |
| BCL2L11 | Homo sapiens BCL2-like 11 (apoptosis facilitator) (BCL2L11), transcript variant 1, mRNA [NM_138621] | 1.686964 | 1.405458 | 0.281506 | 0.888223 |
| JUP | Homo sapiens junction plakoglobin (JUP), transcript variant 1, mRNA [NM_002230] | 1.814124 | 1.532287 | 0.281837 | 0.888223 |
| GPRIN1 | Homo sapiens G protein regulated inducer of neurite outgrowth 1 (GPRIN1), mRNA [NM_052899] | 1.733907 | 1.451969 | 0.281938 | 0.888223 |
| RPL22 | Homo sapiens ribosomal protein L22 (RPL22), mRNA [NM_000983] | 1.897001 | 1.613707 | 0.283295 | 0.888223 |
| SPANXB2 | Homo sapiens SPANX family, member B2 (SPANXB2), mRNA [NM_145664] | 1.710077 | 1.426322 | 0.283755 | 0.888223 |
| MYOZ1 | Homo sapiens myozenin 1 (MYOZ1), mRNA [NM_021245] | 1.89355 | 1.608262 | 0.285288 | 0.888223 |
| ZFP36 | Homo sapiens zinc finger protein 36, C3H type, homolog (mouse) (ZFP36), mRNA [NM_003407] | 2.108809 | 1.822386 | 0.286423 | 0.930933 |
| ALOX15B | Homo sapiens arachidonate 15-lipoxygenase, type B (ALOX15B), transcript variant d, mRNA [NM_001141] | 5.324542 | 5.036473 | 0.288069 | 0.918333 |
| MYOCD | Homo sapiens myocardin (MYOCD), transcript variant 3, mRNA [NM_001146313] | 3.049098 | 2.760155 | 0.288943 | 0.956641 |
| MGST1 | Homo sapiens microsomal glutathione S-transferase 1 (MGST1), transcript variant 1c, mRNA [NM_145791] | 1.634295 | 1.344362 | 0.289932 | 0.888223 |
| ANKRD1 | Homo sapiens ankyrin repeat domain 1 (cardiac muscle) (ANKRD1), mRNA [NM_014391] | 1.670815 | 1.380275 | 0.290539 | 0.888223 |
| TDRD5 | Homo sapiens tudor domain containing 5 (TDRD5), transcript variant 3, mRNA [NM_173533] | 2.054721 | 1.76333 | 0.291391 | 0.916588 |
| CELSR1 | Homo sapiens cadherin, EGF LAG seven-pass G-type receptor 1 (flamingo homolog, Drosophila) (CELSR1), mRNA [NM_014246] | 5.030226 | 4.738737 | 0.291488 | 0.969466 |
| FXN | Homo sapiens frataxin (FXN), nuclear gene encoding mitochondrial protein, transcript variant 2, mRNA [NM_181425] | 2.417699 | 2.126149 | 0.29155 | 0.964566 |
| LHFPL5 | Homo sapiens lipoma HMGIC fusion partner-like 5 (LHFPL5), mRNA [NM_182548] | 3.935712 | 3.643773 | 0.291939 | 0.915657 |
| ENST00000410052 | amyotrophic lateral sclerosis 2 (juvenile) [Source:HGNC Symbol;Acc:443] [ENST00000410052] | 1.618993 | 1.326455 | 0.292538 | 0.888223 |
| PLA2G10 | Homo sapiens phospholipase A2, group X (PLA2G10), mRNA [NM_003561] | 1.664174 | 1.371523 | 0.292651 | 0.888223 |
| ADAM17 | Homo sapiens ADAM metallopeptidase domain 17 (ADAM17), mRNA [NM_003183] | 2.645576 | 2.352868 | 0.292708 | 0.922215 |
| TP53 | Homo sapiens tumor protein p53 (TP53), transcript variant 1, mRNA [NM_000546] | 1.630173 | 1.336764 | 0.293409 | 0.888223 |
| EFHD1 | Homo sapiens EF-hand domain family, member D1 (EFHD1), transcript variant 1, mRNA [NM_025202] | 1.824372 | 1.529482 | 0.294889 | 0.888223 |
| NCK2 | Homo sapiens NCK adaptor protein 2 (NCK2), transcript variant 3, mRNA [NM_001004722] | 3.284097 | 2.988576 | 0.295521 | 0.936398 |
| PARD3 | Homo sapiens par-3 partitioning defective 3 homolog (C. elegans) (PARD3), transcript variant 1, mRNA [NM_019619] | 1.706506 | 1.409816 | 0.29669 | 0.888223 |
| LTBP3 | latent transforming growth factor beta binding protein 3 [Source:HGNC Symbol;Acc:6716] [ENST00000525443] | 3.980184 | 3.683062 | 0.297122 | 0.904046 |
| CAMK2B | Homo sapiens calcium/calmodulin-dependent protein kinase II beta (CAMK2B), transcript variant 6, mRNA [NM_172082] | 1.650237 | 1.352288 | 0.29795 | 0.888223 |
| ENST00000404867 | slit homolog 3 (Drosophila) [Source:HGNC Symbol;Acc:11087] [ENST00000404867] | 3.656392 | 3.356936 | 0.299456 | 0.889199 |
| NFKBIA | Homo sapiens nuclear factor of kappa light polypeptide gene enhancer in B-cells inhibitor, alpha (NFKBIA), mRNA [NM_020529] | 3.827433 | 3.527948 | 0.299485 | 0.925014 |
| SOX8 | Homo sapiens SRY (sex determining region Y)-box 8 (SOX8), mRNA [NM_014587] | 2.665626 | 2.365693 | 0.299933 | 0.909019 |
| SPTB | Homo sapiens spectrin, beta, erythrocytic (SPTB), transcript variant 1, mRNA [NM_001024858] | 3.00364 | 2.703304 | 0.300336 | 0.888223 |
| ADRBK1 | Homo sapiens adrenergic, beta, receptor kinase 1 (ADRBK1), mRNA [NM_001619] | 1.637799 | 1.33608 | 0.301719 | 0.888223 |
| PLDN | Homo sapiens pallidin homolog (mouse) (PLDN), mRNA [NM_012388] | 1.634662 | 1.331668 | 0.302995 | 0.888223 |
| ATM | ataxia telangiectasia mutated [Source:HGNC Symbol;Acc:795] [ENST00000389511] | 6.114246 | 5.809842 | 0.304403 | 0.959545 |
| HSP90AB1 | Homo sapiens heat shock protein 90kDa alpha (cytosolic), class B member 1 (HSP90AB1), mRNA [NM_007355] | 1.690321 | 1.385713 | 0.304608 | 0.888223 |
| MYOCD | Homo sapiens myocardin (MYOCD), transcript variant 2, mRNA [NM_153604] | 1.599784 | 1.294956 | 0.304828 | 0.888223 |
| GATA5 | Homo sapiens GATA binding protein 5 (GATA5), mRNA [NM_080473] | 1.736593 | 1.4317 | 0.304893 | 0.888223 |
| AX721082 | phospholipase C, beta 1 (phosphoinositide-specific) [Source:HGNC Symbol;Acc:15917] [ENST00000404098] | 1.879886 | 1.573719 | 0.306167 | 0.888223 |
| WNT11 | Homo sapiens wingless-type MMTV integration site family, member 11 (WNT11), mRNA [NM_004626] | 1.753195 | 1.445976 | 0.30722 | 0.888223 |
| HDAC1 | Homo sapiens histone deacetylase 1 (HDAC1), mRNA [NM_004964] | 1.86385 | 1.555661 | 0.308189 | 0.888223 |
| FOXO4 | Homo sapiens forkhead box O4 (FOXO4), transcript variant 1, mRNA [NM_005938] | 4.183934 | 3.874802 | 0.309132 | 0.888223 |
| VSX1 | Homo sapiens visual system homeobox 1 (VSX1), transcript variant 2, mRNA [NM_199425] | 6.686165 | 6.375566 | 0.310598 | 0.955749 |
| ADNP2 | Homo sapiens ADNP homeobox 2 (ADNP2), mRNA [NM_014913] | 3.198689 | 2.886823 | 0.311866 | 0.895437 |
| BAMBI | Homo sapiens BMP and activin membrane-bound inhibitor homolog (Xenopus laevis) (BAMBI), mRNA [NM_012342] | 2.08612 | 1.772809 | 0.313311 | 0.916669 |
| ENST00000402380 | ataxin 10 [Source:HGNC Symbol;Acc:10549] [ENST00000402380] | 3.61932 | 3.305884 | 0.313436 | 0.890447 |
| MSH2 | Homo sapiens mutS homolog 2, colon cancer, nonpolyposis type 1 (E. coli) (MSH2), mRNA [NM_000251] | 1.782451 | 1.468536 | 0.313915 | 0.892022 |
| SPTBN4 | Homo sapiens spectrin, beta, non-erythrocytic 4 (SPTBN4), transcript variant sigma5, mRNA [NM_025213] | 1.722281 | 1.40732 | 0.314961 | 0.888223 |
| SOS1 | Homo sapiens son of sevenless homolog 1 (Drosophila) (SOS1), mRNA [NM_005633] | 3.514107 | 3.19902 | 0.315087 | 0.932178 |
| GDNF | Homo sapiens glial cell derived neurotrophic factor (GDNF), transcript variant 1, mRNA [NM_000514] | 1.79422 | 1.479073 | 0.315147 | 0.888223 |
| TH | Homo sapiens tyrosine hydroxylase (TH), transcript variant 1, mRNA [NM_199292] | 4.028083 | 3.712613 | 0.315469 | 0.888223 |
| SCHIP1 | Homo sapiens schwannomin interacting protein 1 (SCHIP1), transcript variant 1, mRNA [NM_014575] | 1.76787 | 1.451354 | 0.316516 | 0.888223 |
| RPS6KB1 | Homo sapiens ribosomal protein S6 kinase, 70kDa, polypeptide 1 (RPS6KB1), mRNA [NM_003161] | 2.459111 | 2.141456 | 0.317655 | 0.913505 |
| CCDC88A | Homo sapiens coiled-coil domain containing 88A (CCDC88A), transcript variant 2, mRNA [NM_018084] | 2.76154 | 2.441077 | 0.320463 | 0.94122 |
| TWIST2 | Homo sapiens twist homolog 2 (Drosophila), mRNA (cDNA clone MGC:117334 IMAGE:6021554), complete cds. [BC103755] | 1.692379 | 1.371191 | 0.321188 | 0.888223 |
| KIAA1598 | Homo sapiens KIAA1598 (KIAA1598), transcript variant 2, mRNA [NM_018330] | 1.706936 | 1.384971 | 0.321965 | 0.888223 |
| EPHB3 | Homo sapiens EPH receptor B3 (EPHB3), mRNA [NM_004443] | 3.846635 | 3.523741 | 0.322894 | 0.915657 |
| WEE2 | Homo sapiens WEE1 homolog 2 (S. pombe) (WEE2), mRNA [NM_001105558] | 2.357757 | 2.034409 | 0.323347 | 0.888223 |
| KRAS | Homo sapiens v-Ki-ras2 Kirsten rat sarcoma viral oncogene homolog (KRAS), transcript variant b, mRNA [NM_004985] | 1.7106 | 1.386434 | 0.324166 | 0.888223 |
| CHRNA3 | Homo sapiens cholinergic receptor, nicotinic, alpha 3 (CHRNA3), transcript variant 2, mRNA [NM_001166694] | 2.184749 | 1.860462 | 0.324287 | 0.914946 |
| GHR | Homo sapiens growth hormone receptor (GHR), transcript variant 12, mRNA [NM_001242462] | 1.854472 | 1.529834 | 0.324638 | 0.888223 |
| GPSM2 | Homo sapiens G-protein signaling modulator 2 (GPSM2), mRNA [NM_013296] | 1.772843 | 1.446806 | 0.326037 | 0.888223 |
| NCK1 | Homo sapiens NCK adaptor protein 1 (NCK1), transcript variant 1, mRNA [NM_006153] | 1.766328 | 1.438974 | 0.327354 | 0.888223 |
| EGFR | Homo sapiens epidermal growth factor receptor (EGFR), transcript variant 1, mRNA [NM_005228] | 2.876539 | 2.549141 | 0.327398 | 0.930933 |
| KRT14 | Homo sapiens keratin 14 (KRT14), mRNA [NM_000526] | 1.617662 | 1.289882 | 0.32778 | 0.888223 |
| ISLR2 | Homo sapiens immunoglobulin superfamily containing leucine-rich repeat 2 (ISLR2), transcript variant 1, mRNA [NM_001130136] | 9.239246 | 8.910672 | 0.328573 | 0.888223 |
| TFCP2L1 | Homo sapiens transcription factor CP2-like 1 (TFCP2L1), mRNA [NM_014553] | 4.098865 | 3.769482 | 0.329383 | 0.918021 |
| ENST00000367088 | dynein, light chain, Tctex-type 1 [Source:HGNC Symbol;Acc:11697] [ENST00000367088] | 1.789552 | 1.459897 | 0.329655 | 0.888223 |
| ZEB2 | Homo sapiens zinc finger E-box binding homeobox 2 (ZEB2), transcript variant 3, non-coding RNA [NR_033258] | 1.754756 | 1.423749 | 0.331006 | 0.888223 |
| NEUROD4 | Homo sapiens neurogenic differentiation 4 (NEUROD4), mRNA [NM_021191] | 1.817891 | 1.48653 | 0.331361 | 0.888223 |
| SYNJ1 | Homo sapiens synaptojanin 1 (SYNJ1), transcript variant 2, mRNA [NM_203446] | 2.199252 | 1.867506 | 0.331747 | 0.923157 |
| FOXS1 | Homo sapiens forkhead box S1 (FOXS1), mRNA [NM_004118] | 1.87959 | 1.547346 | 0.332243 | 0.888223 |
| ARNT | Homo sapiens aryl hydrocarbon receptor nuclear translocator (ARNT), transcript variant 1, mRNA [NM_001668] | 1.810519 | 1.478096 | 0.332422 | 0.888223 |
| SOX12 | Homo sapiens SRY (sex determining region Y)-box 12 (SOX12), mRNA [NM_006943] | 2.330372 | 1.997258 | 0.333114 | 0.907771 |
| RFX3 | Homo sapiens regulatory factor X, 3 (influences HLA class II expression) (RFX3), transcript variant 2, mRNA [NM_134428] | 2.506783 | 2.173033 | 0.33375 | 0.913505 |
| TUBB | Homo sapiens tubulin, beta class I (TUBB), mRNA [NM_178014] | 2.158018 | 1.823333 | 0.334684 | 0.922031 |
| TYRO3 | Homo sapiens TYRO3 protein tyrosine kinase (TYRO3), mRNA [NM_006293] | 2.096785 | 1.761912 | 0.334873 | 0.916063 |
| LRRC17 | Homo sapiens leucine rich repeat containing 17 (LRRC17), transcript variant 1, mRNA [NM_001031692] | 1.728662 | 1.393526 | 0.335136 | 0.888223 |
| RXRG | Homo sapiens retinoid X receptor, gamma (RXRG), transcript variant 1, mRNA [NM_006917] | 1.768857 | 1.433457 | 0.335399 | 0.888223 |
| CDC2 | Homo sapiens cyclin-dependent kinase 1 (CDK1), transcript variant 1, mRNA [NM_001786] | 2.263134 | 1.925944 | 0.33719 | 0.888223 |
| PEX7 | Homo sapiens peroxisomal biogenesis factor 7 (PEX7), mRNA [NM_000288] | 2.068321 | 1.730792 | 0.337529 | 0.894389 |
| ROCK2 | Homo sapiens Rho-associated, coiled-coil containing protein kinase 2 (ROCK2), mRNA [NM_004850] | 1.633381 | 1.295652 | 0.337729 | 0.888223 |
| ENST00000395936 | peripheral myelin protein 22 [Source:HGNC Symbol;Acc:9118] [ENST00000395936] | 3.155261 | 2.81751 | 0.337751 | 0.894553 |
| MLL5 | Homo sapiens myeloid/lymphoid or mixed-lineage leukemia 5 (trithorax homolog, Drosophila) (MLL5), transcript variant 1, mRNA [NM_182931] | 1.79365 | 1.455749 | 0.3379 | 0.888223 |
| DGUOK | Homo sapiens deoxyguanosine kinase (DGUOK), nuclear gene encoding mitochondrial protein, transcript variant 1, mRNA [NM_080916] | 2.068643 | 1.729364 | 0.339279 | 0.888223 |
| DRD2 | Homo sapiens dopamine receptor D2 (DRD2), transcript variant 1, mRNA [NM_000795] | 1.700615 | 1.361238 | 0.339378 | 0.888223 |
| CUL7 | Homo sapiens cullin 7 (CUL7), transcript variant 2, mRNA [NM_014780] | 1.955338 | 1.615866 | 0.339471 | 0.899805 |
| CEBPB | Homo sapiens CCAAT/enhancer binding protein (C/EBP), beta (CEBPB), mRNA [NM_005194] | 1.716363 | 1.376171 | 0.340191 | 0.888223 |
| MYADM | Homo sapiens myeloid-associated differentiation marker (MYADM), transcript variant 1, mRNA [NM_001020818] | 2.285387 | 1.944278 | 0.341109 | 0.932009 |
| STK25 | Homo sapiens serine/threonine kinase 25 (STK25), mRNA [NM_006374] | 2.665713 | 2.324358 | 0.341355 | 0.888223 |
| ADAM12 | Homo sapiens ADAM metallopeptidase domain 12 (ADAM12), transcript variant 2, mRNA [NM_021641] | 1.811085 | 1.467717 | 0.343368 | 0.888223 |
| ENST00000402630 | megakaryoblastic leukemia (translocation) 1 [Source:HGNC Symbol;Acc:14334] [ENST00000402630] | 2.115846 | 1.771773 | 0.344073 | 0.918333 |
| DISC1 | Homo sapiens disrupted in schizophrenia 1 (DISC1), transcript variant g, mRNA [NM_001164544] | 1.971269 | 1.627 | 0.344269 | 0.894891 |
| DCC | Homo sapiens deleted in colorectal carcinoma (DCC), mRNA [NM_005215] | 1.710965 | 1.366673 | 0.344292 | 0.888223 |
| SOX3 | Homo sapiens SRY (sex determining region Y)-box 3 (SOX3), mRNA [NM_005634] | 4.671483 | 4.326914 | 0.344568 | 0.921098 |
| APC | Homo sapiens adenomatous polyposis coli (APC), transcript variant 1, mRNA [NM_001127511] | 5.177064 | 4.832289 | 0.344775 | 0.888223 |
| F2 | Homo sapiens coagulation factor II (thrombin) (F2), mRNA [NM_000506] | 2.78862 | 2.442929 | 0.345691 | 0.888223 |
| LGI4 | Homo sapiens leucine-rich repeat LGI family, member 4 (LGI4), mRNA [NM_139284] | 3.493578 | 3.147817 | 0.345761 | 0.944174 |
| BAI1 | Homo sapiens brain-specific angiogenesis inhibitor 1 (BAI1), mRNA [NM_001702] | 3.521192 | 3.174195 | 0.346997 | 0.900258 |
| AACS | Homo sapiens acetoacetyl-CoA synthetase (AACS), mRNA [NM_023928] | 3.017457 | 2.670177 | 0.347281 | 0.909677 |
| PRKG1 | Homo sapiens protein kinase, cGMP-dependent, type I (PRKG1), transcript variant 2, mRNA [NM_006258] | 1.690276 | 1.342065 | 0.34821 | 0.888223 |
| DCT | Homo sapiens dopachrome tautomerase (dopachrome delta-isomerase, tyrosine-related protein 2) (DCT), transcript variant 1, mRNA [NM_001922] | 1.849975 | 1.501752 | 0.348223 | 0.907771 |
| TGFB2 | Homo sapiens transforming growth factor, beta 2 (TGFB2), transcript variant 2, mRNA [NM_003238] | 2.031897 | 1.682557 | 0.34934 | 0.888223 |
| BMP10 | Homo sapiens bone morphogenetic protein 10 (BMP10), mRNA [NM_014482] | 3.525407 | 3.172287 | 0.35312 | 0.888223 |
| ABLIM1 | Homo sapiens actin binding LIM protein 1 (ABLIM1), transcript variant 3, mRNA [NM_001003408] | 2.602227 | 2.248037 | 0.35419 | 0.888223 |
| DOCK2 | Homo sapiens dedicator of cytokinesis 2 (DOCK2), mRNA [NM_004946] | 1.672233 | 1.316277 | 0.355956 | 0.888223 |
| OBSL1 | Homo sapiens obscurin-like 1 (OBSL1), transcript variant 1, mRNA [NM_015311] | 2.346206 | 1.988866 | 0.35734 | 0.888223 |
| CCNE1 | Homo sapiens cyclin E1 (CCNE1), mRNA [NM_001238] | 1.765932 | 1.40589 | 0.360043 | 0.888223 |
| ZFPM2 | Homo sapiens zinc finger protein, multitype 2 (ZFPM2), mRNA [NM_012082] | 2.420834 | 2.059698 | 0.361136 | 0.888223 |
| AP2S1 | Homo sapiens adaptor-related protein complex 2, sigma 1 subunit (AP2S1), transcript variant AP17, mRNA [NM_004069] | 3.444429 | 3.082055 | 0.362374 | 0.951646 |
| COL5A1 | Homo sapiens collagen, type V, alpha 1 (COL5A1), mRNA [NM_000093] | 3.7634 | 3.400432 | 0.362968 | 0.888223 |
| CENPI | Homo sapiens centromere protein I (CENPI), mRNA [NM_006733] | 2.48553 | 2.122171 | 0.36336 | 0.948232 |
| MYH10 | Homo sapiens myosin, heavy chain 10, non-muscle (MYH10), mRNA [NM_005964] | 1.822406 | 1.456561 | 0.365844 | 0.888223 |
| LPPR4 | Homo sapiens lipid phosphate phosphatase-related protein type 4 (LPPR4), transcript variant 1, mRNA [NM_014839] | 1.797595 | 1.430576 | 0.367019 | 0.888223 |
| MED1 | Homo sapiens mediator complex subunit 1 (MED1), mRNA [NM_004774] | 2.221095 | 1.853968 | 0.367128 | 0.888223 |
| CREB3L2 | Homo sapiens cAMP responsive element binding protein 3-like 2 (CREB3L2), mRNA [NM_194071] | 1.690065 | 1.32211 | 0.367956 | 0.888223 |
| ENST00000406114 | luteinizing hormone/choriogonadotropin receptor [Source:HGNC Symbol;Acc:6585] [ENST00000477576] | 1.776113 | 1.406688 | 0.369425 | 0.888223 |
| RASGRP4 | Homo sapiens RAS guanyl releasing protein 4 (RASGRP4), transcript variant a, mRNA [NM_170604] | 2.220824 | 1.851194 | 0.36963 | 0.897441 |
| NLE1 | Homo sapiens notchless homolog 1 (Drosophila) (NLE1), transcript variant 2, mRNA [NM_001014445] | 1.722666 | 1.352466 | 0.3702 | 0.888223 |
| FOXO3 | Homo sapiens forkhead box O3 (FOXO3), transcript variant 1, mRNA [NM_001455] | 1.674961 | 1.304254 | 0.370707 | 0.888223 |
| ANKS1A | Homo sapiens ankyrin repeat and sterile alpha motif domain containing 1A (ANKS1A), mRNA [NM_015245] | 2.773249 | 2.401792 | 0.371457 | 0.888223 |
| RAP2A | Homo sapiens RAP2A, member of RAS oncogene family (RAP2A), mRNA [NM_021033] | 1.75709 | 1.385563 | 0.371527 | 0.888223 |
| SPRR1A | Homo sapiens small proline-rich protein 1A (SPRR1A), transcript variant 2, mRNA [NM_005987] | 5.547585 | 5.175619 | 0.371966 | 0.888223 |
| RBM4 | Homo sapiens RNA binding motif protein 4 (RBM4), transcript variant 3, mRNA [NM_001198844] | 1.793751 | 1.420858 | 0.372893 | 0.888223 |
| ACSBG1 | Homo sapiens acyl-CoA synthetase bubblegum family member 1 (ACSBG1), transcript variant 1, mRNA [NM_015162] | 1.680528 | 1.306271 | 0.374257 | 0.888223 |
| VAV2 | Homo sapiens vav 2 guanine nucleotide exchange factor (VAV2), transcript variant 2, mRNA [NM_003371] | 1.698267 | 1.323896 | 0.374371 | 0.888223 |
| GSTP1 | Homo sapiens glutathione S-transferase pi 1 (GSTP1), mRNA [NM_000852] | 5.840665 | 5.465741 | 0.374924 | 0.888223 |
| DLG2 | Homo sapiens discs, large homolog 2 (Drosophila) (DLG2), transcript variant 2, mRNA [NM_001364] | 2.192394 | 1.816951 | 0.375444 | 0.888223 |
| MDGA2 | Homo sapiens MAM domain containing glycosylphosphatidylinositol anchor 2 (MDGA2), transcript variant 1, mRNA [NM_001113498] | 2.451981 | 2.076149 | 0.375832 | 0.915189 |
| OSGIN2 | Homo sapiens oxidative stress induced growth inhibitor family member 2 (OSGIN2), transcript variant 2, mRNA [NM_004337] | 2.409443 | 2.033577 | 0.375866 | 0.90517 |
| ERBB2 | Homo sapiens v-erb-b2 erythroblastic leukemia viral oncogene homolog 2, neuro/glioblastoma derived oncogene homolog (avian) (ERBB2), transcript variant 2, mRNA [NM_001005862] | 7.586943 | 7.209302 | 0.377641 | 0.888223 |
| SPN | Homo sapiens sialophorin (SPN), transcript variant 1, mRNA [NM_001030288] | 5.761343 | 5.383422 | 0.377921 | 0.888223 |
| IFNA2 | Homo sapiens interferon, alpha 2 (IFNA2), mRNA [NM_000605] | 2.33795 | 1.959935 | 0.378016 | 0.888223 |
| PAX6 | paired box 6 [Source:HGNC Symbol;Acc:8620] [ENST00000379123] | 5.25673 | 4.878018 | 0.378712 | 0.888223 |
| FADS1 | Homo sapiens fatty acid desaturase 1 (FADS1), mRNA [NM_013402] | 2.571072 | 2.192236 | 0.378836 | 0.888223 |
| MTCH1 | Homo sapiens mitochondrial carrier 1 (MTCH1), nuclear gene encoding mitochondrial protein, mRNA [NM_014341] | 3.922655 | 3.543496 | 0.379158 | 0.899014 |
| PTCH1 | Homo sapiens patched 1 (PTCH1), transcript variant 1a, mRNA [NM_001083602] | 2.519565 | 2.139215 | 0.38035 | 0.890141 |
| PURB | Homo sapiens purine-rich element binding protein B (PURB), mRNA [NM_033224] | 4.98983 | 4.607004 | 0.382825 | 0.889199 |
| GRIN2A | Homo sapiens cDNA, FLJ98903. [AK308862] | 2.003814 | 1.620387 | 0.383427 | 0.888223 |
| BARHL1 | Homo sapiens BarH-like homeobox 1 (BARHL1), mRNA [NM_020064] | 2.608931 | 2.2249 | 0.384032 | 0.960183 |
| NOBOX | Homo sapiens NOBOX oogenesis homeobox (NOBOX), mRNA [NM_001080413] | 7.018195 | 6.633 | 0.385195 | 0.926845 |
| AZU1 | Homo sapiens azurocidin 1 (AZU1), mRNA [NM_001700] | 3.245186 | 2.857723 | 0.387463 | 0.888223 |
| CELF4 | Homo sapiens CUGBP, Elav-like family member 4 (CELF4), transcript variant 1, mRNA [NM_020180] | 3.313398 | 2.924233 | 0.389165 | 0.888223 |
| CD83 | Homo sapiens CD83 molecule (CD83), transcript variant 1, mRNA [NM_004233] | 2.103648 | 1.713553 | 0.390094 | 0.888223 |
| RPS6KA2 | Homo sapiens ribosomal protein S6 kinase, 90kDa, polypeptide 2 (RPS6KA2), transcript variant 1, mRNA [NM_021135] | 2.379597 | 1.988325 | 0.391272 | 0.888223 |
| ENST00000406503 | preferentially expressed antigen in melanoma [Source:HGNC Symbol;Acc:9336] [ENST00000406503] | 1.69457 | 1.30308 | 0.39149 | 0.888223 |
| C11orf9 | Homo sapiens chromosome 11 open reading frame 9 (C11orf9), transcript variant 1, mRNA [NM_013279] | 1.728859 | 1.336993 | 0.391865 | 0.888223 |
| TAF8 | Homo sapiens TAF8 RNA polymerase II, TATA box binding protein (TBP)-associated factor, 43kDa (TAF8), mRNA [NM_138572] | 2.35377 | 1.961574 | 0.392196 | 0.888223 |
| PCDH12 | Homo sapiens protocadherin 12 (PCDH12), mRNA [NM_016580] | 1.822295 | 1.429103 | 0.393191 | 0.888223 |
| ECE2 | Homo sapiens endothelin converting enzyme 2 (ECE2), transcript variant 1, mRNA [NM_014693] | 3.731711 | 3.337836 | 0.393875 | 0.929071 |
| NAV1 | Homo sapiens neuron navigator 1 (NAV1), transcript variant 1, mRNA [NM_020443] | 6.65707 | 6.262772 | 0.394297 | 0.888223 |
| KCNE1 | Homo sapiens potassium voltage-gated channel, Isk-related family, member 1 (KCNE1), transcript variant 2, mRNA [NM_000219] | 3.498201 | 3.103898 | 0.394303 | 0.890271 |
| NR4A3 | Homo sapiens nuclear receptor subfamily 4, group A, member 3 (NR4A3), transcript variant 3, mRNA [NM_173200] | 2.872389 | 2.4774 | 0.394989 | 0.890271 |
| A_19_P00316107 | PREDICTED: Homo sapiens hypothetical LOC100505763 (LOC100505763), miscRNA [XR_109604] | 2.869498 | 2.47362 | 0.395878 | 0.936063 |
| PIWIL1 | Homo sapiens piwi-like 1 (Drosophila) (PIWIL1), transcript variant 1, mRNA [NM_004764] | 2.277679 | 1.879015 | 0.398664 | 0.888223 |
| RTN4R | Homo sapiens reticulon 4 receptor (RTN4R), mRNA [NM_023004] | 1.945442 | 1.544653 | 0.400789 | 0.888223 |
| C1S | Homo sapiens complement component 1, s subcomponent (C1S), transcript variant 2, mRNA [NM_001734] | 1.824058 | 1.422617 | 0.401442 | 0.888223 |
| FGFR4 | Homo sapiens fibroblast growth factor receptor 4 (FGFR4), transcript variant 3, mRNA [NM_213647] | 3.502613 | 3.098307 | 0.404305 | 0.900112 |
| NPPC | Homo sapiens natriuretic peptide C (NPPC), mRNA [NM_024409] | 2.499789 | 2.0936 | 0.406189 | 0.888223 |
| DPYSL3 | Homo sapiens dihydropyrimidinase-like 3 (DPYSL3), transcript variant 2, mRNA [NM_001387] | 8.644252 | 8.237695 | 0.406557 | 0.924898 |
| SHROOM3 | shroom family member 3 [Source:HGNC Symbol;Acc:30422] [ENST00000380735] | 1.859471 | 1.450502 | 0.408969 | 0.888223 |
| EIF2B1 | Homo sapiens eukaryotic translation initiation factor 2B, subunit 1 alpha, 26kDa (EIF2B1), mRNA [NM_001414] | 2.741968 | 2.332977 | 0.40899 | 0.888223 |
| EDF1 | Homo sapiens endothelial differentiation-related factor 1 (EDF1), transcript variant beta, mRNA [NM_153200] | 2.47067 | 2.060293 | 0.410377 | 0.888223 |
| MEIS3P1 | Homo sapiens interleukin 6 signal transducer (gp130, oncostatin M receptor) (IL6ST), transcript variant 1, mRNA [NM_002184] | 1.907658 | 1.493729 | 0.413929 | 0.889256 |
| PATZ1 | Homo sapiens POZ (BTB) and AT hook containing zinc finger 1 (PATZ1), transcript variant 4, mRNA [NM_032051] | 6.19011 | 5.776049 | 0.414061 | 0.895701 |
| DEAF1 | Homo sapiens deformed epidermal autoregulatory factor 1 (Drosophila) (DEAF1), mRNA [NM_021008] | 2.794859 | 2.380413 | 0.414446 | 0.888223 |
| HSF4 | Homo sapiens heat shock transcription factor 4 (HSF4), transcript variant 2, mRNA [NM_001040667] | 2.261826 | 1.84735 | 0.414476 | 0.888223 |
| SCUBE1 | signal peptide, CUB domain, EGF-like 1 [Source:HGNC Symbol;Acc:13441] [ENST00000290460] | 2.184259 | 1.766974 | 0.417286 | 0.903999 |
| TMEM204 | Homo sapiens transmembrane protein 204 (TMEM204), mRNA [NM_024600] | 1.847288 | 1.429917 | 0.417371 | 0.888223 |
| NTF4 | Homo sapiens neurotrophin 4 (NTF4), mRNA [NM_006179] | 4.112537 | 3.693669 | 0.418868 | 0.977542 |
| FGF1 | Homo sapiens fibroblast growth factor 1 (acidic) (FGF1), transcript variant 8, non-coding RNA [NR_026696] | 2.046772 | 1.627418 | 0.419354 | 0.888223 |
| FOXI1 | Homo sapiens forkhead box I1 (FOXI1), transcript variant 1, mRNA [NM_012188] | 3.769898 | 3.349325 | 0.420573 | 0.888223 |
| FSHB | Homo sapiens follicle stimulating hormone, beta polypeptide (FSHB), transcript variant 1, mRNA [NM_000510] | 1.855276 | 1.433799 | 0.421477 | 0.888223 |
| CAPRIN2 | Homo sapiens caprin family member 2 (CAPRIN2), transcript variant 1, mRNA [NM_001002259] | 4.680834 | 4.258743 | 0.422091 | 0.888223 |
| SPRR2A | Homo sapiens small proline-rich protein 2A (SPRR2A), mRNA [NM_005988] | 2.753537 | 2.330778 | 0.422758 | 0.899483 |
| ADAMTS20 | Homo sapiens ADAM metallopeptidase with thrombospondin type 1 motif, 20 (ADAMTS20), mRNA [NM_025003] | 1.831982 | 1.408714 | 0.423268 | 0.888223 |
| DOCK2 | Homo sapiens dedicator of cytokinesis 2 (DOCK2), mRNA [NM_004946] | 3.718527 | 3.294816 | 0.423711 | 0.888223 |
| SMARCD3 | Homo sapiens SWI/SNF related, matrix associated, actin dependent regulator of chromatin, subfamily d, member 3 (SMARCD3), transcript variant 2, mRNA [NM_003078] | 4.881267 | 4.457168 | 0.4241 | 0.941333 |
| JUND | Homo sapiens jun D proto-oncogene (JUND), mRNA [NM_005354] | 1.752505 | 1.326906 | 0.425599 | 0.888223 |
| MYCBP2 | Homo sapiens MYC binding protein 2 (MYCBP2), mRNA [NM_015057] | 2.103632 | 1.676579 | 0.427053 | 0.897995 |
| LIF | Homo sapiens leukemia inhibitory factor (cholinergic differentiation factor) (LIF), mRNA [NM_002309] | 1.904943 | 1.477595 | 0.427348 | 0.888223 |
| CDX2 | Homo sapiens caudal type homeobox 2 (CDX2), mRNA [NM_001265] | 6.366684 | 5.937191 | 0.429493 | 0.888223 |
| KIAA1688 | Homo sapiens Rho GTPase activating protein 39 (ARHGAP39), mRNA [NM_025251] | 2.894585 | 2.464989 | 0.429596 | 0.896853 |
| RUNX1T1 | Homo sapiens runt-related transcription factor 1; translocated to, 1 (cyclin D-related) (RUNX1T1), transcript variant 1, mRNA [NM_004349] | 1.734896 | 1.304152 | 0.430743 | 0.888223 |
| SDCBP | Homo sapiens syndecan binding protein (syntenin) (SDCBP), transcript variant 1, mRNA [NM_005625] | 1.802042 | 1.368779 | 0.433263 | 0.888223 |
| TNFRSF12A | Homo sapiens tumor necrosis factor receptor superfamily, member 12A (TNFRSF12A), mRNA [NM_016639] | 4.868937 | 4.435391 | 0.433546 | 0.888223 |
| SHARPIN | Homo sapiens SHANK-associated RH domain interactor (SHARPIN), transcript variant 1, mRNA [NM_030974] | 6.411169 | 5.977448 | 0.433721 | 0.888223 |
| CIT | Homo sapiens citron (rho-interacting, serine/threonine kinase 21) (CIT), transcript variant 1, mRNA [NM_001206999] | 1.837294 | 1.403133 | 0.434162 | 0.888223 |
| CYP19A1 | Homo sapiens cytochrome P450, family 19, subfamily A, polypeptide 1 (CYP19A1), transcript variant 2, mRNA [NM_031226] | 3.106835 | 2.672027 | 0.434808 | 0.895836 |
| TYROBP | Homo sapiens TYRO protein tyrosine kinase binding protein (TYROBP), transcript variant 1, mRNA [NM_003332] | 4.189944 | 3.754903 | 0.435041 | 0.888223 |
| HDAC3 | Homo sapiens histone deacetylase 3 (HDAC3), mRNA [NM_003883] | 1.763815 | 1.328671 | 0.435144 | 0.888223 |
| LST1 | Homo sapiens leukocyte specific transcript 1 (LST1), transcript variant 1, mRNA [NM_007161] | 4.010824 | 3.574709 | 0.436115 | 0.888223 |
| NOTCH4 | Homo sapiens notch 4 (NOTCH4), mRNA [NM_004557] | 1.974662 | 1.538153 | 0.436509 | 0.888223 |
| CD28 | Homo sapiens CD28 molecule (CD28), transcript variant 1, mRNA [NM_006139] | 4.704023 | 4.267285 | 0.436738 | 0.888223 |
| CAMK1 | Homo sapiens calcium/calmodulin-dependent protein kinase I (CAMK1), mRNA [NM_003656] | 1.901796 | 1.464527 | 0.437269 | 0.888223 |
| EPB41L5 | Homo sapiens erythrocyte membrane protein band 4.1 like 5 (EPB41L5), transcript variant 3, mRNA [NM_001184938] | 1.756138 | 1.317986 | 0.438151 | 0.888223 |
| NRARP | Homo sapiens NOTCH-regulated ankyrin repeat protein (NRARP), mRNA [NM_001004354] | 1.834369 | 1.395805 | 0.438565 | 0.888223 |
| GPRIN1 | Homo sapiens G protein regulated inducer of neurite outgrowth 1 (GPRIN1), mRNA [NM_052899] | 4.618563 | 4.177957 | 0.440606 | 0.912315 |
| TWF2 | Homo sapiens twinfilin, actin-binding protein, homolog 2 (Drosophila) (TWF2), mRNA [NM_007284] | 1.863988 | 1.423069 | 0.44092 | 0.888223 |
| NEUROG1 | Homo sapiens neurogenin 1 (NEUROG1), mRNA [NM_006161] | 2.331315 | 1.88967 | 0.441645 | 0.888223 |
| CITED2 | Homo sapiens Cbp/p300-interacting transactivator, with Glu/Asp-rich carboxy-terminal domain, 2 (CITED2), transcript variant 1, mRNA [NM_006079] | 3.468432 | 3.026647 | 0.441785 | 0.895728 |
| MAMSTR | Homo sapiens MEF2 activating motif and SAP domain containing transcriptional regulator (MAMSTR), transcript variant 2, mRNA [NM_182574] | 7.330029 | 6.885431 | 0.444598 | 0.888223 |
| MEF2C | Homo sapiens myocyte enhancer factor 2C (MEF2C), transcript variant 1, mRNA [NM_002397] | 5.65052 | 5.205785 | 0.444734 | 0.888223 |
| GSN | Homo sapiens gelsolin (GSN), transcript variant 2, mRNA [NM_198252] | 1.769434 | 1.323419 | 0.446016 | 0.888223 |
| ETV1 | Homo sapiens ets variant 1 (ETV1), transcript variant 1, mRNA [NM_004956] | 1.903344 | 1.45675 | 0.446594 | 0.888223 |
| GPC2 | Homo sapiens glypican 2 (GPC2), mRNA [NM_152742] | 3.340071 | 2.892319 | 0.447752 | 0.919444 |
| ANXA1 | Homo sapiens annexin A1 (ANXA1), mRNA [NM_000700] | 1.818889 | 1.369065 | 0.449824 | 0.888223 |
| DAB2IP | Homo sapiens DAB2 interacting protein (DAB2IP), transcript variant 1, mRNA [NM_032552] | 2.271993 | 1.821095 | 0.450898 | 0.888223 |
| BMP2 | Homo sapiens bone morphogenetic protein 2 (BMP2), mRNA [NM_001200] | 1.852649 | 1.401692 | 0.450957 | 0.888223 |
| KLF6 | Homo sapiens Kruppel-like factor 6 (KLF6), transcript variant A, mRNA [NM_001300] | 4.008026 | 3.556218 | 0.451808 | 0.888223 |
| VSX1 | visual system homeobox 1 [Source:HGNC Symbol;Acc:12723] [ENST00000409285] | 2.080833 | 1.628905 | 0.451928 | 0.888223 |
| ENST00000342207 | PTK2 protein tyrosine kinase 2 [Source:HGNC Symbol;Acc:9611] [ENST00000342207] | 3.295955 | 2.842931 | 0.453025 | 0.941588 |
| RPS19 | Homo sapiens ribosomal protein S19 (RPS19), mRNA [NM_001022] | 1.864629 | 1.411506 | 0.453123 | 0.888223 |
| PLCB1 | Homo sapiens phospholipase C, beta 1 (phosphoinositide-specific) (PLCB1), transcript variant 1, mRNA [NM_015192] | 1.892847 | 1.439317 | 0.45353 | 0.888223 |
| LILRB3 | Homo sapiens leukocyte immunoglobulin-like receptor, subfamily B (with TM and ITIM domains), member 3 (LILRB3), transcript variant 2, mRNA [NM_006864] | 2.657197 | 2.203575 | 0.453622 | 0.951513 |
| CDH4 | Homo sapiens cadherin 4, type 1, R-cadherin (retinal) (CDH4), transcript variant 1, mRNA [NM_001794] | 6.060136 | 5.605027 | 0.45511 | 0.888223 |
| TDRD5 | Homo sapiens tudor domain containing 5 (TDRD5), transcript variant 1, mRNA [NM_001199085] | 1.957599 | 1.501964 | 0.455635 | 0.888223 |
| SPDEF | Homo sapiens SAM pointed domain containing ets transcription factor (SPDEF), transcript variant 1, mRNA [NM_012391] | 4.071198 | 3.610463 | 0.460734 | 0.888223 |
| TLR3 | Homo sapiens toll-like receptor 3 (TLR3), mRNA [NM_003265] | 1.777848 | 1.311893 | 0.465955 | 0.888223 |
| EXT1 | Homo sapiens exostosin 1 (EXT1), mRNA [NM_000127] | 1.894191 | 1.427882 | 0.466309 | 0.888223 |
| NRARP | Homo sapiens NOTCH-regulated ankyrin repeat protein (NRARP), mRNA [NM_001004354] | 1.90636 | 1.439295 | 0.467065 | 0.888223 |
| TRAF6 | Homo sapiens TNF receptor-associated factor 6 (TRAF6), transcript variant 1, mRNA [NM_145803] | 2.022168 | 1.554335 | 0.467832 | 0.888223 |
| HOXA11 | Homo sapiens homeobox A11 (HOXA11), mRNA [NM_005523] | 1.97692 | 1.506989 | 0.46993 | 0.888223 |
| PIWIL2 | Homo sapiens piwi-like 2 (Drosophila) (PIWIL2), transcript variant 2, mRNA [NM_018068] | 4.822246 | 4.351313 | 0.470932 | 0.897995 |
| TPT1 | Homo sapiens tumor protein, translationally-controlled 1 (TPT1), mRNA [NM_003295] | 4.034836 | 3.562566 | 0.47227 | 0.950446 |
| POLM | Homo sapiens polymerase (DNA directed), mu (POLM), mRNA [NM_013284] | 2.573296 | 2.100173 | 0.473123 | 0.888223 |
| C10orf49 | Homo sapiens upper zone of growth plate and cartilage matrix associated (UCMA), mRNA [NM_145314] | 1.937001 | 1.461834 | 0.475167 | 0.888223 |
| TULP1 | Homo sapiens tubby like protein 1 (TULP1), mRNA [NM_003322] | 2.759318 | 2.283932 | 0.475386 | 0.888223 |
| RBP1 | Homo sapiens retinol binding protein 1, cellular (RBP1), transcript variant 1, mRNA [NM_002899] | 2.302831 | 1.822298 | 0.480533 | 0.925408 |
| GPC1 | Homo sapiens glypican 1 (GPC1), mRNA [NM_002081] | 2.860584 | 2.379667 | 0.480917 | 0.888223 |
| NCOA6 | Homo sapiens nuclear receptor coactivator 6 (NCOA6), transcript variant 1, mRNA [NM_014071] | 1.886633 | 1.405676 | 0.480957 | 0.888223 |
| VCAN | Homo sapiens versican (VCAN), transcript variant 1, mRNA [NM_004385] | 1.848334 | 1.366427 | 0.481907 | 0.888223 |
| BCL9L | Homo sapiens B-cell CLL/lymphoma 9-like (BCL9L), mRNA [NM_182557] | 2.162847 | 1.680824 | 0.482024 | 0.888223 |
| PVRL2 | Homo sapiens poliovirus receptor-related 2 (herpesvirus entry mediator B) (PVRL2), transcript variant delta, mRNA [NM_001042724] | 2.036182 | 1.553999 | 0.482183 | 0.888223 |
| TFAP2C | Homo sapiens transcription factor AP-2 gamma (activating enhancer binding protein 2 gamma) (TFAP2C), mRNA [NM_003222] | 3.131183 | 2.648383 | 0.4828 | 0.888223 |
| ALS2 | Homo sapiens amyotrophic lateral sclerosis 2 (juvenile) (ALS2), transcript variant 2, mRNA [NM_001135745] | 1.958754 | 1.475927 | 0.482826 | 0.888223 |
| FSHR | Homo sapiens follicle stimulating hormone receptor (FSHR), transcript variant 1, mRNA [NM_000145] | 1.879943 | 1.396885 | 0.483058 | 0.888223 |
| FXN | Homo sapiens frataxin (FXN), nuclear gene encoding mitochondrial protein, transcript variant 2, mRNA [NM_181425] | 2.21299 | 1.728733 | 0.484258 | 0.888223 |
| TACC3 | Homo sapiens transforming, acidic coiled-coil containing protein 3 (TACC3), mRNA [NM_006342] | 2.229231 | 1.743221 | 0.486011 | 0.888223 |
| ENST00000422388 | SLIT and NTRK-like family, member 2 [Source:HGNC Symbol;Acc:13449] [ENST00000370490] | 1.819145 | 1.332531 | 0.486614 | 0.888223 |
| MYADML2 | Homo sapiens myeloid-associated differentiation marker-like 2 (MYADML2), mRNA [NM_001145113] | 2.185126 | 1.698076 | 0.48705 | 0.888223 |
| SIX5 | Homo sapiens SIX homeobox 5 (SIX5), mRNA [NM_175875] | 2.250334 | 1.761408 | 0.488926 | 0.888223 |
| TCHH | Homo sapiens trichohyalin (TCHH), mRNA [NM_007113] | 3.155899 | 2.665227 | 0.490672 | 0.9031 |
| TRPC6 | Homo sapiens transient receptor potential cation channel, subfamily C, member 6 (TRPC6), mRNA [NM_004621] | 14.70492 | 14.21165 | 0.493262 | 0.888223 |
| CSF1 | Homo sapiens colony stimulating factor 1 (macrophage) (CSF1), transcript variant 1, mRNA [NM_000757] | 3.288655 | 2.791647 | 0.497008 | 0.902599 |
| IMPAD1 | Homo sapiens inositol monophosphatase domain containing 1 (IMPAD1), mRNA [NM_017813] | 1.817338 | 1.320268 | 0.49707 | 0.888223 |
| NCAM1 | Homo sapiens neural cell adhesion molecule 1 (NCAM1), transcript variant 3, mRNA [NM_001076682] | 2.012943 | 1.51573 | 0.497213 | 0.888223 |
| INPPL1 | Homo sapiens inositol polyphosphate phosphatase-like 1 (INPPL1), mRNA [NM_001567] | 1.811686 | 1.313978 | 0.497708 | 0.888223 |
| TIRAP | Homo sapiens toll-interleukin 1 receptor (TIR) domain containing adaptor protein (TIRAP), transcript variant 3, mRNA [NM_001039661] | 2.217471 | 1.719534 | 0.497937 | 0.888223 |
| EOMES | Homo sapiens eomesodermin (EOMES), mRNA [NM_005442] | 1.841905 | 1.342853 | 0.499052 | 0.888223 |
| CD4 | Unknown | 3.331568 | 2.831893 | 0.499676 | 0.898947 |
| AGER | Homo sapiens advanced glycosylation end product-specific receptor (AGER), transcript variant 9, mRNA [NM_001206966] | 5.683586 | 5.183603 | 0.499982 | 0.888223 |
| CLCF1 | Homo sapiens cardiotrophin-like cytokine factor 1 (CLCF1), transcript variant 1, mRNA [NM_013246] | 4.097249 | 3.595677 | 0.501572 | 0.888223 |
| CAMK4 | Homo sapiens calcium/calmodulin-dependent protein kinase IV (CAMK4), mRNA [NM_001744] | 3.50471 | 3.003005 | 0.501705 | 0.888223 |
| SHARPIN | Homo sapiens SHANK-associated RH domain interactor (SHARPIN), transcript variant 1, mRNA [NM_030974] | 6.324309 | 5.820693 | 0.503616 | 0.888223 |
| EFNA5 | Homo sapiens ephrin-A5 (EFNA5), mRNA [NM_001962] | 3.444287 | 2.940603 | 0.503684 | 0.888223 |
| MAFB | Homo sapiens v-maf musculoaponeurotic fibrosarcoma oncogene homolog B (avian) (MAFB), mRNA [NM_005461] | 3.257636 | 2.751654 | 0.505982 | 0.888223 |
| GPR55 | Homo sapiens G protein-coupled receptor 55 (GPR55), mRNA [NM_005683] | 1.846826 | 1.339345 | 0.507481 | 0.888223 |
| TNNI3 | Homo sapiens troponin I type 3 (cardiac) (TNNI3), mRNA [NM_000363] | 1.856814 | 1.349234 | 0.50758 | 0.888223 |
| ENST00000381985 | regulatory factor X, 3 (influences HLA class II expression) [Source:HGNC Symbol;Acc:9984] [ENST00000381985] | 1.991039 | 1.482625 | 0.508414 | 0.888223 |
| METRNL | Homo sapiens meteorin, glial cell differentiation regulator-like (METRNL), mRNA [NM_001004431] | 3.282595 | 2.773784 | 0.508811 | 0.937776 |
| FOXF2 | Homo sapiens forkhead box F2 (FOXF2), mRNA [NM_001452] | 4.125352 | 3.615591 | 0.509761 | 0.888223 |
| SGK493 | Homo sapiens protein kinase domain containing, cytoplasmic homolog (mouse) (PKDCC), mRNA [NM_138370] | 4.115301 | 3.605064 | 0.510237 | 0.888223 |
| AK124606 | Homo sapiens cDNA FLJ42615 fis, clone BRACE3014231. [AK124606] | 2.107076 | 1.596411 | 0.510664 | 0.888223 |
| MSI2 | Homo sapiens musashi homolog 2 (Drosophila) (MSI2), transcript variant 1, mRNA [NM_138962] | 1.857535 | 1.346404 | 0.511131 | 0.888223 |
| DLG2 | Homo sapiens discs, large homolog 2 (Drosophila) (DLG2), transcript variant 3, mRNA [NM_001142700] | 2.764333 | 2.252297 | 0.512036 | 0.900741 |
| MYL9 | Homo sapiens myosin, light chain 9, regulatory (MYL9), transcript variant 2, mRNA [NM_181526] | 4.187968 | 3.675462 | 0.512506 | 0.888223 |
| RS1 | Homo sapiens cyclin-dependent kinase-like 5 (CDKL5), transcript variant I, mRNA [NM_003159] | 2.191278 | 1.67723 | 0.514048 | 0.888223 |
| TRPS1 | Homo sapiens trichorhinophalangeal syndrome I (TRPS1), mRNA [NM_014112] | 7.586144 | 7.071756 | 0.514389 | 0.888223 |
| DZIP1 | Homo sapiens DAZ interacting protein 1 (DZIP1), transcript variant 1, mRNA [NM_014934] | 2.805244 | 2.290523 | 0.514722 | 0.888223 |
| S100A6 | Homo sapiens S100 calcium binding protein A6 (S100A6), mRNA [NM_014624] | 2.046279 | 1.531308 | 0.514971 | 0.888223 |
| SCIN | Homo sapiens scinderin (SCIN), transcript variant 2, mRNA [NM_033128] | 2.102393 | 1.587216 | 0.515178 | 0.888223 |
| IGF1 | Homo sapiens insulin-like growth factor 1 (somatomedin C) (IGF1), transcript variant 4, mRNA [NM_000618] | 4.914774 | 4.399416 | 0.515359 | 0.888223 |
| KIAA1598 | Homo sapiens KIAA1598 (KIAA1598), transcript variant 1, mRNA [NM_001127211] | 4.244915 | 3.729438 | 0.515477 | 0.888223 |
| TGFB3 | Homo sapiens transforming growth factor, beta 3 (TGFB3), mRNA [NM_003239] | 2.271111 | 1.755116 | 0.515995 | 0.888223 |
| IRX3 | Homo sapiens iroquois homeobox 3 (IRX3), mRNA [NM_024336] | 1.986388 | 1.469512 | 0.516876 | 0.888223 |
| ZHX2 | Homo sapiens zinc fingers and homeoboxes 2 (ZHX2), mRNA [NM_014943] | 1.887978 | 1.369159 | 0.518818 | 0.888223 |
| ARX | Homo sapiens aristaless related homeobox (ARX), mRNA [NM_139058] | 1.980085 | 1.461178 | 0.518907 | 0.888223 |
| PDLIM5 | Homo sapiens PDZ and LIM domain 5 (PDLIM5), transcript variant 4, mRNA [NM_001011515] | 2.278463 | 1.757437 | 0.521027 | 0.888223 |
| AGTPBP1 | Homo sapiens ATP/GTP binding protein 1 (AGTPBP1), mRNA [NM_015239] | 1.920454 | 1.399008 | 0.521447 | 0.888223 |
| GDAP1L1 | Homo sapiens ganglioside-induced differentiation-associated protein 1-like 1 (GDAP1L1), mRNA [NM_024034] | 1.961197 | 1.436003 | 0.525194 | 0.888223 |
| UNC5B | Homo sapiens unc-5 homolog B (C. elegans) (UNC5B), transcript variant 1, mRNA [NM_170744] | 2.996236 | 2.47092 | 0.525316 | 0.888621 |
| PUS10 | Homo sapiens peroxisomal biogenesis factor 13 (PEX13), mRNA [NM_002618] | 2.46969 | 1.942485 | 0.527206 | 0.888223 |
| NR2E1 | Homo sapiens nuclear receptor subfamily 2, group E, member 1 (NR2E1), mRNA [NM_003269] | 5.555366 | 5.025676 | 0.529689 | 0.888223 |
| ATM | Homo sapiens ataxia telangiectasia mutated (ATM), mRNA [NM_000051] | 2.960547 | 2.430707 | 0.52984 | 0.888223 |
| FLJ36070 | Homo sapiens MEF2 activating motif and SAP domain containing transcriptional regulator (MAMSTR), transcript variant 2, mRNA [NM_182574] | 5.24577 | 4.71323 | 0.532539 | 0.888223 |
| MREG | Homo sapiens melanoregulin (MREG), mRNA [NM_018000] | 1.845852 | 1.313263 | 0.532588 | 0.888223 |
| NELL1 | NEL-like 1 (chicken) [Source:HGNC Symbol;Acc:7750] [ENST00000534263] | 5.859772 | 5.324275 | 0.535497 | 0.94082 |
| ABLIM3 | Homo sapiens actin binding LIM protein family, member 3 (ABLIM3), mRNA [NM_014945] | 6.384769 | 5.84857 | 0.536199 | 0.888223 |
| BCL2L11 | Homo sapiens BCL2-like 11 (apoptosis facilitator) (BCL2L11), transcript variant 9, mRNA [NM_207002] | 2.322931 | 1.785831 | 0.5371 | 0.895437 |
| FLG | Homo sapiens filaggrin (FLG), mRNA [NM_002016] | 2.388785 | 1.851556 | 0.537228 | 0.888223 |
| STX2 | Homo sapiens syntaxin 2 (STX2), transcript variant 1, mRNA [NM_001980] | 2.628122 | 2.089819 | 0.538304 | 0.888223 |
| CACYBP | Homo sapiens calcyclin binding protein (CACYBP), transcript variant 1, mRNA [NM_014412] | 3.580927 | 3.040713 | 0.540214 | 0.888223 |
| TMOD1 | Homo sapiens tropomodulin 1 (TMOD1), transcript variant 1, mRNA [NM_003275] | 2.328712 | 1.7879 | 0.540812 | 0.888223 |
| FOXE3 | Homo sapiens forkhead box E3 (FOXE3), mRNA [NM_012186] | 4.310162 | 3.768126 | 0.542036 | 0.888223 |
| RELN | Homo sapiens reelin (RELN), transcript variant 1, mRNA [NM_005045] | 2.915739 | 2.371377 | 0.544362 | 0.90962 |
| TDRD1 | Homo sapiens tudor domain containing 1 (TDRD1), mRNA [NM_198795] | 2.948629 | 2.403824 | 0.544805 | 0.888223 |
| ESRRA | Homo sapiens estrogen-related receptor alpha (ESRRA), mRNA [NM_004451] | 4.258826 | 3.712966 | 0.54586 | 0.888223 |
| COL5A1 | collagen, type V, alpha 1 [Source:HGNC Symbol;Acc:2209] [ENST00000464187] | 2.313899 | 1.76678 | 0.547119 | 0.888223 |
| PLXNB2 | Homo sapiens plexin B2 (PLXNB2), mRNA [NM_012401] | 5.37913 | 4.83057 | 0.54856 | 0.888223 |
| CACNA1G | Homo sapiens calcium channel, voltage-dependent, T type, alpha 1G subunit (CACNA1G), transcript variant 1, mRNA [NM_018896] | 1.84516 | 1.29535 | 0.54981 | 0.888223 |
| GRLF1 | Homo sapiens Rho GTPase activating protein 35 (ARHGAP35), mRNA [NM_004491] | 2.079276 | 1.527698 | 0.551578 | 0.888223 |
| TBX6 | Homo sapiens T-box 6 (TBX6), mRNA [NM_004608] | 5.015581 | 4.462316 | 0.553266 | 0.888223 |
| LHX8 | Homo sapiens LIM homeobox 8 (LHX8), mRNA [NM_001001933] | 1.883793 | 1.329998 | 0.553795 | 0.888223 |
| LCE2A | Homo sapiens late cornified envelope 2A (LCE2A), mRNA [NM_178428] | 1.967945 | 1.413387 | 0.554558 | 0.888223 |
| MOV10L1 | Homo sapiens Mov10l1, Moloney leukemia virus 10-like 1, homolog (mouse) (MOV10L1), transcript variant 1, mRNA [NM_018995] | 2.07641 | 1.517394 | 0.559016 | 0.888223 |
| CCL19 | Homo sapiens chemokine (C-C motif) ligand 19 (CCL19), mRNA [NM_006274] | 1.997086 | 1.436151 | 0.560934 | 0.888223 |
| LOC100133047 | tumor protein D52 [Source:HGNC Symbol;Acc:12005] [ENST00000523564] | 1.908628 | 1.346159 | 0.562468 | 0.888223 |
| EDNRB | Homo sapiens endothelin receptor type B (EDNRB), transcript variant 2, mRNA [NM_003991] | 3.023741 | 2.459737 | 0.564004 | 0.888223 |
| PPARA | Homo sapiens peroxisome proliferator-activated receptor alpha (PPARA), transcript variant 5, mRNA [NM_005036] | 2.058488 | 1.492128 | 0.56636 | 0.888223 |
| CD276 | Homo sapiens CD276 molecule (CD276), transcript variant 1, mRNA [NM_001024736] | 4.558214 | 3.990069 | 0.568145 | 0.888223 |
| TTC3 | Homo sapiens tetratricopeptide repeat domain 3 (TTC3), transcript variant 1, mRNA [NM_003316] | 2.574481 | 2.00573 | 0.568751 | 0.888223 |
| TNFSF11 | Homo sapiens tumor necrosis factor (ligand) superfamily, member 11 (TNFSF11), transcript variant 1, mRNA [NM_003701] | 5.639854 | 5.069675 | 0.570179 | 0.888223 |
| VANGL2 | Homo sapiens vang-like 2 (van gogh, Drosophila) (VANGL2), mRNA [NM_020335] | 2.005309 | 1.43456 | 0.570749 | 0.888223 |
| LDB2 | Homo sapiens LIM domain binding 2 (LDB2), transcript variant 1, mRNA [NM_001290] | 5.124756 | 4.551582 | 0.573174 | 0.888223 |
| A_19_P00808033 | Homo sapiens striatin, calmodulin binding protein (STRN), mRNA [NM_003162] | 2.692534 | 2.118156 | 0.574378 | 0.888223 |
| PLXNA3 | Homo sapiens plexin A3 (PLXNA3), mRNA [NM_017514] | 2.207344 | 1.63034 | 0.577004 | 0.888223 |
| NPY | Homo sapiens neuropeptide Y (NPY), mRNA [NM_000905] | 4.299127 | 3.72168 | 0.577447 | 0.888223 |
| CUX1 | Homo sapiens cut-like homeobox 1 (CUX1), transcript variant 4, mRNA [NM_001202543] | 4.524676 | 3.945496 | 0.57918 | 0.914567 |
| LAMA4 | Homo sapiens laminin, alpha 4 (LAMA4), transcript variant 3, mRNA [NM_001105207] | 2.08432 | 1.502517 | 0.581803 | 0.888223 |
| NRXN1 | Homo sapiens neurexin 1 (NRXN1), transcript variant alpha2, mRNA [NM_001135659] | 3.413678 | 2.831397 | 0.582281 | 0.888223 |
| ADIPOQ | Homo sapiens adiponectin, C1Q and collagen domain containing (ADIPOQ), transcript variant 2, mRNA [NM_004797] | 3.230644 | 2.647793 | 0.582851 | 0.888223 |
| BCCIP | Homo sapiens BRCA2 and CDKN1A interacting protein (BCCIP), transcript variant B, mRNA [NM_078468] | 2.131172 | 1.547202 | 0.58397 | 0.888223 |
| NIPBL | Homo sapiens Nipped-B homolog (Drosophila) (NIPBL), transcript variant A, mRNA [NM_133433] | 1.907475 | 1.322591 | 0.584884 | 0.888223 |
| PLAUR | Homo sapiens plasminogen activator, urokinase receptor (PLAUR), transcript variant 3, mRNA [NM_001005377] | 2.013501 | 1.427502 | 0.585999 | 0.888223 |
| HMGA2 | Homo sapiens high mobility group AT-hook 2 (HMGA2), transcript variant 1, mRNA [NM_003483] | 2.430124 | 1.843589 | 0.586535 | 0.888223 |
| P2RY2 | Homo sapiens purinergic receptor P2Y, G-protein coupled, 2 (P2RY2), transcript variant 1, mRNA [NM_176072] | 1.959787 | 1.372978 | 0.586809 | 0.888223 |
| HDAC2 | Homo sapiens histone deacetylase 2 (HDAC2), transcript variant 1, mRNA [NM_001527] | 2.529701 | 1.942555 | 0.587146 | 0.888223 |
| B3GNT2 | Homo sapiens UDP-GlcNAc:betaGal beta-1,3-N-acetylglucosaminyltransferase 2 (B3GNT2), mRNA [NM_006577] | 1.996877 | 1.409201 | 0.587676 | 0.888223 |
| VNN1 | Homo sapiens vanin 1 (VNN1), mRNA [NM_004666] | 2.010375 | 1.421699 | 0.588676 | 0.888223 |
| DLG2 | Homo sapiens discs, large homolog 2 (Drosophila) (DLG2), transcript variant 2, mRNA [NM_001364] | 2.396979 | 1.807659 | 0.58932 | 0.888223 |
| ONECUT2 | Homo sapiens one cut homeobox 2 (ONECUT2), mRNA [NM_004852] | 2.153291 | 1.562984 | 0.590307 | 0.888223 |
| RETN | Homo sapiens resistin (RETN), transcript variant 1, mRNA [NM_020415] | 5.801871 | 5.21077 | 0.591102 | 0.888223 |
| ENST00000367388 | LIM homeobox 9 [Source:HGNC Symbol;Acc:14222] [ENST00000367388] | 4.880643 | 4.289342 | 0.591301 | 0.888223 |
| IFRD1 | Homo sapiens interferon-related developmental regulator 1 (IFRD1), transcript variant 2, mRNA [NM_001007245] | 2.23892 | 1.647378 | 0.591541 | 0.888223 |
| NEUROD2 | Homo sapiens neurogenic differentiation 2 (NEUROD2), mRNA [NM_006160] | 2.007979 | 1.416403 | 0.591576 | 0.888223 |
| ANAPC2 | Homo sapiens anaphase promoting complex subunit 2 (ANAPC2), mRNA [NM_013366] | 2.005664 | 1.411897 | 0.593767 | 0.888223 |
| GAB2 | Homo sapiens GRB2-associated binding protein 2 (GAB2), transcript variant 2, mRNA [NM_012296] | 4.103831 | 3.50883 | 0.595001 | 0.888223 |
| COL4A1 | Homo sapiens collagen, type IV, alpha 1 (COL4A1), mRNA [NM_001845] | 6.728603 | 6.133561 | 0.595042 | 0.913505 |
| ABLIM2 | Homo sapiens actin binding LIM protein family, member 2 (ABLIM2), transcript variant 7, mRNA [NM_001130088] | 3.286947 | 2.690691 | 0.596256 | 0.888223 |
| DND1 | Homo sapiens dead end homolog 1 (zebrafish) (DND1), mRNA [NM_194249] | 4.819998 | 4.222672 | 0.597326 | 0.888223 |
| CLASP2 | Homo sapiens cytoplasmic linker associated protein 2 (CLASP2), transcript variant 1, mRNA [NM_015097] | 2.30101 | 1.703539 | 0.597471 | 0.888223 |
| POU4F3 | Homo sapiens POU class 4 homeobox 3 (POU4F3), mRNA [NM_002700] | 2.441111 | 1.843273 | 0.597838 | 0.888223 |
| MTPN | Homo sapiens myotrophin (MTPN), mRNA [NM_145808] | 1.885616 | 1.28625 | 0.599367 | 0.888223 |
| NKX6-2 | Homo sapiens NK6 homeobox 2 (NKX6-2), mRNA [NM_177400] | 2.373267 | 1.773424 | 0.599843 | 0.888363 |
| COL9A2 | Homo sapiens collagen, type IX, alpha 2 (COL9A2), mRNA [NM_001852] | 2.503208 | 1.902856 | 0.600351 | 0.888223 |
| MAEA | Homo sapiens macrophage erythroblast attacher (MAEA), transcript variant 1, mRNA [NM_001017405] | 1.917235 | 1.316635 | 0.600599 | 0.888223 |
| SIX1 | Homo sapiens SIX homeobox 1 (SIX1), mRNA [NM_005982] | 4.826278 | 4.22309 | 0.603189 | 0.888223 |
| DLX1 | Homo sapiens distal-less homeobox 1 (DLX1), transcript variant 1, mRNA [NM_178120] | 2.392715 | 1.78881 | 0.603905 | 0.888223 |
| A_19_P00318531 | Homo sapiens hook homolog 3 (Drosophila) (HOOK3), mRNA [NM_032410] | 2.213234 | 1.609211 | 0.604023 | 0.888223 |
| BLNK | Homo sapiens B-cell linker (BLNK), transcript variant 1, mRNA [NM_013314] | 2.230866 | 1.625084 | 0.605782 | 0.888223 |
| PRDM16 | Homo sapiens PR domain containing 16 (PRDM16), transcript variant 1, mRNA [NM_022114] | 2.692463 | 2.085401 | 0.607062 | 0.888223 |
| FOXG1 | Homo sapiens forkhead box G1 (FOXG1), mRNA [NM_005249] | 3.458262 | 2.849882 | 0.60838 | 0.888223 |
| FOXP1 | Homo sapiens forkhead box P1 (FOXP1), transcript variant 1, mRNA [NM_032682] | 3.066413 | 2.45695 | 0.609463 | 0.888223 |
| THC2628839 | protein kinase domain containing, cytoplasmic homolog (mouse) [Source:HGNC Symbol;Acc:25123] [ENST00000485578] | 2.218013 | 1.60798 | 0.610032 | 0.888223 |
| NR2F2 | Homo sapiens nuclear receptor subfamily 2, group F, member 2 (NR2F2), transcript variant 1, mRNA [NM_021005] | 2.818832 | 2.207769 | 0.611063 | 0.888223 |
| KIF13B | Homo sapiens kinesin family member 13B (KIF13B), mRNA [NM_015254] | 2.371791 | 1.760361 | 0.611429 | 0.888223 |
| MOV10L1 | Homo sapiens Mov10l1, Moloney leukemia virus 10-like 1, homolog (mouse) (MOV10L1), transcript variant 1, mRNA [NM_018995] | 4.524829 | 3.911436 | 0.613394 | 0.888223 |
| FABP9 | Homo sapiens fatty acid binding protein 9, testis (FABP9), mRNA [NM_001080526] | 2.508142 | 1.894615 | 0.613527 | 0.900609 |
| EIF2B3 | Homo sapiens eukaryotic translation initiation factor 2B, subunit 3 gamma, 58kDa (EIF2B3), transcript variant 1, mRNA [NM_020365] | 4.124834 | 3.511111 | 0.613722 | 0.888223 |
| FLG | Homo sapiens filaggrin (FLG), mRNA [NM_002016] | 4.448371 | 3.834523 | 0.613848 | 0.888223 |
| SPRR1A | Homo sapiens small proline-rich protein 1A (SPRR1A), transcript variant 2, mRNA [NM_005987] | 6.977213 | 6.363136 | 0.614077 | 0.888223 |
| IKZF1 | Homo sapiens IKAROS family zinc finger 1 (Ikaros) (IKZF1), transcript variant 1, mRNA [NM_006060] | 2.721654 | 2.106416 | 0.615238 | 0.888223 |
| ADCY1 | Homo sapiens adenylate cyclase 1 (brain) (ADCY1), mRNA [NM_021116] | 4.444942 | 3.828867 | 0.616075 | 0.888223 |
| TTL | Homo sapiens tubulin tyrosine ligase (TTL), mRNA [NM_153712] | 2.146273 | 1.529043 | 0.61723 | 0.888223 |
| THC2735154 | catenin (cadherin-associated protein), alpha 2 [Source:HGNC Symbol;Acc:2510] [ENST00000496251] | 2.015588 | 1.397591 | 0.617997 | 0.888223 |
| NRP2 | Homo sapiens neuropilin 2 (NRP2), transcript variant 6, mRNA [NM_201264] | 3.872805 | 3.25452 | 0.618285 | 0.888223 |
| TBX2 | Homo sapiens T-box 2 (TBX2), mRNA [NM_005994] | 3.495601 | 2.875256 | 0.620345 | 0.892865 |
| SNAI2 | Homo sapiens snail homolog 2 (Drosophila) (SNAI2), mRNA [NM_003068] | 6.704389 | 6.082832 | 0.621557 | 0.888223 |
| KATNB1 | Homo sapiens katanin p80 (WD repeat containing) subunit B 1 (KATNB1), mRNA [NM_005886] | 1.945924 | 1.322284 | 0.62364 | 0.888223 |
| C4orf31 | Homo sapiens neuron-derived neurotrophic factor (NDNF), mRNA [NM_024574] | 8.052731 | 7.428846 | 0.623885 | 0.888223 |
| ENST00000374440 | muscle, skeletal, receptor tyrosine kinase [Source:HGNC Symbol;Acc:7525] [ENST00000374440] | 1.98953 | 1.365121 | 0.624409 | 0.888223 |
| FASLG | Homo sapiens Fas ligand (TNF superfamily, member 6) (FASLG), mRNA [NM_000639] | 3.141654 | 2.516368 | 0.625287 | 0.888223 |
| VDR | Homo sapiens vitamin D (1,25- dihydroxyvitamin D3) receptor (VDR), transcript variant 2, mRNA [NM_001017535] | 6.068622 | 5.442396 | 0.626226 | 0.888223 |
| L1CAM | Homo sapiens L1 cell adhesion molecule (L1CAM), transcript variant 1, mRNA [NM_000425] | 3.748157 | 3.119675 | 0.628482 | 0.888223 |
| DMC1 | Homo sapiens DMC1 dosage suppressor of mck1 homolog, meiosis-specific homologous recombination (yeast) (DMC1), mRNA [NM_007068] | 5.926853 | 5.296305 | 0.630548 | 0.888223 |
| JMJD6 | Homo sapiens jumonji domain containing 6 (JMJD6), transcript variant 2, mRNA [NM_015167] | 5.121382 | 4.489847 | 0.631536 | 0.888223 |
| NPM2 | Homo sapiens nucleophosmin/nucleoplasmin 2 (NPM2), mRNA [NM_182795] | 2.646334 | 2.013734 | 0.632599 | 0.888223 |
| PTPRF | Homo sapiens protein tyrosine phosphatase, receptor type, F (PTPRF), transcript variant 1, mRNA [NM_002840] | 2.120599 | 1.487929 | 0.63267 | 0.888223 |
| CRP | Homo sapiens C-reactive protein, pentraxin-related (CRP), mRNA [NM_000567] | 2.122659 | 1.489312 | 0.633347 | 0.888223 |
| GFRA1 | Homo sapiens GDNF family receptor alpha 1 (GFRA1), transcript variant 1, mRNA [NM_005264] | 2.625747 | 1.992081 | 0.633667 | 0.888223 |
| AHSG | Homo sapiens alpha-2-HS-glycoprotein (AHSG), mRNA [NM_001622] | 2.145084 | 1.505531 | 0.639552 | 0.888223 |
| FOXA1 | Homo sapiens forkhead box A1 (FOXA1), mRNA [NM_004496] | 4.279469 | 3.637402 | 0.642067 | 0.888223 |
| HEG1 | Homo sapiens HEG homolog 1 (zebrafish) (HEG1), mRNA [NM_020733] | 4.131084 | 3.488491 | 0.642594 | 0.888223 |
| WNT2B | Homo sapiens wingless-type MMTV integration site family, member 2B (WNT2B), transcript variant WNT-2B1, mRNA [NM_004185] | 6.171866 | 5.529206 | 0.64266 | 0.888223 |
| DFNB31 | Homo sapiens deafness, autosomal recessive 31 (DFNB31), transcript variant 1, mRNA [NM_015404] | 6.54686 | 5.89924 | 0.647619 | 0.888223 |
| ENST00000409922 | chimerin (chimaerin) 2 [Source:HGNC Symbol;Acc:1944] [ENST00000409922] | 2.50463 | 1.85669 | 0.64794 | 0.888223 |
| HPS1 | Homo sapiens Hermansky-Pudlak syndrome 1 (HPS1), transcript variant 3, mRNA [NM_182639] | 2.636036 | 1.987874 | 0.648162 | 0.888223 |
| FNDC3B | Homo sapiens fibronectin type III domain containing 3B (FNDC3B), transcript variant 1, mRNA [NM_022763] | 2.956521 | 2.304744 | 0.651777 | 0.888223 |
| NRAS | Homo sapiens neuroblastoma RAS viral (v-ras) oncogene homolog (NRAS), mRNA [NM_002524] | 3.064778 | 2.410514 | 0.654265 | 0.888223 |
| A_19_P00318533 | Homo sapiens hook homolog 3 (Drosophila) (HOOK3), mRNA [NM_032410] | 2.012146 | 1.357196 | 0.65495 | 0.888223 |
| BOC | Homo sapiens Boc homolog (mouse) (BOC), mRNA [NM_033254] | 2.816733 | 2.159908 | 0.656824 | 0.900515 |
| CENPI | Homo sapiens centromere protein I (CENPI), mRNA [NM_006733] | 1.966682 | 1.30974 | 0.656942 | 0.888223 |
| ATF5 | Homo sapiens activating transcription factor 5 (ATF5), transcript variant 1, mRNA [NM_012068] | 2.678354 | 2.020383 | 0.657972 | 0.888223 |
| LTK | Homo sapiens leukocyte receptor tyrosine kinase (LTK), transcript variant 1, mRNA [NM_002344] | 5.017938 | 4.3598 | 0.658138 | 0.888223 |
| RP1L1 | Homo sapiens retinitis pigmentosa 1-like 1 (RP1L1), mRNA [NM_178857] | 2.679714 | 2.019997 | 0.659716 | 0.888223 |
| ERO1L | Homo sapiens ERO1-like (S. cerevisiae) (ERO1L), mRNA [NM_014584] | 5.226405 | 4.566263 | 0.660141 | 0.888223 |
| FGF1 | Homo sapiens fibroblast growth factor 1 (acidic) (FGF1), transcript variant 1, mRNA [NM_000800] | 1.986305 | 1.325985 | 0.66032 | 0.888223 |
| PPT1 | Homo sapiens palmitoyl-protein thioesterase 1 (PPT1), transcript variant 1, mRNA [NM_000310] | 2.464989 | 1.804098 | 0.660891 | 0.888223 |
| SS18L1 | Homo sapiens synovial sarcoma translocation gene on chromosome 18-like 1 (SS18L1), mRNA [NM_198935] | 2.051347 | 1.384984 | 0.666362 | 0.888223 |
| CCL5 | Homo sapiens chemokine (C-C motif) ligand 5 (CCL5), mRNA [NM_002985] | 3.485082 | 2.816277 | 0.668805 | 0.888223 |
| CXCR4 | Homo sapiens chemokine (C-X-C motif) receptor 4 (CXCR4), transcript variant 1, mRNA [NM_001008540] | 3.659808 | 2.990959 | 0.668849 | 0.888223 |
| DLG4 | Homo sapiens discs, large homolog 4 (Drosophila) (DLG4), transcript variant 1, mRNA [NM_001365] | 3.403445 | 2.734229 | 0.669216 | 0.888223 |
| HAND1 | Homo sapiens heart and neural crest derivatives expressed 1 (HAND1), mRNA [NM_004821] | 4.30674 | 3.636922 | 0.669818 | 0.888223 |
| CLIC5 | Homo sapiens chloride intracellular channel 5 (CLIC5), nuclear gene encoding mitochondrial protein, transcript variant 2, mRNA [NM_016929] | 2.040075 | 1.369502 | 0.670573 | 0.888223 |
| FGF10 | Homo sapiens fibroblast growth factor 10 (FGF10), mRNA [NM_004465] | 3.506685 | 2.835032 | 0.671653 | 0.888223 |
| CBFA2T2 | Homo sapiens core-binding factor, runt domain, alpha subunit 2; translocated to, 2 (CBFA2T2), transcript variant 2, mRNA [NM_005093] | 2.675936 | 2.004067 | 0.671869 | 0.888223 |
| ENST00000330655 | Homo sapiens myeloid-associated differentiation marker-like 2 (MYADML2), mRNA [NM_001145113] | 3.393607 | 2.720947 | 0.672661 | 0.888223 |
| C19orf20 | Homo sapiens chromosome 19 open reading frame 20 (C19orf20), mRNA [NM_033513] | 1.983087 | 1.31035 | 0.672737 | 0.888223 |
| CLN5 | Homo sapiens ceroid-lipofuscinosis, neuronal 5 (CLN5), mRNA [NM_006493] | 2.464202 | 1.79107 | 0.673132 | 0.888223 |
| TSSK1B | Homo sapiens testis-specific serine kinase 1B (TSSK1B), mRNA [NM_032028] | 2.043655 | 1.370487 | 0.673168 | 0.888223 |
| BAIAP2 | Homo sapiens BAI1-associated protein 2 (BAIAP2), transcript variant 3, mRNA [NM_006340] | 4.076755 | 3.402921 | 0.673834 | 0.888223 |
| BIN1 | Homo sapiens bridging integrator 1 (BIN1), transcript variant 4, mRNA [NM_139346] | 4.629346 | 3.95548 | 0.673866 | 0.888223 |
| RIBC1 | Homo sapiens hydroxysteroid (17-beta) dehydrogenase 10 (HSD17B10), nuclear gene encoding mitochondrial protein, transcript variant 1, mRNA [NM_004493] | 3.175736 | 2.495504 | 0.680233 | 0.888223 |
| PVRL1 | Homo sapiens poliovirus receptor-related 1 (herpesvirus entry mediator C) (PVRL1), transcript variant 3, mRNA [NM_203286] | 4.439447 | 3.75789 | 0.681556 | 0.888223 |
| IL12B | Homo sapiens interleukin 12B (natural killer cell stimulatory factor 2, cytotoxic lymphocyte maturation factor 2, p40) (IL12B), mRNA [NM_002187] | 2.413855 | 1.731154 | 0.682701 | 0.888223 |
| CELSR1 | Homo sapiens cadherin, EGF LAG seven-pass G-type receptor 1 (flamingo homolog, Drosophila) (CELSR1), mRNA [NM_014246] | 2.375167 | 1.691862 | 0.683305 | 0.888223 |
| HDAC9 | Homo sapiens histone deacetylase 9 (HDAC9), transcript variant 1, mRNA [NM_058176] | 2.074499 | 1.390101 | 0.684398 | 0.888223 |
| PROP1 | Homo sapiens PROP paired-like homeobox 1 (PROP1), mRNA [NM_006261] | 3.65185 | 2.966824 | 0.685027 | 0.888223 |
| PPP2R5B | BROAD Institute lincRNA (XLOC_009164), lincRNA [TCONS_00019147] | 4.094882 | 3.408946 | 0.685936 | 0.916701 |
| PSEN1 | Homo sapiens presenilin 1 (PSEN1), transcript variant 1, mRNA [NM_000021] | 2.037348 | 1.351377 | 0.685971 | 0.888223 |
| BOK | Homo sapiens BCL2-related ovarian killer (BOK), mRNA [NM_032515] | 4.099544 | 3.404629 | 0.694915 | 0.9238 |
| GHRHR | GB | 3.237566 | 2.542372 | 0.695194 | 0.888223 |
| FGF7 | Homo sapiens fibroblast growth factor 7 (FGF7), mRNA [NM_002009] | 2.561789 | 1.865564 | 0.696225 | 0.888223 |
| CLU | Homo sapiens clusterin (CLU), transcript variant 1, mRNA [NM_001831] | 2.186193 | 1.489227 | 0.696966 | 0.888223 |
| DPYSL4 | Homo sapiens dihydropyrimidinase-like 4 (DPYSL4), mRNA [NM_006426] | 4.458184 | 3.76044 | 0.697745 | 0.889199 |
| CHN2 | chimerin (chimaerin) 2 [Source:HGNC Symbol;Acc:1944] [ENST00000410098] | 6.254917 | 5.555376 | 0.699541 | 0.888223 |
| ATP2B2 | Homo sapiens ATPase, Ca++ transporting, plasma membrane 2 (ATP2B2), transcript variant 1, mRNA [NM_001001331] | 4.114875 | 3.408987 | 0.705888 | 0.888223 |
| SFRP4 | Homo sapiens secreted frizzled-related protein 4 (SFRP4), mRNA [NM_003014] | 3.360552 | 2.652481 | 0.708072 | 0.888223 |
| ENST00000403441 | preferentially expressed antigen in melanoma [Source:HGNC Symbol;Acc:9336] [ENST00000403441] | 2.263963 | 1.550931 | 0.713032 | 0.888223 |
| A_33_P3228828 | protein tyrosine phosphatase, non-receptor type 11 [Source:HGNC Symbol;Acc:9644] [ENST00000531326] | 2.410612 | 1.697083 | 0.71353 | 0.888223 |
| CASC5 | Homo sapiens cancer susceptibility candidate 5 (CASC5), transcript variant 1, mRNA [NM_170589] | 3.748247 | 3.033891 | 0.714356 | 0.888223 |
| CARD11 | Homo sapiens caspase recruitment domain family, member 11 (CARD11), mRNA [NM_032415] | 4.209206 | 3.491666 | 0.71754 | 0.888223 |
| NAB1 | Homo sapiens NGFI-A binding protein 1 (EGR1 binding protein 1) (NAB1), mRNA [NM_005966] | 2.124597 | 1.405455 | 0.719142 | 0.888223 |
| FEZF2 | Homo sapiens FEZ family zinc finger 2 (FEZF2), mRNA [NM_018008] | 2.265589 | 1.546084 | 0.719505 | 0.888223 |
| ADAM8 | Homo sapiens ADAM metallopeptidase domain 8 (ADAM8), transcript variant 1, mRNA [NM_001109] | 4.660394 | 3.937118 | 0.723276 | 0.888223 |
| IRF8 | Homo sapiens interferon regulatory factor 8 (IRF8), mRNA [NM_002163] | 2.151875 | 1.42641 | 0.725465 | 0.888223 |
| RPS6KA6 | Homo sapiens ribosomal protein S6 kinase, 90kDa, polypeptide 6 (RPS6KA6), mRNA [NM_014496] | 2.053049 | 1.324246 | 0.728802 | 0.888223 |
| SSH3 | Homo sapiens slingshot homolog 3 (Drosophila) (SSH3), mRNA [NM_017857] | 4.506046 | 3.776648 | 0.729398 | 0.888223 |
| GFI1B | Homo sapiens growth factor independent 1B transcription repressor (GFI1B), transcript variant 1, mRNA [NM_004188] | 3.792881 | 3.063228 | 0.729653 | 0.888223 |
| F2R | Homo sapiens coagulation factor II (thrombin) receptor, mRNA (cDNA clone IMAGE:4849569), with apparent retained intron. [BC016059] | 2.84299 | 2.110015 | 0.732974 | 0.888223 |
| ENST00000393237 | c-mer proto-oncogene tyrosine kinase [Source:HGNC Symbol;Acc:7027] [ENST00000393237] | 2.022559 | 1.28881 | 0.73375 | 0.888223 |
| PITX2 | Homo sapiens paired-like homeodomain 2 (PITX2), transcript variant 2, mRNA [NM_153426] | 2.740379 | 2.005887 | 0.734491 | 0.888223 |
| TP53 | Homo sapiens tumor protein p53 (TP53), transcript variant 1, mRNA [NM_000546] | 5.292876 | 4.557982 | 0.734894 | 0.888223 |
| MAP2K2 | Homo sapiens mitogen-activated protein kinase kinase 2 (MAP2K2), mRNA [NM_030662] | 2.243274 | 1.506585 | 0.736689 | 0.888223 |
| ENST00000342645 | homeodomain interacting protein kinase 2 [Source:HGNC Symbol;Acc:14402] [ENST00000342645] | 3.235714 | 2.498534 | 0.73718 | 0.888223 |
| RPS14 | Homo sapiens ribosomal protein S14 (RPS14), transcript variant 3, mRNA [NM_005617] | 2.049133 | 1.310359 | 0.738774 | 0.888223 |
| MYST3 | Homo sapiens K(lysine) acetyltransferase 6A (KAT6A), transcript variant 1, mRNA [NM_001099412] | 4.267147 | 3.528205 | 0.738942 | 0.888223 |
| DYNLT1 | Homo sapiens dynein, light chain, Tctex-type 1 (DYNLT1), mRNA [NM_006519] | 4.817542 | 4.077564 | 0.739977 | 0.888223 |
| MTCH1 | Homo sapiens mitochondrial carrier 1 (MTCH1), nuclear gene encoding mitochondrial protein, mRNA [NM_014341] | 2.189759 | 1.441284 | 0.748475 | 0.888223 |
| SLITRK2 | Homo sapiens SLIT and NTRK-like family, member 2 (SLITRK2), transcript variant 1, mRNA [NM_032539] | 5.235337 | 4.483429 | 0.751908 | 0.888223 |
| FOXJ1 | Homo sapiens forkhead box J1 (FOXJ1), mRNA [NM_001454] | 3.37302 | 2.620892 | 0.752127 | 0.888223 |
| BAIAP2 | Homo sapiens BAI1-associated protein 2 (BAIAP2), transcript variant 2, mRNA [NM_017451] | 3.513706 | 2.761501 | 0.752205 | 0.888223 |
| POU3F2 | Homo sapiens POU class 3 homeobox 2 (POU3F2), mRNA [NM_005604] | 3.727083 | 2.973213 | 0.75387 | 0.888223 |
| MYLK2 | Homo sapiens myosin light chain kinase 2 (MYLK2), mRNA [NM_033118] | 2.598296 | 1.841625 | 0.756671 | 0.888223 |
| RRAS | Homo sapiens related RAS viral (r-ras) oncogene homolog (RRAS), mRNA [NM_006270] | 3.718123 | 2.959179 | 0.758944 | 0.888223 |
| MGMT | Homo sapiens O-6-methylguanine-DNA methyltransferase (MGMT), mRNA [NM_002412] | 3.880481 | 3.120847 | 0.759634 | 0.888223 |
| RUNX1 | Homo sapiens runt-related transcription factor 1 (RUNX1), transcript variant 3, mRNA [NM_001122607] | 5.046753 | 4.286795 | 0.759958 | 0.888223 |
| MINK1 | Homo sapiens misshapen-like kinase 1 (MINK1), transcript variant 3, mRNA [NM_153827] | 2.099133 | 1.335928 | 0.763204 | 0.888223 |
| ACTB | Homo sapiens actin, beta (ACTB), mRNA [NM_001101] | 3.520719 | 2.757428 | 0.763291 | 0.888223 |
| MYH14 | Homo sapiens myosin, heavy chain 14, non-muscle (MYH14), transcript variant 1, mRNA [NM_001077186] | 4.610767 | 3.841032 | 0.769736 | 0.888223 |
| ZFP36L1 | Homo sapiens zinc finger protein 36, C3H type-like 1 (ZFP36L1), transcript variant 1, mRNA [NM_004926] | 2.657327 | 1.886673 | 0.770653 | 0.888223 |
| KIF4A | Homo sapiens kinesin family member 4A (KIF4A), mRNA [NM_012310] | 3.184239 | 2.412209 | 0.772031 | 0.888223 |
| POU3F3 | Homo sapiens POU class 3 homeobox 3 (POU3F3), mRNA [NM_006236] | 2.794068 | 2.019925 | 0.774143 | 0.888223 |
| SCXA | Homo sapiens scleraxis homolog A (mouse) (SCXA), mRNA [NM_001008271] | 4.223828 | 3.449193 | 0.774636 | 0.888223 |
| C20orf149 | Homo sapiens pancreatic progenitor cell differentiation and proliferation factor homolog (zebrafish) (PPDPF), mRNA [NM_024299] | 3.437076 | 2.660869 | 0.776207 | 0.888223 |
| PPL | Homo sapiens periplakin (PPL), mRNA [NM_002705] | 2.489567 | 1.711581 | 0.777987 | 0.888223 |
| STK4 | Homo sapiens serine/threonine kinase 4 (STK4), mRNA [NM_006282] | 2.606111 | 1.825339 | 0.780772 | 0.888223 |
| MYO7A | Homo sapiens myosin VIIA (MYO7A), transcript variant 1, mRNA [NM_000260] | 2.226188 | 1.444048 | 0.78214 | 0.888223 |
| TBX19 | Homo sapiens T-box 19 (TBX19), mRNA [NM_005149] | 4.041478 | 3.250978 | 0.7905 | 0.888223 |
| RANBP9 | Homo sapiens RAN binding protein 9 (RANBP9), mRNA [NM_005493] | 2.255116 | 1.463954 | 0.791163 | 0.888223 |
| ANKRD54 | Homo sapiens ankyrin repeat domain 54 (ANKRD54), transcript variant 1, mRNA [NM_138797] | 2.235006 | 1.441519 | 0.793486 | 0.888223 |
| HES3 | Homo sapiens hairy and enhancer of split 3 (Drosophila) (HES3), mRNA [NM_001024598] | 3.4621 | 2.667466 | 0.794634 | 0.888223 |
| GDNF | Homo sapiens glial cell derived neurotrophic factor (GDNF), transcript variant 3, mRNA [NM_001190468] | 3.191561 | 2.395865 | 0.795696 | 0.888223 |
| PSPN | Homo sapiens persephin (PSPN), mRNA [NM_004158] | 3.567827 | 2.771992 | 0.795836 | 0.894442 |
| GDF10 | Homo sapiens growth differentiation factor 10 (GDF10), mRNA [NM_004962] | 2.156965 | 1.354711 | 0.802253 | 0.888223 |
| CTNNA1 | Homo sapiens catenin (cadherin-associated protein), alpha 1, 102kDa (CTNNA1), mRNA [NM_001903] | 2.523077 | 1.720523 | 0.802554 | 0.888223 |
| MITF | Homo sapiens microphthalmia-associated transcription factor (MITF), transcript variant 1, mRNA [NM_198159] | 3.605758 | 2.802047 | 0.803712 | 0.888223 |
| THC2656683 | sprouty homolog 1, antagonist of FGF signaling (Drosophila) [Source:HGNC Symbol;Acc:11269] [ENST00000505319] | 3.358286 | 2.553257 | 0.805029 | 0.888223 |
| NOS3 | Homo sapiens nitric oxide synthase 3 (endothelial cell) (NOS3), transcript variant 1, mRNA [NM_000603] | 4.400415 | 3.590811 | 0.809604 | 0.888223 |
| GCM1 | Homo sapiens glial cells missing homolog 1 (Drosophila) (GCM1), mRNA [NM_003643] | 2.633453 | 1.823562 | 0.809891 | 0.888223 |
| CA2 | Homo sapiens carbonic anhydrase II (CA2), mRNA [NM_000067] | 5.58656 | 4.774423 | 0.812137 | 0.888223 |
| ENST00000368941 | FIG4 homolog, SAC1 lipid phosphatase domain containing (S. cerevisiae) [Source:HGNC Symbol;Acc:16873] [ENST00000368941] | 2.42838 | 1.615891 | 0.812489 | 0.888223 |
| EN1 | Homo sapiens engrailed homeobox 1 (EN1), mRNA [NM_001426] | 5.292467 | 4.479726 | 0.81274 | 0.888223 |
| CRP | Homo sapiens C-reactive protein, pentraxin-related (CRP), mRNA [NM_000567] | 2.41025 | 1.593149 | 0.817102 | 0.888223 |
| GHRHR | Homo sapiens growth hormone releasing hormone receptor (GHRHR), mRNA [NM_000823] | 2.138455 | 1.316714 | 0.821742 | 0.888223 |
| NDFIP1 | Homo sapiens Nedd4 family interacting protein 1 (NDFIP1), mRNA [NM_030571] | 4.884077 | 4.061735 | 0.822342 | 0.888223 |
| EPHA1 | Homo sapiens EPH receptor A1 (EPHA1), mRNA [NM_005232] | 5.800421 | 4.977701 | 0.82272 | 0.888223 |
| MALL | Homo sapiens mal, T-cell differentiation protein-like (MALL), mRNA [NM_005434] | 2.172198 | 1.347267 | 0.824932 | 0.888223 |
| TMEM99 | Homo sapiens keratin 10 (KRT10), mRNA [NM_000421] | 4.81084 | 3.985861 | 0.824979 | 0.888223 |
| KRT36 | Homo sapiens keratin 36 (KRT36), mRNA [NM_003771] | 2.675393 | 1.846992 | 0.8284 | 0.888223 |
| DISC1 | Homo sapiens disrupted in schizophrenia 1 (DISC1), transcript variant q, mRNA [NM_001164554] | 2.622629 | 1.793055 | 0.829574 | 0.888223 |
| FANCG | Homo sapiens Fanconi anemia, complementation group G (FANCG), mRNA [NM_004629] | 4.709299 | 3.878694 | 0.830605 | 0.888223 |
| ATF2 | Homo sapiens activating transcription factor 2 (ATF2), mRNA [NM_001880] | 2.809518 | 1.975995 | 0.833523 | 0.888223 |
| PRTFDC1 | Homo sapiens phosphoribosyl transferase domain containing 1 (PRTFDC1), mRNA [NM_020200] | 2.128398 | 1.289453 | 0.838945 | 0.888223 |
| CACNB3 | Homo sapiens calcium channel, voltage-dependent, beta 3 subunit (CACNB3), transcript variant 1, mRNA [NM_000725] | 2.548555 | 1.705503 | 0.843052 | 0.888223 |
| ERBB3 | Homo sapiens v-erb-b2 erythroblastic leukemia viral oncogene homolog 3 (avian) (ERBB3), transcript variant s, mRNA [NM_001005915] | 2.145055 | 1.299513 | 0.845541 | 0.888223 |
| HILS1 | Homo sapiens histone linker H1 domain, spermatid-specific 1 (HILS1), transcript variant 1, non-coding RNA [NR_024193] | 4.634281 | 3.788424 | 0.845856 | 0.888223 |
| MFAP2 | Homo sapiens microfibrillar-associated protein 2 (MFAP2), transcript variant 1, mRNA [NM_017459] | 2.752903 | 1.90703 | 0.845872 | 0.888223 |
| CAPZA3 | Homo sapiens capping protein (actin filament) muscle Z-line, alpha 3 (CAPZA3), mRNA [NM_033328] | 2.624636 | 1.774122 | 0.850513 | 0.888223 |
| LCE3A | Homo sapiens late cornified envelope 3A (LCE3A), mRNA [NM_178431] | 4.647745 | 3.794628 | 0.853117 | 0.888223 |
| SPTB | Homo sapiens spectrin, beta, erythrocytic (SPTB), transcript variant 1, mRNA [NM_001024858] | 3.075251 | 2.221829 | 0.853422 | 0.888223 |
| NKX2-8 | Homo sapiens NK2 homeobox 8 (NKX2-8), mRNA [NM_014360] | 3.140858 | 2.286178 | 0.85468 | 0.888223 |
| VAPA | Homo sapiens VAMP (vesicle-associated membrane protein)-associated protein A, 33kDa (VAPA), transcript variant 1, mRNA [NM_003574] | 2.259419 | 1.401027 | 0.858391 | 0.888223 |
| EPHA7 | Homo sapiens EPH receptor A7 (EPHA7), mRNA [NM_004440] | 2.169466 | 1.308979 | 0.860488 | 0.888223 |
| CACNA1H | Homo sapiens calcium channel, voltage-dependent, T type, alpha 1H subunit (CACNA1H), transcript variant 1, mRNA [NM_021098] | 7.074658 | 6.206949 | 0.867709 | 0.888223 |
| TRIM15 | Homo sapiens tripartite motif containing 15 (TRIM15), mRNA [NM_033229] | 2.290524 | 1.422559 | 0.867965 | 0.888223 |
| RYK | Homo sapiens RYK receptor-like tyrosine kinase (RYK), transcript variant 1, mRNA [NM_001005861] | 4.008067 | 3.139237 | 0.868831 | 0.888223 |
| LRRC17 | Homo sapiens leucine rich repeat containing 17 (LRRC17), transcript variant 2, mRNA [NM_005824] | 2.698289 | 1.825051 | 0.873238 | 0.888223 |
| FGF19 | Homo sapiens fibroblast growth factor 19 (FGF19), mRNA [NM_005117] | 2.260672 | 1.38739 | 0.873282 | 0.888223 |
| PRTFDC1 | Homo sapiens phosphoribosyl transferase domain containing 1 (PRTFDC1), mRNA [NM_020200] | 3.673347 | 2.797239 | 0.876108 | 0.888223 |
| NAPA | Homo sapiens N-ethylmaleimide-sensitive factor attachment protein, alpha (NAPA), transcript variant 1, mRNA [NM_003827] | 3.199757 | 2.322079 | 0.877678 | 0.888223 |
| CSDA | Homo sapiens cold shock domain protein A (CSDA), transcript variant 1, mRNA [NM_003651] | 4.336515 | 3.45637 | 0.880145 | 0.888223 |
| TCF7L2 | Homo sapiens transcription factor 7-like 2 (T-cell specific, HMG-box) (TCF7L2), transcript variant 2, mRNA [NM_030756] | 5.117643 | 4.235776 | 0.881867 | 0.888223 |
| PTPN11 | Homo sapiens protein tyrosine phosphatase, non-receptor type 11 (PTPN11), mRNA [NM_002834] | 3.308806 | 2.424905 | 0.8839 | 0.888223 |
| MAFB | Homo sapiens v-maf musculoaponeurotic fibrosarcoma oncogene homolog B (avian) (MAFB), mRNA [NM_005461] | 4.399396 | 3.512859 | 0.886537 | 0.888223 |
| HOXD11 | Homo sapiens homeobox D11 (HOXD11), mRNA [NM_021192] | 3.750456 | 2.86294 | 0.887517 | 0.888223 |
| PLEKHA1 | Homo sapiens pleckstrin homology domain containing, family A (phosphoinositide binding specific) member 1 (PLEKHA1), transcript variant 2, mRNA [NM_001001974] | 5.594813 | 4.702607 | 0.892207 | 0.888223 |
| HLX | Homo sapiens H2.0-like homeobox (HLX), mRNA [NM_021958] | 2.427043 | 1.528662 | 0.898381 | 0.888223 |
| IMMP2L | Homo sapiens IMP2 inner mitochondrial membrane peptidase-like (S. cerevisiae) (IMMP2L), nuclear gene encoding mitochondrial protein, transcript variant 1, mRNA [NM_032549] | 3.306254 | 2.405925 | 0.90033 | 0.888223 |
| BRSK1 | Homo sapiens BR serine/threonine kinase 1 (BRSK1), mRNA [NM_032430] | 6.714935 | 5.813102 | 0.901833 | 0.888223 |
| PSAP | Homo sapiens prosaposin (PSAP), transcript variant 2, mRNA [NM_001042465] | 2.193069 | 1.291144 | 0.901925 | 0.888223 |
| A_19_P00322911 | Homo sapiens twist homolog 2 (Drosophila) (TWIST2), mRNA [NM_057179] | 3.361865 | 2.45851 | 0.903355 | 0.888223 |
| RGS2 | Homo sapiens regulator of G-protein signaling 2, 24kDa (RGS2), mRNA [NM_002923] | 2.618136 | 1.713058 | 0.905078 | 0.888223 |
| KLK8 | Homo sapiens kallikrein-related peptidase 8 (KLK8), transcript variant 2, mRNA [NM_144505] | 2.444223 | 1.53336 | 0.910863 | 0.888223 |
| NAGLU | Homo sapiens N-acetylglucosaminidase, alpha (NAGLU), mRNA [NM_000263] | 2.293673 | 1.382286 | 0.911387 | 0.888223 |
| HOXD9 | Homo sapiens homeobox D9 (HOXD9), mRNA [NM_014213] | 2.760508 | 1.844712 | 0.915796 | 0.888223 |
| FNDC3A | fibronectin type III domain containing 3A [Source:HGNC Symbol;Acc:20296] [ENST00000378383] | 4.029305 | 3.110646 | 0.918659 | 0.888223 |
| PTF1A | Homo sapiens pancreas specific transcription factor, 1a (PTF1A), mRNA [NM_178161] | 3.120611 | 2.199362 | 0.921249 | 0.888223 |
| EPHA2 | Homo sapiens EPH receptor A2 (EPHA2), mRNA [NM_004431] | 2.307069 | 1.384753 | 0.922316 | 0.888223 |
| DVL1 | Homo sapiens dishevelled, dsh homolog 1 (Drosophila) (DVL1), mRNA [NM_004421] | 2.348925 | 1.424963 | 0.923962 | 0.888223 |
| NCOR1 | Homo sapiens nuclear receptor corepressor 1 (NCOR1), transcript variant 1, mRNA [NM_006311] | 2.496706 | 1.57103 | 0.925676 | 0.888223 |
| PAX6 | Homo sapiens paired box 6 (PAX6), transcript variant 1, mRNA [NM_000280] | 2.475742 | 1.545891 | 0.929851 | 0.888223 |
| EDN3 | Homo sapiens endothelin 3 (EDN3), transcript variant 2, mRNA [NM_207032] | 2.328438 | 1.393252 | 0.935186 | 0.888223 |
| WNT2 | Homo sapiens wingless-type MMTV integration site family member 2 (WNT2), transcript variant 1, mRNA [NM_003391] | 3.117961 | 2.179376 | 0.938585 | 0.888223 |
| CABP4 | Homo sapiens calcium binding protein 4 (CABP4), mRNA [NM_145200] | 3.252625 | 2.307952 | 0.944673 | 0.888223 |
| THBS4 | BROAD Institute lincRNA (XLOC_l2_011649), lincRNA [TCONS_l2_00022386] | 3.851744 | 2.905215 | 0.946529 | 0.888223 |
| PTK6 | Homo sapiens PTK6 protein tyrosine kinase 6 (PTK6), mRNA [NM_005975] | 6.460016 | 5.511678 | 0.948337 | 0.888223 |
| CSTA | Homo sapiens cystatin A (stefin A) (CSTA), mRNA [NM_005213] | 3.157541 | 2.20827 | 0.949271 | 0.888223 |
| SOHLH1 | Homo sapiens spermatogenesis and oogenesis specific basic helix-loop-helix 1 | 2.969553 | 1.933203 | 1.03635 | 0.888223 |
| DDX4 | Homo sapiens DEAD (Asp-Glu-Ala-Asp) box polypeptide 4 | 2.652419 | 1.411478 | 1.264188 | 0.888223 |
| DDX31 | Homo sapiens DEAD (Asp-Glu-Ala-Asp) box polypeptide 31 | 3.35977 | 2.044286 | 1.315484 | 0.888223 |
| SYCP3 | Homo sapiens synaptonemal complex protein 3 | 7.107678 | 4.741879 | 2.365799 | 0.888223 |
| DDX3Y | Homo sapiens DEAD (Asp-Glu-Ala-Asp) box polypeptide 3, Y-linked (DDX3Y) | 9.0877 | 4.402031 | 4.685669 | 0.987713 |
